# A phase I/II trial of WT1-specific TCR gene therapy for patients with acute myeloid leukemia and active disease post-allogeneic hematopoietic cell transplantation: skewing towards NK-like phenotype impairs T cell function and persistence

**DOI:** 10.1038/s41467-025-60394-0

**Published:** 2025-06-05

**Authors:** Francesco Mazziotta, Lauren E. Martin, Daniel N. Egan, Merav Bar, Sinéad Kinsella, Kelly G. Paulson, Valentin Voillet, Miranda C. Lahman, Daniel Hunter, Thomas M. Schmitt, Natalie Duerkopp, Cecilia C. S. Yeung, Tzu-Hao Tang, Raphael Gottardo, Yuta Asano, Elise C. Wilcox, Bo Lee, Tianzi Zhang, Paolo Lopedote, Livius Penter, Catherine J. Wu, Filippo Milano, Philip D. Greenberg, Aude G. Chapuis

**Affiliations:** 1 Program in Immunology, Fred Hutchinson Cancer Center, Seattle, WA USA; 2 Translational Sciences and Therapeutics Division, Fred Hutchinson Cancer Center, Seattle, WA USA; 3 Immunotherapy Integrated Research Center, Fred Hutch Cancer Center, Seattle, WA USA; 4 Division of Medical Oncology, University of Washington, Seattle, WA USA; 5 Providence-Swedish Cancer Institute, Seattle, WA USA; 6 Bristol Myers Squibb, Boudry, Switzerland; 7 Vaccine and Infectious Disease Division, Fred Hutchinson Cancer Center, Seattle, WA USA; 8 Cape Town HVTN Immunology Laboratory, Hutchinson Centre Research Institute of South Africa, Cape Town, South Africa; 9 University of Washington, Dept. of Laboratory Medicine and Pathology, Seattle, WA USA; 10 Biomedical Data Science Center, Lausanne University Hospital, Lausanne, Switzerland; 11 University of Lausanne, Lausanne, Switzerland; 12 Agora Translational Research Center, Lausanne, Switzerland; 13 Swiss Institute of Bioinformatics, Lausanne, Switzerland; 14 Department of Medicine, St. Elizabeth’s Medical Center, Boston University, Boston, MA USA; 15 Department of Medical Oncology, Dana-Farber Cancer Institute, Boston, MA USA; 16 Department of Hematology, Oncology, and Tumorimmunology, Campus Virchow Klinikum, Charité - Universitätsmedizin Berlin, Corporate Member of Freie Universität Berlin and Humboldt-Universität zu Berlin, Berlin, Germany; 17 Berlin Institute of Health at Charité-Universitätsmedizin Berlin, BIH Biomedical Innovation Academy, BIH Charité Digital Clinician Scientist Program, Berlin, Germany; 18 Departments of Immunology and Medicine, University of Washington, Seattle, WA USA

**Keywords:** Translational immunology, Cancer

## Abstract

Relapsed and/or refractory acute myeloid leukemia (AML) post-allogeneic hematopoietic cell transplantation (HCT) is usually fatal. We previously reported that post-HCT immunotherapy with Epstein-Barr virus (EBV)-specific donor CD8^+^ T cells engineered to express a Wilms Tumor Antigen 1-specific T-cell receptor (T_TCR-C4_) appeared to prevent relapse in high-risk patients. In this phase I/II clinical trial (NCT01640301), we evaluated safety (primary endpoint), persistence and efficacy (secondary endpoints) of EBV- or Cytomegalovirus (CMV)-specific T_TCR-C4_ in fifteen patients with active AML post-HCT. Infusions were well tolerated, with no dose-limiting toxicities or serious adverse events related to the product. However, T_TCR-C4_ cells did not clearly improve outcomes despite EBV-specific T_TCR-C4_ cells showing enhanced potential for prolonged persistence compared to CMV-specific T_TCR-C4_. Investigating the fate of persisting T_TCR-C4_, we identified a shift towards natural killer-like (NKL) terminal differentiation, distinct from solid tumor-associated canonical exhaustion programs. In one patient, treatment with azacitidine appeared to mitigate this NKL skewing, promoting T_TCR-C4_ persistence. These findings suggest that AML drives a distinct form of T-cell dysfunction, highlight the need for targeted approaches that preserve T-cell fitness, ultimately improving the efficacy of cellular therapies for AML.

## Abstract (web summary)

Relapsed and/or refractory acute myeloid leukemia (AML) postallogeneic hematopoietic cell transplantation has limited treatment options. Here the authors report the clinical results and immune correlates of a phase I/II trial of adoptively transferred virus-specific donor CD8 + T cells engineered to express a WT1-specific T cell receptor in patients with acute myeloid leukemia and active disease post-allogeneic hematopoietic cell transplantation.

## Introduction

Relapsed/refractory acute myeloid leukemia (AML) after allogeneic hematopoietic cell transplantation (HCT) poses a major therapeutic challenge^1,2^, with a 2-year overall survival (OS) rate below 20% and only 4% if relapse occurs within six months post-HCT^1–3^. Salvage therapies, including intensive chemotherapy, donor lymphocyte infusions (DLI), and second HCTs have shown limited efficacy, underscoring the urgent need for novel therapies.

Pioneering trials^4–7^ have demonstrated that adoptive T-cell therapy targeting Epstein-Barr virus (EBV) or cytomegalovirus (CMV) effectively treats post-transplant lymphoproliferative disease, thereby establishing antigen-specific T-cell therapies as a viable approach post-transplant. Building on this foundation, various efforts have sought to extend cell therapy to target tumors. The Wilms’ Tumor 1 (WT1) protein is an attractive AML immunotherapy target^8^, as WT1 overexpression promotes proliferation and oncogenicity^9–11^. In a phase I/II trial, targeting WT1 in the post-HCT setting, we genetically modified matched donor CD8^+^ T cells to express a high-affinity WT1-specific T-cell receptor (TCR_C4_) specific for the HLA-A*0201-restricted WT1_126-134_ epitope. To minimize the potential for graft-versus-host disease (GVHD) mediated by infused donor cells^12^, EBV-specific or CMV-specific CD8^+^ T cells were transduced (T_TCR-C4_). Prophylactic infusion of EBV-specific T_TCR-C4_ in patients without detectable disease, but at high relapse risk post-transplant (Arm 1), yielded 100% relapse-free survival in 12 patients at a median follow-up of 44 months^13^. Although the trial was not randomized, this strategy showed an advantage when compared to historical controls who did not receive the same treatment. However, in 15 patients with prior evidence of disease post-HCT discussed here, infused EBV-specific or CMV-specific T_TCR-C4_ did not yield a superior overall survival compared to historical controls^1,2,14,15^.

The persistence of functional antigen-specific T cells is required for sustained immunotherapy efficacy^16–18^. In solid tumors and lymphomas, reduced persistence generally correlates with T-cell exhaustion^16,19^, characterized by markers like PD-1, CTLA-4, Tim3, LAG-3, BTLA and/or TIGIT^20^. Targeting these immune-inhibitory receptors with checkpoint-blocking antibodies mitigates exhaustion^21,22^. In AML, recent studies have questioned the presence of T-cell exhaustion^23^, proposing alternative mechanisms of immune dysfunction^24,25^. Natural killer-like (NKL) markers expressed on CD8^+^ T cells have correlated with adverse outcomes, suggesting that NKL skewing may contribute to T-cell dysfunction^24–26^. However, whether AML directly induces terminally differentiated, dysfunctional antigen-specific T cells remains unclear^27^.

Understanding the mechanisms of dysfunction that are operative in AML is critical for designing strategies to overcome the current limitations observed in T-cell therapies. Here, we leveraged adoptive transfer of T_TCR-C4_ in refractory or relapsed AML patients to track AML-specific T cells, elucidate AML-induced T cell states, identify the mechanisms responsible for T-cell dysfunction, and inform the design of effective anti-AML therapies.

## Results

### T_TCR-C4_ is safe, well tolerated, and produces comparable outcomes to conventional treatments for high-risk relapsed/refractory AML patients post-HCT

From April 2013 through February 2019, 15 HLA-A2-expressing patients with relapsed and/or refractory AML post-HCT were enrolled on trial NCT01640301 (Table 1, and Supplementary Fig. 1). The median age at diagnosis was 40. Pre-HCT, 27% of patients had secondary or treatment-related AML, at diagnosis 47% were adverse risk, and 53% favorable/intermediate risk per European LeukemiaNet (ELN) stratification (Supplementary Table 1)^28^. Post-HCT, four patients had measurable residual disease (MRD) by ~28 days (refractory), two relapsed by 3 months, and nine relapsed after day 100. The median time to relapse was 496 days post-HCT. Thirteen patients received salvage therapy before T_TCR-C4_, including six who underwent a second HCT. Within a median of two weeks before infusion, two patients had overt disease, five were MRD-positive and the rest had no evaluable disease (NED) (Supplementary Fig. 2). Ten patients received EBV-specific T_TCR-C4_ and five received CMV-specific T cells (Table 1). Patients received one to four infusions depending on their place in the T_TCR-C4_ dose escalation (Supplementary Fig. 3).

**Table 1:** Clinical characteristics of AML patients receiving T_TCR-C4_ infusions

Pt	AML WT1 expression	Disease status 28 days post-HCT	Number of HCT before T_TCR-C4_ infusion	Salvage therapy before T_TCR-C4_ infusion	Lymphodepletion	Disease status at 1^st^ T_TCR-C4_ infusion	Days between salvage and T_TCR-C4_ infusion	Virus specificity	T_TCR-C4_ infusions received
**1**	**NA**	**NED**	**1**	**Yes**	**No**	**NED**	**87**	**EBV**	**3**
**2**	**Yes**	**NED**	**2**	**Yes**	**No**	**MRD**	**60**	**CMV**	**4**
**4**	**Yes**	**NED**	**2**	**Yes**	**No**	**NED**	**89**	**EBV**	**2**
**5**	**Yes**	**MRD**	**1**	**No**	**No**	**Overt**		**EBV**	**1**
**6**	**NA**	**NED**	**2**	**Yes**	**No**	**NED**	**59**	**CMV**	**4**
**7**	**No**	**MRD**	**1**	**Yes**	**No**	**MRD**	**33**	**EBV**	**1**
**8**	**Yes**	**MRD**	**1**	**No**	**No**	**MRD**		**EBV**	**2**
**9**	**Yes**	**MRD**	**1**	**Yes**	**No**	**MRD**	**52**	**CMV**	**4**
**14**	**Yes**	**NED**	**2**	**Yes**	**No**	**MRD**	**61**	**EBV**	**2**
**15**	**No**	**NED**	**2**	**Yes**	**No**	**NED**	**110**	**CMV**	**2**
**19**	**Yes**	**NED**	**1**	**Yes**	**No**	**Overt**	**243**	**EBV**	**1**
**23**	**Yes**	**NED**	**1**	**Yes**	**No**	**NED**	**246**	**EBV**	**1**
**26**	**Yes**	**NED**	**1**	**Yes**	**Yes**	**NED**	**53**	**EBV**	**2**
**27**	**Yes**	**NED**	**1**	**Yes**	**Yes**	**NED**	**178**	**EBV**	**1**
**28**	**Yes**	**NED**	**2**	**Yes**	**Yes**	**NED**	**31**	**CMV**	**2**

*AML* Acute Myeloid Leukemia, *HCT* Hematopoietic Cell Transplantation, *NED* No Evidence of Disease, *MRD* Measurable Residual Disease, *EBV* Epstein-Barr Virus, *CMV* Cytomegalovirus.

This table summarizes the clinical data of patients with AML relapsed/refractory after HCT, who received T_TCR-C4_ infusions.

As in Arm 1^13^, T_TCR-C4_ infusions were safe and well tolerated (Supplementary Table 2). The incidence of acute and chronic GVHD (aGVHD, cGVHD) was lower compared to DLIs or second HCTs as salvage strategies^14,29^, with only one patient ( ~ 7%) developing grade 3 aGVHD and one ( ~ 7%) developing moderate cGVHD post-T_TCR-C4_ infusion. Similarly to our prophylactic arm^13^, a biopsy obtained at the time of cGVHD showed lower T_TCR-C4_ frequencies in tissue ( ~ 0.08% of total CD3^+^ T cells) (Supplementary Fig. 4) compared to PB (60/mcL, ~8% of total CD8^+^ T cells), suggesting no correlation between PB T_TCR-C4_ concentration and tissue GVHD.

Among the patients enrolled, four showed no overt relapse and/or MRD following T_TCR-C4_ infusion(s) (Supplementary Fig. 5) supporting T_TCR-C4_’s potential biologic activity. However, when all 15 patients were analyzed together, the median OS was 242 days, the 2-year OS was 33%, and the 3-year OS was 20% (Supplementary Fig. 6), indicating that these individual outcomes did not translate into a significant survival advantage over historical salvage treatments^1,2,14,15^. For example, DLI post-HCT relapse showed a 2-year OS of 21% ( ± 3%) from relapse and 56% ( ± 10%) from DLI in cases of remission or with a favorable karyotype, dropping to 15% ( ± 3%) in aplasia or active disease^29^.

### Virus-specific substrate T cells and AML presence at the time of infusion are major determinants of T_TCR-C4_ persistence

**Fig. 1:**
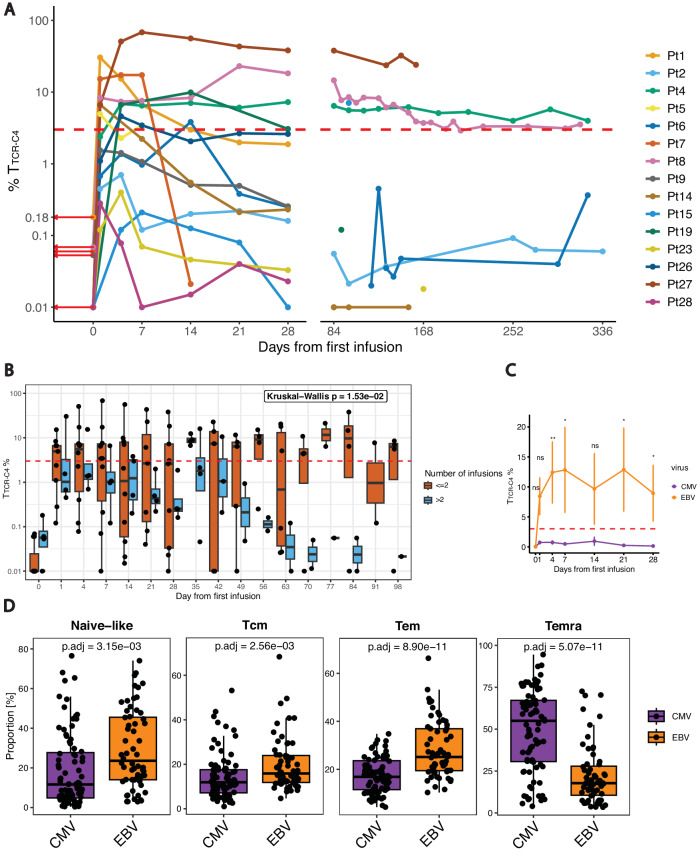
Virus-dependent terminal differentiation skewing of T_TCR-C4_. **A** Line plot showing the percentage (log scale, *y*-axis) of T_TCR-C4_ in PBMCs after the first T_TCR-C4_ infusion for all patients (*n* = 15). Data points represent individual samples at different timepoints post-infusion. Each patient is represented by a distinct color. Red arrows indicate WT1-specific CD8^+^ T cells percentages at day 0. **B** Boxplots comparing T_TCR-C4_ percentages (log scale, *y*-axis) post-first infusion (*x*-axis) in all patients (*n* = 15), colored by the number of infusions (light blue: ≤2; dark orange: > 2). The dashed red line indicates the 3% threshold used to define persisting T_TCR-C4_ cells. Statistical significance was determined using Kruskal-Wallis test, with *p* < 0.05 considered significant. Boxplots show the interquartile range (IQR); lines denote medians. The bounds of the box represent the 25th and 75th percentiles; whiskers span 1.5x IQR. **C** Line plot of T_TCR-C4_ percentages (*y*-axis) derived from EBV- (dark orange) or CMV-specific (dark violet) substrate cells over a 28-day period (*x*-axis) post-first infusion (*n* = 15). Error bars indicate mean ± standard error of the mean. Statistical significance was determined using a two-sided Wilcoxon Rank Sum test. (* *p* < 0.05, ** *p* < 0.01). Significant differences were observed at days 4 (*p* = 0.00699), 7 (*p* = 0.012), 21 (*p* = 0.0303), and 28 (*p* = 0.0295). **D** Boxplots showing differential abundance analysis of CMV-specific (dark violet) and EBV-specific (dark orange) CD8^+^ T-cell subsets derived from the analysis of mass cytometry data (*n* = 143). CD8^+^ T-cell subsets are defined based on marker co-expression showed in Supplementary Fig. 8B. Differential abundance between conditions (CMV vs. EBV) was computed using the edgeR method within the diffcyt framework. *P*-values were adjusted for multiple testing using the Benjamini-Hochberg procedure. Adjusted *p*-values (threshold for significance of *p* < 0.05) are displayed above each comparison. Boxplots show the IQR; lines denote medians. The bounds of the box represent the 25th and 75th percentiles; whiskers span 1.5x IQR.

In our cohort, baseline PB WT1-specific tetramer^+^ T cells were low (Fig. 1A**–**red arrows, day 0) indicating limited endogenous WT1-specific T cells. By day 28 post-infusion, four patients showed T_TCR-C4_ levels exceeding 3% of the CD8^+^ T cells (persistence threshold), with three maintaining these levels beyond day 100. Patients with T_TCR-C4_ frequencies below 3% were eligible for additional infusions (two infusions in 6/15 patients, >2 infusions in 4/15 patients) (Table 1). However, more than two infusions did not significantly increase persistence (Fig. 1B).

We next explored the contribution of substrate cell virus-specificity to post-infusion persistence. Within 28 days, EBV-specific T_TCR-C4_ were more abundant than CMV-specific T_TCR-C4_, which remained below the persistence threshold (Fig. 1C). These differences endured when EBV-specific T_TCR-C4_ recipients from Arm 1 were included (Supplementary Fig. 7)^13^. As central-memory CD8^+^ T cells (Tcm) have shown improved persistence compared to more differentiated phenotypes post-transfer^30^, we sought to explore whether differences in endogenous virus-specific cells could recapitulate these findings. We analyzed the phenotypes of EBV- and CMV-specific T cells using mass cytometry data^31^ from 143 healthy individuals and cancer patients. Multidimensional scaling (MDS) showed segregation of CMV- and EBV-specific CD8^+^ T cells along the first dimension (MDS dim.1) suggesting distinct T-cell differentiation states between these groups (Supplementary Fig. 8A). Next, Flow Self-Organizing Maps (FlowSOM)^32^ metaclustering identified four CD8^+^ T-cell subsets: naïve (CD95^-^,CD45RA^+^, CCR7^+^, CD27^+^, CD28^+^), effector memory (Tem) (CD95^+^, CD45RA^-^, CD45RO^+^, CD27^+^, CD28^+^, CD127^+^), Tcm (CD95^+^, CD45RA^-^, CD45RO^+^, CCR7^+^, CD27^+^, CD28^+^, CD127^+^), CD45RA^+^ effector memory (Temra) (CD95^+^, CD45RA^+^, CD45RO^−^, CD57^+^, KLRG1^+^) (Supplementary Fig. 8B)^33,34^. Differential abundance analysis revealed a significant (false discovery rate/FDR < 0.05) increase of Temra cells in CMV-specific T cells and of naïve, Tcm and Tem in EBV-specific T cells (Fig. 1D, and Supplementary Fig. 8C). These results suggest that the Temra state of CMV-specific substrate cells compromises post-infusion persistence, whereas EBV-specific cells, derived from less differentiated precursors, facilitate persistence.

Persistence varied among EBV-specific T_TCR-C4_ recipients (Fig. 1C), with only three of ten exhibiting long-term persistence ( > 100 days post-infusion) (Fig. 1A, and Supplementary Fig. 9). We reasoned that persistence was influenced by AML high-risk factors, including post-transplant remission duration and disease burden at the time of T_TCR-C4_ infusion^3^. Patient 1 was excluded from this analysis due to unavailable WT1-expression data. Six of the remaining patients did not show long-term persistence: four (5,7,14, and 19) had detectable disease (MRD or overt) within two weeks of T_TCR-C4_ infusion, and two (23, 26) who were disease-free at infusion, had relapsed within 3 months post-HCT, suggesting difficult-to-control disease. Statistical testing revealed a trend (Fisher’s exact test, *p* = 0.08) linking reduced persistence with these high-risk clinical factors (detectable disease pre-infusion or early relapse post-HCT). Patient 8, initially MRD-positive before the first infusion, became MRD-negative through salvage therapy before the second infusion, achieving the longest observed T_TCR-C4_ persistence. Reclassifying this patient with the MRD-/non-early relapse group, renders the association between disease risk factors and persistence statistically significant (*p* < 0.05) (Supplementary Fig. 10). Notably, cell blood counts and blasts percentage did not significantly correlate with persistence (Supplementary Table 3). These findings suggest that aggressive disease post-HCT, independently of virus-specificity, was associated with reduced T_TCR-C4_ persistence in vivo.

### Long-term persistent T_TCR-C4_ acquire NKL/terminal differentiation markers associated with progressive loss of function in vivo

**Fig. 2:**
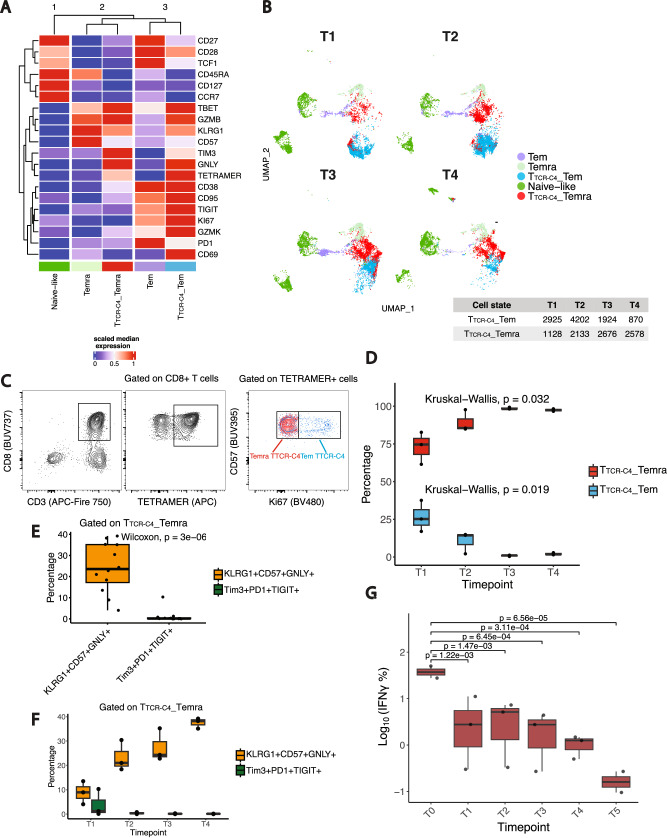
CD8^+^ T cell subset phenotypes and functional states over time. **A** Heatmap of fluorescence intensity for 20 markers across Naïve-like, Tem, T_TCR-C4__Tem (tetramer^+^), Temra, T_TCR-C4__Temra (tetramer^+^) PB CD8^+^ T cells. Median expression values are highlighted (red = high, blue = low). Data were scaled post-aggregation to highlight population-level differences between tetramer^+^ and tetramer^-^ samples. K-means algorithm categorized clusters by similarity into three groups (1-3, top of heatmap). Data were derived from spectral flow-cytometry. **B** UMAP plots of the CD8^+^ T-cell subsets, colored by subset and split by timepoint. The table shows the absolute numbers of T_TCR-C4__Tem and T_TCR-C4__Temra cells at timepoints T1-T4. **C** Contour plots illustrating the gating strategy for T_TCR-C4__Tem (blue) and T_TCR-C4__Temra (red): CD3^+^CD8^+^ were first gated, followed by selection of tetramer^+^ cells, and finally separation based on Ki67 expression: Ki67^+^ (T_TCR-C4__Tem, blue) and Ki67^-^ (T_TCR-C4__Temra, red) cells. This strategy was based on marker expression in Fig. 2A. **D** Boxplots of T_TCR-C4__Tem and T_TCR-C4__Temra percentages over time (T1-T4, *n* = 3). Statistical significance was assessed using Kruskal-Wallis test. Boxplots show the IQR; lines denote medians. The bounds of the box represent the 25th and 75th percentiles; whiskers span 1.5x IQR. **E** Boxplots showing cytotoxic (KLRG1^+^, CD57^+^, GNLY^+^; dark red) vs. exhausted (TIM3^+^, PD1^+^, TIGIT^+^; blue) T cells among T_TCR-C4__Temra (*n* = 3). Statistical significance was assessed using a two-sided Wilcoxon rank-sum test. Boxplots show the IQR; lines denote medians. The bounds of the box represent the 25th and 75th percentiles; whiskers span 1.5x IQR. **F** Boxplots displaying cytotoxic (dark red) vs. exhausted (blue) T cells among T_TCR-C4__Temra over time (T1-T4, *n* = 3). Boxplots show the IQR; lines denote medians. The bounds of the box represent the 25th and 75th percentiles; whiskers span 1.5x IQR. **G** Boxplots showing log10-transformed percentages of T_TCR-C4_ IFNγ^+^ cells within the CD8^+^ population following WT1-peptide stimulation across timepoints post-first infusion (*n* = 3, Supplementary Table 6). A linear mixed-effects model assessed the effect of Timepoint (T0 as intercept) on IFNγ production, with a two-sided hypothesis test. Boxplots show the IQR; lines denote medians. The bounds of the box represent the 25th and 75th percentiles; whiskers span 1.5x IQR.

To investigate the fate of T_TCR-C4_ post-transfer in patients with persistent T_TCR-C4_, and compare these with endogenous (TCR_C4_^−^) T cells, we used a validated^24,25^ 24-color spectral flow-cytometry panel (Supplementary Table 4). PB was analyzed at ~1 (T1), ~7 (T2), 28 (T3) days, and ~4 months (T4) post-transfer (Supplementary Table 5). FlowSOM clustering revealed five CD8^+^ T-cell states categorized into three higher-level clusters (Fig. 2A, top dendrogram): naïve-/cm-like cells (Fig. 2A, dark green) (CD45RA^+^, CCR7^+^, CD27^+^, CD28^+^); Tem subgroup, including endogenous CD8^+^ Tem (CD28^+^, CD27^+^, Ki67^+^, CD38^+^, TIGIT^+^, PD1^+^, Tbet^+^) (Fig. 2A, violet), and phenotypically similar T_TCR-C4__Tem (tetramer^+^) (Fig. 2A, blue); Temra subgroup of endogenous CD8^+^ T cells (CD45RA^+^, CD57^+^, KLRG1^+^, GZMB^+^) (Fig. 2A, light green) and closely clustered T_TCR-C4__Temra (Fig. 2A, red). Two-dimensional Uniform Manifold Approximation and Projection (UMAP) revealed a progressive increase in T_TCR-C4__Temra and decline in T_TCR-C4__Tem from T1 to T4 (Fig. 2B). T_TCR-C4__Temra expressed minimal Ki67 compared to T_TCR-C4__Tem (Fig. 2A), enabling manual gating of these two subsets (Fig. 2C, and Supplementary Fig. 11). Over time, T_TCR-C4__Temra significantly increased, while T_TCR-C4__Tem decreased (*p* < 0.05) (Fig. 2D**)**, suggesting differentiation from proliferative T_TCR-C4__Tem to non-proliferative T_TCR-C4__Temra. T_TCR-C4__Temra predominantly expressed cytotoxic/KLR markers (KLRG1, CD57, GNLY), previously linked to T-cell dysfunction in AML^24,25^, rather than classical T-cell exhaustion markers (Tim3, PD1, and TIGIT) (Fig. 2E**)**^20,35^. Stratification by timepoint revealed progressive increase of the cytotoxic subset versus the exhausted subset **(**Fig. 2F**)**. This phenotypic shift coincided with a significant decline over time in T_TCR-C4_ CD8^+^ T cells producing IFNγ (Fig. 2G, and Supplementary Fig. 12, 13, Supplementary Table 6). Production of INFγ by Tet^-^ cells (negative control) was near undetectable (Supplementary Figs. 12, 13).

Previous work^36^ showed that NKL skewing of CAR-T cell products correlates with increased innate-like serum cytokines. Although none of the 49 cytokines we tested showed a significant increase over time, the pro-inflammatory cytokine IL-18 exhibited an increasing trend (Supplementary Fig. 14, and Supplementary Table 7), consistent with previous findings^36^. Notably, the reported changes in IL-15 concentration were below the threshold limit of detection of our assay (3.84 pg/ml). It is possible that changes in IL-15 concentration still occur in this context, but they were not detectable by our assay.

Overall our findings show that long-term persisting EBV-specific T_TCR-C4_ skew towards a distinct NKL phenotype linked to functional decline.

### Single-cell profiling of CD8^+^ T cells reveals distinct immune states of endogenous and T_TCR-C4_ cells

To explore the relationship between CD8^+^ endogenous T-cell and T_TCR-C4_ states, we performed single-cell RNA sequencing (scRNAseq) on available PB and bone marrow (BM) samples from patients with T_TCR-C4_ ≥ 3% of CD8^+^ T cells at day 28 or later after first infusion. This analysis included two prophylactic^13^ and five treatment-arm patients **(**Supplementary Table 8**)**.

**Fig. 3:**
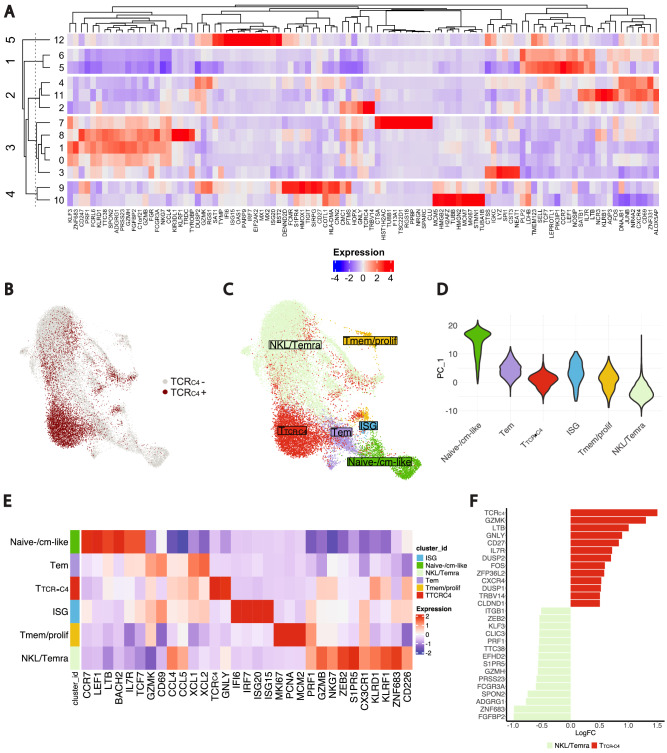
Single cell transcriptomic analysis of endogenous and T_TCR-C4_ CD8^+^ T-cell states and their differentiation dynamics. **A** Heatmap showing the top 10 differentially expressed genes per cluster. The “top 10” refers to the 10 genes with the most significant differential expression across the identified clusters. The dendrogram on the left displays the similarity between the 13 clusters, which were determined through unsupervised clustering based on gene co-expression patterns. The top dendrogram shows the relationships between the genes based on their expression patterns. K-means algorithm grouped the 13 clusters into 5 main categories, labeled 1 to 5 on the left side of the heatmap. Blue indicates low expression; red, high. **B** UMAP plot of the CD8^+^ T cells (endogenous and T_TCR-C4_^+^) on a two-dimensional space. T_TCR-C4_^+^ cells were identified using scGate, an R package which scores cells based on TCR_C4_ expression and defines thresholds to classify cells as positive or negative for the population of interest. TCR_C4_^+^ cells are colored in dark red, while TCR_C4_^-^ cells (endogenous) are represented in light gray. **C** UMAP plot displaying the two-dimensional distribution of annotated CD8^+^ T-cell transcriptional states, colored by subset. **D** Violin plot illustrating the distribution of CD8^+^ T-cell subsets identified through gene expression (Fig. 3A) and TCR_C4_ score (Fig. 3B) along the principal component 1 (PC_1) axis. Each violin represents a different CD8^+^ T-cell state. The proximity of each subset along PC_1 (*y*-axis) indicates transcriptional similarity, with subsets closer together showing more similar gene expression profiles. **E** Heatmap showing the differential expression of manually curated genes across the five annotated CD8^+^ T-cell subsets. Blue and red indicate the relative expression levels of each marker within each subset, with blue representing lower expression and red indicating higher expression. **F** Bar plot showing differential gene expression analysis comparing T_TCR-C4_ and NKL/Temra (T_TCR-C4_^-^) CD8^+^ T cells. Differential expression was performed using a two-sided Wilcoxon rank-sum test implemented in the Seurat package. *P*-values were adjusted for multiple testing using the Bonferroni correction. Genes with an adjusted *p*-value (padj) <0.01 and log2 fold change (logFC) > 0.5 or < −0.5 were included. Bars represent log2 fold change values, with red indicating genes upregulated in T_TCR-C4_ and green representing genes upregulated in NKL/Temra.

Unsupervised clustering of PB CD8^+^ T cells (n = 24,472) identified 13 clusters, with the TCR_C4_ transgene expressed across several clusters, but primarily in cluster 2 (Fig. 3A). Using the marker-based purification algorithm scGate^37^, we identified TCR_C4_^+^ cells (Fig. 3B) labeled as T_TCR-C4_, while endogenous (TCR_C4_^–^) T cells included clusters 5 and 6, labeled as naïve-/cm-like, expressing *CCR7*, *SELL*, *TCF7*, *LEF1*, *IL7R*; clusters 2, 4 and 11, labeled as Tem, expressing genes associated with activation (*CD69*, *TIGIT*, *GZMK*); cluster 12, labeled as interferon signaling genes (ISG), expressing *ISG15*, *ISG20*, *IRF7*, *IFI6*; clusters 9 and 10, labeled as T memory/proliferative (Tmem/prolif), expressing proliferation (*MKI67*, *MCM5*, *MCM7*) and memory (*CD27*) genes; clusters 0,1,3,7, and 8, labeled as NKL/Temra, expressing genes associated with cytotoxicity/NKL (*KLRF1*, *KIR3DL1*, *NKG7*, *FCGR3A*, *GZMB*, *KLRD1*, *PRF1*) (Fig. 3A–E, and Supplementary Fig. 15A)^33,34,38–40^.

To assess transcriptional similarities among cell states, we performed principal component analysis, positioning naïve-/cm-like and NKL/Temra as differentiation spectrum extremes, with T_TCR-C4_ as the intermediate state between these two extremes (Fig. 3D). Next, to ensure unbiased labeling of CD8^+^ T cells, we constructed a scRNAseq reference atlas comprising 109,051 CD8^+^ T cells, using a published scRNAseq dataset of tumor-infiltrating lymphocytes^41^. We aligned and projected our scRNAseq dataset onto the reference atlas (Supplementary Fig. 15B), confirming the correspondence between the transcriptional states in our dataset and the reference. Differential gene expression (DGE) analysis using manually curated markers further supported this annotation (Supplementary Table 9, Fig. 3E)^33,34,38,39^.

Consistent with previous work^34^, we observed a progressive reduction of stem-like markers (*IL7R*, *TCF7*) from naïve-/cm-like cells through Tem and NKL/Temra cells, while activation markers (*CD69*, *GZMK*) peaked in Tem. NKL markers, including KLR-exhaustion markers (*S1PR5*, *ZEB2*)^42,43^, were highest in NKL/Temra. (Supplementary Fig. 15C). These results were confirmed through pairwise DGE between T_TCR-C4_ and endogenous NKL/Temra. T_TCR-C4_ expressed genes associated with activation (*GZMK*, *LTB*, *GNLY*) and memory (*IL7R*, *CD27*), while NKL/Temra cells expressed cytotoxicity (*GZMH, PRF1*) and NKL/Temra genes (*KLF3*, *FCGR3A, ZEB2*, *S1PR5*) (Fig. 3F)^26,36,42–44^. These findings align with previous CAR-T cell studies^36^, and suggest that T_TCR-C4_ expressing activation and NKL markers represent an intermediate state between Tem and NKL/Temra.

### Clonal differentiation and trajectory analysis reveal T_TCR-C4_ as an intermediate state in CD8^+^ T cell differentiation

**Fig. 4:**
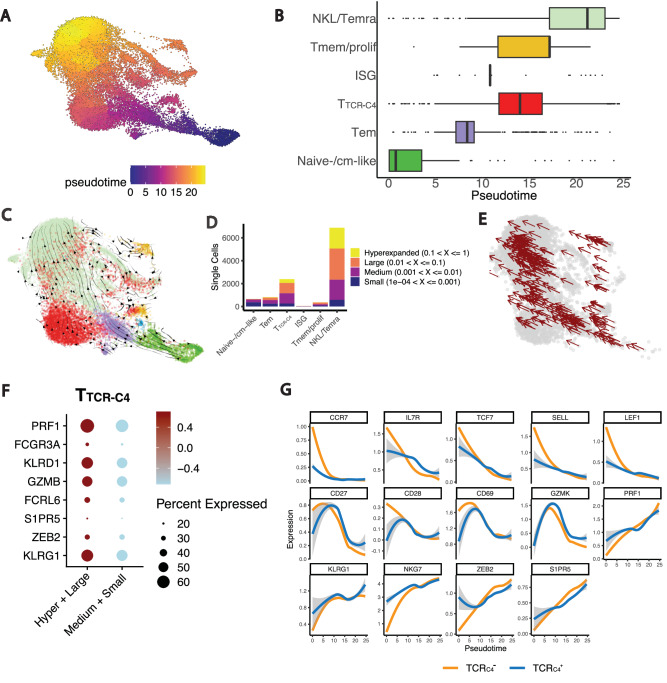
CD8^+^ T-cell differentiation dynamics and clonal expansion. **A** UMAP plot illustrating the developmental trajectory of CD8^+^ T-cell transcriptional states, as predicted by Monocle. The color scale represents the pseudotime, where blue indicates earlier developmental stages and orange/yellow indicates later stages. **B** Boxplots showing the distribution of the 5 CD8^+^ T-cell subsets (*y*-axis) along the pseudotime (*x*-axis). The position of the boxes provides context for how the subsets are positioned within the developmental trajectory, with subsets at lower pseudotime values representing earlier stages of differentiation, while those at higher pseudotime values correspond to later stages. Boxplots show the IQR; lines denote medians. The bounds of the box represent the 25th and 75th percentiles; whiskers span 1.5x IQR. **C** UMAP plot colored by the annotated CD8^+^ subsets overlaid with the predicted velocity stream computed through scVelo. The velocity streams represent the predicted direction and magnitude of gene expression changes for each individual cell, providing insights into the dynamic transitions between cellular states. These streams highlight the likely trajectories cells follow as they evolve over time, offering a predictive view of future cellular states based on current transcriptional dynamics. **D** Stacked bar plot showing cell count of different T-cell states (*x*-axis) in specific clonal frequency ranges. Colors indicate the clonal frequencies. **E** UMAP visualization of hyperexpanded and large CD8^+^ T cell clones, defined in (**D**), showing shared TCR sequences across different clusters. Each point represents a single cell, while arrows indicate the inferred directionality of clonal expansion across clusters. **F** Dot plot showing the expression of NKL genes in T_TCR-C4_ with a higher degree of clonal expansion (Hyperexpanded, large) vs. less clonally expanded T_TCR-C4_ (Medium, small). Clonal frequency ranges are defined in (**D**). **G** Line plots displaying the smoothed gene expression of selected genes along the pseudotime for TCR_C4_^-^ (orange) and TCR_C4_^+^ (blue) cells. These plots illustrate the dynamic changes in gene expression (*y*-axis) as cells progress along the inferred trajectory (*x*-axis). The smoothed curves show how the expression of each gene varies at different pseudotime points.

To analyze T-cell differentiation dynamics we used Monocle trajectory^45^ and RNA velocity^46^ inference. This analysis confirmed T_TCR-C4_ as an intermediate state between Tem and the terminal NKL/Temra state (Fig. 4A–C), and removing the TCR_C4_ transgene did not alter this differentiation trajectory (Supplementary Fig. 16A, B). Next, to explore clonal differentiation dynamics, we analyzed the TCR sequencing from our dataset (Supplementary Table 8). Similarly to previous findings^25,36^, the most clonally expanded subsets were T_TCR-C4_ and endogenous NKL/Temra cells (Fig. 4D). We then employed TCR amino acid sequences to trace differentiation pathways, hypothesizing that T cells differentiate progressively as they clonally expand. Using shared TCR sequences across clusters, we inferred trajectories by tracking transitions from less expanded to more expanded populations (Fig. 4E). Clonally expanded T_TCR-C4_ upregulated NKL/cytotoxicity genes, suggesting skewing towards the NKL phenotype concomitant with clonal expansion (Fig. 4F). This analysis highlighted a distinct clonal differentiation pattern of endogenous and T_TCR-C4_ progressing towards NKL/Temra.

Single-gene expression over pseudotime revealed early peaks of naïve/stem-like markers (*CCR7*, *IL7R*, *TCF7*, *SELL*, *LEF1*) (Supplementary Fig. 17, first row), naïve/memory (*CD27*, *CD28*) and activation markers (*GZMK*, *CD69*), with the latter remaining high throughout mid-pseudotime before declining (Supplementary Fig. 17, second row). TCR_C4_ expression peaked at mid-pseudotime, but remained expressed until the end of pseudotime. NKL/Temra-associated genes (*PRF1*, *KLRG1*, *NKG7*, *ZEB2*, *S1PR5*) peaked at the end of pseudotime (Supplementary Fig. 17, third row). Smoothed gene expression analysis of endogenous (TCR_C4_⁻) and TCR_C4_⁺ cells revealed an increase in NKL/cytotoxicity markers along pseudotime. At the start of the pseudotime trajectory, endogenous TCR_C4_⁻ cells predominantly expressed naïve-/cm-like genes (*CCR7, SELL, LEF1*), consistent with the presence of naïve endogenous CD8⁺ T cells. In contrast, ex vivo expanded TCR_C4_⁺ cells lacked naïve markers. As the trajectory progressed, the expression of naïve-/cm-like genes became comparable between the two groups (Fig. 4G).

Overall, our findings suggest that T_TCR-C4_ largely existed in an intermediate state between the Tem phenotype and the NKL/Temra stage.

### AML drives T_TCR-C4_ towards NKL/terminal differentiation instead of the dominant exhaustion pattern associated with T cells in solid tumors

**Fig. 5:**
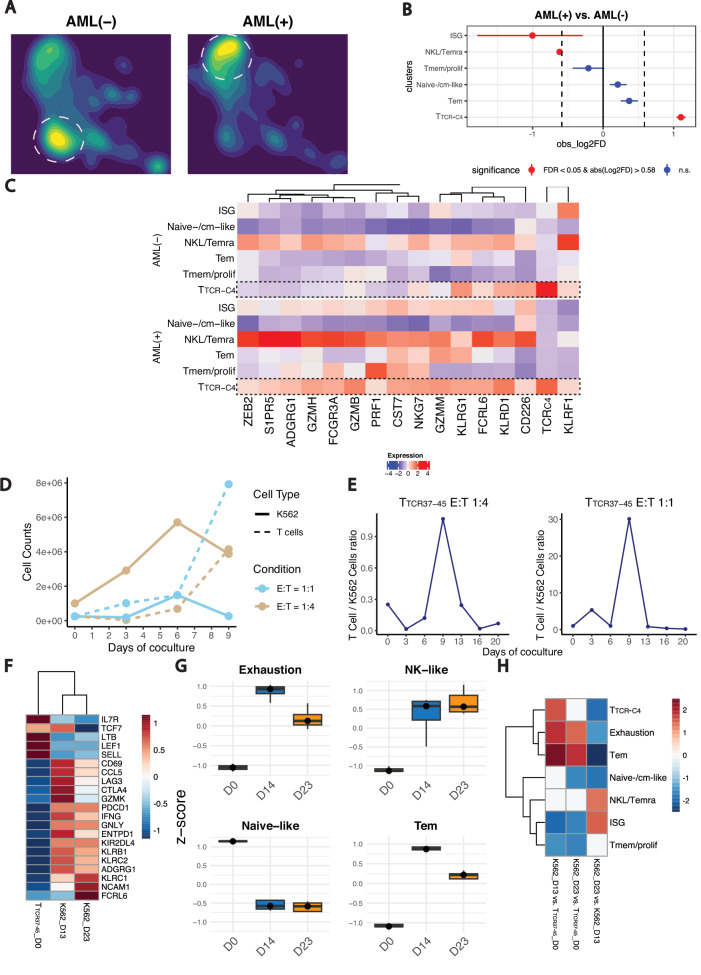
AML induces T_TCR-C4_ NKL/Temra differentiation skewing. **A** UMAP plot of CD8^+^ T cells colored by density and split by group (AML(-) and AML( + )). AML(+) samples are those with detectable leukemic cells in BM or PB, while AML(-) lack detectable disease. Color intensity reflects CD8^+^ T-cell density (blue = low, yellow = high). Dashed lines highlight areas of highest density in each group. **B** Point-range plot showing the pairwise (AML(+) vs. AML(-)) proportional difference for each CD8^+^ T-cell subset. Horizontal lines extending from each point denote the 95% confidence intervals of the log2FD. Colors indicate the statistical significance (red: FDR < 0.05, blue: FDR ≥ 0.05); vertical dashed lines mark the absolute value of log2FD cutoff for significance. **C** Heatmap displaying DGE of NKL genes across the CD8^+^ T-cell subsets in AML(-) vs. AML(+) (blue = low, red = high expression). Dashed boxes highlight NKL/Temra genes. **D** Line plots showing the absolute cell counts over time (days of coculture) for T_TCR37-45_ (dashed lines) and K562 cells (solid lines) at E:T ratios of 1:1 (sky blue) and 1:4 (tan). **E** Line plots showing the T-cell-to-tumor-cell ratio over time for each E:T condition (left: 1:4; right: 1:1). Cell ratios were calculated based on flow cytometry data. **F** Heatmap depicting significant DEGs across T_TCR37-45__D0 (T_TCR37-45_-only), K562_D13 (T_TCR37-45_ T cells after 13 days of coculture with K562 AML cell line), K562_D23 (T_TCR37-45_ T cells after 23 days of coculture with K562 AML cell line) conditions. Signifcance was determined using DESeq2 (FDR < 0.05). Blue = low, red = high expression. **G** Boxplots illustrating the z-score of Exhaustion, NK-like, Naïve-like and Tem signatures aross co-culture timepoints. Each box represents the distribution of z-scores for the genes in the respective signature at each timepoint. The center line indicates the median; the box limits represents the IQR, and the whiskers span 1.5 x IQR. **H** Heatmap illustrating the enrichment of the top 50 DEGs from scRNAseq-derived subsets (T_TCR-C4_, Tem, Naïve-/cm-like, NKL/Temra, ISG, Tmem/prolif) and manually curated exhaustion markers across three comparisons: K562_D13 vs. T_TCR37-45__D0, K562_D23 vs. T_TCR37-45__D0, K562_D23 vs. K562_D13. Red = high, blue = low enrichment. T_TCR37-45_ represents the baseline (T_TCR37-45_-only condition).

To investigate AML’s impact on the differentiation of endogenous CD8^+^ T cells and T_TCR-C4_, patients samples were grouped as AML- (no AML detected, including two prophylactic arm cases)^13^, or AML+ (blasts evident in BM and/or PB) (Supplementary Table 10, and Supplementary Fig. 18A, B). The increase of ISG and NKL/Temra in AML+ and T_TCR-C4_ in AML- (Fig. 5A) was significant (Fig. 5B), with a wide confidence interval for ISG, making the magnitude of this difference unclear. T_TCR-C4_ overexpressed NKL/Temra genes in AML+ compared to AML- (Fig. 5C), suggesting that AML blasts may induce a transcriptional shift towards NKL/Temra in T_TCR-C4_ and endogenous T cells.

Next, we evaluated previously described T-cell dysfunction signatures^25,47,48^ and found that progenitor exhausted (Tpex) signatures were enriched in naïve-/cm-like and Tem, while Terminally exhausted (Ttex) signatures were enriched in Tmem/prolif (Supplementary Fig. 19A). However, intersecting these signatures with the top 50 differentially expressed genes per subset, revealed limited overlap (*IL7R, LEF1, SELL, CCR7, TCF7, FOXP1, FOS*) for Tpex and naïve-/cm-like, three genes (*CXCR4, NR4A2, JUN*) for Tpex and Tem, and only *TYMS* for Ttex and Tmem/prolif. The absence of canonical markers of exhaustion suggests that these subsets do not predominantly express this program. In contrast, NKL/Temra showed significant overlap with previously published signatures of NKL skewing (Supplementary Fig. 19B, and Supplementary Table 11)^25^. Additionally, T_TCR-C4_ in the setting of AML+ samples showed hyperexpression of NKL instead of exhaustion genes (Supplementary Fig. 19C). These findings align with a previous report in large B-cell lymphoma showing that terminally differentiated CD57^+^ CAR-T cells with senescence features can transition to an NK-like rather than exhaustion program^40^. We also projected scores from published NKL signatures^25,42,43^ onto our UMAP, revealing overlaps that link the NKL/Temra CD8^+^ T cells we identified with an end-term differentiation subset (Supplementary Fig. 19D).

To clarify whether AML-induced dysfunction aligns with T-cell exhaustion or NKL skewing, we used a previously described model^26^. We exposed in vitro CD8^+^ transgenic WT1-specific T cells targeting the HLA A*0201-restricted WT1_37-45_ epitope (T_TCR37-45_) to high-WT1-expressing HLA-A*0201-transduced K562 acutely transformed chronic myelogenous leukemia (Supplementary Fig. 20A**)**. As K562 primarily express standard proteasomes and are not readily lysed by T_TCR-C4_ targeting the immunoproteasome-specific WT1_126–134_ peptide, we used T_TCR37-45_ due to its proteasome-agnostic WT1-derived peptide recognition^49^. Exposing T_TCR37-45_ to fresh K562 every 3-4 days at stable effector-to-target (E:T) ratios (1:1, 1:4) caused initial tumor lysis (Fig. 5D), but control was lost by day 13, marked by a declining T_TCR37-45_-to-tumor ratio (Fig. 5E). Irrelevant TCR-expressing T cells exerted no control (Supplementary Fig. 20B). Bulk-RNA sequencing on sorted T cells (Supplementary Fig. 21) found increased (FDR < 0.05) naïve/stem-like genes (*IL7R, TCF7, LTB*, *LEF1*, *SELL*) at day 0, followed at day 13 by increased effector (*CCL5, GZMK*), activation/exhaustion (*CD69*, *LAG3*, *CTLA4*, *PDCD1*), and NKL/Temra markers (*KLRB1*, *ENTPD1*, *KLRC1*, *KLRC2, NCAM1*) (Fig. 5F-H). By day 23, the activation/exhaustion markers decreased, while NKL/Temra persisted or increased (Fig. 5F–H). To integrate these findings with the scRNAseq dataset, we performed a gene-set enrichment analysis with the top 50 differentially expressed genes from each CD8^+^ subset identified in scRNAseq, including naïve-/cm-like, Tem, NKL/Temra, T_TCR-C4_, Tmem/prolif and ISG from the scRNAseq dataset. We also included a manually curated exhaustion signature (Supplementary Table 12). Exhaustion and Tem signatures exhibited enrichment in later timepoints, yet there was no further increase at day 23 versus day 13 (Fig. 5H). In contrast, the NKL/Temra increased between days 13 and 23 corresponding to T_TCR37-45_ loss of tumor control (Fig. 5E).

We performed GSEA on the scRNA-seq data to assess whether T_TCR-C4_ cells lose antigen reactivity and tumor control as they differentiate into NKL T_TCR-C4_ cells. Our analysis showed no significant differences between NKL and non-NKL T_TCR-C4_ states (Supplementary Fig. 22A, B), and TCR signaling genes were not downregulated in NKL T_TCR-C4_ cells compared to non-NKL T_TCR-C4_ cells (Supplementary Fig. 22C, D).

To confirm that AML-exposed T cells skewed towards a NKL/Temra rather than exhausted (Tex) phenotype, we projected onto a scRNAseq reference atlas^41^ a BM AML CD8^+^ T-cell dataset, compiled from published sources^23,25,50–52^, alongside datasets representing pancreatic^53^, melanoma^54^, and lung^55^ solid tumors (Supplementary Fig. 23A, B). Unlike solid tumors, which included a Tex cluster, AML datasets aligned with our spectral flow-cytometry and scRNAseq findings confirming the absence of Tex and the presence of NKL/Temra cells (Supplementary Fig. 23C). Although these studies did not specifically analyze tumor-antigen reactive T cells, the findings collectively suggest that the NKL (versus exhaustion) signature is intricately associated with AML-induced T-cell dysfunction.

### Prolonged azacitidine exposure enhances self-renewal and Tcm features of T_TCR-C4_, supporting T_TCR-C4_-mediated AML control

**Fig. 6:**
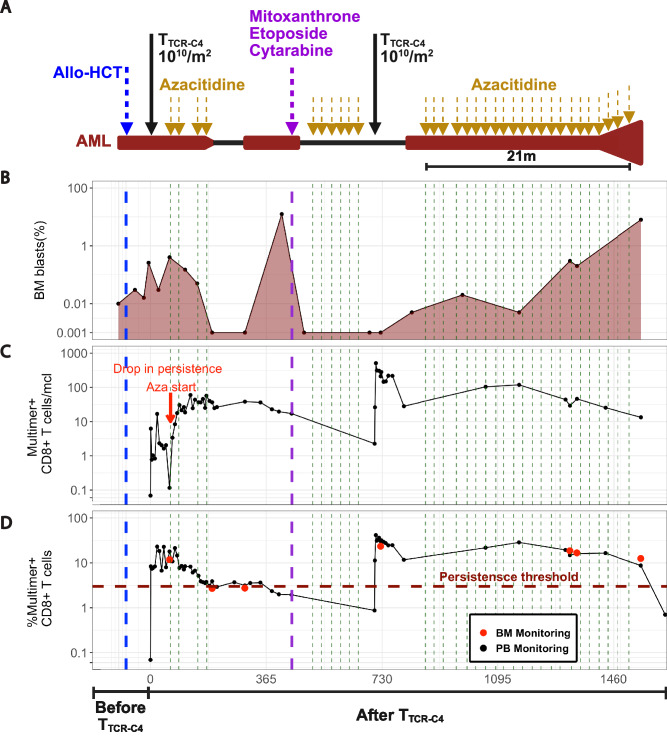
Timeline and disease response in a patient with relapsed AML post-allogeneic HCT treated with T_TCR-C4_ and Azacitidine. **A** Timeline of patient’s treatment regimen. Dark red highlights timeframes with detectable BM AML **B** Percent of BM AML blasts (*y-*axis, log scale) by multiparametric flow cytometry (MFC) at specific timepoints (black dots, dark red shaded area). **C** Multimer^+^ cells/µl and **D** Percent multimer^+^ of CD8^+^ T cells in PB (black dots) and BM (red dots) collected before and after T_TCR-C4_ infusions. The red arrow indicates the lack of T_TCR-C4_ persistence before the start of Azacitidine.

**Fig. 7:**
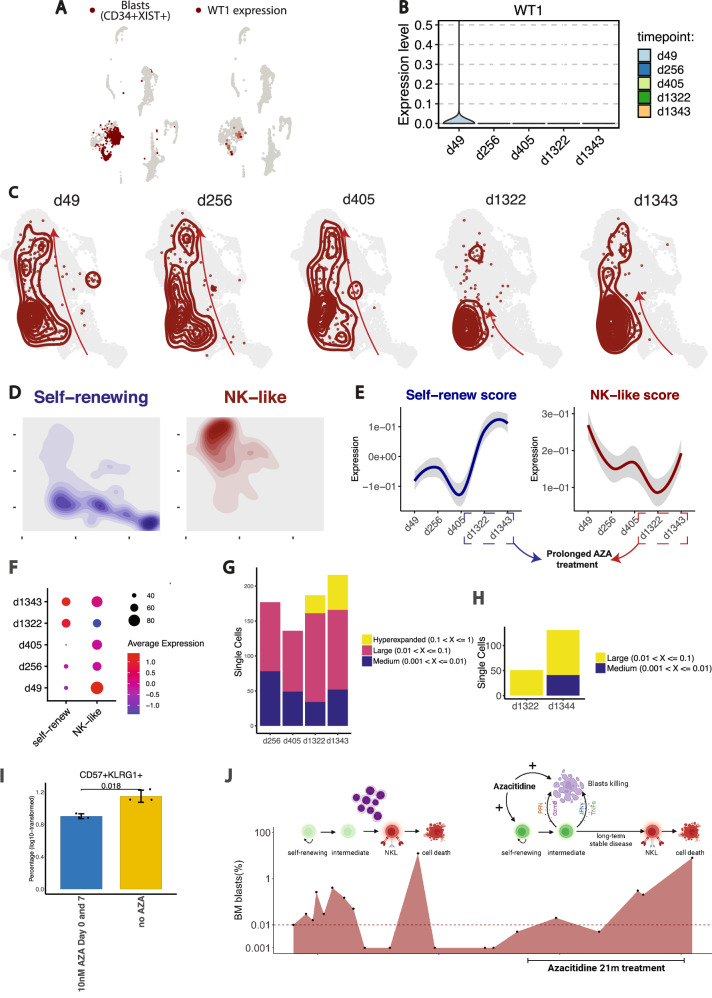
Longitudinal single-sell analysis of T_TCR-C4_ transcriptional skewing and clonal evolution in a patient with relapsed AML post-allogeneic HCT. **A** UMAP plots showing a blast score calculated from *CD34* and *XIST* (female-specific) co-expression in a patient treated with a sex-mismatched transplant (female patient, male donor), along with *WT1* expression. Cells with high scores are in dark red. **B** Violin plot of the *WT1* expression across timepoints post-T_TCR-C4_ infusion (d49, d256, d405, d1322, d1343). **C** UMAP plots of PB CD8^+^ T cells from the scRNAseq dataset, with the T_TCR-C4_ subset from patient 8 highlighted (dark red). Kernel density contours depict the density of T_TCR-C4_ cells within the CD8^+^ T cell landscape at each timepoint (d49, d256, d405, d1322, and d1343). Arrows were added manually to indicate the different skewing of T_TCR-C4_ across the timepoints examined. **D** UMAP plots of PB CD8^+^ T cells, with density contours highlighting cells with high (above 75^th^ percentile) self-renewing (*TCF7*, *LEF1*, *SELL*, *CCR7*, *BCL2*, *IL7R*, *CD27*, *CD28*; blue) and NK-like (*ZEB2*, *S1PR5*, *CX3CR1*, *KLRG1*, *NKG7*, *FCRL6*, *KLRD1*, *ADGRG1*; red) gene scores. **E** Line plots showing the temporal changes in the self-renew (left) and NK-like (right) scores of T_TCR-C4_. The blue (self-renew) and dark red (NK-like) lines represent LOESS-smoothed means of the scores, with shaded areas indicating the 95% confidence interval. The *y*-axis reflects score expression values. **F** Dot plot showing the self-renew and NK-like scores of T_TCR-C4_ across timepoints post-infusion. **G** Stacked bar plot showing cell count of PB T_TCR-C4_ within specific clonal frequency ranges over time (d256, d405, d1322, d1343). Colors indicate the clonal frequencies. **H** Stacked bar plot showing cell count of BM T_TCR-C4_ within specific clonal frequency ranges at d1322 and d1343. Colors indicate the clonal frequencies. **I** Bar plot illustrating the log10-transformed percentage of CD57^+^KLRG1^+^ T_TCR-C4_ after CD3/CD28 stimulation ± 10 nM AZA. Data are presented as mean ± standard deviation (*n* = 6, 3 biological replicates/condition). Two-sided Welch’s t-test was used for statistical testing, assuming unequal variances between groups. **J** Cartoon showing the influence of blasts and azacitidine on T_TCR-C4_. The red area under the curve represents the blasts percentage over time. Blasts induce T_TCR-C4_ skewing towards NKL and cell death; azacitidine supports self-renewal and long-term persistence. Created in BioRender. Mazziotta, F. (https://BioRender.com/twt3jx5).

Given the link between NKL differentiation and AML-induced T-cell dysfunction, we explored the state of T_TCR-C4_ in patient 8, the only patient of 4 (Supplementary Fig. 5) who exhibited prolonged T_TCR-C4_ and MRD persistence indicative of disease control despite incomplete leukemia clearance, enabling T_TCR-C4_/AML interplay analysis. This female patient underwent a non-myelo-ablative (Flu/3Gy-TBI) HCT from a male donor, with MRD (0.26% blasts by flow cytometry) detected 28 days post-HCT (Fig. 6A) and received T_TCR-C4_ (10^10^ cells/m^2^) 77 days post-HCT. AML blasts expressed detectable but limited WT1 protein pre-HCT (Supplementary Fig. 24). scRNAseq at day 49 post-infusion revealed circulating CD34^+^ female-origin blasts (XIST^+^) expressing WT1 (Fig. 7A, B). Persistent MRD and limited T-cell persistence at day 63 post-infusion (T cells declined to only 0.1 multimer^+^ CD8^+^ T cells/µl on day 60), (Figs. 6B, 6C**-**red arrow, 6D) prompted azacitidine treatment (five days every 28 days). Post-azacitidine, T_TCR-C4_ counts increased (~50 multimer^+^ CD8^+^ T cells/ul; >3% CD8 multimer^+^ T cells) without additional T_TCR-C4_ (Fig. 6D), leading to azacitidine discontinuation after seven cycles when no AML was detected. However, AML recurrence at day 414 post-infusion triggered chemotherapy (mitoxantrone, etoposide and cytarabine), additional azacitidine cycles followed by a second T_TCR-C4_ (10^10^ cells/m^2^) infusion (Fig. 6A**)**. Azacitidine was held post-infusion but restarted upon MRD detection 162 days post-second T_TCR-C4_ infusion, continuing for over 20 monthly cycles, maintaining marrow MRD below 1% for 21 months (641 days, from day 868 to day 1509) before progression was detected on day 1546 (Fig. 6A, B). During this 21-month period, blasts were detectable via scRNAseq in BM but not in PB (Fig. 7B, and Supplementary Fig. 25A, B). Throughout the azacitidine cycles, platelet counts decreased (~100 × 103 platelets/µl) mid-cycle before rising (~300 × 103 platelets/µl) immediately before the next cycle, consistent with transient azacitidine-mediated myelosuppression. Neutrophil counts, however, paradoxically increased mid-cycle (~4.5 × 103 neutrophils/µl) and decreased (~1.6 × 103 neutrophils/µl) just before the next cycle, suggesting that treatment-related AML contraction facilitated enhanced mid-cycle neutrophil production despite azacitidine’s myelosuppressive effect (Supplementary Fig. 25C). This 21-month response exceeds the typical 4-month median for post-HCT azacitidine treatment^56^, suggesting that persisting T_TCR-C4_ plus azacitidine contributed to long-term disease control.

T_TCR-C4_ transcriptomics were analyzed to identify associations with disease control. We scored genes associated with self-renewal and NK-like profiles and found that at earlier timepoints (d49, d256, and d405 post-first infusion), T_TCR-C4_ were predominantly skewed towards an NKL/Temra transcriptional state. However, at later timepoints (d1322, d1343) although T_TCR-C4_ remained along the same differentiation trajectory (from less differentiated states to NKL/Temra T_TCR-C4_), they exhibited fewer NKL features, and showed an enrichment of self-renewal markers (Fig. 7C–F). scTCRseq analysis of PB at days 265, 405, 1322, 1343 post-first T_TCR-C4_ infusion revealed progressive clonal expansion of T_TCR-C4_ (Fig. 7G). T_TCR-C4_ clonal expansion peaked during the 21 months of continued azacitidine treatment, suggesting that during this phase, T_TCR-C4_ were capable of both self-renewal, which in turn facilitated their long-term persistence, and antigen recognition, which triggered further expansion and disease control. Similarly, BM scRNAseq revealed clonal expansion of T_TCR-C4_ between d1322 and d1344 post-first infusion (Fig. 7H) despite no additional T_TCR-C4_. To reproduce this in vitro, we induced T_TCR-C4_ differentiation (see “Methods”) in the presence or absence of azacitidine. After 14 days of stimulation, we observed that the presence of azacitidine significantly (*p* < 0.05) reduced the skewing towards CD57^+^KLRG1^+^ T_TCR-C4_ (Fig. 7I, and Supplementary Fig. 26, Supplementary Table 13). CD8^+^CD57^+^KLRG1^+^ cells have previously been shown to exhibit an NKL transcriptional profile^25^ and reduced leukemia cell killing capabilities^24^. With the caveat that the evidence is based on one patient, these findings suggest that azacitidine-exposed T_TCR-C4_, retained self-renewing (versus NKL) features, which may have supported their long-term persistence and enhanced AML-targeting efficacy that may have contributed to long-term disease control (Fig. 7J).

## Discussion

We previously demonstrated that EBV-specific T_TCR-C4_ infusion appears to prevent AML recurrence leading to a survival advantage in patients at high risk of post-HCT relapse^13^. In the current study, among 15 AML patients who received T_TCR-C4_ post-HCT relapse, we observed indirect evidence supporting anti-leukemic activity but found no survival advantage in patients receiving either EBV- or CMV-specific CD8^+^ T cells engineered to express TCR_C4_. We analyzed the AML/T_TCR-C4_ interplay to investigate how AML affects antigen-specific T cells and identified T-cell characteristics associated with AML control in one case.

The characteristics of substrate cells from which T cell products are derived can impact post-transfer persistence. In murine and macaque models, antigen-specific Tcm-like CD8^+^ T cells persist long-term post-adoptive transfer, unlike Temra-like cells^30,57^. In our study, T_TCR-C4_ generated from EBV-specific CD8^+^ T cells persisted longer compared to CMV-specific substrate cells which may reflect the distinct biology of EBV and CMV^58,59^. CMV reactivates periodically, requiring rapidly activated responses that promote Temra differentiation^60,61^, while EBV tends to remain quiescent, preserving Tcm phenotypes^62–64^. While reactivation frequency plays a role, the reactivation microenvironment is also critical. CMV reactivates in non-professional antigen-presenting cells, including fibroblasts, which lack co-stimulatory signals and favor Temra differentiation, wheras EBV reactivates in B cells in lymphoid tissues, providing co-stimulation that may preserve the Tcm phenotype^65,66^. Our analysis of an independent mass-cytometry dataset^31^ confirmed that EBV-specific cells better maintain self-renewing features than CMV-specific cells. Taken together these findings establish that self-renewal capacity may predict the potential for prolonged T-cell persistence^30^. However, EBV-specific T_TCR-C4_ were unable to sustain long-term responses in all patients, suggesting additional influences by non-intrinsic AML-related factors.

Despite efforts to investigate T-cell dysfunction in AML^23,24,50^, several critical questions have remained unanswered^27^, mainly due to the challenge of identifying AML-specific T cells. In this study, infused T_TCR-C4_ offered the possibility of examining AML-specific T cells and AML biology. We found that T-cell exhaustion is not the primary cause of AML-specific T-cell dysfunction. Instead, T_TCR-C4_ expressing exhaustion markers (PD1, TIGIT, Tim3) also expressed markers of activation (CD38, CD69) and proliferation (Ki67), indicating an effector-like T-cell state. We found that AML-exposed T cells follow a differentiation trajectory from effector-memory to NKL cells, which was associated with compromised T_TCR-C4_ cell persistence and function. These findings are supported by previous works highlighting the importance of T-cell senescence and NKL transition, in addition to exhaustion, as potential mechanisms underlying CAR-T cells dysfunction^26,40^.

AML’s distinct properties, including myeloid cell origin and immunosuppressive effects^67^ via production of reactive oxygen species and other soluble factors, may distinctly redirect T cells toward functional impairment that precludes transitioning to a classical exhausted phenotype and explain the observed NKL skewing in T_TCR-C4_ and endogenous CD8^+^ T cells. The absence of classical exhausted cells in AML has several implications. In solid tumors, chronic antigen stimulation induces a multi-step epigenetic shift of CD8^+^ T cells into an exhaustion state^68^, starting from PD1^+^TCF1^+^ precursor/progenitor exhausted cells, whose frequency correlates with response to checkpoint inhibitors^69^. In contrast, our AML findings suggest that the expression of common activation/exhaustion markers reflect recent T-cell activation rather than true exhaustion, which may also explain the limited efficacy of checkpoint inhibitors in AML^23,50,70,71^. The presence of a distinct dysfunctional program supports the need for new immunotherapy strategies to enhance the efficacy of anti-AML adoptive T-cell therapy to prevent T-cell dysfunction. For example, knocking out ID3 and SOX4 transcription factors in chimeric antigen receptor-engineered (CAR)-T cells in vitro reduces NKL skewing and enhances effector functions^26^. Whether these strategies can improve cell therapy in AML in vivo remains to be determined.

Contrary to a previous report^36^, we did not detect serum cytokine changes linked to NK-like skewing. This may reflect the limited sensitivity of serum cytokine levels in capturing T_TCR-C4_ effects within the tumor microenvironment, compared to the pronounced systemic effects of CAR-T cells. Indeed, CAR-T cells in AML carries a higher incidence of CRS^72^ compared to TCR-T cells^13^.

A potential strategy to reduce NKL skewing of T_TCR-C4_, as suggested by the outcome in one of our treated patients, could involve the use of the hypomethylating agent azacitidine. While azacitidine has direct anti-leukemia effects^73^, the prolonged disease control observed in our patient was unusual and prompted further investigation into the underlying mechanisms. Azacitidine administration correlated with clonal expansion and persistence of central memory, as opposed to NKL differentiated T_TCR-C4_, leading to a prolonged equilibrium between anti-leukemic T_TCR-C4_ and AML MRD. In support of this, a previous study investigating azacitidine combined with nivolumab in AML patients relapsed post-HCT^74^ showed that following the interruption of azacitidine and the initiation of nivolumab, CD8^+^CD57^+^KLRG1^+^ T cells significantly increased over time in non-responders. In our study, uninterrupted administration of azacitidine reduced the skewing in NKL (in vivo) and CD57^+^KLRG1^+^ (in vitro) CD8^+^ T cells, suggesting that a prolonged treatment with azacitidine in addition to having anti-leukemic activity may be beneficial to avoid terminal differentiation and support T_TCR-C4_ persistence. Other studies have also shown that azacitidine can have a negative effect on T regulatory cells^75^, and enhance CAR-T cell activity towards AML^76^. Additionally, in our patient, before receiving the first T-cell infusion, the low WT1 expression in the residual AML likely did not activate T_TCR-C4_, causing near disappearance of the T cells. However, after azacitidine introduction, T_TCR-C4_ frequencies increased, likely driven by azacitidine-induced WT1 expression^77^. Intermittent T_TCR-C4_ activation by transient induction of WT1 presentation on AML cells by cycles of azacitidine with subsequent temporary clearance of AML may have helped sustain T_TCR-C4_ persistence and response to stimulation. Although further investigation is needed to determine if azacitidine acts preferentially on leukemia cells by increasing WT1 expression, thereby promoting antigen-recognition, and/or T_TCR-C4_ effector functions, these results support evaluating azacitidine as a favorable adjunct to T-cell immunotherapy.

Paradoxically, our ability to track dysfunction was limited to patients with persisting T_TCR-C4_, in whom T cells remained relatively functional. We hypothesize that patients with more aggressive disease experience accelerated T-cell dysfunction and death rather than establishment of progenitor cells capable of self-renewal; however we were unable to formally test this in our study. Despite a limited sample size, our findings on the nature of T-cell dysfunction in AML were confirmed in larger cohorts of endogenous CD8^+^ T cells. Additionally, our interpretation of azacitidine’s effect on T_TCR-C4_ is based on a single patient and thus should be viewed as preliminary.

In our study, most of the described patients did not undergo lymphodepletion prior to T cell infusions, as this was introduced into the trial only after the safety of T_TCR-C4_ was established. Lymphodepletion could potentially favor cell product expansion and persistence^78^. However, of the three patients who did receive lymphodepletion in this study, only one demonstrated long-term T_TCR-C4_ persistence. In the prophylactic setting of our previously reported trial^13^, no patient underwent lymphodepletion, yet all patients experienced sustained remissions. These findings suggest that the efficacy of T_TCR-C4_ may be influenced more by post-HCT AML status rather than preceding lymphodepletion.

Further validation in larger, randomized trials will be needed to confirm and better define the mechanisms of AML-induced T-cell dysfunction and to clarify whether the limited efficacy of T_TCR-C4_ in this study reflects an insufficient therapeutic effect of WT1 targeting in active disease or whether cellular therapies are generally less effective in this setting compared to the prophylactic context^13^. However, our study does illuminate some of the complex mechanisms underlying AML-induced T-cell dysfunction and strengthens support for a distinct pathway outside the traditional paradigm of T-cell exhaustion, emphasizing the need to address this unique dysfunction in future immunotherapy strategies.

## Methods

### Clinical protocol

The trial was approved by the Fred Hutchinson Cancer Center (FHCC) Institutional Review Board, the US Food and Drug Administration and the National Institutes of Health Recombinant DNA Advisory Committee. The study was conducted in accordance with the Declaration of Helsinki and was registered at ClinicalTrials.org under the identifier NCT01640301 on July 13, 2012. All participants and donors provided written informed consent prior to enrollment. Eligible participants included ‘high-risk’ AML patients with relapsed or refractory disease (overt or MRD) post-HCT along with their fully HLA-matched (10 of 10) related or unrelated donors expressing HLA A*0201 (HLA-A2). The primary endpoints were safety and toxicity, assessed by the nature and severity of adverse events related to the study treatment, and disease response, evaluated in patients with active disease (MRD or overt relapse). Secondary endpoints included the persistence and migration of transferred T cells to the bone marrow, the maintenance of TCR expression and function in transduced T cells, and clinical outcomes following T-cell therapy.

### Patient selection

HLA-A2 genotype was confirmed by high-resolution typing before enrollment. Exclusion criteria included: refractory central nervous system disease, HIV seropositivity, grade ≥ 3 GVHD and no available CMV/EBV-seropositive matched donor. The sample size for this study was not based on formal power calculations, but on feasibility, the potential to provide descriptive information, determine whether further study was warranted and evaluate toxicity. Sex and age were not considered in the study design due to the sample size and the exploratory nature of the study. Accordingly, no sex- or age-based analyses were performed. The sex and age of participants were recorded based on clinical documentation and are reported in aggregate. Consent to specifically report or share individual-level data, that could potentially identify the patients based on age, sex, institution of treatment and disease was not obtained. Participants were not compensated for their participation in this study. The first participant was enrolled on April 29, 2013 and the last participant was enrolled on February 1, 2019.

### Treatment plan

Isolation of TCR_C4_, lentiviral vector construction, and T_TCR-C4_ generation were performed as previously described^13^. Patients were eligible to receive a first infusion of T_TCR-C4_ after demonstrating relapse (overt or MRD) at any time post allogeneic HCT. Patients 26, 27, 28 received lymphodepleting treatment before the first T_TCR-C4_ infusion with cyclophosphamide (300 mg/m^2^ IV) and fludarabine phosphate (30 mg/m^2^ IV) daily on days -4 to -2. Patients 1, 2, 4, 5, 6, 7, 8, 9 received four escalating doses of T_TCR-C4_ (patient 3 received the cells on Arm 1) starting at 10^9^ cells per m^2^ on day 0, then 3.3 × 10^9^ cells per m^2^ on day 14 and 10^10^ cells per m^2^ on days 28 and 42. followed by low-dose subcutaneous IL-2 (2.5 × 10^5^ IU twice daily) for 14 d (stage 1) administered to enhance the survival of transferred T cells. After safety was established, the remaining 7 patients received 2 doses of 10^10^ cells per m^2^ on day 0 and on day 28 followed by 14 days of low-dose subcutaneous IL-2. A second T_TCR-C4_ infusion was administered only if the frequency of T_TCR-C4_ was <3% of total peripheral CD8^+^ T cells. Patients 5, 7, 19, 23, 27 received 1 infusion, patients 4, 8, 14, 15, 26, 28 received 2 infusions, patient 1 received 3 infusions, the remainder received 4 infusions (Table 1). Patients were monitored for toxicities, based on Common Toxicity Criteria v.4.0. Treatment was declared safe based on the toxicity rate ( < 30%). Non-hematologic toxicity requiring treatment discontinuation was defined as any grade 3 or 4 non-hematologic toxicity (CTCAE 4) that was deemed to be caused by infusion of the study treatment. Hematologic toxicity requiring treatment discontinuation (blood/bone marrow CTCAE 4) was defined as any new or recurrent onset of grade 4 hematologic toxicity that occurred after the first T cell infusion, and lasted for two consecutive days, and was attributed to any other identifiable cause other than T cell infusion. Exceptions included grade 4 lymphopenia if it returned to pre-infusion levels within 14 days, as a transient drop in lymphocyte counts was expected after T cell infusions, and platelet counts < 20,000/mm^3^, which are common in this post-HCT population.

### Assessment of disease status

Morphology, multiparameter flow cytometry, standard cytogenetics or genomic technologies were routinely performed on bone marrow aspirates and peripheral blood samples that were obtained from all patients. Any level of residual disease was considered to indicate positivity for MRD.

### T cell tracking by WT1 peptide/HLA (pHLA) tetramers

WT1 pHLA-specific tetramers (produced by the FHCC Immune Monitoring Core Facility) were used to detect T_TCR-C4_ in PBMCs collected after infusions, with a staining sensitivity of 0.01% of total CD8^+^ T cells, as previously described^13^. T_TCR-C4_ percentages were calculated using FlowJo v.10 (Treestar).

### Patient outcomes and survival analysis

Kaplan-Meier OS curves were estimated using the *survminer* (v0.4.9) and *survival* (v3.3-1) packages in R. OS was calculated from the date of first T_TCR-C4_ infusion to the date of death or censoring. Outcomes of responding patients were represented using a swimmer plot. Day 0 was defined as the post-HCT relapse date or, for HCT-refractory patients, as day 28 post-HCT, at which timepoint persistent disease was observed. R package *swimplot* (v1.2.0) was used for visualization.

### Analysis of T_TCR-C4_ persistence

Persistence of TCR-T cells (%T_TCR-C4_) over time was visualized using *ggplot2* (v3.4.4) R package. Wilcoxon rank-sum tests were used for pairwise comparisons between T_TCR-C4_ with different virus-specificity (EBV vs. CMV) over time. Kruskal-Wallis tests were applied for multi-group comparisons.

Fisher’s exact test was used to evaluate the association between disease risk and T_TCR-C4_ persistence. The test was chosen due to the small sample size and binary classification of persistence (long-term vs. short-term) and disease risk (disease + /early post-HCT relapse vs. MRD-/non early post-HCT relapse). Statistical significance was assessed with a *p*-value threshold of 0.05. One patient was excluded from this analysis due to the unavailability of WT1-expression data, which could confound interpretation of the persistence results.

To correlate quantitative parameters before infusion with T_TCR-C4_ persistence we used first a logistic regression model to evaluate the association between persistence and absolute white blood cells, neutrophils, lymphocytes, monocytes, eosinophils, basophils, and blasts percentage. Next, we fit lasso regression using the *glmnet* R package to identify key predictors of persistence. Cross-validation was performed to determine the optimal lambda parameter, and model coefficients were extracted at the optimal penalty value.

### Flow-cytometry

Cryopreserved PBMCs were thawed and allowed to rest overnight in RPMI medium supplemented with 10% fetal bovine serum (R10). The cells underwent stimulation with a cocktail containing the WT1_126-134_ peptide at a final concentration of 1 μg/ml in R10 and intracellular cytokine staining, as previously described^13^. Flow cytometry was conducted on an LSRII instrument (Becton Dickinson) with data acquisition using FACS-Diva software v.8.0.1. Flow cytometry data were subsequently analyzed using FlowJo v.10 (Treestar).

To test the overall decline of IFNγ production over time in T_TCR-C4_ (tetramer positive) cells and account for repeated measures from the same subjects, we employed a linear mixed-effects model using the lmer function from the lmerTest package. This model included Timepoint as a fixed effect and subject IDs as a random effect. To ensure the appropriate distribution for analysis, the percentage values were log-transformed (using log10) to stabilize variance and normalize the data. We modeled the relationship between log10-transformed percentages of tetramer positive IFNγ-producing cells and the Timepoint factor, with T0 serving as the reference level (intercept) for comparison.

The full formula used to fit the model was: lmer(Tet.pos_IFNg ~ Timepoint + (1 | id), data = df), where Tet.pos_IFNg represents the log10-transformed percentages of tetramer positive IFNγ-producing cells, Timepoint is the timepoint, id is the subject IDs, and df is the dataset employed (Supplementary table 6). All statistical analyses were conducted using R (v4.3.2), with results considered significant at *p* < 0.05.

### Spectral flow cytometry

Post-infusion PB samples were analyzed using a 5-laser Cytek Aurora. Antibodies used are listed in Supplementary Table 4. T_TCR-C4_ were identified by binding to the APC dye-labeled HLA-A2:WT1_126-134_ tetramer. Spectral flow-cytometry data were biexponentially transformed, compensated and preprocessed (aggregates and dead cell removal) in FlowJo V10 (TreeStar). Pregated CD8^+^ T cells were exported from FlowJo and loaded in R (v4.3.2). First we created a flowSet using *flowCore* (v2.12.2)^79^ and subsequently analyzed the data using *CATALYST* (v1.24.0)^80^.

Ki67 was then used to manually gate subsets of interest using the FlowJo software. Statistical analysis included Kruskal-Wallis to compare T_TCR-C4__Temra and T_TCR-C4__Tem percentages over time, and Wilcoxon rank-sum test for comparing KLRG1^+^CD57^+^GNLY^+^ and Tim3^+^PD1^+^TIGIT^+^ T_TCR-C4__Temra.

### In silico mass cytometry validation

Mass cytometry files used to generate an atlas of virus-specific CD8^+^ T cells^31^ were downloaded from(https://zenodo.org/records/8330231).Data were analyzed using *flowCore* and *CATALYST* R packages as specified above.

### Serum cytokine analysis

Quantitative cytokine analysis was performed at our institution using a Luminex-based multiplex immunoassay. Serum samples were collected, processed, and stored at -80 °C prior to analysis. Cytokine concentrations were measured using a Luminex instrument following the manufacturer’s protocols. Results were reported as concentration values (pg/mL). Statistical analysis was performed in R (v4.3.2), with the Wilcoxon rank-sum test used for pairwise comparisons between timepoints and the Kruskal-Wallis test applied to study the overall concentration changes over time.

### Single Cell RNA Sequencing

Patients with T_TCR-C4_ ≥ 3% of the total CD8^+^ T cells at day 28 or later after first infusion were selected for scRNAseq analysis. Available PBMCs or BMMCs were thawed, washed and loaded on a 10x Chromium Controller based on the 3’ Chromium or 5’ Chromium Single Cell V(D)J Reagent Kit manual (10x Genomics). Library preparation was performed as per manufacturer’s protocol with no modifications. Library quality was confirmed by TapeStation High Sensitivity (Agilent, evaluates library size), Qubit (Thermo Fisher, evaluates dsDNA quantity), and KAPA qPCR analysis (KAPA Biosystems, evaluates quantity of amplifiable transcript). Samples were mixed in equimolar fashion and sequenced on an Illumina HiSeq 2500 rapid run mode according to the standard 10X Genomics protocol. TCR target enrichment, 5’ gene expression library, and TCR library were carried out according to the 5’ Chromium Single Cell V(D)J Reagent Kit manual (10x Genomics). The 10X Genomics software Cell Ranger (v2.0.0) was used to process the raw data FASTAQ files with default parameters. The EmptyDrops method^81^ was used to identify cells with low RNA contents. The “count” function was used to perform alignment, filtering, barcode counting and UMI counting. Reads were aligned to the hg38 human reference genome (Ensembl) and the known transgene codon-optimized sequence using Spliced Transcripts Alignment to a Reference (STAR)^82^.

For V(D)J sequencing assembly and paired clonotype calling, we used CellRanger “vdj” function. This function leverages Chromium cellular barcodes and UMIs to assemble V(D)J transcripts for each cell. CellRanger V(D)J calling produces an output named “filtered_contig_annotations.csv” for each sample, which lists CDR3 amino acid and nucleotide sequences for single cells identified by their barcodes.

### scRNAseq quality control and subsetting

The filtered feature matrices generated by the CellRanger pipeline were used for downstream quality control (QC) and analyses. We used the function read10xCounts from the R package *DropletUtils* (version 1.14.2) to load the CellRanger output in R as a SingleCellExperiment^83^ object. Doublet cells filtering was performed on each sample using the *scds* package (v1.10.0)^84^. QC and filtering were conducted using the *scater* R package (v1.22.0)^85^. Genes with zero counts across all cells were removed from the analysis. This was achieved by filtering out genes that had no detected expression in any cell (row sums of counts equal to zero). Cells were filtered based on feature counts, the percentage of mitochondrial and ribosomal genes, and the number of expressed features. Cells with values beyond a specific threshold, between 1 and 3.5 median absolute deviations (MAD) from the median, were excluded. These MAD thresholds were established according to the quality of each sample. Features with a count greater than 1 in at least 3 cells were retained for downstream analysis. We then split cells by sample in 15 datasets, normalized, found the 2000 most variable genes and scaled for each dataset using *Seurat* (v4.3.0.9001) SplitObject, NormalizeData, FindVariableFeatures and ScaleData respectively^86,87^. For batch correction, we used the FindIntegrationAnchors and IntegrateData functions from *Seurat*. The integrated dataset was then used for scaling (ScaleData) and dimensionality reduction (PCA and UMAP) using RunPCA and RunUMAP, respectively. UMAP dimension reduction and clustering were computed using the first 20 principal components (PCs). The number of PCs capturing most of the variation in our data was selected using *Seurat* function ElbowPlot which visualizes the standard deviation of each PC. Clusters were identified via shared-nearest-neighbor-based (SNN) clustering and further analyzed at a resolution of 0.6. To assess whether NK cells were depleted after T_TCR-C4_ infusion, we used the scGate tool^37^ to identify NK cells as positive for *NCAM1, KLRD1, KLRG1*, and negative for *CD3D, CD3G*. NK cells constituted ~10% of total peripheral blood cells, indicating no significant depletion. CD8^+^ cell subset was identified using scGate (v1.0.1) and subsequently extracted with Seurat subset function.

### scRNAseq CD8^+^ endogenous and T_TCR-C4_ analysis

The CD8^+^ subset was used to find the 2000 most variable features (FindVariableFeatures), scale the data (ScaleData), and run dimensionality reduction (runPCA and runUMAP). SNN was used for clustering with a resolution of 0.5 for downstream analysis. Differential gene expression across and between clusters or condition (e.g. AML(+) vs AML(-)) was computed using *Seurat* FindMarkers and FindAllMarkers with default parameters. TCR_C4_-transgene^+^ cells were identified using *scGate* (v1.0.1). For visualization purposes, we used Seurat built-in functions alongside *ComplexHeatmap* (v2.15.1)^88^, *scCustomize* (v1.1.0.9001) (https://github.com/samuel-marsh/scCustomize) and *SCP* (https://github.com/zhanghao-njmu/SCP). The pan-cancer CD8^+^ single-cell reference atlas was built using the CD8.thisStudy_10X.seu.rds file^41^ downloaded from Zenodo and processed using the make.reference function from the *ProjecTILs* R package (v3.2.0)^89^. We then used Run.Projectils function, from the same package, to project the cell states from the query dataset onto the reference.

Cell trajectory inference was computed using the *Monocle*^45^ R package (v1.3.4), with UMAP used as dimensionality reduction. Single-gene expression patterns along the pseudotime were visualized using the plot_genes_in_pseudotime function in *Monocle*. RNA velocity was conducted by exporting the CD8^+^ T-cell Seurat object as an h5ad file using the R package *seurat-disk* (v0.0.0.9020) (https://github.com/mojaveazure/seurat-disk), loading this file in Python (v3.8.14) as AnnData object^90^, and estimating velocities with the *scvelo* Python package (v0.2.4)^46^ using the deterministic model.

We classified patients as AML(+) or AML(-) based on the presence of detectable blasts in BM or PB. Three patients lacked BM or PB evaluations at one timepoint each. For two of these patients, we used the donor-recipient (male-female) sex mismatch. Patient 8 at day 49 exhibited cells expressing the female-specific gene *XIST* alongside the AML-associated genes *CD34* and *WT1* **(**Supplementary Fig. 18A**)**; similarly, patient 26 expressed *XIST* in a *RPS4Y1* (male-specific gene) negative region at days 7 and 28, thus classified as AML(+) at these timepoints **(**Supplementary Fig. 18B**)**. Patient 4 was categorized as AML(-) at day 100 and AML(+) at day 581, as previously reported^49^.

To assess the significance of differences in cell proportions per CD8^+^ cell state between groups (AML(+) vs AML(-)), a permutation test was applied. Specifically, the permutation_test and permutation_plot functions from the R package *scProportionTest* (version 0.0.0.9000) (https://github.com/rpolicastro/scProportionTest) were used with default parameters.

For scRNAseq in silico validation, we compiled scRNAseq datasets from independent studies on AML^23,25,50–52^, lung cancer^55^, pancreatic cancer^53^ and melanoma^54^. CD8^+^ T cells were identified and extracted using the *scGate* R package. These cells were then projected onto the pan-cancer CD8^+^ T cell reference atlas^41^ using *ProjecTILs* R package.

Signatures of T-cell dysfunction^25,47,48^ and KLR-exhaustion^42,43^, were scored and integrated into our CD8^+^ T-cell Seurat object using *Seurat* ’s AddModuleScore. These signatures were then projected onto the CD8^+^ two-dimensional UMAP or visualized using DotPlot in *Seurat* to assess expression patterns.

To assess changes in TCR-signaling as T_TCR-C4_ undergo NK-like skewing, we first identified the NKL T_TCR-C4_ and the non-NKL T_TCR-C4_. This was achieved by extracting the UMAP coordinates of T_TCR-C4_ and endogenous NKL/Temra and computing pairwise Euclidean distances between them. Cells within a defined threshold distance (0.1) were considered overlapping. T_TCR-C4_ overlapping with endogenous NKL-Temra were labeled T_TCR-C4__NKL and visualized using *Seurat* ’s DimPlot. To identify transcriptional differences between NKL T_TCR-C4_ and non-NKL T_TCR-C4_ cells, we performed differential expression analysis using *Seurat* ’s FindMarkers. Genes were ranked based on their log fold change and adjusted *p*-values, and GSEA was conducted using *fgsea* (v1.28.0) to evaluate the enrichment in KEGG TCR signaling pathway. To further assess the expression of key TCR signaling genes, we generated dot plots for CD3D, CD3E, CD3G, LCK, ZAP70 and LAT. Additionally, we used the KEGG TCR signaling pathway and *Seurat* ’s AddModuleScore to compute a TCR signaling score, and then compare its expression between non-NKL T_TCR-C4_ and NKL T_TCR-C4_.

The TCR repertoire was analyzed using the R package scRepertoire (v1.10.1)^91^. Initially the degree of single cells clonal expansion was defined based on the number of cells sharing the same clonotype. The function occupiedscRepertoire was used to visualize the degree of clonal expansion by cell-state over time. To infer clonal trajectories, we first identified hyperexpanded and large clones and mapped them to their respective clusters and UMAP coordinates. We then tracked clones that appeared in multiple clusters, starting from their least abundant state and progressing to their more expanded forms. Finally, centroids for each clone in different clusters were calculated, and directional vectors were generated to infer the movement of these clones.

### In vitro chronic antigen stimulation model

The in vitro dysfunction model was established as previously described^26^. The WT1^+^ K562 tumor cell line (CCL-243, obtained from the ATCC) was transduced with lentiviral constructs to express HLA-A*02:01 and GFP, then sorted for purity using the Sony MA900 cell sorter. Cells where then cultured in media consisting of IMDM with GlutaMAX (Gibco, Life Technologies, #31980030) with 10% FBS, and 1% of penicillin/streptomycin.

T_TCR37-45_ cells were obtained following previously published protocols^49^. We selected TCR-T cells targeting WT1_37-45_ as K562 cell line primarily express the standard proteasome and is not lysed by T_TCR-C4_ targeting WT1_126-134_^49^. T cells expressing an irrelevant virus-specific TCR were used as negative control. Co-cultures of T cells with K562 cells were established at a 1:1 and 1:4 E:T ratio (2.5 × 10^5^ T cells, 2.5 × 10^5^ or 1 × 10^6^ tumor cells, respectively). After 3-4 days of coculture, 250 μl of the cell suspension was used for T cell counting and flow-cytometry staining. The remaining cell suspension was spun down, and cells were resuspended in fresh media. A Novocyte 3 lasers flow cytometer was used to quantify GFP^+^ tumor cells and T cells and to maintain constant E:T ratios by reseeding K562 cells. Notably, during the peak of T-cell expansion (day 9–13), the volumes of the cocultures were reduced to ensure the reseeding of an adequate number of tumor cells, thereby maintaining constant E:T ratios. This protocol was followed for 23 days.

### Bulk RNA sequencing

Bulk RNA sequencing was performed using BGISEQ-500 platform at BGI Genomics. Briefly, total RNA was extracted using the Qiagen RNeasy Micro Kit according to the manufacturer’s instructions. For the construction of low input polyA mRNA-seq libraries, the *SMARTseq* (v4) Package was used. Sequencing was performed on a DNBseq T7 machine (MGI) with paired-end 150 bp reads, generating 30 M raw reads per sample. Raw sequencing data were filtered and trimmed using the software Soapnuke developed by BGI Genomics. The filtered reads were then aligned to the reference transcriptome using *Bowtie2* (v2.2.5). Gene read counts were subsequently generated from the alignment results using *RSEM* (v1.2.8).

To assess differences over time across conditions (T_TCR37-45__D0, K562_D14, and K562_D23), we used the likelihood ratio test (LRT) with a full model of ~Condition and a reduced model including only the intercept (reduced = ~ 1). Normalized counts were obtained using the rlog transformation, and differential expression analysis was performed using the R package *DESeq2* (v1.42.0)^92^. We manually curated a list of genes of interest (Supplementary Table 14) and filtered the results to retain only those genes that were significant (padj <0.05) with an absolute log2 fold-change > 1). For visualization purposes, average normalized counts of biological replicates were calculated using the avereps function from the R package *limma* (v3.56.2) and visualized using the R package *pheatmap* (v1.0.12). To visualize the expression patterns of manually curated gene signatures, we calculated z-scores for the averaged normalized counts across conditions (T_TCR37-45__D0, K562_D14, and K562_D23). Finally, to compare the enrichment of scRNAseq-derived gene signatures (top 50 differentially expressed genes) and of a manually curated exhaustion signature across conditions, we used the *hciR* (v1.7) function fgsea_all with default parameters and plot_fgsea for plotting.

### In vitro 5-Azacytidine treatment of CD8^+^ T_TCR-C4_ cells

A total of 1 × 10^6^ CD8^+^ T_TCR-C4_ cells were seeded on day 0 and treated with 10 nM or no Azacytidine on days 0 and 7. To activate and drive differentiation of the T cells, T Cell Transact (Miltenyi, 714 130-111-160) was added on days 1 and 8. CD8^+^ T_TCR-C4_ cells were derived from healthy donor PBMCs, and the experiments were performed using independent biological replicates. Flow cytometry analysis was performed on a Symphony A3 715 cytometer at multiple time points: day 0, day 7, and day 14. Data were analyzed using R, where CD57^+^KLRG1^+^ cell frequencies where log10-transformed and normality was assessed by the Shapiro Wilk test. Statistical analysis (Welch’s t-test) and visualization were conducted using the ggpubr (v0.6.0.999) package.

### Reporting summary

Further information on research design is available in the Nature Portfolio Reporting Summary linked to this article.

## Supplementary information

Supplementary Information

Reporting Summary

Transparent Peer Review file

## Source data

Source Data

## Data Availability

The gene expression data, including de-identified BAM files and count matrices, generated in this study have been deposited in the NCBI Gene Expression Omnibus (GEO) under accession number GSE285214. Previously published mass cytometry data analyzed in this manuscript are available on Zenodo. Previosly published AML scRNAseq data analyzed in this study are available from the European Genome-Phenome Archive (EGA) (https://ega-archive.org) under the accession numbers EGAS50000000357, EGAS00001004444 and EGAS00001004894; the NCBI’s Database of Genotypes and Phenotypes (dbGaP; https://www.ncbi.nlm.nih.gov/gap) under accession number phs003015.v1.p1; and the GEO repository under accession numbers GSE128933 and GSE185381. Previously published solid tumor scRNAseq data are available in the GEO repository under accession number GSE215121 (melanoma), GSE148071 (lung), and GSE211644 (pancreas). The remaining data ara available within the Supplementary Information, or Source Data file. Source data are provided with this paper.
