# Supplementary material for: A phase I/II trial of WT1-specific TCR gene therapy for patients with acute myeloid leukemia and active disease post-allogeneic hematopoietic cell transplantation: skewing towards NK-like phenotype impairs T cell function and persistence
Source: Nat Commun. 2025 Jun 5;16:5214. doi: 10.1038/s41467-025-60394-0 (PMC12141728; doi:10.1038/s41467-025-60394-0)
Supplement: Supplementary file 1 — Supplementary Information [file 41467_2025_60394_MOESM1_ESM.pdf]

Supplementary Figure 1

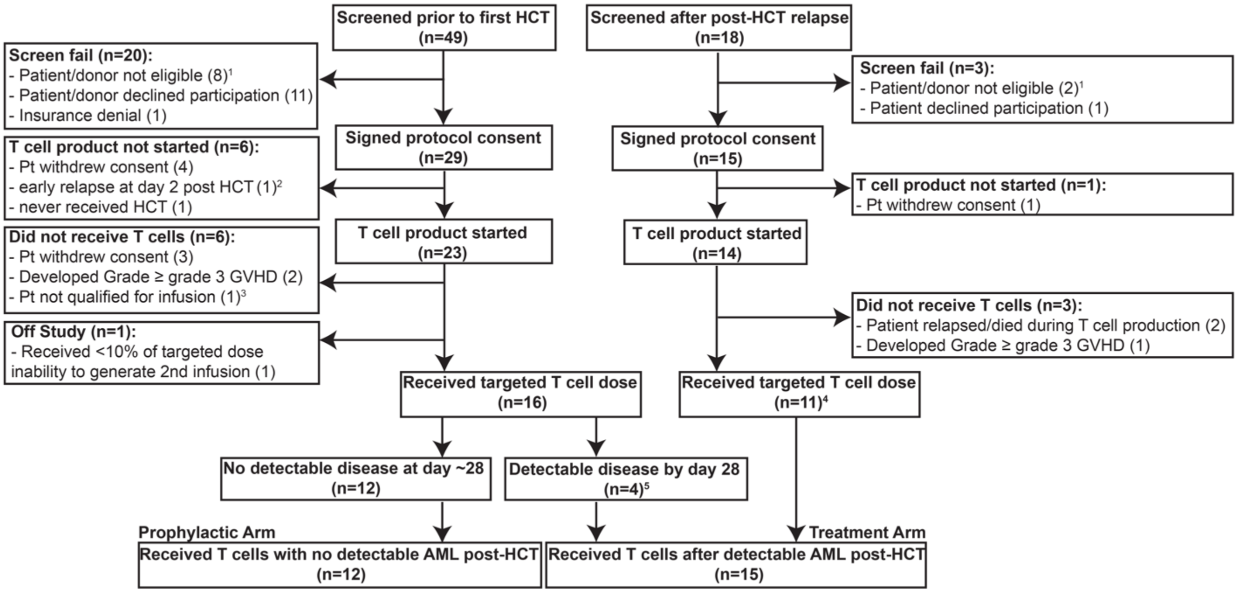

[1]Reasons included patient or donor not HLA A*0201, allele mismatch, or donor EBV and CMV sero-negative.
[2]Patient relapsed day 2 after HCT and died of progressive disease.
[3]Patient had ongoing infections and never met criteria for T_{TCR-C4} infusions.
[4]Patient screened/enrolled after they had relapsed post-HCT.
[5]Patients screened prior to their HCT, all had detectable AML before or at day 28 post-HCT and thus were not eligible for Arm 1.

Supplementary Figure 1. Flow diagram of patients enrolled on the clinical study. Follow-up of the 67 patients screened for participation in the study and the 44 patients who signed the informed consent. Patients with no detectable disease post-HCT were assigned to the Prophylactic Arm[1], while those with evidence of disease post-HCT were treated in the Treatment Arm described here. Between April 2013 and February 2019, a total of 44 pt/donor pairs were enrolled, among whom 12 disease-free (Arm 1) and 15 relapsed/refractory (Arm 2) patients received TCR_{C4} transduced cells.

Supplementary Figure 2

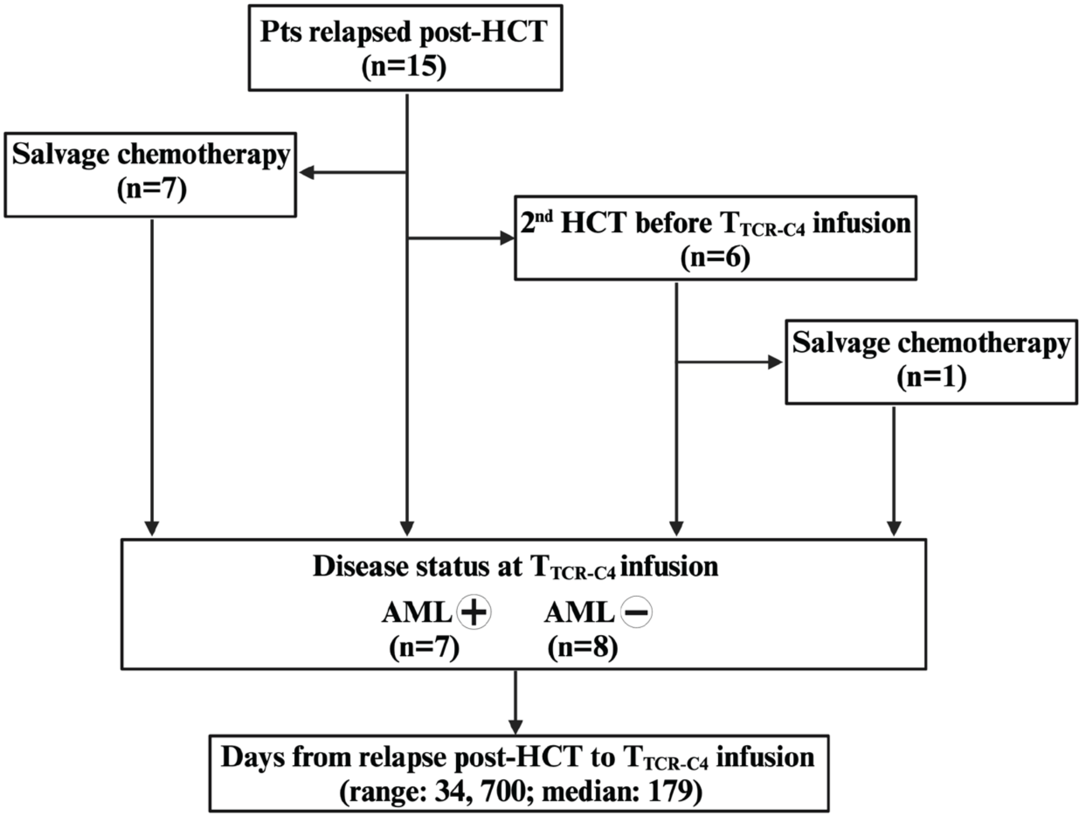

Supplementary Figure 2. Flow diagram of patients' treatment history. The cohort included 15 patients who relapsed after HCT. 7 patients received salvage chemotherapy before the infusion of $T_{TCR\text{-}C4}$, while 6 patients had two HCTs before the infusion, and 1 patient among them also received salvage chemotherapy. At the time of $T_{TCR\text{-}C4}$ infusion, 7 patients had detectable AML, while 8 patients were in remission (AML-negative). The time from relapse post-HCT to $T_{TCR\text{-}C4}$ infusion ranged from 34 to 700 days, with a median of 179 days.

Supplementary Figure 3

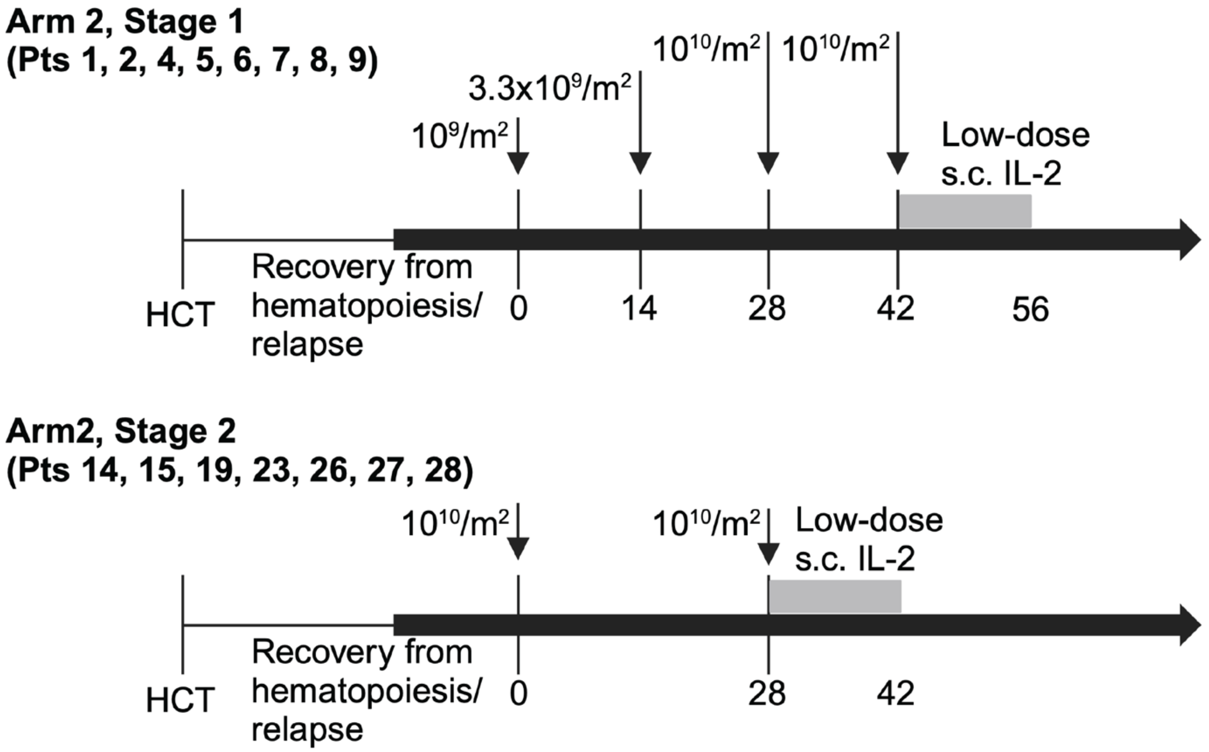

Supplementary Figure 3. Study schema. The first 8 patients who received $T_{TCR\text{-}C4}$ post-HCT relapse (Treatment Arm, Stage1) received 4 escalating infusions of 10^9-10^{10}/m^2 14 days apart, the last infusion followed by s.c. low-dose IL2 for 14 days. After the safety of the highest dose was established, all subsequent patients received 2 infusions of 10^{10}/m^2 $T_{TCR\text{-}C4}$ 28 days apart, the second infusion followed by s.c. low-dose IL2 for 14 days**.**

Supplementary Figure 4

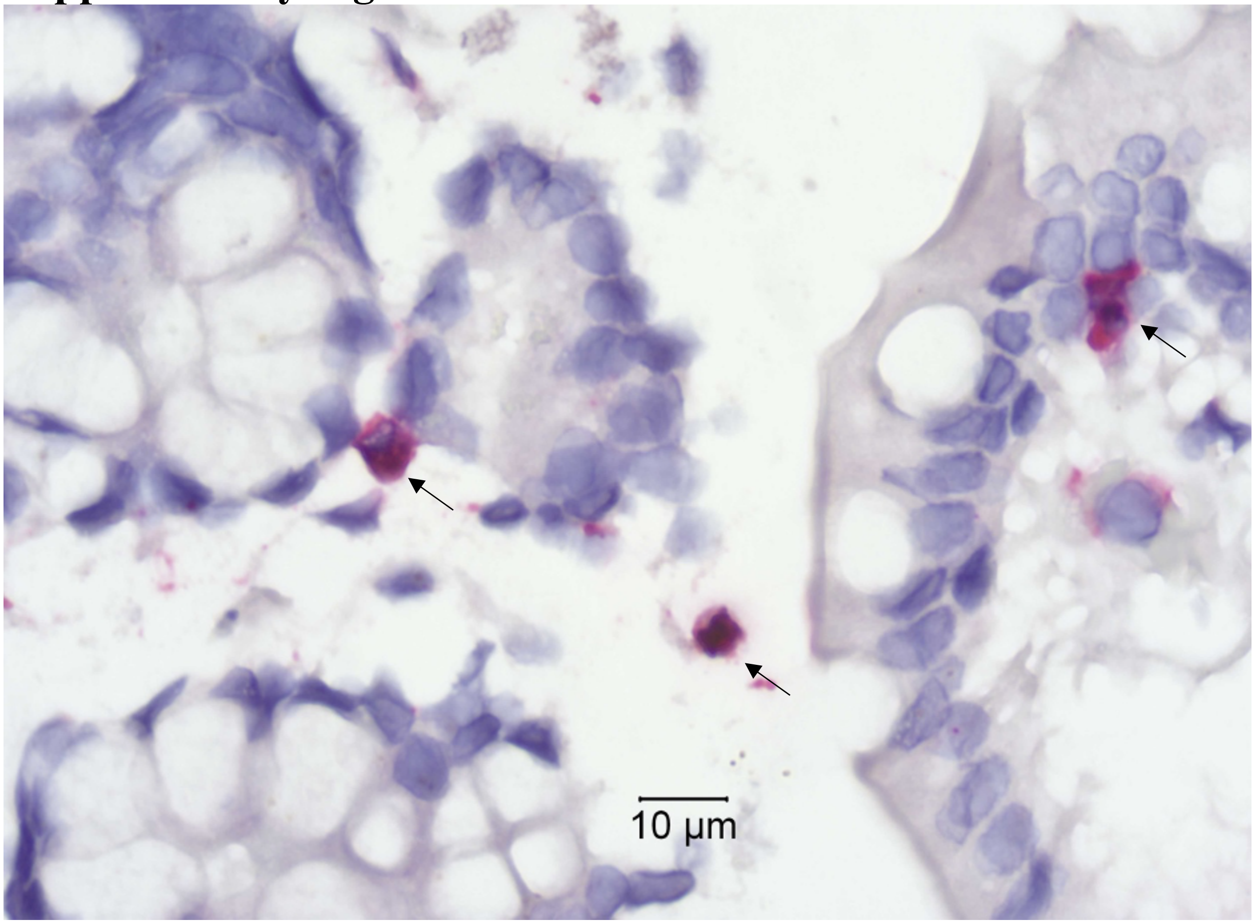

Supplementary Figure 4. WPRE staining of gastrointestinal biopsies in cGVHD. Representative high-magnification section of gastrointestinal biopsies from patient 8, taken on day +120 post-$T_{TCR\text{-}C4}$ infusion at the onset of clinical moderate cGVHD. Black arrows indicate $WPRE^{+}$ cells, marking $T_{TCR\text{-}C4}$ cells, which constitute ~0.08% of total $CD3^{+}$ T cells in the entire biopsy.

Supplementary Figure 5

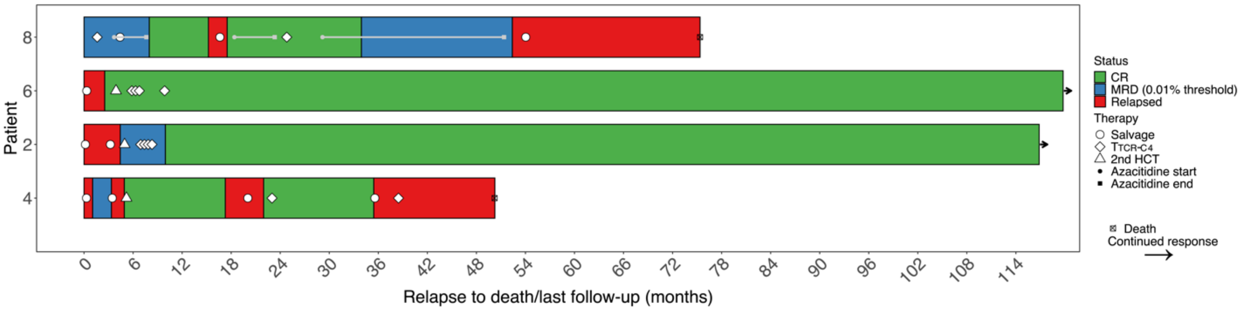

Supplementary Figure 5. Swimmer Plot of Patient Responses.
The swimmer plot illustrates the response duration for four patients enrolled in the trial. Time 0 represents the relapse or persistent disease after the first HCT that qualified patients for trial inclusion. The end of each bar indicates the date of death or last follow-up, with arrows marking ongoing responses. "Salvage" denotes any salvage strategy, including chemotherapy, radiotherapy, or intrathecal treatment. Salvage with a second HCT is marked by a triangle.

Patient 8: Relapsed patient who received $T_{TCR\text{-}C4}$ infusion while in MRD and later underwent azacitidine salvage treatment for persistent MRD (duration shown as a gray line). The patient relapsed, underwent additional salvage chemotherapy and azacitidine, and received a second $T_{TCR\text{-}C4}$ infusion. Following this, the patient experienced prolonged stable disease (MRD+) with detectable long-term persisting PB $T_{TCR\text{-}C4}$. Eventually, the disease progressed, and the patient relapsed and died (see Results for more details).

Patient 6: At relapse, the patient received salvage chemotherapy followed by a second HCT in remission. Subsequently, the patient received four $T_{TCR\text{-}C4}$ infusions. The patient was alive at the last follow-up.

Patient 2: Following relapse this patient underwent salvage treatment and a second HCT while in overt disease and remained MRD-positive 28 days post-HCT. This result was consolidated with four $T_{TCR\text{-}C4}$ infusions, achieving MRD negativity. The patient was alive at the last follow-up.

Patient 4: After relapse, the patient achieved complete remission following a second HCT. However, after ~15 months, the patient experienced extramedullary relapse, treated with azacitidine and radiotherapy, achieving complete remission. The patient maintained remission for ~1 year after the first $T_{TCR\text{-}C4}$ infusion. Subsequently, the patient relapsed extramedullary and was refractory to salvage treatment and $T_{TCR\text{-}C4}$. Further details on this case and mechanisms of AML escape have been described previously.[2]

Supplementary Figure 6

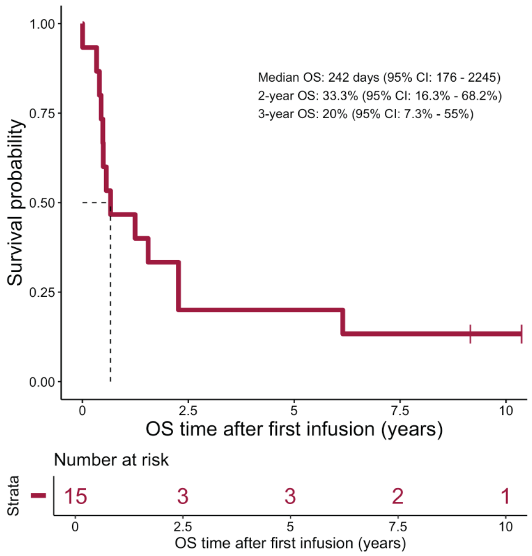

Supplementary Figure 6. Overall survival of AML patients treated with T_{TCRC4} T cells. Kaplan Meier estimate of overall survival (OS) of 15 AML patients with evidence of disease post-HCT, who were subsequently treated with T_{TCRC4} T cells

Supplementary Figure 7

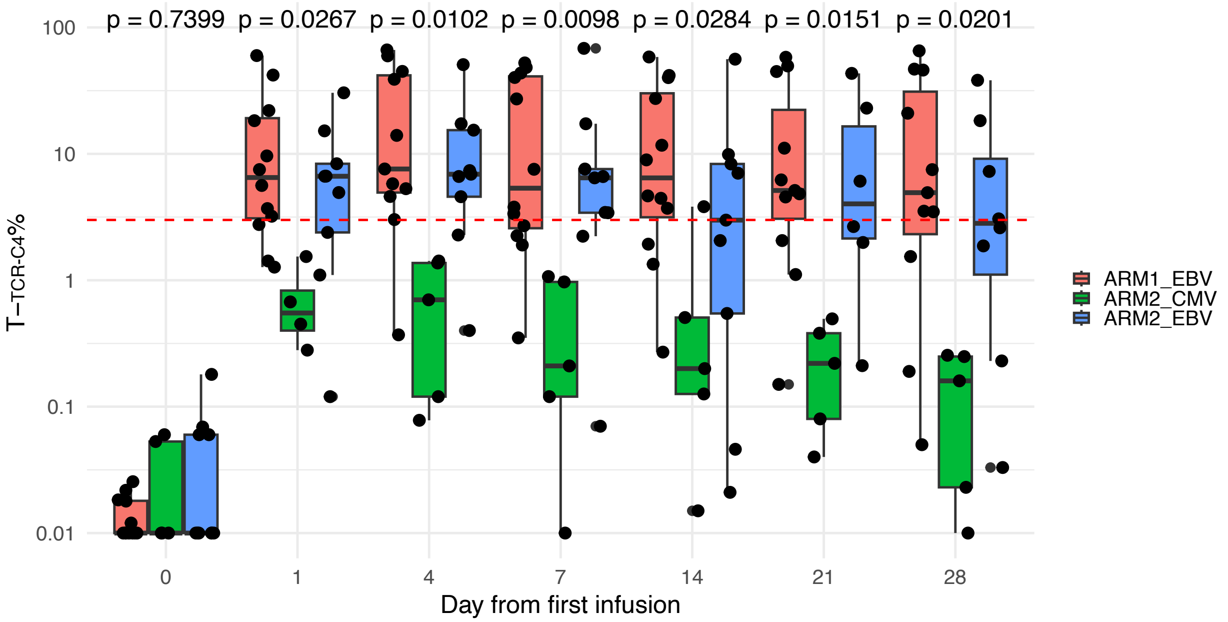

Supplementary Figure 7. EBV specific vs. CMV specific T_{TCR-C4} persistence. Boxplots comparing the percentage of EBV-specific T_{TCR-C4} cells in Arm 1 (red) and Arm 2 (blue) with the percentage of CMV-specific T_{TCR-C4} cells in Arm 2 (green) across post-infusion timepoints (n=27, 12 from Arm1 and 15 from Arm2). The y-axis indicates the percentage (log scale) of T_{TCR-C4} cells, while the x-axis represents the time post-infusion. Data were derived from flow-cytometry analysis. The horizontal dashed red line indicates the 3% threshold used to define persisting T_{TCR-C4} cells. The y-axis is displayed on a log10 scale. Statistical comparison between all three groups at each time point was performed using a Kruskal-Wallis test, with p-values displayed above the corresponding boxplots. Boxplots show the interquartile range (IQR); lines denote medians. The bounds of the box represent the 25th and 75th percentiles; whiskers span 1.5x IQR

Supplementary Figure 8

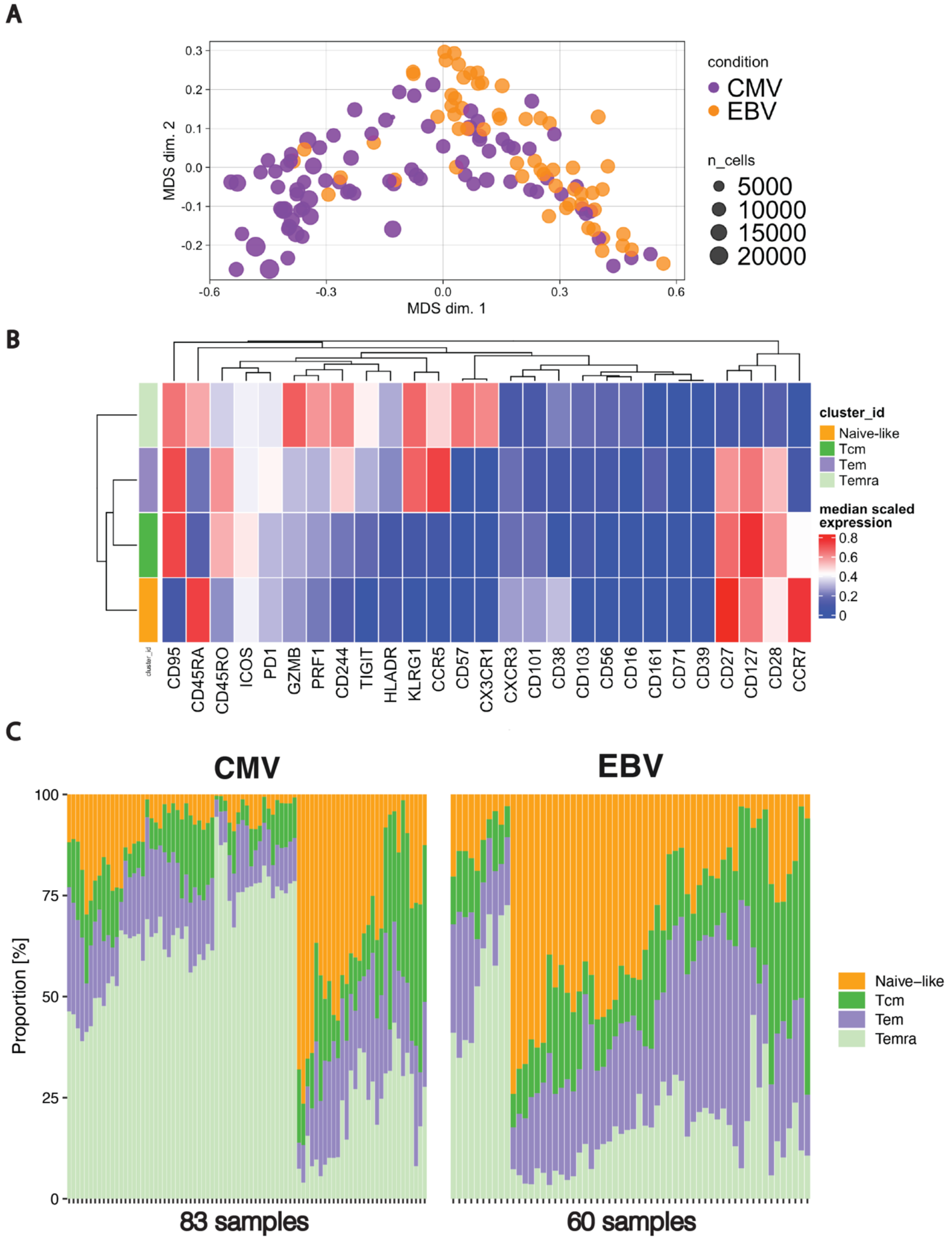

Supplementary Figure 8. EBV and CMV specific CD8^{+} T-cell states. (A) Multidimensional (MDS) scaling plot illustrates the distribution of samples based on the expression of top varying markers, which are those markers showing the most variation in expression levels across the dataset. These markers are selected for their ability to highlight meaningful differences between samples or conditions. In this context, samples associated with CMV (violet dots) and EBV (orange dots) are plotted along the first two MDS dimensions (MDS dim. 1, MDS dim. 2). This unsupervised method visualizes sample clustering based on marker expression, with dissimilarities calculated using the median expression of markers across all cells in each sample. The plot provides an overview of variance and potential clustering patterns among the samples. **(B)** Heatmap illustrating the median expression levels of 27 markers across four PB CD8^{+} T-cell subsets: Naïve-like (CD95-, CD45RA+, CCR7+, CD27+, CD28+), Effector Memory (Tem) (CD95+, CD45RA-, CD45RO+, CD27+, CD28+, CD127+), Central Memory (Tcm) (CD95+, CD45RA-, CD45RO+, CCR7+, CD27+, CD28+, CD127+), and Effector Memory RA+ (Temra) (CD95+, CD45RA+, CD45RO-, CD57+, KLRG1+). The data are scaled to normalize marker expression across the dataset before clustering, ensuring each marker contributes proportionally to cluster definitions while minimizing the impact of extreme values or outliers. Blue represents lower expression, and red indicates higher expression. Hierarchical clustering on the top dendrogram groups markers based on their expression patterns, revealing co-occurring markers across subsets, while the clustering on the left shows relationships between subsets based on their marker profiles, highlighting their similarities. **(C)** Stacked bar plots depicting the distribution of annotated T-cell states across individual samples (x-axis) (n =143), stratified by virus specificity (CMV on the left, EBV on the right). Each color represents a distinct T-cell state

Supplementary Figure 9

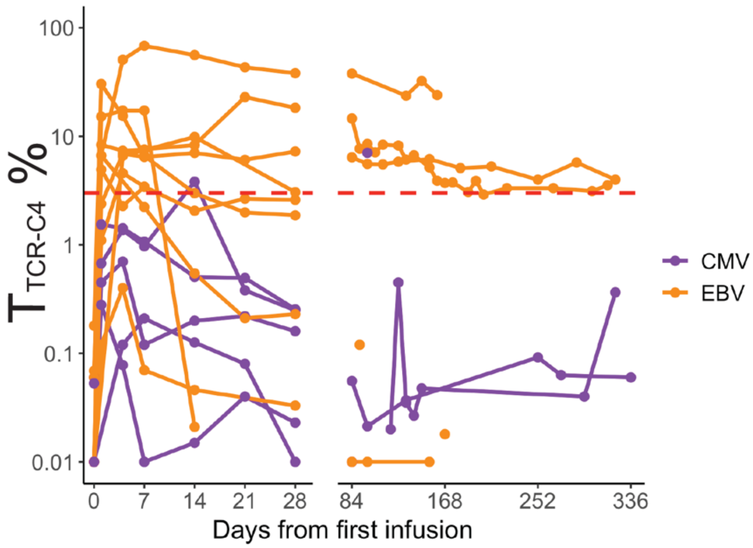

Supplementary Figure 9. Persistence of T_{TCR-C4} post-first infusion Line plot showing the percentage (log scale) of T_{TCR-C4} in PBMCs collected after the first infusion across all patients (n =15), colored by virus-specificity (violet = CMV; orange = EBV), derived from flow-cytometry data

Supplementary Figure 10

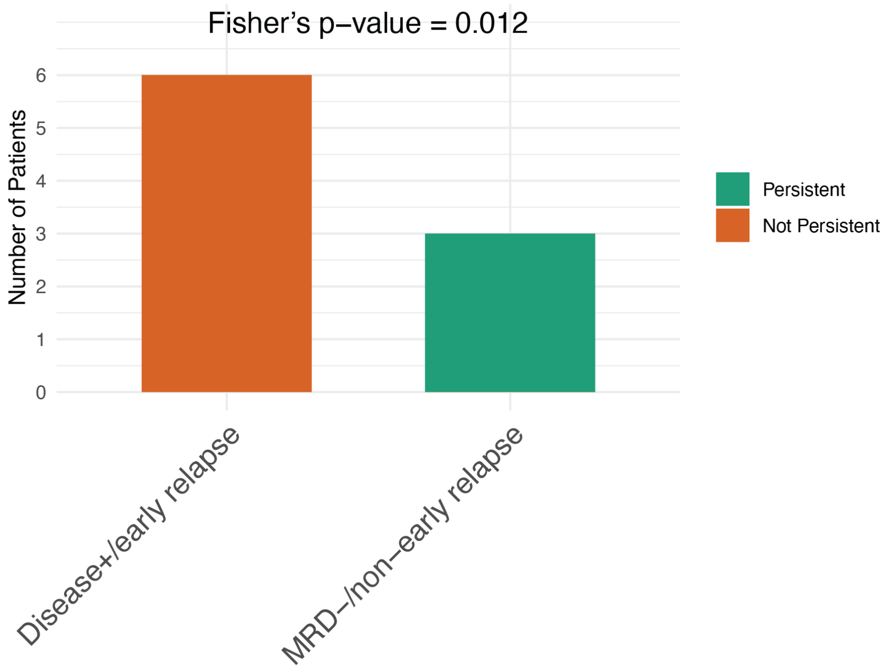

Supplementary Figure 10. $T_{TCR\text{-}C4}$ persistence in relation to clinical risk factors. Bar plot showing the distribution of $T_{TCR\text{-}C4}$ persistence (green) and non-persistence (red) based on clinically-defined risk factors: detectable disease pre-infusion (Disease+)/early relapse post-HCT vs. MRD-/absence of early relapse (n =9). Two-sided Fisher's exact test was used for statistical assessment, with p-values indicating significant association ($p < 0.05$).

Supplementary Figure 11

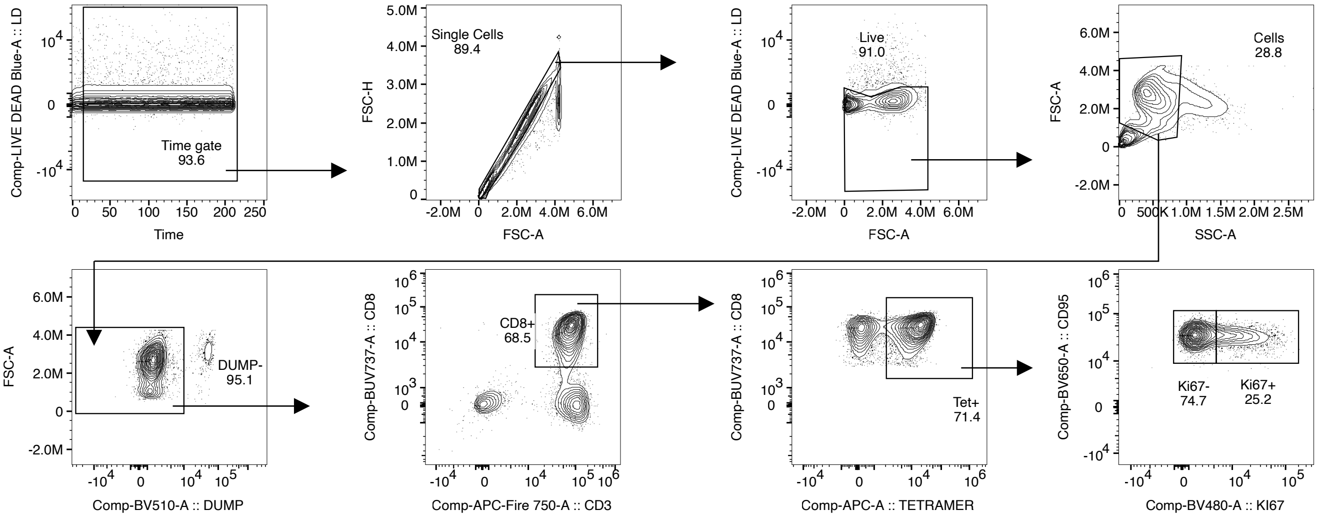

Supplementary Figure 11. Spectral flow-cytometry gating strategy to analyze Ki67⁺ and Ki67⁻ $T_{TCR\text{-}C4}$. Representative gating strategy used to distinguish Ki67⁺ and Ki67⁻ TTCR-C4 cells (tetramer-positive) at timepoints T1, T2, T3, and T4 following the first infusion. Myeloid lineage cells were excluded using a dump channel containing antibodies against CD33 and CD14 (see Supplementary Table 4 for full panel details).

Supplementary Figure 12

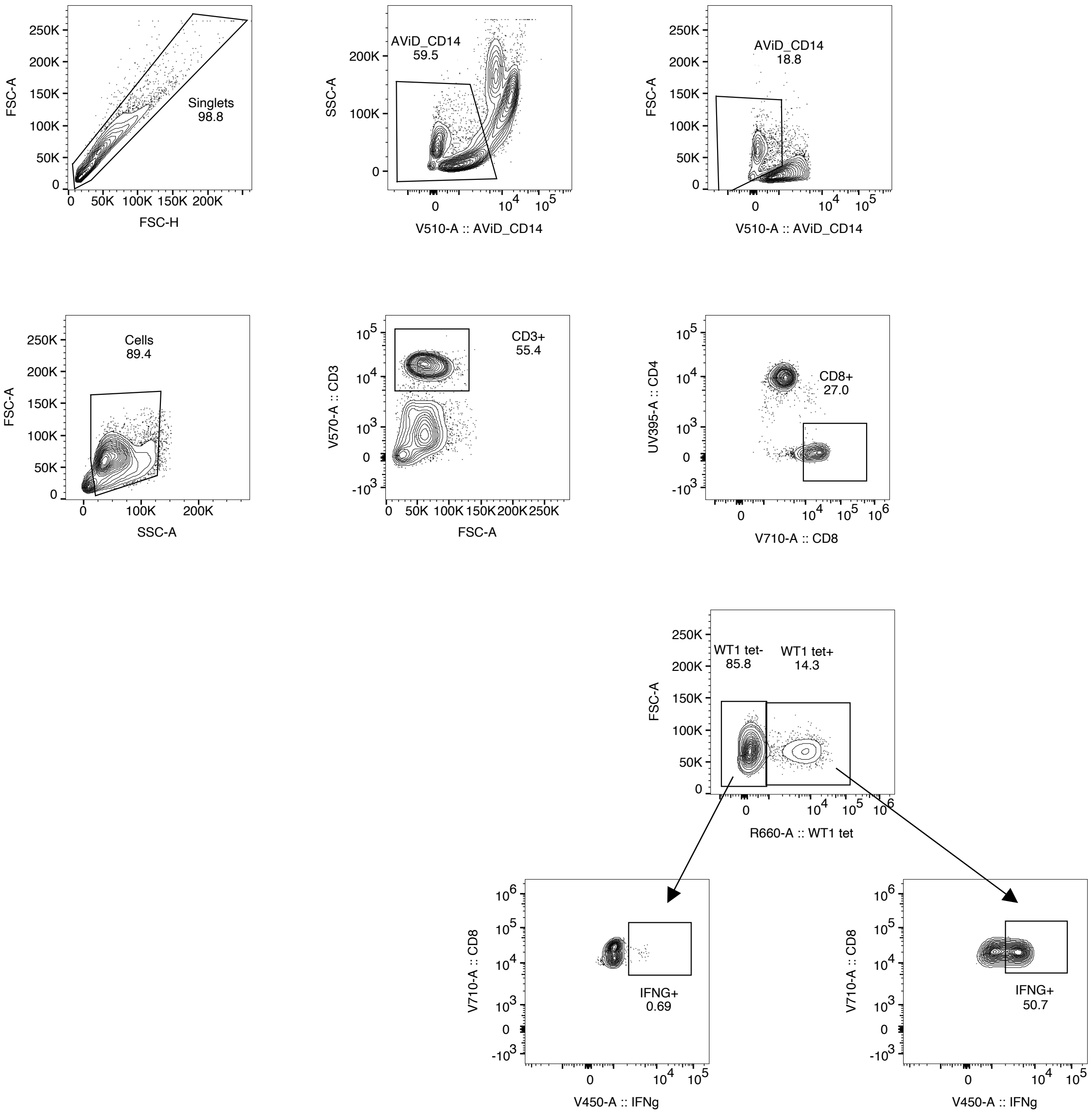

Supplementary Figure 12. Gating strategy for IFNγ producing Tet$^+$ $T_{TCR\text{-}C4}$ cells. Gating strategy used to calculate the tetramer-positive (Tet$^+$) and tetramer-negative (Tet$^-$) IFNγ-producing cells within the total CD8$^+$ T cell population. AVID_CD14 is a dump channel for dead and CD14$^+$ cells. The percentage of the IFNγ-producing cells was calculated relative to the total CD8$^+$ T cells for both the Tet$^+$ and Tet$^-$ subsets (7.27% and 0.59%, respectively)

Supplementary Figure 13

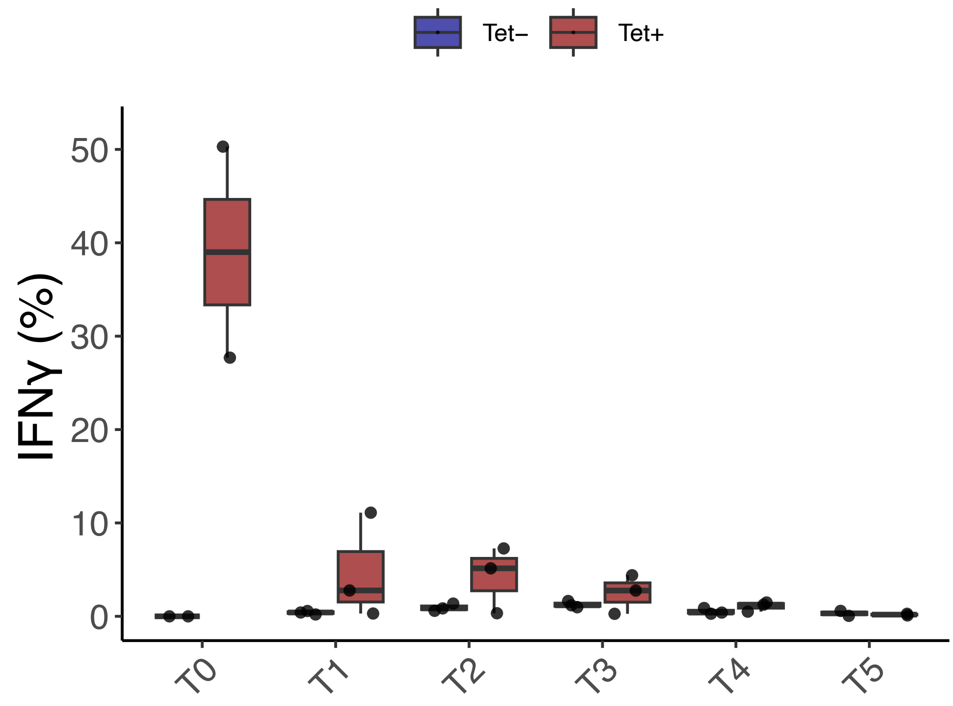

Supplementary Figure 13. Analysis of IFNγ production by Tet⁺ (T_{TCR-C4}) and Tet⁻ CD8⁺ T cells Boxplots showing the percentage of Tet^+ T_{TCR-C4} and Tet^- IFNγ-producing cells (IFNγ%), at different timepoints (T0 to T5), following WT1-peptide stimulation, within the total $CD8^+$ population (n = 3 total number of patients). The x-axis represents the timepoints (days) from the first infusion (**Supplementary Table 6**). The y-axis represents the percentage of IFNγ-producing cells. Tet^+ cells are shown in dark red, and Tet^- cells are shown in dark blue. Boxplots show the interquartile range (IQR); lines denote medians. The bounds of the box represent the 25th and 75th percentiles; whiskers span 1.5x IQR. The data points represent individual observations, jittered for better visibility.

Supplementary Figure 14

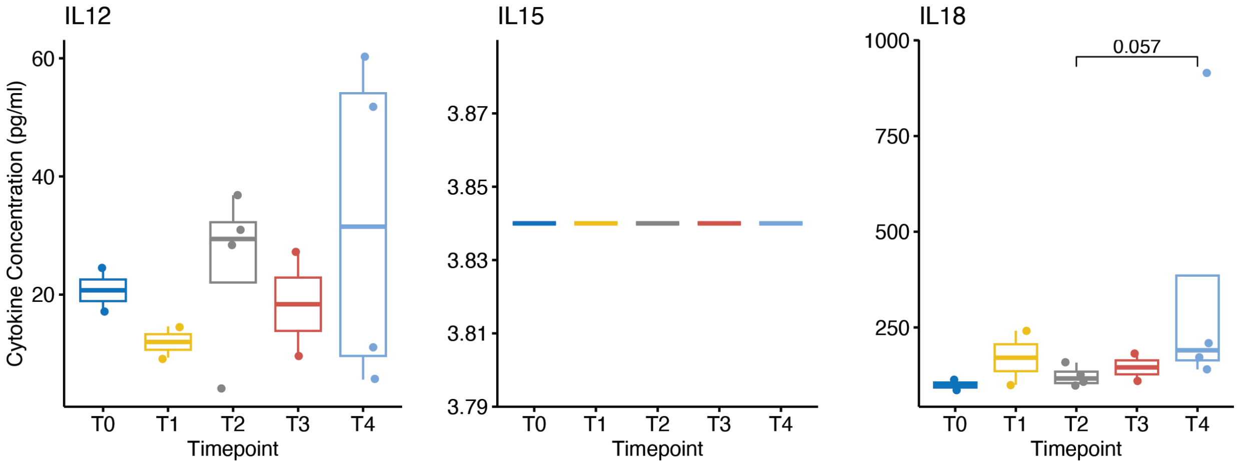

Supplementary Figure 14. Serum cytokines levels before and after T_{TCR-C4} infusion. The boxplots show the concentration (pg/ml) of IL12, IL15, IL18 at different timepoints post-infusion (n = 4 total number of patients). Statistical testing was performed using two-sided Wilcoxon rank sum test with Bonferroni correction. IL-15 levels were below the limit of detection of the assay at all the timepoints. Boxplots show the interquartile range (IQR); lines denote medians. The bounds of the box represent the 25th and 75th percentiles; whiskers span 1.5x IQR

Supplementary Figure 15

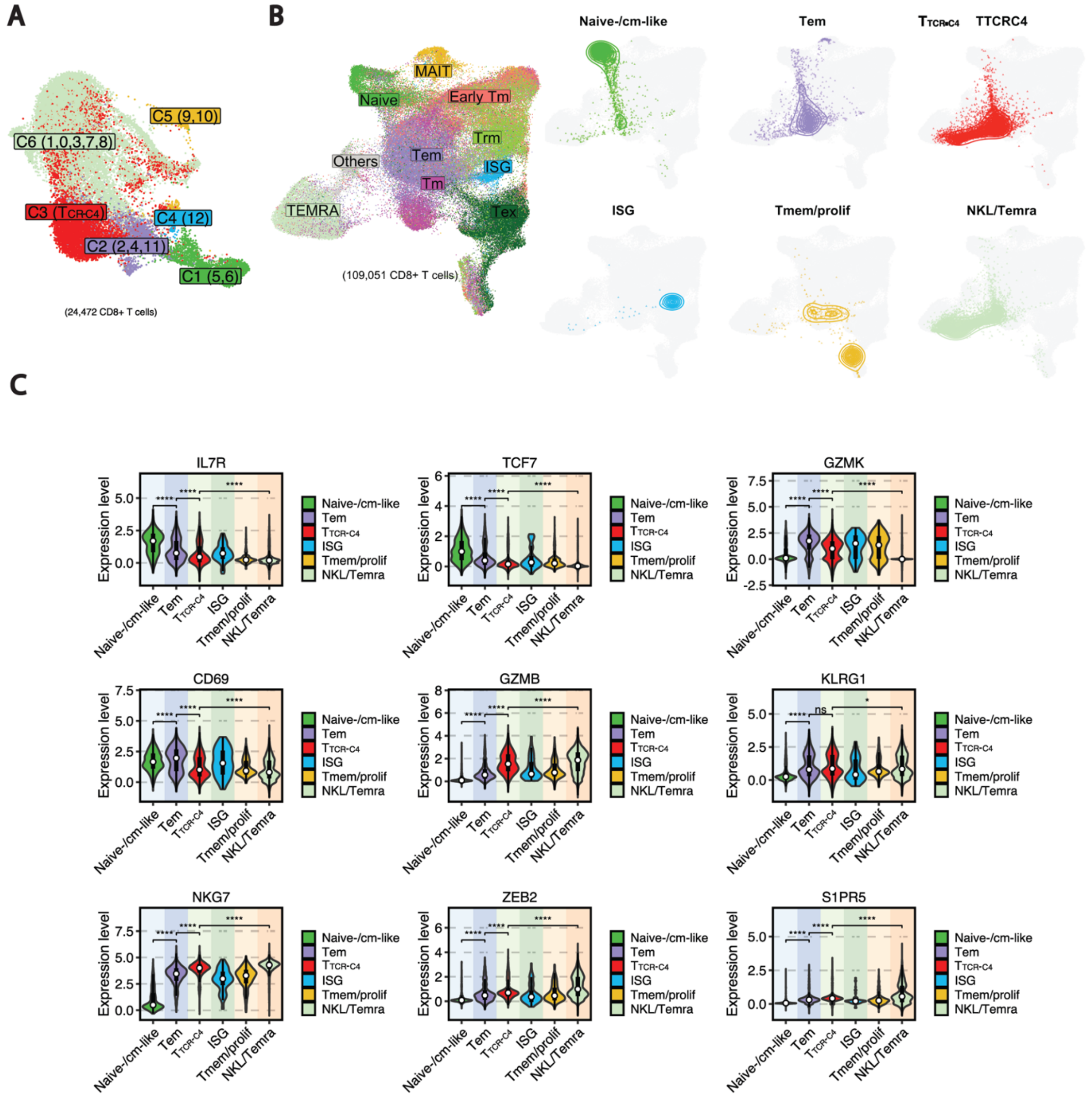

Supplementary Figure 15. **Identification of transcriptional endogenous and $T_{TCR\text{-}C4}$ $CD8^+$ T-cell states**. **(A)** UMAP plot showing the two-dimensional distribution of K-means cluster groups, identified by numbers (1 to 5) to the left of the dendrogram in the heatmap in Fig. 3A. Clusters within each group are indicated in brackets. C3 ($T_{TCR\text{-}C4}$) was identified using scGate[3] based on TCR_{C4} expression. **(B)** UMAP plot showing the distribution of $CD8^+$ T-cell states from a published independent dataset[4] used as a reference atlas (left). The UMAP projections of each T-cell state defined in Fig. 3C (query) are overlaid onto the reference atlas as contour plots. Contours corresponding to each subset from the query are color-coded as in Fig. 3C, allowing for easy visualization of the distribution of each subset within the reference atlas. **(C)** Violin plots displaying the expression levels (y-axis) of stem-like (*IL7R*, *TCF7*), activation (*GZMK*, *CD69*), and cytotoxicity/NK-like (*GZMB*, *KLRG1*, *NKG7*, *ZEB2*, *S1PR5*) markers across $CD8^+$ T-cell transcriptional states (x-axis). Statistical significance was assessed using a two-sided Wilcoxon rank sum test using the function FeatureStatPlot from the SCP framework (https://github.com/zhanghao-njmu/SCP). Asterisks indicate the following thresholds of significance: *$p < 0.05$, **$p < 0.01$, ***$p < 0.001$, ****$p < 0.0001$. Exact p-values: *IL7R*, *TCF7*, *GZMK*, *GZMB*, *NKG7*, *ZEB2*, and *S1PR5* all showed $p < 2.22e\text{-}16$ for significant comparisons. For *CD69*, $p = 2.7e\text{-}06$ (Naïve-/cm-like vs. Tem), and $p < 2.22e\text{-}16$ for other significant comparisons. *KLRG1* comparisons yielded $p < 2.22e\text{-}16$ (Naïve-/cm-like vs. Tem), $p = 0.079$ (Tem vs. $T_{TCR\text{-}C4}$), and $p = 0.014$ ($T_{TCR\text{-}C4}$ vs. NKL/Temra).

Supplementary Figure 16

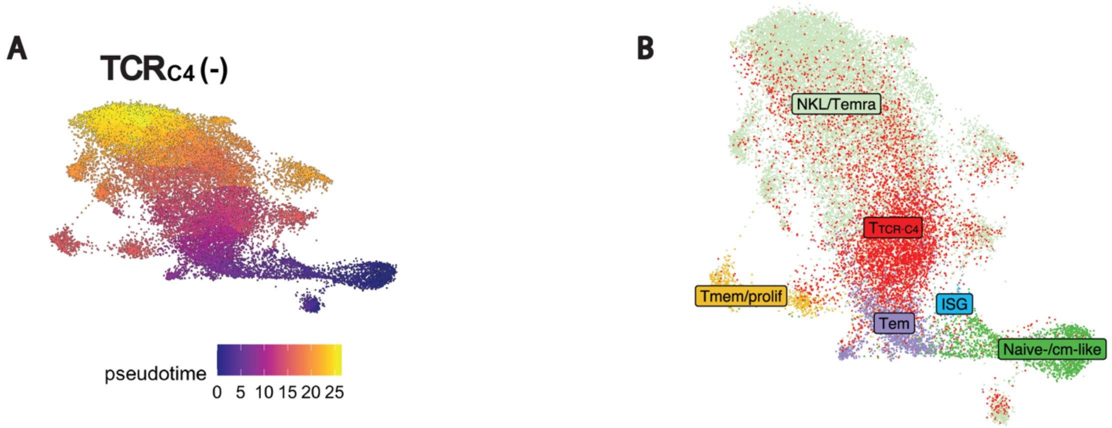

Supplementary Figure 16. CD8^{+} transcriptional states after TCR$_{C4}$ removal. (A) UMAP plot depicting the inferred developmental trajectory of CD8^{+} T-cell transcriptional states, as predicted by Monocle, following the removal of the TCR$_{C4}$ transcript. The color scale, ranging from blu to orange/yellow, represents the pseudotime, where blue indicates earlier developmental stages and orange/yellow indicates later stages of the trajectory. **(B)** UMAP plot showing the distribution of CD8^{+} T-cell transcriptional states after TCR$_{C4}$ removal, with the same coordinates as panel A. The T-cell subsets are labeled according to the clustering defined in Fig. 3C, providing context for how the subsets are positioned within the developmental trajectory in A.

Supplementary Figure 17

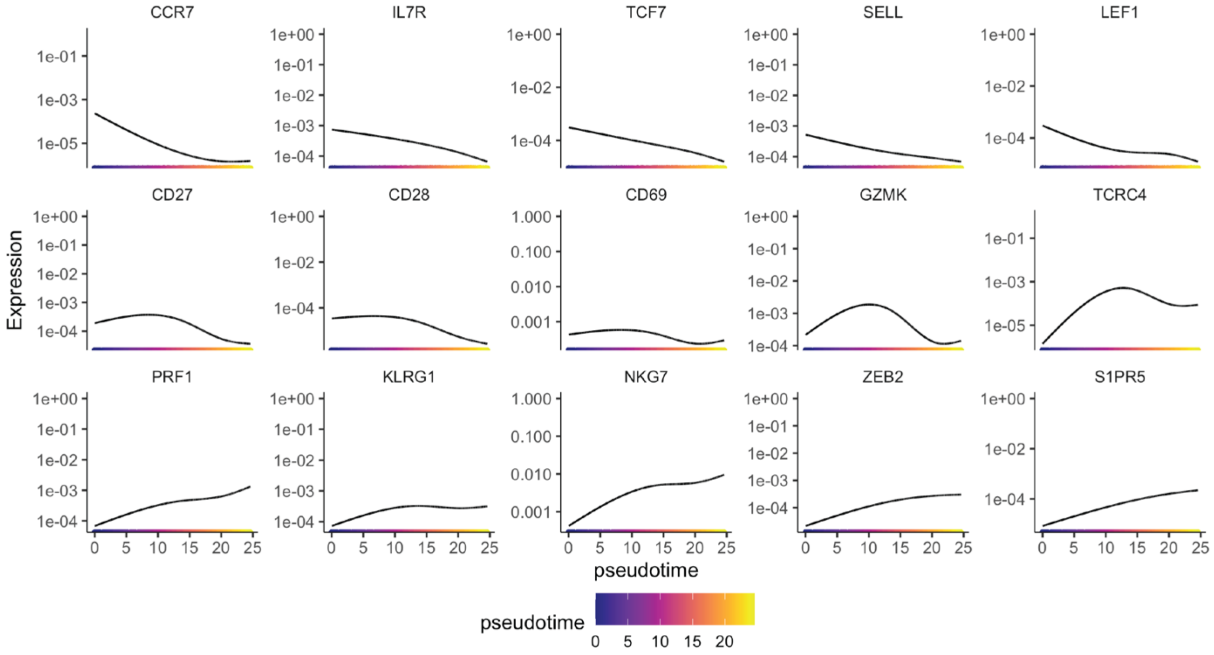

Supplementary Figure 17. Smoothed Gene Expression Dynamics Over Pseudotime. Line plots illustrating the smoothed expression patterns of key genes defining distinct T-cell states over pseudotime, highlighting transcriptional changes associated with T-cell differentiation.

Supplementary Figure 18

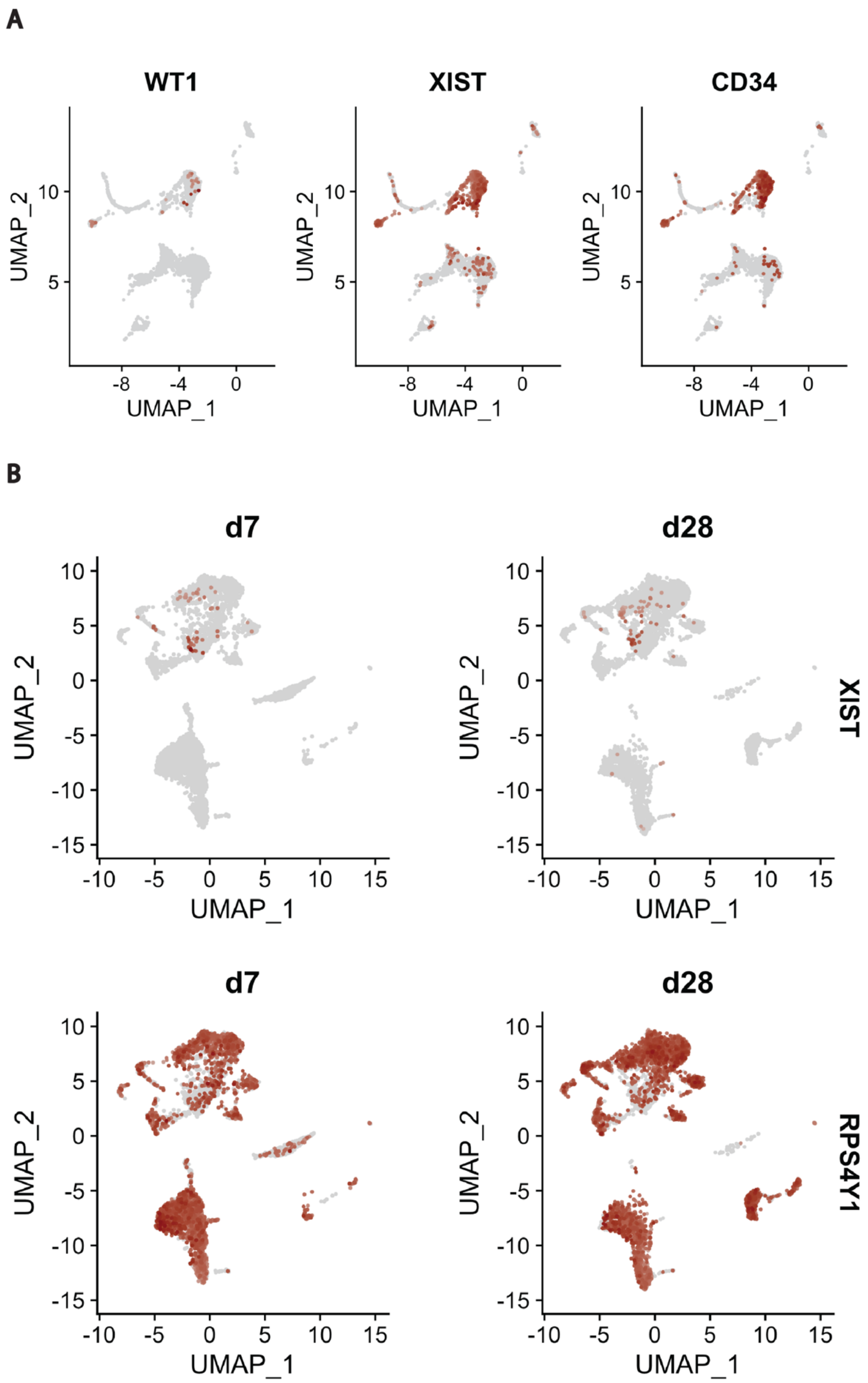

Supplementary Figure 18. UMAP visualization of gene expression in AML patient samples. (A) UMAP plots at day 49 post-first T_{TCR-C4} infusion depict the expression patterns of leukemia-associated markers *WT1* and *XIST* (a female-specific gene) in patient 8, previously transplanted with a sex-mismatched donor (female patient, male donor). Additionally, *CD34* expression across all cell populations is shown. Cells with high expression of each marker are highlighted in red, while other cells appear in grey.
(B) UMAP plots illustrate a longitudinal analysis of leukemia-associated gene expression in patient 26, who was transplanted with a sex-mismatched donor (female patient, male donor), at days 7 (d7) and 28 (d28) post-first T_{TCR-C4} infusion. *XIST* indicates leukemic cells, while *RPS4Y1* (a male-specific gene) marks non-leukemic cells. Cells with high gene expression are represented in dark red, and those with lower expression appear in light grey.

Supplementary Figure 19

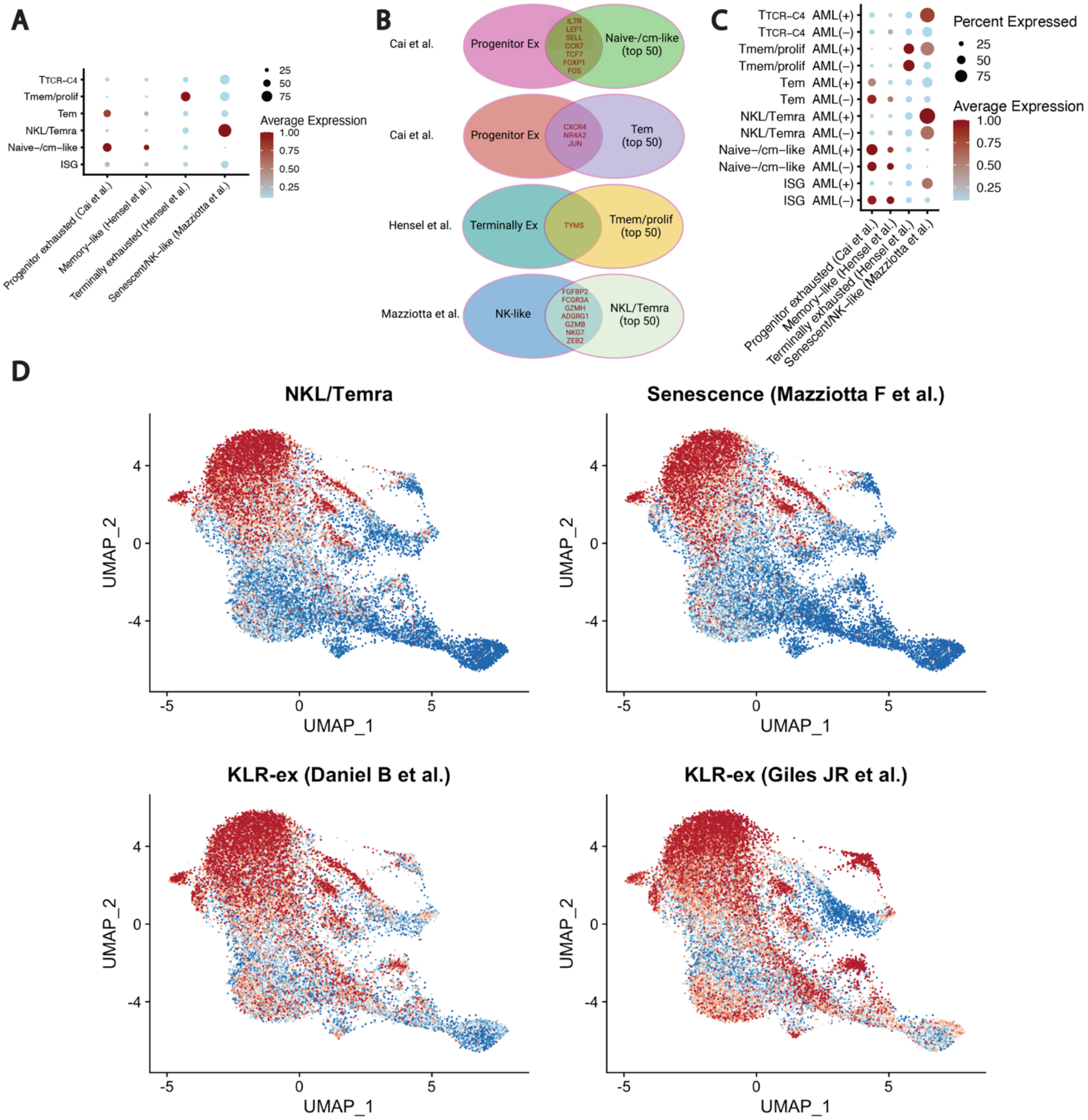

Supplementary Figure 19. T-cell exhaustion and KLR signatures in endogenous and T_{TCR-C4} $CD8^+$ T cells. (A) Dot plot showing the expression of gene signatures of exhaustion[5,6] and terminal differentiation/NK-like[7] (x-axis) across the annotated endogenous and T_{TCR-C4} T-cell states (y-axis). **(B)** Venn diagrams illustrating the overlap between progenitor exhausted, terminally exhausted and NK-like signatures and the top 50 differentially expressed genes in Naïve-/cm-like, ISG and NKL/Temra subsets, respectively. Representative overlapping genes are shown in the figure. The full list of overlapping genes is provided in Supplementary Table 11. **(C)** Dot plot showing the expression of gene signatures of exhaustion and terminal differentiation/NK-like (x-axis) across the annotated endogenous and T_{TCR-C4} T-cell states in AML(-) and AML(+) samples (y-axis). **(D)** UMAP plots illustrate the spatial distribution of $CD8^+$ T cells based on their expression of published KLR gene signatures.[7-9] These signatures, derived from prior studies, were applied to our scRNAseq dataset to calculate a score for each cell, reflecting the degree of expression of the KLR features. The scores are visualized using a gradient, where blue indicates low expression and red indicates high expression. Regions with high expression (red) align with the subset we identified as Temra NK-like cells, suggesting a phenotypic overlap within this subset.

Supplementary Figure 20

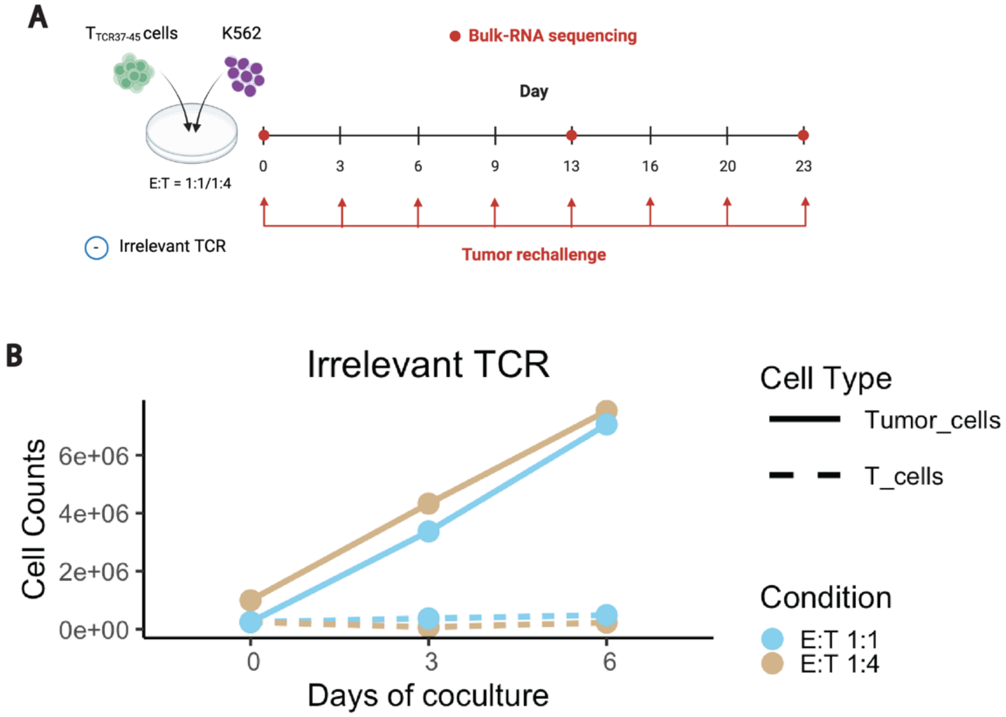

Supplementary Figure 20. In-vitro dysfunction coculture system. (A) Experimental design of the *in vitro* model used to study chronic tumor exposure of WT1-specific T cells ($T_{TCR37-45}$ cells). The diagram shows two conditions with Effector ($T_{TCR37-45}$) to Target (K562) (E:T) ratios of 1:1 and 1:4. Red arrows indicate tumor re-challenge every 3-4 days, while red circles on the timeline mark the time points when RNA extraction for bulk-RNA sequencing was performed. An irrelevant TCR-expressing T cell line was used as a negative control. The experiment involved exposing transgenic $CD8^{+}$ WT1-specific T cells to HLA-A*0201-transduced K562 cells expressing high levels of WT1. Created in BioRender. Mazziotta F. (2025) https://BioRender.com/zqb8xqw. **(B)** Line plots showing the changes in absolute cell counts (y-axis) over time (x-axis, days of coculture) for T cells with irrelevant TCR (dashed lines) and K562 tumor cells (solid lines) in coculture at E:T ratios of 1:1 and 1:4.

Supplementary Figure 21

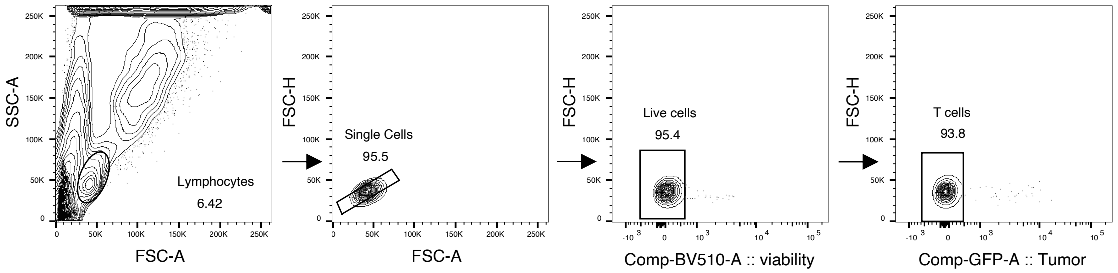

Supplementary Figure 21. Sorting strategy for transcriptional profiling of $T_{TCR37\text{-}45}$ cells following repeated antigen stimulation. Lymphocytes were first gated based on FSC/SSC properties, followed by selection of single cells (FSC-A vs. FSC-H) and live cells (viability vs. FSC-H). Tumor cells were excluded by gating out GFP^+ events (K562 cells), and TCR-transduced T cells were identified and sorted as GFP^- cells for bulk RNA sequencing.

Supplementary Figure 22

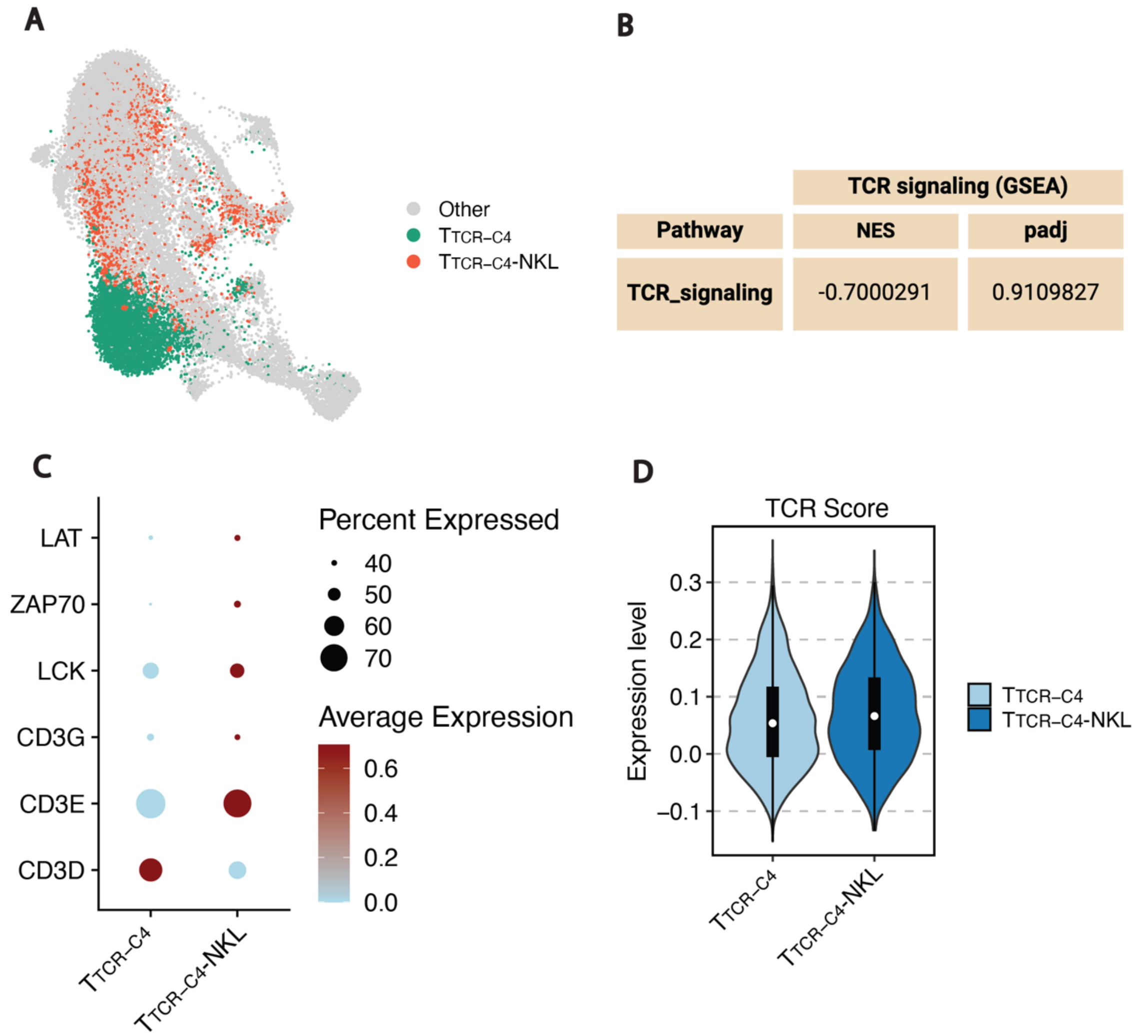

Supplementary Figure 22. Comparison of TCR signaling in $T_{TCR\text{-}C4}$ and NKL-skewed $T_{TCR\text{-}C4}$ cells. (A) UMAP plot depicting the coordinates of NKL-skewed $T_{TCR\text{-}C4}$ ($T_{TCR\text{-}C4}$-NKL, red) and non NKL-skewed $T_{TCR\text{-}C4}$ ($T_{TCR\text{-}C4}$, green). **(B)** Table showing the results of GSEA performed on DGE between $T_{TCR\text{-}C4}$-NKL and $T_{TCR\text{-}C4}$. DEGs were identified using a two-sided Wilcoxon rank-sum test with Bonferroni correction. No significant differences (padj > 0.05) were observed in TCR signaling pathway enrichment (KEGG) between the two $T_{TCR\text{-}C4}$ cell states. **(C)** Dot plot showing the expression of TCR signaling genes (y-axis) in $T_{TCR\text{-}C4}$ and $T_{TCR\text{-}C4}$-NKL. Expression levels are represented by color intensity, with darker red indicating higher expression. **(D)** Violin plot comparing the TCR signaling score derived from KEGG TCR signaling pathway genes between $T_{TCR\text{-}C4}$ and $T_{TCR\text{-}C4}$-NKL. The plot highlights the expression of TCR signaling score between the two groups, with boxplots superimposed for a detailed view of the central tendency and variability.

Supplementary Figure 23

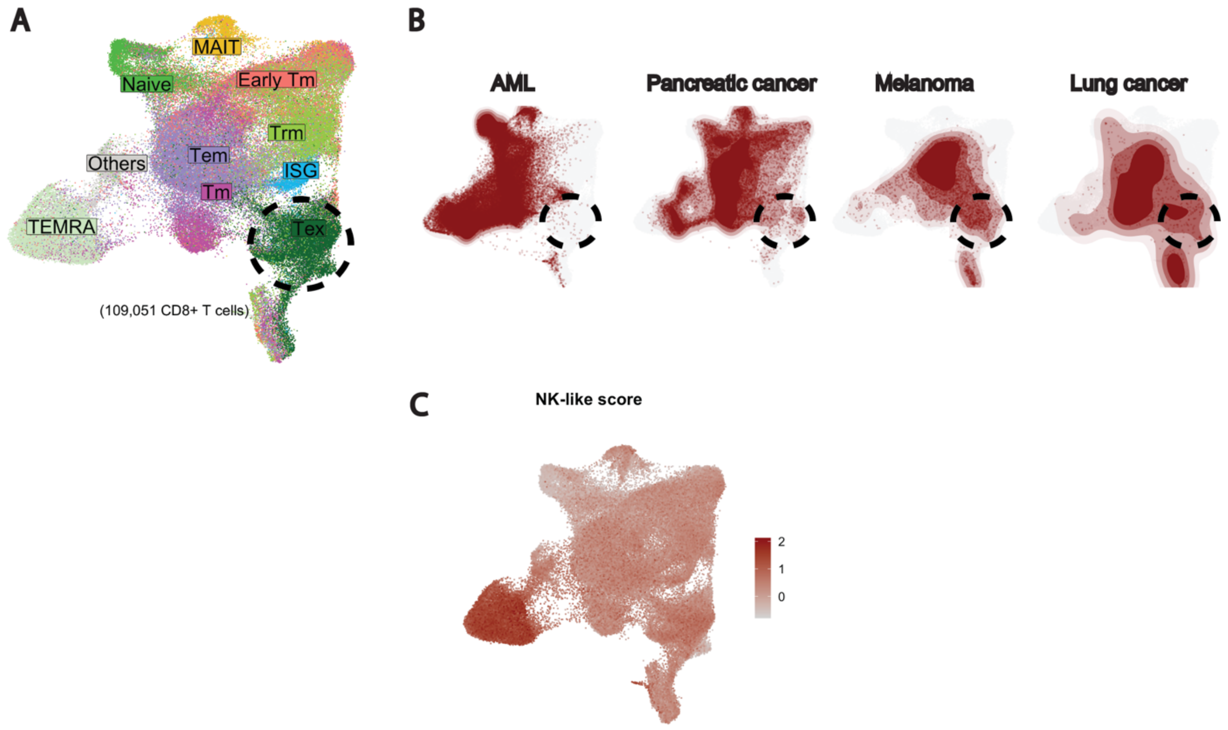

Supplementary Figure 23. UMAP projections of published AML, solid tumor datasets, and NK-like module scores based on gene expression in a tumor-infiltrating lymphocyte reference atlas.[4] (A) UMAP plot showing the distribution of $CD8^{+}$ T-cell states from a published independent dataset used as reference atlas.[4] Dashed line indicates the UMAP coordinates of Tex $CD8^{+}$ T-cell subset. **(B)** UMAP plot of $CD8^{+}$ T-cell reference atlas (light grey) overlaid with projections (dark red) of $CD8^{+}$ T cells collected from various independent published scRNAseq datasets (queries) of AML[7,10-13], pancreatic cancer,[14] , melanoma, [15] and lung cancer.[16] Dashed line indicates the area corresponding to Tex. **(C)** UMAP plot projection showing the module score based on the expression of the following genes: *KLRG1*, *KLRD1*, *ZEB2*, *FCRL6*, *ADGRG1*, *S1PR5*, *FCGR3A*, *GZMB*, and *NKG7*. Each point represents an individual cell, and the color scale ranges from light grey (low module score) to dark red (high module score), reflecting the intensity of the gene set expression across cells.

Supplementary Figure 24

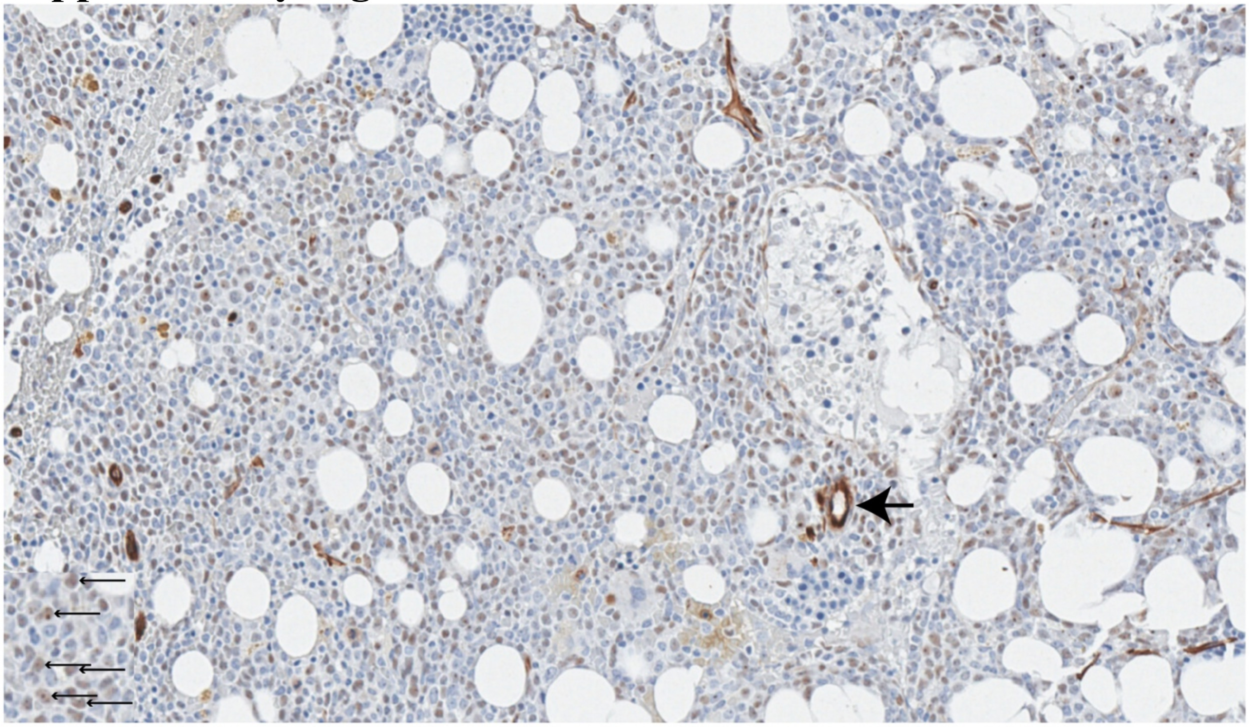

Supplementary Figure 24. Immunohistochemistry of bone marrow biopsy stained for WT1. This immunohistochemistry image shows a bone marrow biopsy stained for WT1. Black arrows indicate WT1-positive nuclei, visible as distinct dark-stained areas within the cells, which signify expression of the WT1 protein.

Supplementary Figure 25

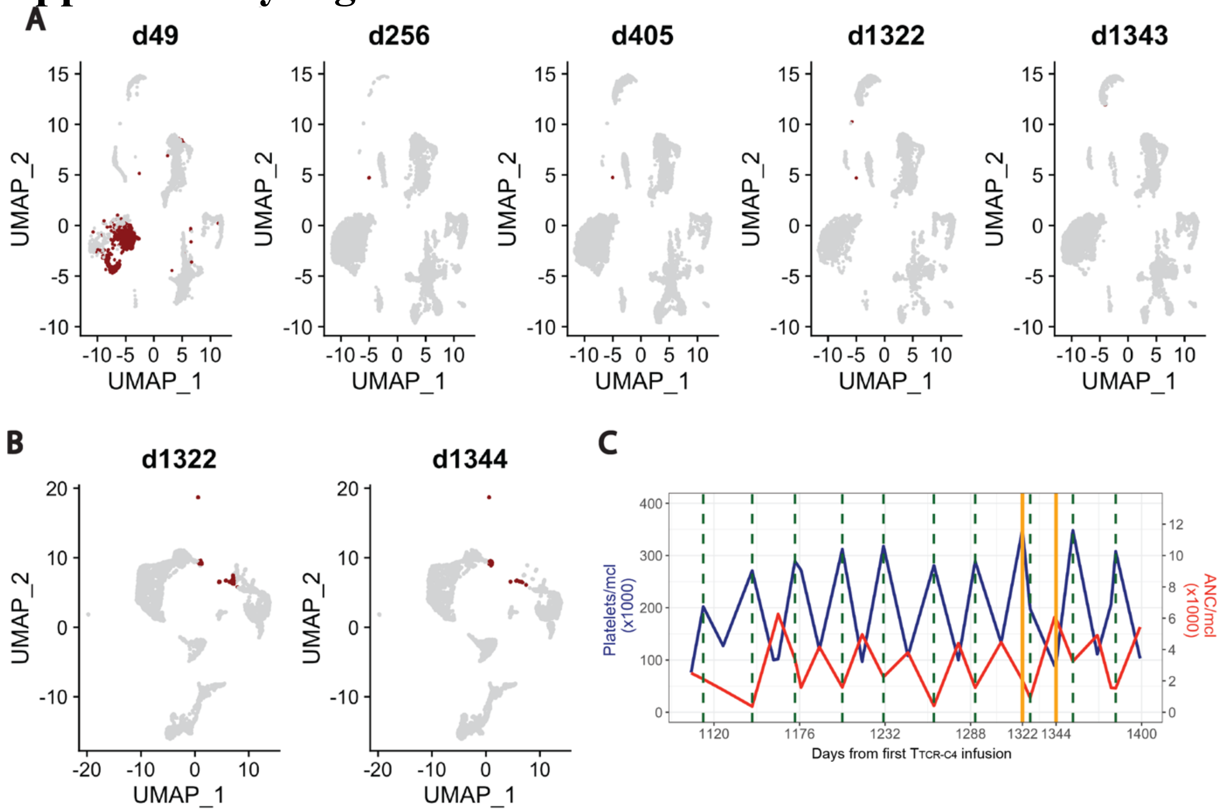

Supplementary Figure 25. Patient 8 blasts and T cells characterization (A) UMAP plot showing a blast score (dark red) calculated based on the co-expression of *CD34* and *XIST* (a female-specific gene) in a patient previously transplanted with a sex-mismatched donor (female patient, male donor). The score was used to identify cells expressing a high blast score. Each UMAP plot represents a different timepoint (day 49, 256, 405, 1322, 1343) after the first infusion, with light grey indicating low expression and dark red indicating high expression of the blast score. **(B)** UMAP plot showing a blast score calculated based on the co-expression of *XIST*, *CD34*, *CD33*, and *KIT*. This score was used to visualize cells with high blast scores in bone marrow samples. Each UMAP plot represents a specific timepoint (day 1322 and 1344) post-first $T_{TCR\text{-}C4}$ infusion, **(C)** Line plot showing platelets and neutrophil absolute counts over time after the first infusion. Platelets (left y-axis, dark blue line plot, measured as platelets/µL × 1000) and neutrophils (right y-axis, red line plot, measured as ANC/µL × 1000) are shown in relation to the days after the first infusion (x-axis). Vertical dark green dashed lines indicate the timing of azacytidine cycles, while solid orange lines represent the timepoints when BM scRNA-seq was performed.

Supplementary Figure 26

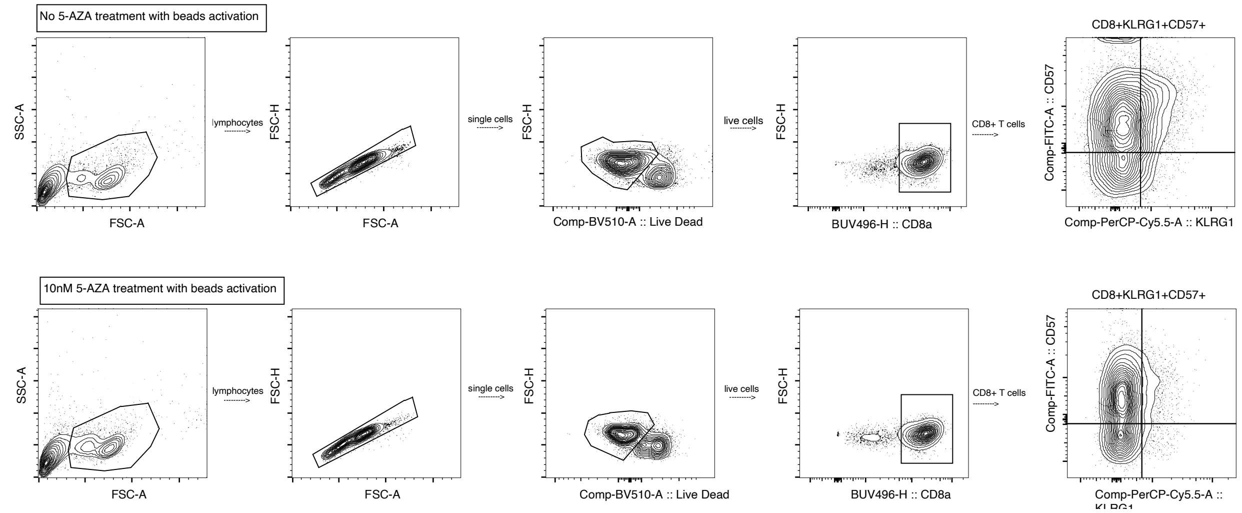

Supplementary Figure 26. Gating strategy and quantification of CD57$^+$KLRG1$^+$ CD8$^+$ T cells in the presence or absence of azacitidine. Representative flow cytometry plots showing the gating strategy to identify CD8$^+$ TCR-T cells co-expressing CD57 and KLRG1 following treatment without azacitidine (first row) or with 10nM azacitidine (10 nM 5-AZA, second row).

References

1. Chapuis, A.G., *et al.* T cell receptor gene therapy targeting WT1 prevents acute myeloid leukemia relapse post-transplant. *Nature Medicine* **25**, 1064-1072 (2019).
2. Lahman, M.C., *et al.* Targeting an alternate Wilms' tumor antigen 1 peptide bypasses immunoproteasome dependency. *Science translational medicine* **14**, eabg8070-eabg8070 (2022).
3. Andreatta, M., Berenstein, A.J. & Carmona, S.J. scGate: marker-based purification of cell types from heterogeneous single-cell RNA-seq datasets. *Bioinformatics (Oxford, England)* **38**, 2642-2644 (2022).
4. Zheng, L., *et al.* Pan-cancer single-cell landscape of tumor-infiltrating T cells. *Science (New York, N.Y.)* **374**, abe6474-abe6474 (2021).
5. Cai, C., *et al.* Identification of human progenitors of exhausted CD8(+) T cells associated with elevated IFN-gamma response in early phase of viral infection. *Nat Commun* **13**, 7543 (2022).
6. Hensel, N., *et al.* Memory-like HCV-specific CD8(+) T cells retain a molecular scar after cure of chronic HCV infection. *Nat Immunol* **22**, 229-239 (2021).
7. Mazziotta, F., *et al.* CD8+ T-cell Differentiation and Dysfunction Inform Treatment Response in Acute Myeloid Leukemia. *Blood* (2024).
8. Daniel, B., *et al.* Divergent clonal differentiation trajectories of T cell exhaustion. *Nature immunology* **23**, 1614-1627 (2022).
9. Giles, J.R., *et al.* Shared and distinct biological circuits in effector, memory and exhausted CD8+ T cells revealed by temporal single-cell transcriptomics and epigenetics. *Nature immunology* **23**, 1600-1613 (2022).
10. Penter, L., *et al.* Mechanisms of response and resistance to combined decitabine and ipilimumab for advanced myeloid disease. *Blood* (2023).
11. Lasry, A., *et al.* An inflammatory state remodels the immune microenvironment and improves risk stratification in acute myeloid leukemia. *Nature Cancer* (2022).
12. Abbas, H.A., *et al.* Single cell T cell landscape and T cell receptor repertoire profiling of AML in context of PD-1 blockade therapy. *Nature communications* **12**, 6071-6071 (2021).
13. Dufva, O., *et al.* Immunogenomic Landscape of Hematological Malignancies. *Cancer cell* **38**, 424-428 (2020).
14. Schalck, A., *et al.* Single-Cell Sequencing Reveals Trajectory of Tumor-Infiltrating Lymphocyte States in Pancreatic Cancer. *Cancer Discov* **12**, 2330-2349 (2022).
15. Zhang, C., *et al.* A single-cell analysis reveals tumor heterogeneity and immune environment of acral melanoma. *Nat Commun* **13**, 7250 (2022).
16. Wu, F., *et al.* Single-cell profiling of tumor heterogeneity and the microenvironment in advanced non-small cell lung cancer. *Nat Commun* **12**, 2540 (2021).

Supplementary Table 1

Patients characteritics		
Age, years	Median	40
	Range	3 - 74
Gender	Male	40%
	Female	60%
ELN risk stratification	Favorable/Intermediate	53%
	Adverse	47%
Other AML characteristics	Secondary or treatment-related	27%
T_{TCRC4} virus-specificity	CMV	33%
	EBV	67%
Number of allo-HCT before T_{TCRC4} infusion	1	60%
	2	40%
Disease status at 1st T_{TCRC4} infusion	CR_{MRD-}	53%
	CR_{MRD+}	33%
	Refractory	13%
Lymphodepletion	y	20%
	n	80%
GVHD after T_{TCRC4} infusion	aGVHD	13%
	cGVHD	20%

Supplementary Table 2

Adverse events						
NCI CTCAE v4.0*		Definitely/probably associated with TTCR-C4		Unrelated/unlikely** associated with TTCR-C4		
		Grade 3	Grade 4	Grade 3	Grade 4	Grade 5
Cytokine-mediated	Cytokine Release Syndrome (fevers, chills, rigors, nausea)	2		1		
Blood and lymphatic system disorders	Lymphopenia	5	2	3	3	
	Thrombocytopenia	5	2	7	6	
	Neutrophil count decreased	8	1	12	9	
	Anemia	2		15		
GVHD-associated	Anorexia			1		
	Diarrhea			1		
	Maculo-papular rash	1		3		
	Hypoxia	1		1		
	Reduced joint range of motion			1		
	Skin sclerosis			1		
Metabolic/renal disorders	Hypokalemia			2	1	
	Hyponatremia			10		
	Hypophosphatemia			4		
	Proteinuria			1		
	Hypocalcemia			2		
	Hypoalbuminemia			1		
	Hyperglycemia			7		
	Chronic Kidney Disease			1		
Miscellaneous	Infection			6	1	
	Hypothension	2		1		
	Hypertension	2				
	Peripheral motor neuropathy			1		
	Fatigue			2		
	Duodenal Obstruction			1		
	Abdominal pain			1		
	Rectal Pain	1				
	Nausea			1		
	Aspartate Aminotransferase increased	1			1	
	Mucositis oral			2		
	Dry Mouth			1		
	Pulmonary Edema				1	
	Multi-organ Failure					1
	Alopecia				1	
	Thromboembolic event				1	
	Activated partial thromboplastin time prolonged			1		
	Sinus tachycardia			1		

*National Cancer Institute Common Terminology Criteria for Adverse Events version 4.0;
**Adverse events recorded as having definite or probable association with TTCR-C4 were considered related

Supplementary Table 3

Cell blood counts and blasts percentage pre-infusion

pt_id	WBC	%Neu	%Lymph	%Mono	%Eo	%Bas	Neu_abs	Lymph_abs	Mono_abs	Eo_abs	Bas_abs	Bl%_flow	Persistence
1	3.93	47	24	19	9	1	1.85	0.94	0.75	0.35	0.04	0	0
2	11.6	34	48	11	6	0	3.94	5.56	1.28	0.7	0	1.1	0
4	5.54	50	30	16	3	1	2.76	1.66	0.89	0.17	0.06	0	1
5	4.04	9	11	3	0	0	0.36	0.43	0.11	0	0	70.2	0
6	7.11	68	17	9	5	1	4.83	1.21	0.64	0.31	0.03	0	0
7	1.77	32	51	12	2	3	0.57	0.9	0.21	0.04	0.05	0.08	0
8	3.4	63	19	12	3	3	2.14	0.65	0.41	0.1	0.1	0.26	1
9	5.87	62	23	11	3	0	3.63	1.35	0.65	0.18	0	0	0
14	7.51	39	53	3	0	0	2.93	3.98	0.23	0	0	0.02	0
15	4.68	80	11	7	2	0	3.75	0.51	0.33	0.09	0	0	0
19	1.12	42	36	20	1	0	0.48	0.4	0.22	0.01	0	0	0
23	7.21	66	16	13	5	0	4.76	1.15	0.94	0.36	0	0	0
26	4.26	91	3	4	2	0	3.87	0.13	0.17	0.09	0	0	0
27	2.78	92	1	2	5	0	2.55	0.03	0.06	0.14	0	0	1
28	2.85	93	1	2	3	1	2.64	0.03	0.06	0.09	0.03	0	0

Absolute counts are expressed in cells/µL

Supplementary Table 4

Spectral Flow-cytometry panel					
**Marker**	Clone	Fluorescent Tag	Vendor	Catalog number	Dilution
**TIGIT**	A15153G	BV421	BioLegend	372709	2:100
**CD197 (CCR7)**	3D12	BB700	BD Biosciences	566438	1:100
**Ki-67**	B56	BV480	BD Biosciences	566172	2:100
**CD57**	NK-1	BUV395	BD Biosciences	567622	1.5:100
**CD45RA**	HI100	BV570	BioLegend	304131	0.5:100
**CD27**	O323	BV605	BioLegend	302829	1:100
**CD95 (FAS)**	DX29	BV650	BioLegend	305641	2:100
**CD127 (IL-7Ra)**	A019D5	BV711	BioLegend	351327	2:100
**CD28**	CD28.2	BV785	BioLegend	302949	1:100
**T-Bet**	4B10	KIRAVIA Blue 520	CellSignalingTechnology	14456S	2:100
**CD8**	RPA-T8	BUV737	Invitrogen	367-0088-42	2:100
**TCF1 (TCF7)**	C63D9	PE	CellSignalingTechnology	14456s	2:100
**CD279 (PD-1)**	EH12.2H7	PE-Dazzle 594	BioLegend	329939	2:100
**CD69**	FN50	PE-Cy5	BioLegend	310907	1:100
**Granulysin**	DH2	PE-Cy7	BioLegend	348012	2:100
**KLRG1**	13F12F2	PerCP-eFluor 710	Invitrogen	46-9488-42	1:100
**Granzyme K**	G3H69	RB780	BD Biosciences	569226	1:100
**Tetramer**		APC	Fred Hutchinson in-house production		1:100
**Granzyme B**	GB11	Alexa Fluor 700	BD Biosciences	560213	1:100
**CD38**	HIT2	APC-Fire 810	BioLegend	303549	1:100
**CD3**	UCHT1	APC-Fire 750	BioLegend	300469	1:100
**CD366 (TIM3)**	F38-2E2	PE-Fire 810	BioLegend	345059	2:100
**Viability**		LD Blue	Invitrogen	L34961	0.1:100
**CD33 (DUMP)**	WM53	BV510	BioLegend	303421	1:100
**CD14 (DUMP)**	63D3	BV510	BioLegend	367123	1:100

Supplementary Table 5

Spectral flow-cytometry timepoints post-TTCR-C4 infusion				
	Days after TTCR-C4 infusion			
IDs	T1	T2	T3	T4
8	1	7	28	117
4	1	7	28	110
27	3	8	28	149

Supplementary Table 6

IFN-γ production assessment by flow cytometry						
	Days after TTCRC4 infusion					
IDs	T0	T1	T2	T3	T4	T5
8	0	7	28	96	201	445
4	0	7	42	86	212	408
27	NA	7	28	149	265	NA

Percentages of tetramer+/- IFN-γ producing cells				
id	Timepoint	Tet+ %	Tet -%	log10_Tet+
4	T0	27.7	0	1.442479769
4	T1	0.3	0.41	-0.522878745
4	T2	0.33	1.36	-0.48148606
4	T3	0.27	1.64	-0.568636236
4	T4	0.5	0.89	-0.301029996
4	T5	0.095	0.58	-1.022276395
8	T0	50.3	0	1.701567985
8	T1	2.77	0.2	0.442479769
8	T2	7.27	0.59	0.861534411
8	T3	4.4	0.97	0.643452676
8	T4	1.25	0.4	0.096910013
8	T5	0.27	0.051	-0.568636236
27	T1	11.1	0.57	1.045322979
27	T2	5.14	0.85	0.710963119
27	T3	2.76	1.18	0.440909082
27	T4	1.48	0.28	0.170261715

Flow-cytometry panel					
**Antibody**	**Fluorochrome**	**Clone**	**Manufacturer**	**Catalogue Number**	**Dilution**
LIVE/DEAD	Fixable Aqua Dead Cell Stain		Invitrogen	L34957	1:100
IFNγ	V450	B27	BD Biosciences	560371	1:100
CD14 (Dump)	BV510	M5E2	BioLegend	301842	1:100
CD4	BUV395	SK3	BD Biosciences	563550	2:100
CD3	BV570	UCHT1	BioLegend	300436	1:100
CD8	BV711	RPA-T8	BD Biosciences	563677	1:100
Tetramer	APC	Fred Hutchinson in-house production			1:100

Supplementary Table 7

Timepoints for cytokine assay					
	Days after TTCR-C4 infusion				
IDs	T0	T1	T2	T3	T4
15	NA	1	21	56	97
27	NA	1	21	84	178
4	pre-infusion	NA	7	NA	110
8	pre-infusion	NA	21	NA	92

Supplementary Table 8

Single-cell RNA and TCR-sequencing timepoints after TTCR-C4 infusion						
IDs	Days after first infusion					Arm
10	D320					Prophylactic
18	D253					Prophylactic
4	D100	D581				Treatment
8	D49	D265	D405	D1322	D1343	Treatment
15	D97					Treatment
26	D7	D28				Treatment
27	D21	D58	D178			Treatment

Treatment Arm (TCR sequencing performed)
Performed
Not performed

Supplementary Table 9

Markers defining CD8+ T-cell states				
Naïve-/cm-like	Tem	ISG	Tmem-prolif	NKL/TEMRA
CCR7	GZMK	IFI6	MKI67	GNLY
LEF1	CD69	IRF7	PCNA	PRF1
LTB	CCL4	ISG20	MCM2	GZMB
BACH2	CCL5	ISG15		NKG7
IL7R	XCL1			ZEB2
TCF7	XCL2			S1PR5
				CXrCR1
				KLRD1
				KLRF1
				ZNF683
				CD226

Supplementary Table 10

AML status by timepoint			
ID	Days after 1st infusion	Status	Blasts
10	320	AML-	No blasts
18	253	AML-	No blasts
4	100	AML-	No blasts
	581	AML+	
8	49	AML+	
	256	AML-	No blasts
	405	AML+	12.50%
	1322	AML+	0.30%
	1343	AML+	0.20%
15	97	AML+	9.80%
26	D7	AML+	
	D28	AML+	9%
27	21	AML-	No blasts
	58	AML-	No blasts
	178	AML+	11%

(red)	Blasts identified via scRNAseq (Extended data fig. 15)
(green)	Described in Lahman, M.C., et al. Targeting an alternate Wilms' tumor antigen 1 peptide bypasses immunoproteasome dependency. Science translational medicine 14, eabg8070-eabg8070 (2022).

Supplementary Table 11

Overlap scRNAseq T cell states and published exhaustion/dysfunction signatures			
Overlap Tem - Tpex	Overlap Naïve-/cm-like - Tpex	Overlap Tmem/prolif - Ttex	Overlap NKL/Temra - Term/SenL
CXCR4	IL7R	TYMS	FGFBP2
NR4A2	LEF1		ZNF683
JUN	SELL		GZMH
	CCR7		ADGRG1
	TCF7		GZMB
	FOXP1		NKG7
	FOS		TBX21
			PRF1
			FCGR3A
			ZEB2

Supplementary Table 12

single-cell RNA-sequencing top 50 DEG and manually curated exhaustion signature						
**Naïve-/cm-like**	**Tem**	**TTCRC4**	**ISG**	**Tmem/prolif**	**NKL/Temra**	**exhaustion**
LTB	GZMK	TCRC4	MX1	HMOX1	GZMH	PDCD1
SELL	DUSP2	GNLY	ISG15	GAPDH	ZEB2	LAG3
NOSIP	ZNF331	PTMS	IFI6	STMN1	NKG7	HAVCR2
LEF1	NR4A2	YBX3	STAT1	ACTG1	TTC38	CTLA4
LEPROTL1	FOS	TRBV14	IRF7	CARHSP1	S1PR5	TIM3
IL7R	CSRNP1	CMC1	EIF2AK2	MCM5	GZMB	LAG3
TCF7	ZFP36L2	HOPX	PARP9	H2AFV	CST7	CD160
CCR7	JUNB	TIGIT	IFITM1	CACYBP	RAP1B	BTLA
PIK3IP1	CXCR4	RNF19A	MX2	ANP32B	SYNGR1	ENTPD1
LDHB	RGS1	LAG3	BST2	HMGB2	HLA-DRB5	
TMEM123	DNAJB1	UCP2	LGALS9	COX5A	DIP2A	
SERINC5	PTGER4	CCL5	TRIM22	S1PR4	S100A4	
NELL2	IFNGR1	FGFBP2	CD28	NUDT21	FLNA	
PIM1	CD27	FDFT1	UBE2L6	HLA-DQB1	KLRD1	
FOXP1	DUSP1	PDE4D	EPSTI1	HMGN2	HLA-DRB1	
CAMK4	PIK3R1	ALOX5AP	LY6E	HLA-DMA	EMP3	
PABPC1	RHOH	CD6	IFI35	RAD21	HLA-DPB1	
PASK	BTG1	S100A10	CHMP5	CNN2	YWHAQ	
BCL2	SLC2A3	H2AFZ	OAS1	TFDP1	CLIC3	
RCAN3	TSPYL2	KLF3	SAMD9L	TUBA1B	ANXA1	
OXNAD1	CREM	CLDND1	SAT1	HLA-DRA	C12orf75	
SARAF	ZFP36	PPP1R18	XAF1	SAE1	GSTP1	
FLT3LG	PPP1R15A	MARCKSL1	TAP1	SIRPG	BATF	
DGKA	PDE4B	ADGRG1	CARD16	TUBB	CCL4	
TRABD2A	MAP3K8	CD5	ZBP1	UQCRC1	ABI3	
TXK	IDI1	ZNF683	CD38	ANXA5	ARPC5L	
SOCS3	CD74	PRF1	PLSCR1	SLBP	APOBEC3G	
CD55	JUN	EFHD2	JAK2	CDCA7	LPCAT1	
SATB1	GABARAPL1	ITGB1	GBP1	FABP5	FCRL6	
EEF1B2	PMAIP1	PLEK	OAS3	DENND2D	DSTN	
PCED1B	TNFAIP3	SPON2	TYMP	PRDX3	PTPN18	
PIM2	EML4	FCGR3A	SAMD9	SMC3	CALR	
SESN3	PPP2R5C	PRSS23	SUB1	DUT	FGR	
TPT1	ISG20		LYST	NASP	CD3G	
MYC	SPOCK2		OAS2	ANP32E	ADRB2	
FHIT	ARL4C		LDHA	CD81	C1orf21	
ABLIM1	RSL24D1		SLFN5	MKI67		
TNFSF8	FYN		PPM1K	MCM7		
SNX9	MCL1		IFIT3	TOX		
SPINT2	KLRB1		HSH2D	CXCR3		
EEF1A1	COTL1		OASL	PCNA		
PLP2			GBP4	MCM3		
NFKBIZ						
INPP4B						
EIF3E						
NPM1						
SNHG8						
CD69						
VIM						
EIF3L						

Supplementary Table 13

KLRG1+CD57+ CD8+ T cells frequencies after Azacitidine exposure				
**Sample**	**Marker**	**Condition**	**Frequency**	**Frequency_log**
10nM AZA Day 0 and 7_C4	KLRG1+CD57+	10nM AZA Day 0 and 7	8.63	0.936010796
10nM AZA Day 0 and 7_C5	KLRG1+CD57+	10nM AZA Day 0 and 7	7.58	0.879669206
10nM AZA Day 0 and 7_C6	KLRG1+CD57+	10nM AZA Day 0 and 7	7.73	0.888179494
no AZA_E1	KLRG1+CD57+	no AZA	17.2	1.235528447
no AZA_E2	KLRG1+CD57+	no AZA	12.7	1.103803721
no AZA_E3	KLRG1+CD57+	no AZA	12.8	1.10720997

Flow panel					
**Marker**	Clone	Fluorescent Tag	Vendor	Catalog number	Dilution
**CD57**	HNK-1	FITC	BioLegend	359604	1.5:100
**CD8**	RPA-T8	BUV496	BD Biosciences	612942	1:100
**KLRG1**	13F12F2	PerCP-eFluor 710	Invitrogen	46-9488-42	1:100
**Viability**		BV510	Cell Signaling	59863S	0.1:100

Supplementary Table 14

Manually curated signatures			
**Naïve-like**	**Tem**	**NKL/Temra**	**Exhaustion**
CCR7	CCL2	GNLY	PDCD1
LEF1	CCL5	ADGRG1	LAG3
IL7R	XCL2	KLRD1	HAVCR2
LTB	XCL1	KLRB1	CTLA4
SELL	CD69	KIR2DL4	HAVCR2
TCF7	GZMK	PRF1	LAG3
	IFNG	KLRC1	CD160
		KLRG1	BTLA
		B3GAT1	ENTPD1
		S1PR5	
		ZEB2	
		NKG7	
		FCRL6	
		KLRC2	
		NCAM1	

References

1. Chapuis, A.G.*, et al.* T cell receptor gene therapy targeting WT1 prevents acute myeloid leukemia relapse post-transplant. *Nature Medicine* **25**, 1064-1072 (2019).
2. Lahman, M.C.*, et al.* Targeting an alternate Wilms' tumor antigen 1 peptide bypasses immunoproteasome dependency. *Science translational medicine* **14**, eabg8070-eabg8070 (2022).
3. Andreatta, M., Berenstein, A.J. & Carmona, S.J. scGate: marker-based purification of cell types from heterogeneous single-cell RNA-seq datasets. *Bioinformatics (Oxford, England)* **38**, 2642-2644 (2022).
4. Zheng, L.*, et al.* Pan-cancer single-cell landscape of tumor-infiltrating T cells. *Science (New York, N.Y.)* **374**, abe6474-abe6474 (2021).
5. Cai, C.*, et al.* Identification of human progenitors of exhausted CD8(+) T cells associated with elevated IFN-gamma response in early phase of viral infection. *Nat Commun* **13**, 7543 (2022).
6. Hensel, N.*, et al.* Memory-like HCV-specific CD8(+) T cells retain a molecular scar after cure of chronic HCV infection. *Nat Immunol* **22**, 229-239 (2021).
7. Mazziotta, F.*, et al.* CD8+ T-cell Differentiation and Dysfunction Inform Treatment Response in Acute Myeloid Leukemia. *Blood* (2024).
8. Daniel, B.*, et al.* Divergent clonal differentiation trajectories of T cell exhaustion. *Nature immunology* **23**, 1614-1627 (2022).
9. Giles, J.R.*, et al.* Shared and distinct biological circuits in effector, memory and exhausted CD8+ T cells revealed by temporal single-cell transcriptomics and epigenetics. *Nature immunology* **23**, 1600-1613 (2022).
10. Penter, L.*, et al.* Mechanisms of response and resistance to combined decitabine and ipilimumab for advanced myeloid disease. *Blood* (2023).
11. Lasry, A.*, et al.* An inflammatory state remodels the immune microenvironment and improves risk stratification in acute myeloid leukemia. *Nature Cancer* (2022).
12. Abbas, H.A.*, et al.* Single cell T cell landscape and T cell receptor repertoire profiling of AML in context of PD-1 blockade therapy. *Nature communications* **12**, 6071-6071 (2021).
13. Dufva, O.*, et al.* Immunogenomic Landscape of Hematological Malignancies. *Cancer cell* **38**, 424-428 (2020).
14. Schalck, A.*, et al.* Single-Cell Sequencing Reveals Trajectory of Tumor-Infiltrating Lymphocyte States in Pancreatic Cancer. *Cancer Discov* **12**, 2330-2349 (2022).
15. Zhang, C.*, et al.* A single-cell analysis reveals tumor heterogeneity and immune environment of acral melanoma. *Nat Commun* **13**, 7250 (2022).
16. Wu, F.*, et al.* Single-cell profiling of tumor heterogeneity and the microenvironment in advanced non-small cell lung cancer. *Nat Commun* **12**, 2540 (2021).

IRO REC'D MAY 22 2019

FRED HUTCHINSON CANCER RESEARCH CENTER (FHCRC)
UNIVERSITY OF WASHINGTON SCHOOL OF MEDICINE (UW)
DEPARTMENT OF MEDICINE, DIVISION OF ONCOLOGY
SEATTLE CHILDRENS

Current Version: 05/15/2019
Previous Version: 12/28/2018

PROTOCOL 2498.00

Title of Protocol: Phase I/II study of adoptive immunotherapy after allogeneic HCT with virus specific $CD8^{+}$ T cells that have been transduced to express a WT1-specific T cell receptor for patients with relapsed AML

Investigator	Professional Title	Telephone Number
Aude Chapuis, MD	Assistant Member, FHCRC<br>Assistant Professor, UW	(206) 667-4369
Daniel Egan, MD (PI)	Affiliate Investigator	
Merav Bar, MD	Affiliate Investigator, FHCRC<br>Affiliate Assistant Professor, UW	Pager: (206) 540-0168
Philip D. Greenberg, MD	Member and Head, Program in Immunology, FHCRC, Professor, UW	(206) 667-4462
Tom Schmitt, PhD.	Postdoctoral Fellow, FHCRC	(206) 667-2787
Jerald P. Radich, MD	Member, FHCRC, Professor, UW	(206) 667-4118
Ann E. Woolfrey, MD	Member, FHCRC, Professor, UW	(206) 667-4453
Ann Dahlberg, MD	Assistant Member, FHCRC	(206) 667-1959
Cecilia Yeung, MD	Assistant Member, FHCRC	(206) 288-7104
Frederick R. Appelbaum, M.D.	Member and Executive VP and Deputy Dir., Clinical Research Division, FHCRC Professor and Head, Division of Medical Oncology, UW	(206) 667-4412

Biostatistician

Ted A. Gooley, PhD	Member, FHCRC,<br>Affiliate Professor, UW	(206) 667-6533

Research Staff

Elizabeth Barnum, RN	Research Nurse	(206) 667-3792

Emergency (24 hours) Phone: (206) 598-8902; Fax: (206)-598-4034

FHCRC IRB Approval

MAY 22 2019

Table of Contents

2. INTRODUCTION **5**
3. BACKGROUND **5**
3.A. HCT and Donor Lymphocyte Infusion as a Treatment for High Risk AML, MDS, and CML 5
3.B. Rationale for Treating AML, MDS, and CML with WT1-specific CD8+ T Cells 6
3.C. Rationale for Using Donor-derived CD8+ T Cells Transduced with Lentivirus to Express High Affinity WT1-specific TCR for Patients with High Risk or Relapsed AML, MDS, and CML 7
3.D. Rationale for Transducing Donor derived EBV- or CMV-specific CD8+ T Cells to Express High Affinity WT1-specific TCR 8
3.E. Rationale for Treating Patients with High Risk AML, MDS, or CML with Prophylactic Infusions of WT1-specific T cells (Arm 1 of the Protocol) 9
3.F. Rationale for Treating Patients with Evidence of Persistent or Relapsed Disease Post-transplant (Arm 2 of the Protocol) 10
3.G. Rationale for the Proposed WT1-specific T Cell Doses to be Infused in this Study 10
3.H. IL-2 Administration to Augment Persistence of Adoptively Transferred T Cells 12
3.I. Safety Concerns Regarding Using Donor-derived EBV- or CMV-specific CD8+ T cells Lentivirally Transduced to Express a High Affinity WT1-specific TCR 13
3.J. Rationale for Allowable Doses of Immunosuppressive Therapy During the Study 15
3.K. Previous Human Experience with TCR_{C4}: BB-IND 12046 – FHCRC Protocol 1655: Phase I/II Study of Adoptive Immunotherapy with CD8+ WT1-specific CTL Clones for Patients with Advanced MDS, CML, AML, or ALL after Allogeneic Hematopoietic Stem Cell Transplant. 18
3.L. Ongoing Human Experience with TCR_{C4} in the current study (FHCRC protocol 2498 Study) 18
3.L.1. Patient characteristics on Protocol 2498 20
3.L.2. Persistence of TCR_{C4}-transduced cells on Protocol 2498 22
3.L.3. Toxicities observed on protocol #2498 24
3.L.4. Efficacy observed on protocol #2498, Arm 1 27
3.L.5. Efficacy observed on protocol #2498, Arm 2 28
3.L.6. Protocol #2498: Conclusions to date 29
3.M. Rationale for Lymphodepleting Chemotherapy Prior to T Cell Therapy 29
3.N. Rationale for Adjusting Eligibility Criteria for Ongoing Treatment on Protocol 2498 30
4. OBJECTIVES **30**
4.A. Primary Objectives 30

4.B. Secondary Objectives 30

5. STUDY ENDPOINTS 31

5.A. Primary Endpoints 31

5.B. Secondary Endpoints 31

6. STUDY SCHEMA 31

7. PATIENT SELECTION 32

7.A. Eligibility for Enrollment 32

7.B. Exclusions for Enrollment 32

7.C. Definition of Relapse Status or MRD 33

8. DONOR SELECTION 33

8.A. Inclusions 33

8.B. Exclusions 33

9. CONSENTING 34

10. PROTOCOL REGISTRATION 34

11. PROCEDURE TO OBTAIN PBMC FOR GENERATION OF WT1-SPECIFIC T CELLS 34

12. GENERATION OF WT1-SPECIFIC T CELLS 35

13. T CELL PRODUCT HANDLING PRIOR TO INFUSION 35

14. PLAN OF TREATMENT 35

14.A. Plan of Treatment – Arm 1 35

14.B. Plan of Treatment – Arm 2 39

14.C. Other Study Agents 43

14.C.1. Interleukin-2 43

14.C.2. Cyclophosphamide 43

14.C.3. Fludarabine 43

15. EVALUATION 43

15.A. Patient and Donor Screening Prior to Enrollment 44

15.B. Patient Evaluation after Transplant and Prior to Planned Initiation of CTL Infusions 44

15.C. Patient Evaluation at Time of Planned Initiation of T Cell Infusions (within 30 days of T cell infusion, unless otherwise indicated) 45

15.D. Evaluation Immediately Prior to each T Cell Infusion (Day 0) 45

15.E. Evaluation During Each T Cell Infusion (Day 0) 46

15.F. Evaluation After Each T Cell Infusion 46

15.G. Clinical and Laboratory Evaluation for Toxicity Following T Cell Infusions 47

15.H. Evaluation of Persistence and Function of Adoptively Transferred T Cells and for Evidence of Leukemia/MDS 49

15. I. Evaluation for long term effects of using lentivirally transduced T cells 50

16. MANAGEMENT OF T CELL INFUSION IN PATIENTS WHO RELAPSE OR PROGRESS DURING T CELL THERAPY 51

17. OPTIONS FOR FURTHER TREATMENT AFTER COMPLETION OF STUDY THERAPY. 51
18. TOXICITIES ... 51
18.A. Toxicity Grading ... 51
18.B. Immediate Toxicities: ... 51
18.C. Delayed Toxicities ... 53
18.D. Management of Severe Treatment-related Toxicities with Methylprednisolone ... 53
18.E. Definition and Management/Evaluation of Non-hematologic and Hematologic Toxicities Requiring Treatment Discontinuation ... 53
18.F. Chronic GVHD ... 55
18.G. Detection of Replication Competent Lentivirus (RCL) ... 55
20. PROTOCOL REGISTRATION AND SPECIAL CONSIDERATIONS ... 56
20.A. Projected Target Accrual - Arm 1 ... 56
20.B. Projected Target Accrual - Arm 2 ... 56
21. GUIDELINES FOR ADVERSE EVENT REPORTING ... 57
21.A. Reporting of Adverse Events (AEs) ... 57
21.B. Definitions ... 57
22. DATA AND SAFETY MONITORING PLAN ... 58
22.A. Primary Monitoring ... 58
22.B. Monitoring Plan ... 58
22.C. Monitoring the Progress of Trials and the Safety of Participants ... 58
23. RECORDS ... 59
24. STATISTICAL CONSIDERATIONS ... 60
24.A. Analysis of Toxicity (Arm 1 and Arm 2) ... 61
24.B. Analysis of Efficacy in Preventing Relapse (Arm 1) ... 64
24.C. Analysis of Anti-leukemic Potential Efficacy (Arm 2) ... 65
25. TERMINATION OF THE STUDY ... 66
26. REFERENCES ... 67
27. APPENDICES ... 74
APPENDIX A ... 74
APPENDIX B ... 75
APPENDIX C ... 78
APPENDIX D ... 79
APPENDIX E ... 81
APPENDIX F ... 82

2. INTRODUCTION

Allogeneic hematopoietic cell transplantation (HCT) has been shown to be an effective treatment for many otherwise incurable hematological malignancies, which at least partially reflects a therapeutic "graft-versus-leukemia" (GVL) effect mediated by transferred T cells.[1] The benefit of GVL is often accompanied by harmful side effects, including graft-versus-host disease (GVHD).[2,3] One way to harness the GVL activity of T cells and reduce toxicity might be to target the leukemia cells with $CD8^+$ cytotoxic T cells (CTL) specific for antigens expressed by the malignant cells. Wilms' tumor antigen 1 (WT1), a protein involved in regulation of gene expression that can promote the malignant phenotype, is over-expressed in Acute Myeloid Leukemia (AML), Myelodysplastic Syndrome (MDS), and Chronic Myeloid Leukemia (CML),[4-7] as well as many other malignancies. WT1-specific CTL can recognize and kill both in vitro and in vivo WT1-expressing leukemia cells, which express abnormally high levels of WT1, without affecting normal cells that express much lower levels.[8-14]

Our group has treated patients with relapsed leukemia after allogeneic HCT on a clinical trial using WT1-specific $CD8^+$ T cell clones generated and expanded from each patient's normal human leukocyte antigen (HLA)-matched donor. Results indicate that this treatment has a very acceptable safety profile, but anti-leukemic efficacy has been limited. Analysis of the reasons for failure to reproducibly provide therapeutic benefit suggests this reflects in some patients' poor persistence in vivo of the infused T cells and/or low avidity of the T cells for the leukemic target cells. In this phase I/II clinical trial, Epstein Barr virus (EBV)- or cytomegalovirus (CMV)-specific memory T cells that have the potential for longer in vivo persistence following transfer because they have already been programmed with the traits of memory T cells will be obtained from each patient's HLA-matched donor and transduced with a lentiviral vector to express a characterized high affinity WT1-specific T cell receptor (TCR). The transduced cells will be expanded and infused into AML, MDS, or CML patients who are at high risk of relapse after HCT. The objectives of the study are to assess if the transduced cells can be safely administered to patients, maintain their function and persist *in vivo*, localize to sites of disease, and potentially prevent or treat relapse after HCT.

3. BACKGROUND

3.A. <u>HCT and Donor Lymphocyte Infusion as a Treatment for High Risk AML, MDS, and CML</u>

Every year about 13,000 people are diagnosed with AML, 11,000 with MDS, and 5,000 with CML in the United States, with the incidence increasing in the advanced age group.[15] AML is often incurable with standard systemic therapy, as evidenced by a one year relapse rate of ~50% in the ~80% of AML patients with unfavorable cytogenetics[16,17] and ~ 90% relapse rate in AML patients who are induced into second remission with chemotherapy only.[18-20] High risk MDS patients, as defined by an International Prognostic Scoring System (IPSS) score of 1.5-2 without therapy, have a median survival of 1.1 year, and those with an IPSS score >2.5 a median survival of 0.4 year.[21] Patients with CML beyond chronic phase (CP) have a poor prognosis, especially if disease has progressed into blast phase (BP) (3% three-year survival).[22] Allogeneic HCT has been shown to have curative potential for patients with high risk AML, high risk MDS or CML in accelerated or blast phase, partially as a result of a GVL effect mediated by alloreactive T cells.[1]

However, despite the many advances in the field of HCT,[23] relapse after transplantation continues to be a major problem, particularly in patients entering HCT with high risk/poor prognosis diseases. Patients with MDS or AML with poor prognostic factors, who receive myeloablative transplants from HLA-identical siblings, have a relapse rate of ~25% at three months and ~40% at one year post-transplant.[24-28] Approximately 32% of patients undergoing non-myeloablative transplant for high risk AML have clinically relapsed or have detectable minimal residual disease (MRD) three months after transplant, and there is a three year relapse/progression rate after HCT of 55%.[29] Relapse of AML or MDS after allogeneic HCT is typically characterized by chemo-refractory and rapidly progressive disease, with >75% of patients dying within a year.[30,31]

Current options for patients who relapse after HCT include salvage chemotherapy, withdrawal of immunosuppressive therapy (WIST), donor lymphocyte infusion (DLI) and a second HCT. Although WIST followed by DLI has shown some long term benefit in the small number of patients who both relapse >6 months after HCT and can be successfully induced into remission by chemotherapy before DLI, in general efficacy is limited and the desired GVL effect is often accompanied or overshadowed by harmful side effects, including ~10% of patients developing grade III-IV acute GVHD within 30 days of DLI and an overall rate of grade III-IV acute GVHD of 20-30%.[2,3,32-34] A second HCT has some curative potential, but is associated with high morbidity and ~50% mortality rates.[35]

Patients with chronic myeloid leukemia (CML) in either accelerated phase (AP) or blast crisis (BC) who receive transplants from an HLA-identical allogeneic donor have a probability of leukemic relapse post-transplant of approximately 50%, with most occurring within the first two years.[36-39] Similar to relapsed AML after HCT, treatment of patients who relapse with CML in AP or BC with salvage chemotherapy, interferon, imatinib mesylate, or DLI has yielded few durable remissions.[2,3,40,41] This markedly contrasts to results with DLI given to patients who have relapsed after HCT for CML in chronic phase, where ~75% of patients attain remission,[2,3] suggesting the leukemia in this setting is more susceptible to immunologic effector mechanisms not specifically targeted to the leukemia. However, as described above, DLI is also associated with significant toxicity from GVHD. Ultimately, alternative more-targeted options for the treatment of relapsed leukemia post-transplant are required.

3.B. Rationale for Treating AML, MDS, and CML with WT1-specific $CD8^+$ T Cells

One way to maximize the GVL effects with less toxicity would be to selectively eliminate leukemia cells using $CD8^+$ CTL specific for antigens expressed by the leukemia cells, but absent or expressed at only low levels by normal tissue. WT1 is a zinc finger, a protein involved in regulation of gene expression that has multiple functions, including gene transactivation, gene repression, and RNA binding.[42] WT1 is an intracellular protein involved in the activation and repression of gene expression and regulation of posttranscriptional RNA processing.[43] Up to 24 different isoforms of WT1 can be generated by alternative splicing, alternative translation initiation, and RNA editing.[43,44] All WT1 isoforms have four zinc finger domains at the carboxyl terminus of the protein, which are involved in binding to DNA motifs in the promoter region of WT1-regulated genes.[45] In one isoform, insertion of three amino acids, lysine, threonine, serine (KTS) in the zinc finger domains by alternative splicing produces a WT1 isoform that is primarily involved in RNA processing. WT1 can be detected at low levels in adult podocytes of the kidney, sertoli cells of the testes, granulosa cells of the ovary, mesothelial cells of the lung, the uterus, and $CD34^+$ hematopoietic progenitor cells.[46] Over-expression of WT1 has been reported in various solid tumors, as well as hematologic malignancies, including ~90-100% of AML,

MDS, and CML.[5] WT1 has been shown to be expressed in blast cells of the vast majority of patients with AML.[5,47]

WT1 appears to have a role in oncogenesis, as suggested by studies showing inhibition of proliferation and induction of apoptosis of leukemia cells and other tumors following specific down-regulation of WT1 expression by antisense oligonucleotides.[7] Additionally, WT1 increases expression of the anti-apoptotic molecule bcl-2, and constitutive expression of WT1 in hematopoietic progenitor cells increases proliferation and inhibits differentiation of myeloid cells in response to granulocyte colony-stimulating factor (G-CSF).[6] Unfractionated leukemic cells express approximately 10 to >100-fold the level of WT1 protein found in normal $CD34^+$ cells[46] with even higher levels detected in $CD34^+/CD38^-$ leukemic stem cells compared to normal hematopoietic stem cells.[48] This difference has made it possible to monitor WT1 expression as a sensitive molecular marker for relapse of leukemia and for progression of MDS from refractory anemia to more malignant forms.[49-52]

Immunologic studies have demonstrated $CD8^+$ T cells can also distinguish cells based on this difference in protein expression, suggesting it may be safe to therapeutically target the WT1 protein. Using peptides representing epitopes predicted to bind the A2 and A24 human Class I molecules, $CD8^+$ CTL have been generated that lyse leukemia cells but not normal $CD34^+$ cells, and inhibit growth of leukemia colonies but not myeloid or erythroid colonies from normal $CD34^+$ cells.[8-10]

In vivo studies in mice demonstrated that WT1-specific $CD8^+$ T cells inhibit engraftment of leukemia-initiating stem cells but not normal $CD34^+$ hematopoietic cells in non-obese diabetic/severe combined immunodeficiency (NOD/SCID) mice[9] and eliminate $WT1^+$ leukemia cells in vivo without any evidence of injury to normal tissues, including kidney, lung, and bone marrow.[14] Finally, detection of WT1-specific $CD8^+$ T cells in patients with myeloid and lymphoblastic leukemias has been associated with beneficial GVL effects,[53-56] and clinical vaccine trials with WT1 peptide have shown not only that $CD8^+$ T cells can be induced from patients' T cells but that induction of a WT1-specific CTL response is associated with anti-leukemic activity in some patients including complete responses (CRs) in a few patients with advanced disease.[57-62]

The WT1-specific TCR being used in this protocol (C4 TCR) (described in **Section 3.C**) targets the $WT1_{126-134}$ epitope (RMFPNAPYL), which is highly conserved among species, expressed in all WT1 isoforms, and has not been reported as hotspot for mutation in AML. In fact, no mutations of this epitope have been reported. This epitope is presented by the class I major histocompatibility complex (MHC) HLA-A*0201, and is considered a promising lead for immunotherapeutic targeting.[63]

3.C. Rationale for Using Donor-derived $CD8^+$ T Cells Transduced with Lentivirus to Express High Affinity WT1-specific TCR for Patients with High Risk or Relapsed AML, MDS, and CML

A review of cancer vaccine trials involving 440 patients revealed an objective clinical response rate of <3%, highlighting the substantive obstacles to generating an effective anti-tumor immune response in cancer patients,[64,65] and, similarly, the majority of patients that have been vaccinated to WT1 have failed to achieve clinical benefit. Adoptive T cell therapy, in which the tumor-reactive T cells are generated and expanded in the laboratory and then infused into the patient, can overcome some of the obstacles to establishing an effective response. Our lab has

developed methods to generate high avidity $CD8^+$ T cell responses specific for WT1 by primary in vitro sensitization of naïve T cells (T_N) from healthy donors,[66] and we have been performing a clinical trial in allogeneic HCT patients with relapsed leukemia, in which WT1-specific $CD8^+$ T cell clones are generated and expanded from each patient's normal matched donor for use in therapy **BB-IND 12046 – FHCRC Protocol 1655 – Phase I/II Study of Adoptive Immunotherapy with $CD8^+$ WT1-specific CTL Clones for Patients with Advanced MDS, CML, AML, or ALL after Allogeneic Hematopoietic Stem Cell Transplant [BB-IND 12046 - FHCRC Protocol 1655)**. Although we have been successful in generating WT1-specific $CD8^+$ T cell clones from >85% of healthy donors, the avidity of the T cells generated from each donor for WT1 is variable and the lower avidity responses may have had limited anti-leukemic activity.

Transfer of high affinity TCR genes into primary T cells as a strategy to impart specificity and high avidity for a cell expressing the desired target antigen can circumvent this problem. Genes encoding the α and β chains of a TCR have already been introduced into T cells and clinically evaluated in patients,[67] and T cells transduced with a MART-1 or gp100 specific TCR were shown in patients with metastatic melanoma to migrate to the site of disease and mediate cancer regression.[68,69]

One potential limitation to use of TCR-transduced T cells is that such T cells can exhibit decreased avidity compared to the original "donor" T cell due to reduced expression of the introduced TCR. However, our lab described the introduction of a point mutation that inserts a cysteine (cys) into the Cα and the Cβ domains of a WT1-specific A*0201-restricted TCR isolated from a high avidity $CD8^+$ T cell clone to create a disulfide-bond.

The cys-modification promoted preferential pairing of the introduced α and β chains with each other, resulting in more efficient expression of the introduced TCR compared to the unmodified TCR, and reduced mismatching with endogenous TCR chains, resulting in the transduced T cells demonstrating higher avidity for WT1-expressing leukemia cells compared to cells expressing chains without the mutation.[70] Another modification that has proven beneficial for increasing TCR transgene expression is codon optimization. Redundancy in the genetic code allows some amino acids to be encoded by more than one codon, but certain codons are less 'optimal' than others due to the relative availability of matching tRNAs as well as other factors.[71] Modifying the TCRα and β gene sequences to encode each amino acid by the optimal codon for mammalian gene expression, and to eliminate mRNA instability motifs and cryptic splice sites, has been shown to significantly enhance TCRα and β gene expression.[72]

The WT1-specific TCR being used in this protocol (C4 TCR) was selected after screening >1000 T cell clones isolated from the repertoires of normal healthy individuals for avidity as reflected by WT1-specific cytolytic activity for targets expressing decreasing levels of WT1 and affinity for peptide/MHC tetramers. Thus, patients on this protocol will have their donor $CD8^+$ T cells transduced with a lentiviral vector containing the C4 WT1-specific TCR chains that have been cys-modified and codon optimized.

3.D. <u>Rationale for Transducing Donor derived EBV- or CMV-specific $CD8^+$ T Cells to Express High Affinity WT1-specific TCR</u>

T cell therapy is often limited by the ability of transferred T cells to expand and persist in vivo after transfer. The in vivo fate of transferred T cells is dependent in part on the intrinsic properties of the T cells from which infused cells were derived, and TCR gene therapy provides the opportunity to choose the nature and origin of the T cell type used for adoptive cell therapy.

Conventional CD8+ T cells can be divided into naïve T cells (T_N), and antigen experienced memory T cells (T_M). Memory T cells can be further divided into central memory T cell (T_{CM}) and effector memory T cell (T_{EM}) subsets, which have distinct transcriptional programs that dictate many characteristics, including homing, phenotype, and function.[73] When T_N and T_{EM} cells are stimulated in vitro, they expand and differentiate largely into short-lived effector cells, which effectively kill targets, but generally have limited proliferative capacity and fail to persist for long periods after transfer in vivo.[73-76] Although T_{CM} cells also expand and differentiate into effector cells in response to in vitro stimulation, studies in non-human primates indicate that these effector cells have been imprinted and retain some of the beneficial properties of the parent T_{CM} cell from which they were derived, and in particular the capacity for self-renewal, which translates into improved in vivo persistence and response to antigen challenge in vivo.[74]

Over 90% of the human population is infected with EBV,[77] and the sero-prevalence of CMV is ~60% in the total US population (40-49 year old: ~65%; 50-59 year old: ~74%; 60-69 year old: 83%),[78] with almost the entire population being either EBV or CMV seropositive. The CD8+ T cells that are reactive with these viruses contain cells of central memory origin, with the proportion varying between donors, but in general is represented in a higher fraction among EBV-specific T cells.[79-83] Therefore, introducing the WT1-specific high affinity TCR into EBV-specific CD8+ T cells (or CMV-specific if the donor is EBV negative) may result in a more persistent and potent anti-leukemic response by the transduced T cells.

In addition to ensuring the transduced T cells will contain a large fraction of T cells derived from the T_{CM} cell pool, which have properties that enhance persistence after transfer, the use of virus-specific cells serves two additional purposes: 1) it restricts the antigen specificity of the endogenous TCRs expressed by the substrate cells to a known foreign pathogen, decreasing the likelihood of transducing donor-derived T cells that recognize host antigens; and 2) it removes the possibility of transducing T_N cells that contribute disproportionally to GVHD following HCT. [84,85]

3.E. Rationale for Treating Patients with High Risk AML, MDS, or CML with Prophylactic Infusions of WT1-specific T cells (Arm 1 of the Protocol)

Patients with high risk AML, CML, or MDS, but with no evidence of disease post HCT are eligible for treatment on Arm 1 of this study. The relapse rate for high risk AML, MDS, and CML after HCT remains high, with ~40-55% of patients eventually relapsing,[24-29] and 85% of the relapses occurring within two years post HCT (FHCRC data). Treatment options available for relapsed leukemia after transplant generally have limited efficacy and/or are very toxic. Palliative therapy or enrollment in experimental clinical trials is therefore often considered in this patient group. The high risk of relapse and poor prognosis of relapsed patients highlights the importance of developing and testing strategies in patients with high risk AML, MDS, or CML that might prevent relapse, such as prophylactic infusion of WT1-specific T cells. Grade II-IV acute GVHD after allogeneic HCT requiring high dose glucocorticoids occurs in about 70% of patients (with grade III-IV 15-20%), with the cumulative incidence reaching a plateau by day +60 after myeloablative transplant and day +90 after non-myeloablative transplant.[86] Patients diagnosed with grade III-IV acute GVHD will be excluded from this protocol, as the use of drugs such as high doses of glucocorticoids would ablate the infused T cells. Patients with high risk AML, MDS, or CML, with no evidence of disease post HCT, and with no grade III-IV acute GVHD will be eligible to enroll in Arm 1 of this protocol.

3.F. Rationale for Treating Patients with Evidence of Persistent or Relapsed Disease Post-transplant (Arm 2 of the Protocol)

Patients with evidence of residual or relapsed AML post-HCT are eligible for treatment on Arm 2 of this study. Immune-based therapies are generally ineffective in the setting of large established tumor burdens, and such patients frequently have confounding toxicities from tumor progression. In murine leukemia models, the transfer of leukemia-specific T cells has been shown to be effective in mice with established disease only when the leukemia burden has been reduced with chemotherapy prior to T cell infusions.[87] The lack of efficacy observed with advanced disease likely reflects multiple factors, including faster growth kinetics of the leukemia cells as compared to the infused CTL, alterations in T cell signal transduction (particularly of tumor-reactive T cells) in the tumor-bearing host, induction of T cell apoptosis, the presence of non-specific and/or specific regulatory cells that suppress the anti-tumor response, and the induction of antigen-specific T cell anergy during the course of tumor progression.[88,89] Therefore, it would likely be difficult to detect anti-leukemic effects of infused CTL if only patients with advanced disease were treated.

Patients on this study who have evidence of residual disease post-HCT (Arm 2) will preferentially receive WT1-specific CTL infusions after detection of MRD – for example, immediately following detection of molecular relapse/progression by polymerase chain reaction (PCR) for leukemia-specific gene markers or detection of cytogenetic relapse/progression by analysis of bone marrow.

3.G. Rationale for the Proposed WT1-specific T Cell Doses to be Infused in this Study

In a prior **Phase I/II BB-IND 12046 – FHCRC Protocol 1655** study, the most avid WT1-specific T cell clones that could be derived from sibling donors were used to treat 11 patients with high risk or relapsed leukemia post HCT. The first four patients on the study received a starting dose of 3.3 x 10^8 cells/m^2 followed by a dose of 1 x 10^9 cells/m^2 WT1-specific T cells. No unexpected adverse events (AEs) were observed among those patients. However, infused multimer$^+$ CTL frequencies did not exceed 0.05% of total $CD8^+$ T cells (limit of detection) after 3-7 days, suggesting that doses up to 1 x 10^9 cells/ m^2 were insufficient to establish persistent responses. Therefore, the initial dose for the last seven patients was increased to 3.3 x 10^9 cells/m^2, followed by two doses of 1 x 10^{10}cells/m^2. The last T cell infusion was followed by 14 days of low dose interleukin-2 (IL-2) administered subcutaneously (SC). With the exception of transient lymphopenia, which is consistently observed after antigen-specific CTL infusions,[90] only two cases of flu-like symptoms and one case of grade ≥3 fatigue were observed (**Table 1**). No grade II-IV GVHD was attributed to the T cell therapy.

Table 1. AEs on BB-IND 12046 – FHCRC Protocol 1655

**Grade ≥ 3 AE related to CTL infusion**	**19**
Lymphopenia	16
Flu-like syndrome	2
Fatigue	1

Based on the observation that the frequencies of multimer+ CTL reached baseline undetectable levels within 3-7 days with infusions up to 1 x 10^9 cells/m^2 donor-derived WT1-specific T cells, and that no toxicities were observed with doses up to 1 x 10^{10} cells/m^2, the proposed starting WT1-specific T cell dose for the current study was one log lower than the maximum dose administered on Protocol 1655 or 1 x 10^{10} cells/m^2. The maximum WT1-specific T cell dose to be infused in this study, in both arms, is 1 x 10^{10} cells/m^2, which was safely administered in our previous study.

As of February 1, 2014 seven patients have been treated on the current study, and collectively received 17 T cell infusions represented the maximum target dose of 1 x 10^{10} WT1-specific CTL/m^2.

All adverse events were evaluated starting from the time of the first infusion to 30 days after the patients had taken the last dose of s.c. IL-2, and AEs that were deemed possibly, probably or likely related were collected and graded according to NCI CTCAE v4.0 (**Table 2**).

Categories	NCI CTCAE v4.0[1]	Grade 1	Grade 2	Grade 3	Grade 4
**Cytokine Release Syndrome**	Fever	5	3	1	
	Chills	2	1	1	
	Generalized aches/pain/headache	10	2		
	Fatigue	1	1		
	Tachypnea	4			
	Hypotension	5	3	1	
	Sinus tachycardia	4	2		
	Sinus bradycardia	1			
	Nausea	3	3		
	Vomiting	1			
	Diarrhea	2			
**Hematological Abnormalities**	Lymphopenia	3	6	4	1
	Lymphocyte count increased		2		
	Anemia	1	1		
	Thrombocytopenia	1	2	2	
**Chemistry Abnormalities**	ALT increased	6	2		
	AST increased	8			
	Alkaline phosphatase increased	4			
	Creatinine increased	3			
	Hypoalbuminemia	4			
	Hypocalcemia	7			
	Hypomagnesemia	1			
	Hyponatremia	5			

	Hypokalemia	3			
**Miscellaneous**	Dry eyes		2		
	Decreased respiratory rate	4			

[1] National Cancer Institute Common Terminology Criteria for Adverse Events version 4.0.

Table 2. Protocol 2498 Adverse events

Adverse events experienced by patients treated on protocol 2498 have been reviewed by the FDA, and in March 2014 an approval from the FDA was received to decrease the number of T cell infusion to two, to include the original last two infusions at a dose of 1×10^{10} WT1-specific T cell/m^2.

In our previous experience with **BB-IND 12046 – FHCRC Protocol 1655**, *in vivo* activity was only observed in patients with detectable transferred cells in the peripheral blood and/or bone marrow and no activity was observed in the absence of detectable WT1-specific CTL. Furthermore, no end-organ toxicities were observed with frequencies of transferred donor-derived WT1-specific CTL >6% for >160 days. Therefore, to limit the potential risk of toxicity until safety has been demonstrated subsequent infusions of C4 CTL will be administered only if the frequency of WT1-specific T cells is ≤3% of total CD8$^+$ T cells in the peripheral blood (50% of the long-term tolerated dose on **BB-IND 12046 – FHCRC Protocol 1655**).

For **BB-IND 12046 – FHCRC Protocol 1655**, the most avid WT1-specific T cell clones that could be derived from sibling donors were used to treat eleven patients with high risk or relapsed leukemia post HCT. CTL clones with similar/higher avidities for peptide-pulsed T2 B-LCL, and an increased capacity to lyse established leukemia cells lines compared to C4 TCR transduced EBV$^+$ CD8$^+$ cell products (C4 CTL) have been infused into high-risk post-transplant patients and were safe with no CTL-related end-organ toxicities.

Additional safety data have been generated in pre-clinical studies in our lab (unpublished data). In these studies, splenocytes from P14 mice were transduced to express unmodified TCR-α and -β genes isolated from a WT1-specific murine T cell clone, or enhanced affinity TCR-α and -β genes generated by saturation mutagenesis of the CDR3α region of the wild-type TCR-α chain to yield a TCR with a > 100-fold higher affinity. Recipient B6 mice were infused with 1×10^7 CD8$^+$ gene-modified T cells. High frequencies (10-15%) of infused cells were achieved and detectable approximately one week after infusion, and there was no evidence of end organ toxicity (including bone marrow, kidney, and lung) in mice that received T cells expressing either the wild-type or enhanced affinity TCR up to six weeks after infusion.
To limit the potential risk of toxicity until safety has been demonstrated, subsequent planned infusion of WT1-specific T cells will be delayed until the frequency of WT1-specific T cells is ≤3% of CD8$^+$ T cells (see **Sections 14 and 24**).

3.H. IL-2 Administration to Augment Persistence of Adoptively Transferred T Cells

IL-2 is a cytokine secreted by activated T cells with a crucial role in the generation of an effective immune response. IL-2 promotes the activation and proliferation of antigen-specific T cells via the high affinity three-chain (αβγ) IL-2 receptor, which is induced following TCR triggering, and at high concentrations can also activate resting natural killer (NK) cells and T cells via the intermediate affinity two-chain (βγ) IL-2 receptor. Studies in cancer patients have

evaluated IL-2 alone, with adoptively transferred in vitro-cultured lymphokine-activated killer (LAK) cells generated by culture in high dose IL-2, or with tumor infiltrating lymphocytes (TIL).[91]

These studies used high doses (up to 50 x 10^6 units every 8 hours) of intravenous or subcutaneous IL-2. Although clinical responses were observed in a fraction of patients with renal cell carcinoma or melanoma, severe toxicity was a significant problem. The plasma levels obtained with high dose IL-2 exceed the concentrations needed to saturate intermediate affinity receptors, and can therefore induce non-specific widespread activation of NK cells and T cells that can mediate the observed toxicities via cytokine release and lysis of normal cells. However, $CD8^+$ T cells activated by target recognition in vivo express high affinity IL-2 receptors and are responsive to very low concentrations of IL-2, suggesting that the doses necessary to augment survival and in vivo persistence of transferred T cells may be much lower than the doses that induce toxicity.

Studies in cancer patients and HIV seropositive individuals have investigated the immunomodulatory effects of administering lower doses of IL-2. Doses of 1.25 x 10^5 to 5 x 10^5 U/m^2/day administered subcutaneously have been shown to be well tolerated for up to 84 days.[92] In cancer patients, there is little evidence for anti-tumor activity from administration of low dose IL-2 alone. However, studies from our group in melanoma patients suggest that the persistence, in vivo function, and anti-tumor activity of adoptively transferred $CD8^+$ melanoma-specific CTL clones can be significantly enhanced by daily SC administration of low doses of IL-2 for 14 days following T cell transfer.[93]

The dose of IL-2 that will be used in this study was chosen based on its predicted ability to saturate high affinity IL-2 receptors and to sustain CTL activity/survival with minimal toxicity. Patients who have been treated on **BB-IND 12046 - FHCRC Protocol 1655** received the same dosing of IL-2 planned for this trial and it has been well tolerated, with no evidence of induction of GVHD from non-specific T cell activation.

3.I. Safety Concerns Regarding Using Donor-derived EBV- or CMV-specific $CD8^+$ T cells Lentivirally Transduced to Express a High Affinity WT1-specific TCR

Expression of WT1 can be detected at low levels in normal kidneys, testes, ovary, pleura, pericardium, the uterus and in $CD34^+$ hematopoietic progenitor cells.[46] Patients who were treated on **BB-IND 12046 – FHCRC Protocol 1655** did not experience toxicities in any of these organs expressing physiologic levels of WT1, such as hematopoietic suppression or graft failure, renal failure, pleuritis or pericarditis, abdominal pain (from splenic capsule or ovaries), and/or testicular or ovarian pain.

The "parental" WT1 specific $CD8^+$ T cell clone C4 that provided the TCR to be used in this trial was isolated from a healthy donor. Of all the clones we screened, the TCR from this clone had the highest affinity TCR found in any tested clone (lowest K_D value) as determined by tetramer titration. Even though this TCR was isolated from the repertoire of a healthy individual, it remains possible that patients who receive an infusion of virus-specific T cells transduced with the C4 TCR gene may potentially experience on-target organ toxicities, justifying the dose-escalation plan and the stopping rules described in the protocol.

WT1 expression in normal and malignant tissues is comparable between mice and humans. We have developed a mouse model in which on-target toxicities can be assessed using a murine WT1-specific TCR that has been mutated to have an affinity that is ~100-250 fold higher than

can be isolated from the normal repertoire. Mice injected with CD8⁺ T cells transduced with this mutated high affinity TCR did not demonstrate any toxicity in WT1 expressing organs, and the transduced T cells functioned normally *in vivo*, responded to immunization with WT1, and recognizing WT1⁺ tumor cells with no evidence of activation from cells. These results suggest that the risk of toxicities to WT1-expressing organs after infusion of T cells transduced with a WT1-specific TCR derived from a naturally occurring T cell clone may be low.

Acute GVHD can potentially be induced by alloreactive T cells or from the creation of new TCR specificities for host proteins in transduced T cells that result from mispairing of the endogenous α and β TCR chains with the introduced β or α chains. Laboratory studies have indicated that donor T cells that mediate acute GVHD are mainly found to have arisen from the T_N population (T_N) and not from the memory T cell pool, and removing T_N from the stem cell graft has been effective in preventing GVHD in rodent models.[85,94] This principle is now being tested in a clinical trial utilizing T_N depleted allogeneic peripheral blood cells in myeloablative HCT (**FHCRC Protocol 2222**).

By first enriching for EBV- or CMV-specific memory T cells for transduction with the WT1-specific TCR, most donor T cells that have either an alloreactive specificity or are T_N should be excluded, therefore reducing the probability of inducing acute GVHD from the T cell infusions. We have also demonstrated that, if the inserted TCR genes have been cys-modified, mismatching of the inserted modified TCR chains with the endogenous chains is minimal, with <5% of the introduced chains forming mismatched pairs.[66] The use of virus-specific T cells for transduction with WT1-specific TCR genes also limits the repertoire of endogenous TCR chains available to form mismatched pairs, further reducing the potential risk of the introduced α and β chains causing alloreactivity.

It is possible that toxicity from acquired alloreactivity, if it occurred, could present not only in organs usually affected in acute GVHD (e.g. skin, gut, and liver) but also potentially at unusual sites for GVHD due to alloreactive cells that have a unique specificity. However, of note, no toxicity from mismatched pairing has been described in human trials utilizing TCR transduced T cells, even though in the trials that have been performed the combined strategies discussed above that will be employed in our trial to reduce the risk were not employed, suggesting the risk from this mechanism should be very low.

IL-2 may also potentially increase the risk of acute GVHD. However, in **BB-IND 12046 - FHCRC Protocol 1655**, patients who received the same dosing of IL-2 as in this protocol have not experienced acute or worsening GVHD as a result of IL-2 therapy. Furthermore, IL-2, when given in 2-6 times higher doses than in the current protocol in association with DLI, did not increase the rate of acute GVHD compared to DLI without IL-2.[33] A recent study demonstrated that IL-2 administration to patients with steroid-refractory chronic GVHD was associated with preferential, sustained regulatory T cell expansion in vivo and amelioration of the manifestations of chronic GVHD in a substantial proportion of patients.[95] Therefore, based on our experience and the reported observations of others,[33,95] we do not expect an increased risk of GVHD due to the administration of low dose SC IL-2 in this study.

Antigen-specific CD8⁺ T cells targeting CMV pp65 epitopes generated using antigen-presenting cells have been isolated either from bone marrow donors and adoptively transferred to immunodeficient bone marrow transplant recipients, or isolated from and re-infused in HIV⁺ patients receiving antiviral therapy. No toxicities were observed in either setting and in a subset of patients, in whom the clones provided persistent reconstitution of CD8⁺ CMV-specific CTL

responses.[96,97] Donor-derived or autologous ex vivo expanded $CD8^+$ T cells targeting EBV have also been infused into patients with EBV-related lymphoproliferative disorders and similarly to CMV-specific $CD8^+$ T cells, no organ toxicities occurred in patients who experienced clinical benefit or with prolonged persistence of the infused cells.[98]
Another safety concern is the potential for transformation from insertional mutagenesis, which has been documented following integration of retroviral vectors into the host genome. This is well-documented in two X-linked SCID gene therapy trials in which five patients developed Acute Lymphoblastic Leukemia (ALL), which was shown to reflect transactivation of either the LMO2 or CCND2 gene by the retroviral insert in transduced $CD34^+$ hematopoietic stem cells (HSCs). However, retroviral transduction of HSCs represents a unique situation in which the retroviral vector preferentially inserts near genes that are actively expressed in stem cells, including genes that may confer a proliferative/survival advantage. These HSC and their lymphoid progeny then proliferate extensively, particularly in the context of having been transferred into an empty lymphoid compartment, allowing for rare pro-oncogenic integrations to yield very large numbers of progeny that are more susceptible to additional oncogenic events.

It has also been suggested that the development of leukemia in these patients may reflect in part the nature of the gene inserted and severity of disease, since none of >30 patients with SCID from adenosine deaminase (ADA) deficiency who received therapy with gene modified $CD34^+$ hematopoietic stem cells has experienced vector related toxicities.[99]

By contrast to these treatment settings, transferred T cells are more differentiated with less proliferative potential, and a review of 31 patients treated with more than 10^{11} retrovirally-transduced lymphocytes detected no adverse events from insertional mutagenesis.[100] Importantly, self-inactivating lentiviral vectors, in distinction to gamma-retroviral vectors, do not preferentially integrate near active promoters and have been shown to have a much lower risk of insertional mutagenesis.[101] However, these concerns further justify initially pursuing therapy with transduced T cells in patients with high risk or relapsed AML, MDS, or CML, as described in our plan, rather than patients with better prognosis disease.

The potential toxicities are listed in the protocol consent form, and will be discussed with patients as a part of consenting. If eligible patients elect to enroll on the protocol, they will be monitored and managed for potential toxicities as outlined in **Section 15** and **Section 19** and stopping rules applied as described in **Section 24**.

3.J. Rationale for Allowable Doses of Immunosuppressive Therapy During the Study

There are limited data examining the effects of pharmacological immunosuppression on the in vivo fate of donor-derived T cells, their function, and their antitumor efficacy.[102] Among the eleven patients who received WT1-specific T cell infusions on **BB-IND 12046 - FHCRC Protocol 1655**, four patients received corticosteroids (at doses up to prednisone 0.5 mg/kg/day) during the time of T cell infusions. Of those patients, two patients received tacrolimus, and one patient received Mycophenolate mofetil (MMF) at a dose of 1 gm twice daily. Prednisone and tacrolimus did not seem to affect the persistence of the WT1-specific T cells, as the patients who received these drugs demonstrated persistence of more than 14 days after administration of WT1-specific T cells at a dose of $\geq 3.3 \times 10^9$ cells/m^2, which was not different form the persistence demonstrated by patients who did not receive immunosuppression at the time of T cell infusion (see Patients #1, #21, #27 in **Table 3**). One patient who received MMF (2 gm/day) during the time of T cell infusion demonstrated persistence up to 14 days with an infused cell dose of 3.3×10^9 cells/m^2 (Patient #6 in **Table 3**). Based on the effects of

immunosuppressive medications on T cell persistence observed in our previous study, for the current study we will allow continuation of calcineurin inhibitors, corticosteroids (prednisone ≤0.5 mg/kg/day, or an equivalent dose of an alternative glucocorticoid), and low doses of MMF (≤1 gm/day).

As we expect the toxicities related to the WT1-specific T cell infusion to occur within 14 days, the low dose immunosuppressive drugs will not affect our ability to evaluate toxicity related to the infused cells.

Table 3. WT1 T cell persistence

Patient	INFUSION #1			INFUSION #2			INFUSION #3		
	Immuno-suppression	WT1 Cell Dose	Persistence (Days After Infusion)	Immuno-suppression	WT1 Cell Dose	Persistence (Days After Infusion)	Immuno-suppression	WT1 Cell Dose	Persistence (Days After Infusion)
**No IL-21 in Cell Culture**									
#1	Pred 45 mg QDAY<br>Tacro 1.5 mg BID	3.3x10^{8}/m^{2}	3	Pred taper<br>Tacro 1.5 mg BID	1x10^{9}/m^{2}	7	Pred 10 mg QDAY<br>Tacro 1.5 mg BID	3.3x10^{9}/m^{2}	14
#2	None	3.3x10^{8}/m^{2}	7	None	1x10^{9}/m^{2}	7	None	3.3x10^{9}/m^{2}	1
#6	MMF 1 gm BID<br>HC 30 mg/day	3.3x10^{8}/m^{2}	0	MMF 1 gm BID<br>HC 30 mg/day	1x10^{9}/m^{2}	1	MMF 1 gm BID<br>HC 30 mg/day	3.3x10^{9}/m^{2}	14
#10	None	3.3x10^{8}/m^{2}	0	None	1x10^{9}/m^{2}	1	None	3.3x10^{9}/m^{2}	1
#15	None	3.3x10^{9}/m^{2}	7	N/A			N/A		
#17	None	3.3x10^{9}/m^{2}	7	None	1x10^{10}/m^{2}	14	None	1x10^{10}/m^{2}<br>(+IL-2)	1
#20	None	3.3x10^{9}/m^{2}	1	None	1x10^{10}/m^{2}	1	N/A		
**IL-21 in Cell Culture**									
#21	Pred 15 mg QOD	3.3x10^{9}/m^{2}	14+	Pred 15 mg QOD	1x10^{10}/m^{2}	14+	Pred 15 mg QOD	1x10^{10}/m^{2}<br>(+IL-2)	427+
#24	None	3.3x10^{9}/m^{2}	14+	None	1x10^{10}/m^{2}	11	None	1x10^{10}/m^{2}<br>(+IL-2)	220+
#27	Pred 60 mg QDAY<br>Tacro 4 mg BID	3.3x10^{9}/m^{2}	14+	Off Pred<br>Tacro 4 mg BID	1x10^{10}/m^{2}	14+	Off Pred<br>Tacro 2 mg BID	1x10^{10}/m^{2}<br>(+IL-2)	160+
#28	None	3.3x10^{9}/m^{2}	14+	None	1x10^{10}/m^{2}	14+	None	1x10^{10}/m^{2}<br>(+IL-2)	84+

Acronyms: Pred = Prednisone, Tacro = tacrolimus, MMF = Mycophenolate mofetil, HC = hydrocortisone

3.K. Previous Human Experience with TCR_{C4}: BB-IND 12046 – FHCRC Protocol 1655: Phase I/II Study of Adoptive Immunotherapy with CD8+ WT1-specific CTL Clones for Patients with Advanced MDS, CML, AML, or ALL after Allogeneic Hematopoietic Stem Cell Transplant.

Initially, TCR_{C4} was evaluated in a prospective, single-arm clinical trial (**FHCRC protocol 1655**) to determine the safety and potential toxicities associated with infusing donor $CD8^+$ T cell clones specific for WT1 in patients. The secondary objectives were to assess in vivo persistence of transferred CTL, localization of transferred cells to bone marrow, and potential anti-leukemic activity. HLA-A*0201$^+$ patients at high risk for relapse and undergoing HLA-matched allogeneic HCT at the FHCRC for AML, ALL, MDS, and CML were eligible for enrollment and treatment. $CD8^+$ T cells from an EBV-seropositive healthy donor were stimulated with the HLA A*0201-restricted EBV_{BMFL1} peptide (GLCTLVAML) and lentivirally transduced to express the C4 TCR. The transduced cells were then selected based on binding both EBV and WT1 multimers and expanded using the rapid expansion method (REP) described in the accompanying **IND Section 7**.

Eleven patients received therapy on this protocol with three to five infusions of WT1-specific T cells (at doses of 3.3×10^9 to 1.0×10^{10} cells/m^2) generated and expanded from the patient's matched donor. The last infusion is followed by 5×10^5 U/m^2/day IL-2 for two weeks.No grade II-IV GVHD was attributed to the T cell therapy. With the exception of transient lymphopenia, which is consistently observed after antigen-specific CTL infusions,[90] only two cases of flu-like symptoms and one case of grade $\geq$3 fatigue were observed. The infused T cells were detectable in the blood and localized to the bone marrow without observed toxicities to WT1-expressing organs. A patient exhibited reduction in leukemic blasts after infusion of CTL (11.8% to 1.8% of PBMC). One patient, who entered HCT with >40% blasts in the bone marrow was treated prophylactically after HCT, and remained in CR at >22 months despite >80% risk of relapse. Three patients with relapsed leukemia after HCT were treated with salvage therapy and achieved remission, and then immediately received T cells, and remained in CR at >11, >13, and >21 months post therapy.

3.L. Ongoing Human Experience with TCR_{C4} in the current study (FHCRC protocol 2498 Study)

For the current trial, TCR_{C4} is being introduced into HLA-A*0201-restricted Epstein–Barr virus (EBV)- or cytomegalovirus (CMV)-specific donor cells. Initially, this protocol planned for each patient to receive a total of 4 infusions following a dose-escalation schedule: 1×10^9 cells/m^2, 3.3×10^9 cells/m^2, 1×10^{10} cells/m^2, and 1×10^{10} cells/m^2 with this last dose followed by low-dose subcutaneous (s.c.) Interleukin-2 (IL-2) at 2.5×10^5 IU BID x 14 days, administered to enhance the survival of transferred T cells.[103] The trial started out as a two-armed study, with patients who had no detectable disease after HCT treated on the 'Prophylactic Arm' (Arm 1). For safety concerns, the first 3-6 patients (Arm 1/Stage 1) were planned to receive infusions separated by a 28-day interval between infusions. If no unexpected toxicities were detected, the interval between infusions was planned to be reduced to 14 days (Arm 1/Stage 2). Patients with relapsed disease after HCT were planned to receive infusions on the 'Treatment Arm (Arm 2). The first 3-6 patients (Arm 2/Stage 1) were planned to receive the same infusion doses as Arm 1/Stage 1, but these were planned to be separated by a shorter interval to reach higher and potentially therapeutic doses in a more limited time. The initial treatment plan is shown in **Figure 1**, below.

Figure 1. Initial treatment plan of protocol 2498, Arms 1 and 2

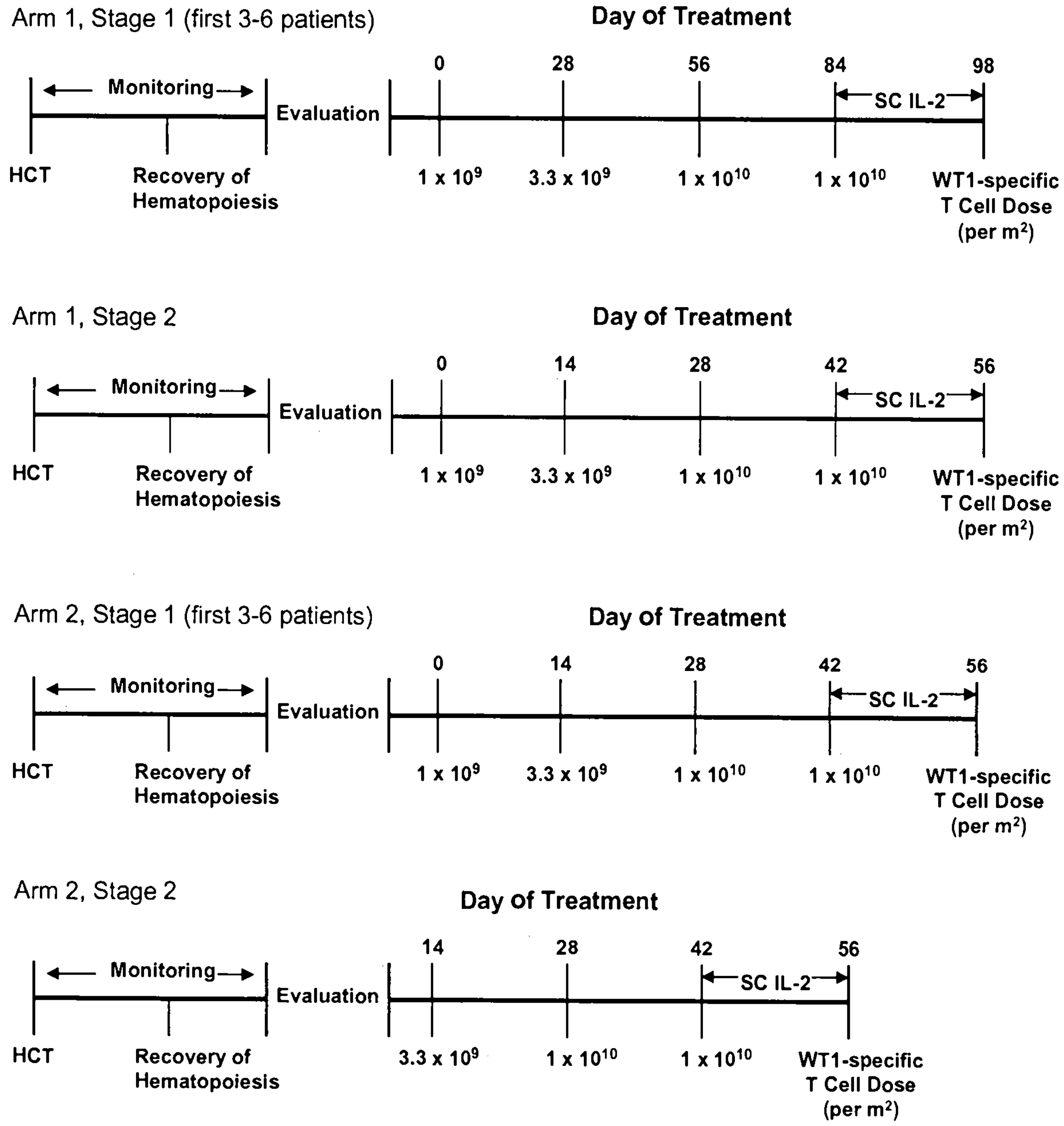

Because the dose-escalation schedule was well tolerated and no severe or unexpected toxicities were observed in the first 7 patients treated with up to 10^{10} TCR_{C4}-transduced cells/m^2, both the FDA and the FHCRC IRB approved discontinuation of the dose-escalating schedule in May 2014 such that all patients prospectively enrolled (irrespective of their disease burden post-transplant) have been receiving a first dose of 10^{10} cells/m^2 on day 0, and a second infusion of 10^{10} cells/m^2 at least 14 days after the first infusion, or later if the persistence of the cells from the initial infusion is >3% of total CD8+ cells. The second infusion is followed by 14 days of s.c. low-dose IL-2 (**Figure 2,** below).

Figure 2. Revised treatment plan of protocol 2498, Arms 1 and 2

Day of Treatment

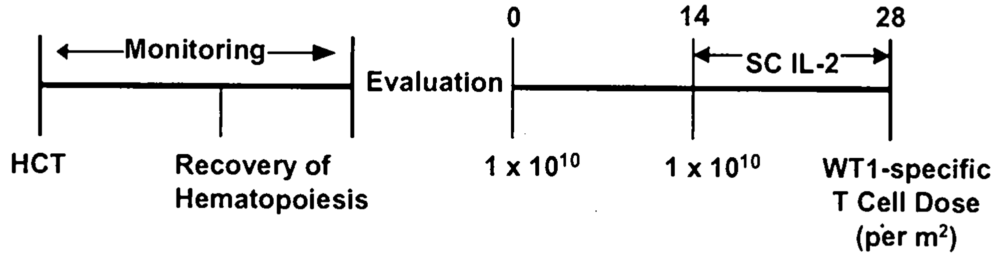

3.L.1. Patient characteristics on Protocol 2498

As of January 1, 2016, 25 patients with high-risk AML received a total of 48 doses of donor-derived virus-specific cells (**Tables 4A and 4B**). Thirteen patients received prophylactic treatment on Arm 1 and twelve patients were treated on Arm 2 due to detectable disease post-HCT. Overall, 33 of the 48 T-cell infusions represented the maximum target dose of 1×10^{10} WT1-specific CTL/m^2, and, of those, 11 infusions were followed by low-dose s.c. IL-2.

Table 4A. Patient characteristics on Protocol 2498, Arm 1

M = male; F = female; AML = acute myeloid leukemia; y = years; HCT = hematopoietic cell transplantation; CR = complete response; PB = peripheral blood; MRD= minimal residual disease

Pt.	Age/ Gender	Disease	EBV/ CMV	Arm	Comments
3	49 M	AML with complex cytogenetics, received HCT after 2nd CR	EBV	1	Poor T cell persistence, remains in remission 43 months after transplant
10	64 M	high-risk AML, received HCT in CR1. No evidence of disease after HCT.	EBV	1	Persistent T cells, in remission 30 months after HCT.
11	59 F	High risk AML, received HCT in CR1, No evidence of disease after HCT.	EBV	1	Persistent T cells, remains in remission 36 months after HCT.
12	55 F	HCT at CR1, high risk AML. No evidence of disease after HCT	EBV	1	Persistent T cells, remains in remission 33 months after transplant
13	59 F	high-risk AML, HCT in CR1, no evidence of disease after HCT	EBV	1	Persistent T cells, remains in remission 30 months after transplant
16	65 M	MDS-> AML, 5.5% blasts at HCT.	EBV	1	Poor T cell persistence, remains in remission 28 months after transplant
17	30 M	Extramedullary AML, MLL gene-rearrangement.	EBV	1	Poor T cell persistence, remains in remission 26 months after transplant
18	47 M	Relapsed AML in CR2	EBV	1	Persistent T cells, remains in remission 19 months after transplant
19	77 F	Relapsed AML in CR2	EBV	1	Persistent T cells, remains in remission 18 months after transplant
21	59 F	AML with complex cytogenetics, FLT3-ITD mutation.	EBV	1	Persistent T cells, remains in remission 13 months after transplant
22	30 M	AML with complex cytogenetics	EBV	1	Relapsed disease (extramedullary and cytogenetic relapse in marrow) at 8 months after transplant; did not receive planned T cell doses
24	57 M	AML in CR2; MRD at time of SCT	EBV	1	Persistent T cells, remains in remission 10 months after transplant
25	67 F	MDS -> AML, poor-risk cytogenetics	EBV	1	Persistent T cells, remains in remission 5 months after transplant

Table 4B. Patient characteristics on Protocol 2498, Arm 2
M = male; F = female; AML = acute myeloid leukemia; y = years; HCT = hematopoietic cell transplantation; CR = complete response; PB = peripheral blood; MRD= minimal residual disease

Pt.	Age/ Gender	Disease	EBV/ CMV	Arm	Comments
1	56 M	AML, relapse with para-spinal chloroma 5 years after 1st myeloablative HC	EBV	2	Disease progression. Removed from study before 4th infusion
2	51 F	AML, 2nd HCT for relapse 9 years after 1st myeloablative HCT. Entered HCT with 16% leukemia blasts and blasts were again detectable after 2nd HCT	CMV	2	Normalization of counts and decreased abnormal blast population to 0.006% after T cell infusions. Remains in remission.
4	25 M	AML, relapse with medullary and extra medullary disease after 2nd HCT	EBV	2	Patient remained in CR for 1 year after infusion, then relapsed with extramedullary disease when $WT1^{+}$ $CD8^{+}$ cells measured at >3% of total CD8 cells. Disease progressed despite 2nd infusion.
5	49 M	AML, disease progression after HCT (70% PB blasts at T cell infusion)	EBV	2	Disease progression. Off study at 6 days, and expired 12 days after 1st infusion.
6	20 F	AML, second HCT for relapse 2 years after 1st myeloablative HCT	CMV	2	Poor T cell persistence (0.04% at day 173), in CR 24 months after HCT.
7	33 F	AML, MRD after myeloablative HCT	EBV	2	Progressive disease. Received Azacitidine after 1 T cell infusion.
8	63 F	AML, received HCT in CR2. MRD early after transplant	EBV	2	Persistent T cells, disease progressed after infusion, and now receiving systemic therapy.
9	67 F	AML, received HCT in CR2. Relapse early after transplant	CMV	2	Poor T cell persistence, progressive disease.
14	17 M	AML, 2nd HCT for relapse 5 years after first HCT.	EBV	2	Disease progression despite persistent T cells; received azacitidine.
15	69 F	MDS -> AML, second HCT for relapse 1 year after 1st HCT	CMV	2	Disease progression despite persistent T cells.
20	5 F	Relapsed AML after transplant; active disease (chloromas); MLL gene-rearrangement.	EBV	2	Disease progression despite persistent T cells; receiving re-induction chemotherapy
23	75 M	Relapsed AML after transplant	EBV	2	CR at time of T cell infusion; continued complete remission

3.L.2. Persistence of TCR$_{C4}$-transduced cells on Protocol 2498

Patients who received cells generated from EBV-specific cells generally demonstrated a high-level of persistence. Of the 16 patients who received transduced EBV-specific cells and could be followed beyond 4 weeks after their latest infusion, 12 had persistent frequencies >3% (range 3-60% of CD8 T cells) for an average duration of 44 weeks (range 3-85 weeks) (**Figures 3A and 3B**, below) representing an average maximum of 215 cells/microliter (range 10-989 cells/microliter). The remaining 5 patients who received transduced EBV-specific cells exhibited frequencies <3% within 1-14 days. Patient 4, who received the lowest dose (1×10^9), had cells detectable in the blood at frequencies of 4% to 8% of total CD8$^+$ T cells until 14 months after infusion, with a decrease to <3% by 16 months after infusion. Until adequate long-term safety is formally demonstrated, patients cannot receive additional T cell infusions if their WT1-specific CD8$^+$ T cell frequency from the previous dose is ≥3% of total CD8$^+$ T cells. Therefore, at this time, Patients 10, 11, 12, 18, 19, 24 and 15 have not received a second infusion. Of the 4 pts (Pts 2, 6, 9 and 15) who received CMV-transduced cells, Pt 2 demonstrated persistence of the TCR$_{C4}$-transduced cells for 49 weeks after the final infusion at frequencies well below 1%. Pts 6 and 9 had no persistence beyond 4 and 2 weeks, respectively, after their last infusion. Pt 15, however, had persistence of the TCR$_{C4}$-transduced cells above 7%, with the last follow-up 68 days after infusion.

Figure 3A. Observed frequencies of infused WT1-specific T cells (% CD8+ cells) in Arm 1 patients.

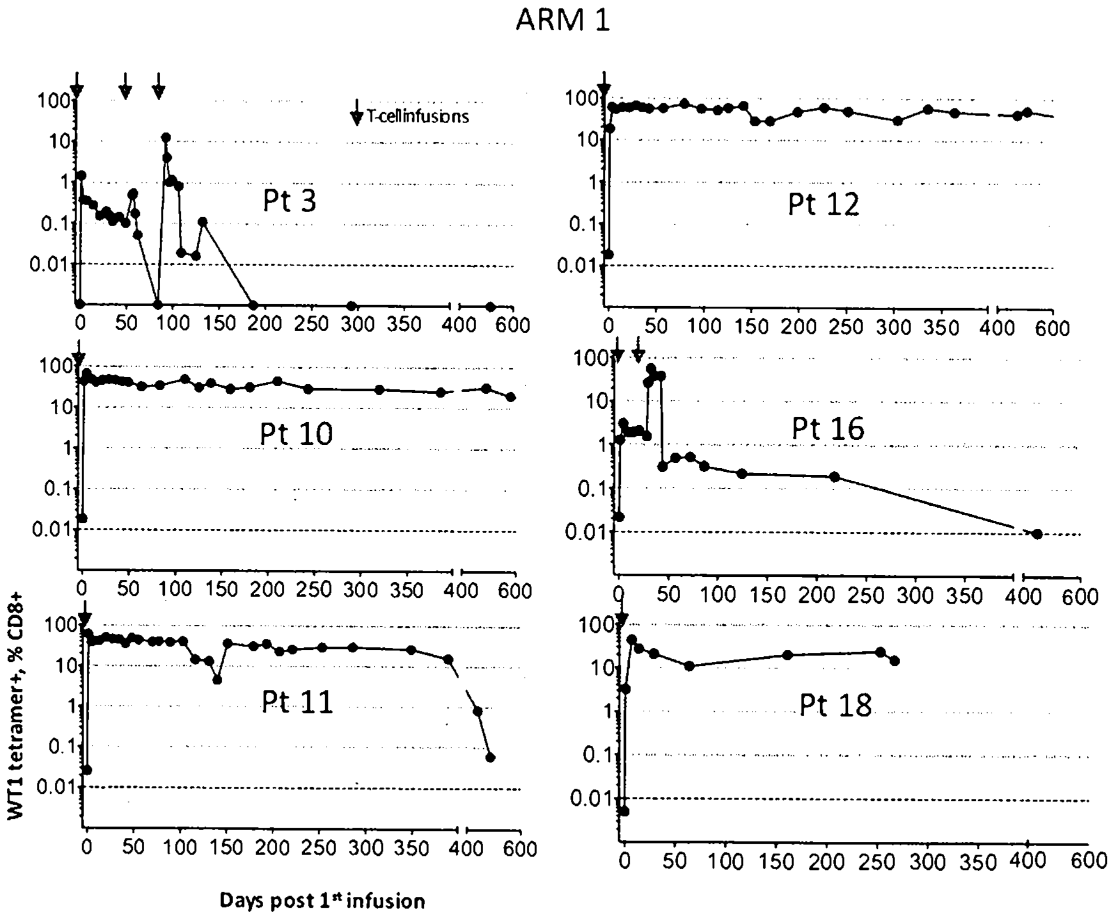

Figure 3B. Observed frequencies of infused WT1-specific T cells (% CD8+ cells) in Arm 2 patients.

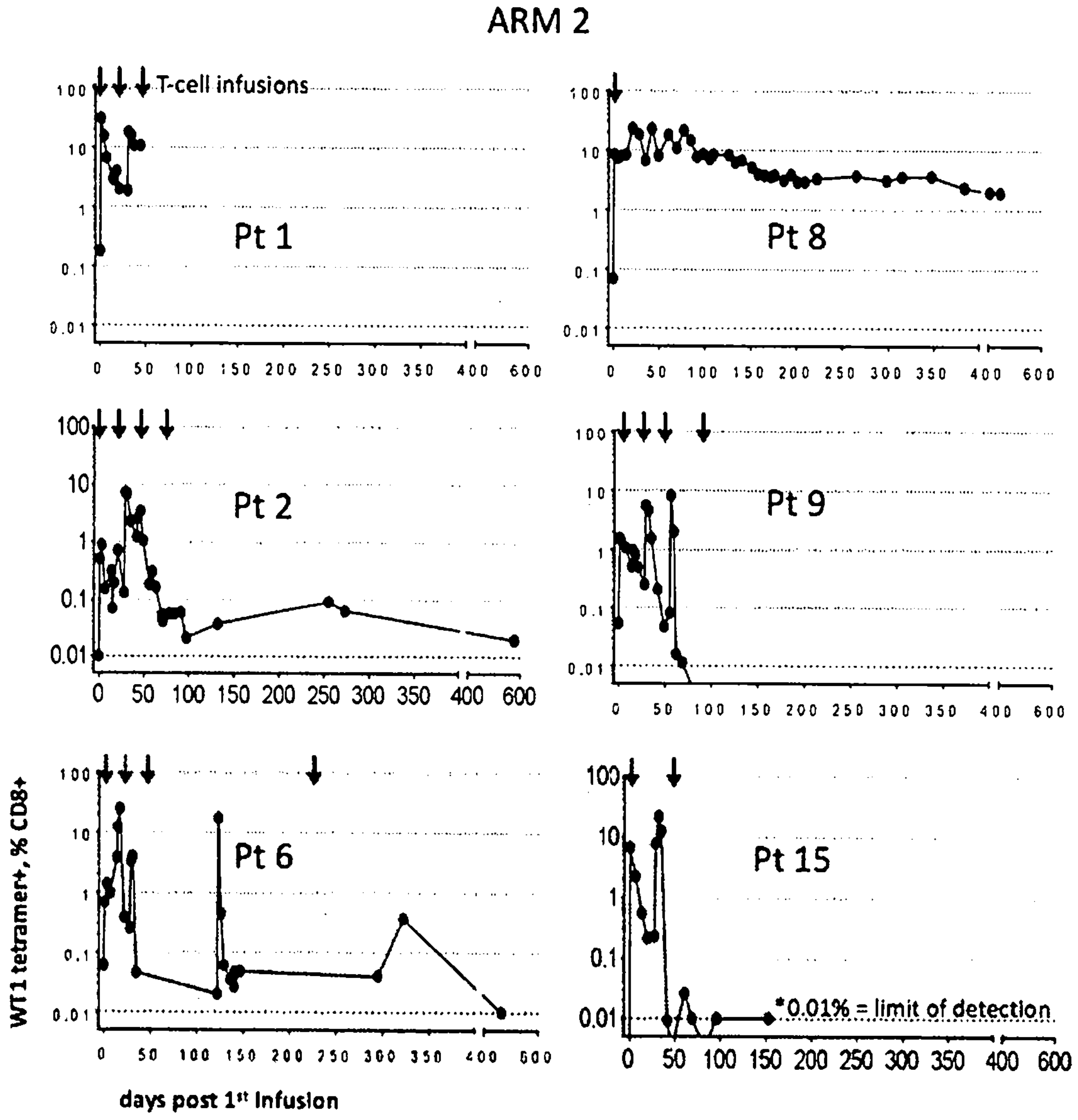

3.L.3. Toxicities observed on protocol #2498

All AEs were evaluated starting from the time of the first infusion to 30 days after the patients had taken the last dose of s.c. IL-2 for the first 7 patients then only AEs that were above or equal to a Grade 3 were recorded for subsequent patients. AEs that were deemed possibly, probably, or likely related were collected and graded according to the National Cancer Institute Common Terminology Criteria for Adverse Events version 4.0 (NCI CTCAE v4.0) (**Table 5**).

Table 5. Adverse events

Categories	NCI CTCAE v4.0	Grade 1	Grade 2	Grade 3	Grade 4
Cytokine Release Syndrome	Fever	4	3	2	
	Chills	2	1	1	
	Generalized aches/pain/headache	10	3		
	Fatigue	1	1		
	Tachypnea	4			
	Hypotension	5	2	4	
	Sinus tachycardia	3	2		
	Sinus bradycardia	1			
	Nausea	3	3		
	Vomiting	1			
	Diarrhea	1			
Hematological Abnormalities	Lymphopenia	3	5	8	1
	Anemia	1		3	
	Thrombocytopenia	1	2	3	
	Lymphocyte count increased		2		
Chemistry Abnormalities	ALT increased	6	2	2	
	AST increased	8		2	
	Alkaline phosphatase increased	4		1	
	Creatinine increased	3			
	Hypoalbuminemia	4			
	Hypocalcemia	7			
	Hypomagnesemia	1			
	Hyponatrema	5			
	Hypokalemia	3			
Miscellaneous	Dry eyes		2		
	Decreased respiratory rate	4			
	Maculopapular Rash			1	

Expected transient symptoms of **cytokine release syndrome** (CRS) were observed, as this syndrome is associated with activation of large numbers of antigen-specific CTL transferred into patients with targets expressing the antigen, or low-dose s.c. IL-2, or both. Specifically, 9 patients experienced fevers (≥38.3°C) with or without accompanying chills, including 2 patients with fever > 40.0°C following the infusion. Blood cultures were negative for bacterial or fungal growth in all cases. Many patients also experienced ≤grade 2 generalized aches, tachycardia or bradycardia, and digestive tract symptoms. With immediate management by administration of the antihistamine diphenhydramine and acetaminophen, plus the narcotic meperidine for chills, all symptoms resolved within 24 hours. The most severe CRS was observed in two patients who experienced transient grade 3 hypotension during the T cell infusion that rapidly responded to i.v. fluids. A grade 1 decrease in respiratory rate was observed in 2 patients during the 24 hours after the T cell infusions, but was associated with administration of meperidine for CRS. All side effects were managed in the outpatient General Clinical Research Center (GCRC) or on the general hospital ward without ICU support.

Importantly, we have observed only a single incident of *possible* on-target/off-tissue toxicity in all of the 25 patients who received WT1-specific T cell infusions. Specifically, patient 22 on Arm 1 developed left pleuritic chest pain in October 2016 that was attributed to pleuritis, but with no definitive underlying etiology. He did not have any detectable WT1-specific T cells in peripheral blood, making WT1-specific T cells unlikely to be the cause. However, because WT1 is expressed in the pleural tissue and without another identifiable cause, this SAE was reported. Notably, in the interim, the patient has developed frank leukemic recurrence with leukemic pleural effusion, making that the likely etiology in retrospect. Otherwise, there has not been any observed graft failure, renal failure, nephrotic-range proteinuria, pericarditis, or testicular or ovarian pain in any of the study cohort.

The **hematological abnormality** most commonly encountered was lymphopenia, which is a predictable, transient side effect of T cell infusions presumably reflecting redistribution of peripheral lymphocytes.[90,104] The temporary drop in total lymphocyte counts returned to pre-infusion levels within 7 to 11 days in all patients. Three cases of grade 3 anemia and 2 cases of lymphocytosis were observed during the time the patient was receiving T cell infusions, but did not occur immediately after infusions and the relationship to the T cell infusions remains unclear. Thrombocytopenia is very common in the first year after allogeneic transplant, [105] and to date all patients treated on this study started the T cell infusion with existing thrombocytopenia. Overall, we observed a general upward trend in platelet counts during infusions (average platelet counts before infusion: 78 000/µl, after infusions: 140 000/µl), but 3 of 6 evaluable patients had transient drops in platelet counts immediately after infusions most of which did not reach levels associated with a toxicity grade (**Figure 4**, below).

Grade 1 and 2 transient electrolyte and liver function abnormalities were also observed. One patient developed transient grade 2 transaminitis directly after the third T cell infusion, and this resolved without treatment within 7 days. The relationship of other observed metabolic abnormalities to the T cell infusions could not be excluded, but were clinically perceived to more likely reflect concurrent and common post-HCT etiologies, including side effects resulting from medications (e.g., electrolyte abnormalities are common side effects of calcineurin inhibitors and antifungals; elevated liver enzymes are a common side effect of antifungals), poor nutritional status (hypoalbuminemia), and secondary infections.

Figure 4. Kinetics of platelet counts after infusions (Pt. 2)

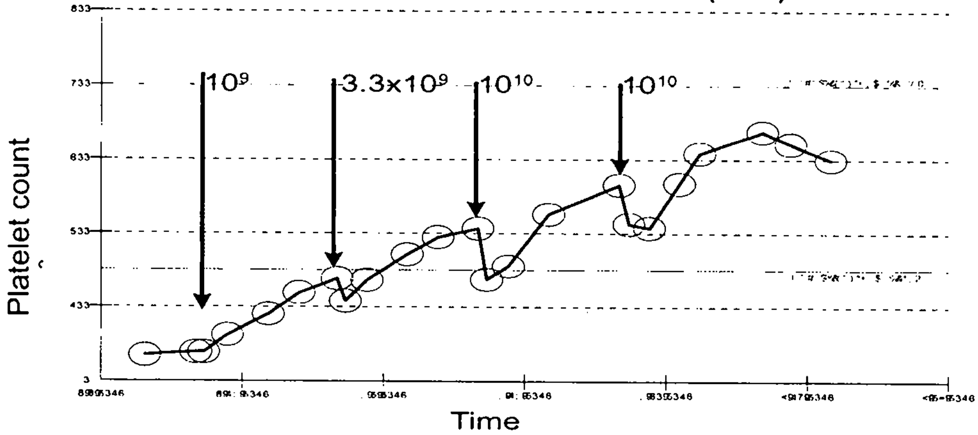

One patient (Pt 6) without any prior GHVD was diagnosed at 10 months after transplant, which was 4 months after her final WT1-specific T cell infusion, with grade III late acute GVHD affecting liver (stage III), GI tract and skin, as well as chronic GVHD involving oral mucosa and

eyes. Symptoms and transaminitis responded favorably to steroids. A second patient (Pt 13) was diagnosed 13 months after transplant, which was 10 months after her final WT1-specific T cell infusion, with grade III late acute GVHD affective liver (stage III). These are the only cases of grade III/IV GVHD observed in our study population, and notably WT1-specific T cells were near undetectable (0.04% of CD8+ cells) or undetectable, respectively, at the time of diagnosis making any association with the WT1-specific T cells unlikely.

One patient (Pt 16), with a prior history of acute GI GVHD, was diagnosed with chronic GVHD affecting liver (stage I, transaminitis only) and oral mucosa at 7 months after HCT, which was 3.5 months after WT1-specific T cell infusion. As above, WT1-specific T cells were undetectable at the time of the transaminitis, making any association with WT1- T cells unlikely. A patient (Pt 11) with a history of acute GVHD involving the skin and GI tract had a GVHD flare in the setting of tapering tacrolimus at 3 months following WT1-specific T cell infusion. Two patients (Pts 2 and 10) developed chronic GVHD after treatment with WT1-specific T cells, one of whom had a prior history of acute GVHD.

One patient (Pt 8) was diagnosed with acute GVHD affecting the skin and GI tract at 2.5 months after HCT, which was 7 days after receiving her first infusion of WT1-specific T cells. Symptoms were mild and responded to prednisone. This patient also had a prior history of cryptogenic organizing pneumonia (COP) occurring before transplant, and was diagnosed with a flare of COP at 6.5 months after HCT, which was 4 months after infusion of WT1-specific T cells. Initially responsive to steroids, she had a repeat flare of COP 4 months later while tapering prednisone, and required continued steroid therapy.

3.L.4. Efficacy observed on protocol #2498, Arm 1

Thirteen patients with high-risk leukemia entering HCT (**Table 6**, below), and who had no detectable disease after HCT, received WT1-specific cells within a median of 90 days post-HCT. With a maximum follow-up of 1,286 days and a median of 739 days after HCT, 12 of 13 patients are alive in CR and have not relapsed to date. A single patient (Pt 22) who *did not receive the intended dose of T-cells* due to logistical difficulty with generating his T cell product, subsequently relapsed with extramedullary disease in the mediastinum and also with evidence of cytogenetic relapse in the marrow. Progression-free survival is shown in **Figure 5**. For comparison, patients undergoing HCT with high-risk disease have an ~30% chance of relapse at 1 year. [25,27,28,106,107] This suggests the infusion of WT1-specific cells in the post-HCT setting may prevent leukemia recurrence.

Table 6. Cumulative risk factors for patients on Arm 1

Pt.	>CR1	Cytogenetics	refractory (>1cycle to achieve CR)	Disease at HCT	MDS->AML or secondary AML	Chloroma
3	X	X				
10		X	X			
11		X	X			
12		X (FLT3+)		MRD (cyto)		
13		X	X			
16				5.5% blasts	X	
17		X (MLL)				X
18	X					
19	X					
21		X (FLT3+)				
22			X			X
24		X			X	
25	X			X		

Figure 5: Progression-Free Survival of Arm 1 since HCT

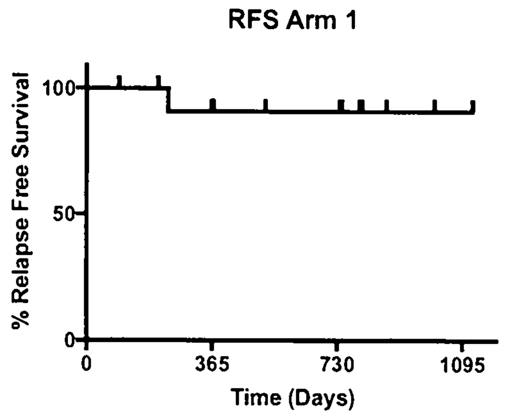

3.L.5. Efficacy observed on protocol #2498, Arm 2

Twelve patients with high-risk leukemia entering HCT with detectable disease after HCT had a higher cumulative risk based on adverse factors compared to patients treated on Arm 1 (**Table 7**, below). Patients received T cell infusions at a median of 121 days after HCT. Although 3 of 12 patients remain free of disease, with a median follow-up of 193 days after the first T cell infusion, 25% are still alive (**Figure 6**, below). For comparison, patients who relapse within 6 months after HCT have <10% chances of being alive at 1 year.[108] This suggests WT1-specific T-cells may confer a survival advantage despite the absence of complete clearance of relapsed AML post HCT.

Table 7. Cumulative risk factors for patients on Arm 2

Pt.	>CR1	Cyto-genetics	Refractory (>1cycle to achieve CR)	Disease at HCT	Secondary AML	Chloroma	2nd Transplant
1	X	X	X		X		
2	X	X	X	16% blasts			X
4	X	X	X	0.02% blasts		X	X
5	X	X	X	42% blasts	X		
6	X	X	X				X
7	X	X	X	3.8% by cyto			
8	X	X	X	0.01% blasts	X		
9	X	X		no counts			
14	X	X	X	0.02% blasts	X		X
15	X	X	X		X		
20	X	X				X	
23		X	X	X			

Figure 6: Overall survival of Arm 2 since 1st CTL infusion

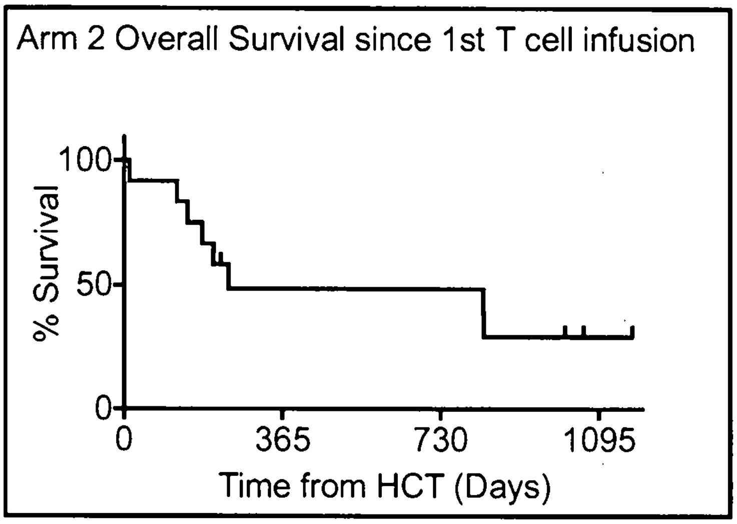

3.L.6. Protocol #2498: Conclusions to date

To date, WT1-specific T cells were detectable for a *cumulative* total of 471 weeks in 25 treated patients, and at frequencies reaching 60% of total CD8 and 998 cells/µl. No durable toxicities to tissues expressing physiological levels of WT1 (including cells of the hematopoietic, urogenital and renal systems, pleura, or pericardium) were detected during the monitoring period. There was a single cases of pleuritis (Pt 22) that in occurred in the absence of detectable WT1-specific T cells, and in retrospect was likely due to leukemic disease itself involving in the pleural space. Therefore, adoptive transfer of doses up to 1 × 10^{10} cells/m^2 of donor-derived virus-specific T cells transduced with the WT1-specific TCR_{C4} did not appear to injure normal tissues expressing physiologic levels of WT1, did not cause acute GVHD, and CRS symptoms induced by the infusion of high numbers of antigen-specific cells were readily managed in this preliminary series.

There is increasing evidence that WT1-specific T cells may protect against post-transplant leukemic relapse, as evidenced by the observed progression-free survival in Arm 1 of the study. Outcomes in Arm 2 of the study remain suboptimal, in terms of progression-free survival, survival and T cell persistence. Furthermore, most patients on the study have received engineered T cells while in clinical remission without detectable disease, limiting the study team's ability to directly observe anti-leukemic activity.

Thus, the study plan has been modified in an attempt to: 1) potentially improve outcomes in Arm 2 patients by the addition of lymphodepleting chemotherapy; and 2) increase the number of patients with AML and detectable disease at the time of T cell therapy.

3.M. Rationale for Lymphodepleting Chemotherapy Prior to T Cell Therapy

Transfer of T cells into lymphopenic hosts results in enhanced cell expansion of T cells with a memory phenotype and with enhanced effector function.[109,110] The favorable impact of lymphodepleting conditioning on survival and efficacy of transferred T cells has been observed in animal models,[111-114] donor lymphocyte infusions post-stem cell transplant,[115] and in numerous clinical trials with adoptive T cell transfer in melanoma and other cancers.[116-118] There are several proposed mechanisms by which lymphodepletion augments T cell expansion and function. One such explanation is that depletion of endogenous T cells minimizes T cell competition for homeostatic cytokines such as IL-7 and IL-15, as evidenced by the fact that proliferation is reduced in a dose-dependent manner when "irrelevant" T cells are infused along with a T cell population of interest.[119] Lymphodepleting chemotherapy may also significantly decrease $CD4^+$ $CD25^+$ regulatory T cells, which have been shown to suppress *in vitro* function of tumor-reactive T cells.[120,121]

This study protocol initially did not include a component of lymphodepleting chemotherapy, given the potential adverse effects of chemotherapy on engraftment in the early post-transplant period (particularly for patients receiving the intervention prophylactically). However, due to the lack of observed efficacy in Arm 2 subjects treated thus far, the very poor prognosis of post-transplant relapse, and the demonstrated safety with T-cells transduced to express TCR_{C4}, we believe the potential benefit of adding lymphodepleting chemotherapy warrants further study in this population with established post-transplant relapse (Arm 2). We anticipate that in vivo expansion and persistence may be improved following lymphodepletion, and that hopefully this will be accompanied by an improved anti-leukemic effect. Notably, for the proposed modification, a combination of cyclophosphamide and fludarabine will be employed, as has been demonstrated in several clinical trials, with proposed doses for our study in line with the

current CD19 CAR T-cell trial **FHCRC protocol 2639,** ongoing pediatric studies **(FHCRC/Seattle Children's PLAT-02 protocol)**, and **FHCRC protocol 9296**, in which patients with AML who are at high risk for relapse but who are not transplant candidates will receive autologous T-cells transduced to express TCR_{C4}.[115,116,122-124]

3.N. Rationale for Adjusting Eligibility Criteria for Ongoing Treatment on Protocol 2498
As of January 1, 2017, a total of 42 patients were enrolled on the current protocol. Of these, 25 patients were treated. Of the 42 enrolled patients, 40 patients had an underlying diagnosis of AML. Only 2 patients were enrolled with a diagnosis of MDS; of these, 1 opted out of treatment because of disease remission and 1 expired prior to treatment with transduced T-cells. The availability of competing study protocols has been identified as the major barrier of enrollment for MDS patients. No CML patients have been enrolled on the protocol, with poor accrual attributed to the very small number of patients with CML who undergo transplant in the present era of TKI therapy, compounded by the additional requirement of HLA*0201 expression in this already small target population. Thus, *only patients with AML* have been treated to date. Moving forward, given the poor accrual of MDS and CML patients thus far, it is not expected that continued efforts at enrolling and treating patients with MDS or CML will yield sufficient information as to justify the risk of added lymphodepleting chemotherapy.

On Arm 2 of the study, of 12 patients with post-transplant MRD or relapse who were treated on the study, an objective response was observed in one patient with MRD at the time of T-cell infusion, with subsequent clearance of disease; she remains in remission at 43 months after T cell infusion. Five patients with detectable disease at the time of T cell infusion demonstrated disease progression. However, 6 patients (50% of the Arm 2 cohort) were in CR at the time of initial T cell infusion – of which 2 remain in CR – while 4 patients relapsed/progressed. By definition, all Arm 1 patients were in CR at the time of treatment. Thus, most patients on the study have received engineered T cells while in clinical remission and without detectable disease, significantly limiting the study team's ability to measure anti-leukemic activity.

Thus, this protocol has been modified to limit prospective enrollment to patients with relapsed AML on Arm 2 of the study protocol, all of whom will be treated with lymphodepleting chemotherapy prior to WT1-specific T cells. Any patients previously enrolled on the protocol, but who have not completed the study intervention, will remain eligible to receive T-cells, as outlined in the originally approved plan of treatment.

4. OBJECTIVES

4.A. Primary Objectives

a) Determine the safety and potential toxicities associated with treating patients with high risk or relapsed AML, MDS, and CML after allogeneic HCT by adoptive transfer of virus-specific CD8 T cells genetically-modified to express a high affinity WT1-specific TCR.
b) Determine the anti-leukemic activity associated with treating patients with relapsed AML, MDS and CML after allogeneic HCT by adoptive transfer of virus-specific CD8 T cells genetically-modified to express a high affinity WT1-specific T cell receptor (TCR).

4.B. Secondary Objectives

a) Determine the in vivo persistence of transferred T cells and ability to migrate to and accumulate in bone marrow.

b) Determine the maintenance of TCR expression and function of transduced T cells.

5. STUDY ENDPOINTS

5.A. Primary Endpoints

a) Evidence and nature of toxicity related to study treatment.
b) Disease response when treating patients with active disease (MRD or overt disease).

5.B. Secondary Endpoints

a) Persistence and migration of transferred T cells to bone marrow.
b) Maintenance of TCR expression and function of transduced T cells.
c) Disease-free survival, relapse and time to progression after T cell therapy.

6. STUDY SCHEMA

The study schema is described below in **Figure 7**, representing the initial eligibility criteria and treatment plan. Note that prospective enrollment is now limited only to patients with post-transplant relapse of AML on Arm 2 of the study.

Figure 7. Study schema

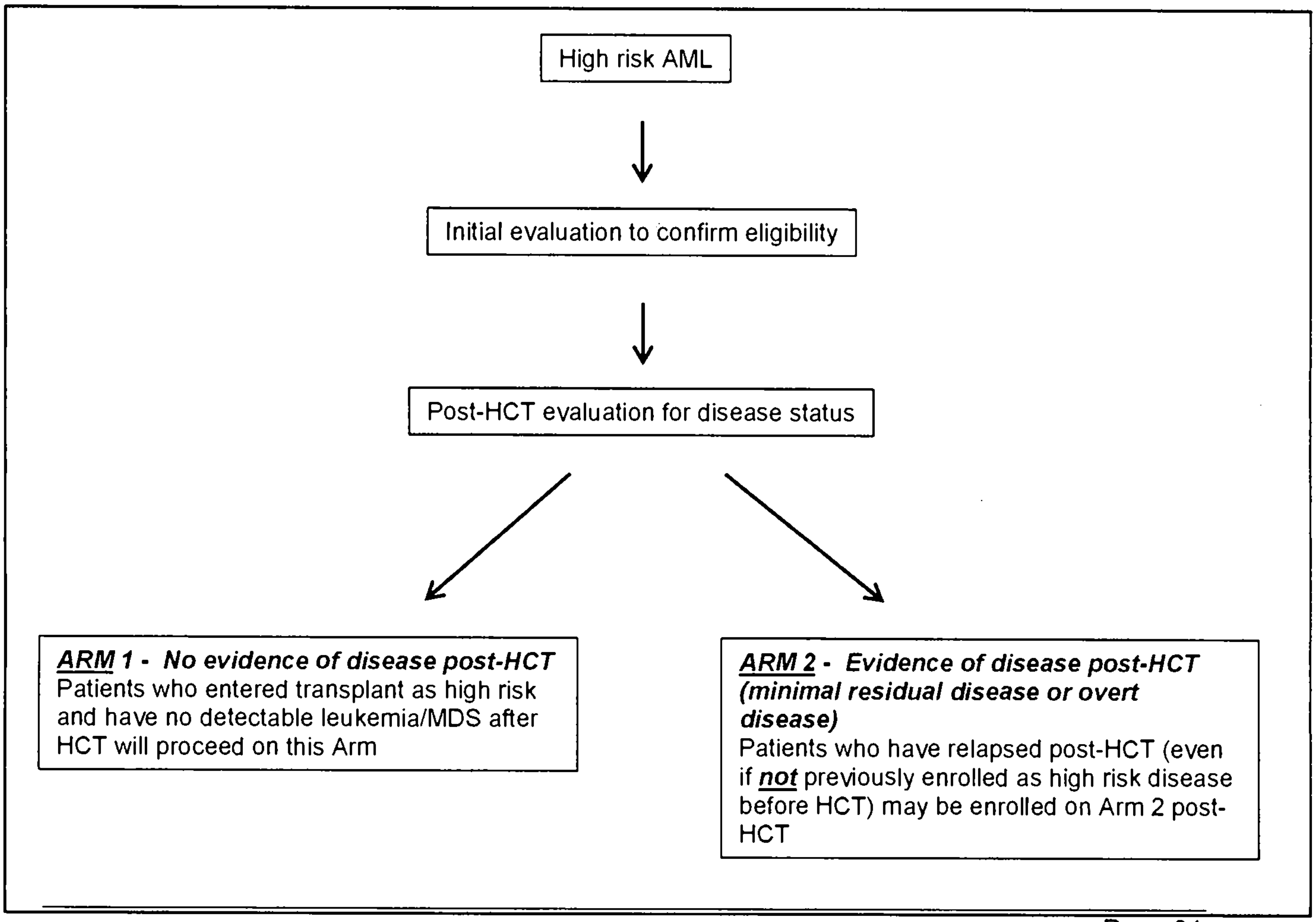

7. PATIENT SELECTION

7.A. Eligibility for Enrollment

1. Patients must express HLA-A*0201.

2. Patients who are currently undergoing or who previously underwent matched allogeneic HCT for:
   a. AML: Prospective enrollment will now be limited to patients with relapsed disease (overt relapse or minimal residual disease) at any time post allogeneic HCT.
   b. MDS will no longer be a criterion for eligibility.
   c. CML will no longer be a criterion for eligibility.

3. Patients must have an HLA-matched donor of hematopoietic stem cells (related or unrelated).

4. Patients must be able to provide blood and bone marrow samples and undergo the procedures required for this protocol.

5. Patients must be ≥15kg, as patients with lower weight would be incapable of providing high volume and frequent blood samples for monitoring and analysis.

6. Patients must be able to give informed consent. Parent or legal representative will be asked to consent for patients younger than 18 year old.

7.B. Exclusions for Enrollment

1. Central nervous system (CNS) tumor refractory to intrathecal chemotherapy and/or cranio-spinal radiation.

2. In patients whose leukemic cells are available for evaluation, the expression of WT1 in the patient's bone marrow will be determined. If WT1 expression in the patient's bone marrow is not highly expressed by PCR, the patient will be excluded from the study. Patients with no evaluable leukemia will be eligible for enrollment based on the high frequency of positive leukemias (>90%),[5,47] and leukemia will be evaluated for WT1 expression if recurrence is detected.

3. HIV seropositive. Testing for HIV should be within 6 months of enrollment.

4. Medical or psychological conditions that would make the patient unsuitable candidate for cell therapy at the discretion of the PI.

5. Pregnancy or breast-feeding. Women of childbearing potential must have a negative serum or urine β-hCG pregnancy test result within 14 days before the first dose of WT1-specific T cell infusion. Woman of non-childbearing potential will be defined as being postmenopausal greater than one year or who have had a bilateral tubal ligation or hysterectomy. All recipients of WT1-specific T cells will be counseled to use effective birth control during participation in this study and for 12 months after the last T cell infusion.

7.C. Definition of Relapse Status or MRD

1. Morphologic relapse defined as one or more of the following:
a. Abnormal blasts in peripheral blood in absence of growth factor therapy.
b. Abnormal blasts in bone marrow that represent >5% of nucleated cells.
c. Extramedullary chloroma or granulocytic sarcoma.

2. Flow cytometric relapse defined as:
a. The re-appearance in the peripheral blood or bone marrow of cells with an abnormal immunophenotype detectable by flow cytometry that is characteristic of the patient's leukemia.

3. Cytogenetic relapse defined as:
a. The appearance in one or more metaphases from bone marrow or peripheral blood cells of either a non-constitutional cytogenetic abnormality identified in at least one cytogenetic study performed prior to transplant or a new abnormality known to be associated with leukemia.

4. Molecular relapse defined as:
a. Positive PCR assay for the expression of genes associated with leukemia.

5. MRD defined as:
a. Flow cytometric, cytogenetic or molecular relapse without fulfilling the criteria for morphologic relapse.

8. DONOR SELECTION

8.A. Inclusions

1. Patient and donor (related or unrelated) must be HLA-matched and express HLA-A*0201.

a) Donor must be EBV or CMV seropositive.

b) Donor must be age 18 or older.

c) In good general health.

d) Able to give informed consent.

8.B. Exclusions

1. Less than 18 years old.
2. Active infectious hepatitis.
3. HIV or HTLV seropositive.
4. Pregnancy or nursing.

5. Significant medical conditions (e.g. immunosuppressive therapy) that would make the donor an unsuitable T cell donor.

6. Unable to give informed consent.

9. CONSENTING

An enrollment conference will be held with the patient, and the related donor (unrelated donors are consented through the National Marrow Donor Program). The PI or a delegated representative will discuss this study and alternative treatments available for high risk and relapsed AML. All known risks and potential hazards of treatment with virus-specific $CD8^+$ T cells transduced to express a WT1-specific TCR will be discussed. Informed consent will be obtained from the patient using forms approved by the Institutional Review Board (IRB) of the FHCRC.

Patients with high risk disease may be consented and enrolled on the study prior to transplant. Patients with high risk disease who met study criteria before transplant but had not been enrolled pre-transplant, may be enrolled and consented after transplant. In addition, any patient who experiences relapse post-HLA matched HCT (MRD or overt relapse) will be eligible to enroll in the study post-HCT.

10. PROTOCOL REGISTRATION

Patients will be assigned to the protocol by the Clinical Coordinator who will register the patient with the Registration Office, (206) 667-4728, between 8:30 am and 4:00 pm, Monday through Friday. After hours, the Registration Office can be reached by paging (206) 995-7437.

11. PROCEDURE TO OBTAIN PBMC FOR GENERATION OF WT1-SPECIFIC T CELLS

The preferred source for EBV/CMV-specific $CD8^+$ T cells isolation is the GCSF-mobilized PBSC product. If insufficient peripheral blood mononuclear cell (PBMC) product is obtained from the PBSC product, or if the patient is enrolled on study after PBSC collection and no cryopreserved PBSC product can be used, a separate leukapheresis or high volume peripheral blood draw should be performed as described below.

1. The G-CSF-mobilized PBSC product will be the preferred source for EBV/CMV-specific $CD8^+$ T cells isolation, since it will not require the donor to undergo an additional procedure. 5% of the PBSC product will be removed for the purpose of generating WT1-specific T cells. If insufficient PBMC product is obtained from the PBSC product, an additional day of 6 or 12 liter leukapheresis may be added, at the discretion of the PI and the attending physician. No additional G-CSF will be given after completion of stem cell collection.

2. If the patient is enrolled on study after PBSC collection, an attempt will be made to use a cryopreserved PBSC product, if available.

3. If fresh or cryopreserved PBSC product cannot be used for T cell isolation or inadequate EBV/CMV-specific $CD8^+$ T cells were isolated from the PBSC product, a separate 6-12 liter leukapheresis via intravenous access will be scheduled.

2. Donors must be age 18 or older and meet all the donor criteria for leukapheresis as described in **Protocol Appendix C** in order to undergo leukapheresis.
3. Donors will not be considered for leukapheresis if they have a medical condition precluding leukapheresis, but may undergo leukapheresis at a later time if condition resolves. Exclusions include:
   i. Infection, with or without antibiotic treatment.
   ii. Recent hepatitis exposure, hepatitis A or B antigenemia, or hepatitis C antibody positivity.
   iii. Pregnancy or nursing.
   iv. HIV or HTLV infection.

4. If a portion of the PBSC product cannot be used for T cell selection, and the donor is unable or unwilling to undergo separate leukapheresis, 400 mL of peripheral blood can alternatively be drawn for generation of the T cells. This is a non-preferred option as the total number of $CD8^+$ T cells is significantly lower than from leukapheresis or stem cell collection, and will require additional expansion of the transduced cells to achieve the therapeutic dose.

12. GENERATION OF WT1-SPECIFIC T CELLS

Donor peripheral blood lymphocytes will be enriched for virus-specific $CD8^+$ T cells (preferably EBV because of the high frequency of T_{CM}, but alternatively CMV) using clinical grade reagents according to Standard Operating Procedures (SOPs) in the Cell Processing Facility (CPF). The methods employed to enrich for virus-specific $CD8^+$ T cells and generate WT1-specific T cells are described in details in **IND Section 7 – Chemistry, Manufacturing and Controls**.

13. T CELL PRODUCT HANDLING PRIOR TO INFUSION

For each infusion dose, the gene-modified cell product is formulated at the desired cell dose in a final volume of 250ml (final volume may be less for patients who are 15-30kg). The final product will be prepared and labeled according to SOPs in the CPF. After releasing from the Cell Processing Facility (CPF), the cell product will be transported to the infusion facility by a protocol delegated staff member. During the time of transportation the cell product will be kept in a cooler with a cool pack. A nurse will then administer the cells to the patient over approximately 45 minutes (+/- 15minutes) (or longer for patients who are 15-30kg and if clinically indicated, as described in **Section 14** below).

14. PLAN OF TREATMENT

14.A. Plan of Treatment – Arm 1

Two T cell infusions at a dose of $1x10^{10}$ cells/m^2 will be infused at an interval of at least 14 days (**Figure 8**). First infusion may be given as soon as $CD8^+$ WT1-specific transduced T cells have been generated. The second infusion will be followed by IL2 injections as discussed below (**Section 14A.c.4**). To limit the potential risk of toxicity second infusions of WT1-specific T cells will be delayed beyond 14 days until the frequency of WT1-specific T cells is $\leq$3% of $CD8^+$ T cells. Second infusion may also be delayed due to the patient's clinical status per the PI and the attending physician discretion.

Figure 8. Treatment schema – Arm 1

Day of Treatment

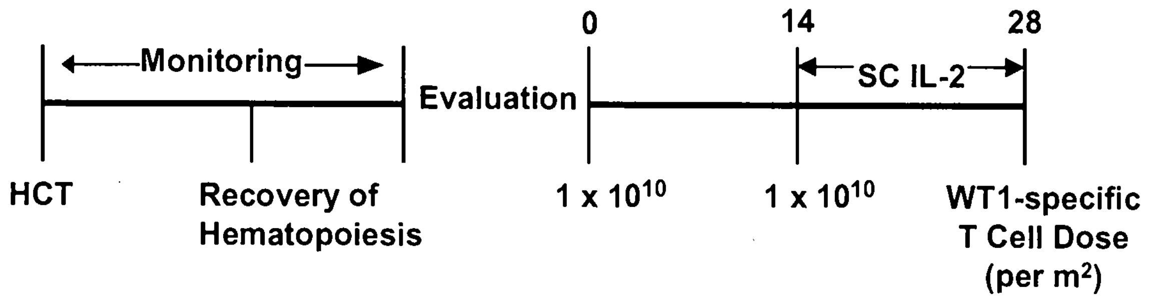

A) Eligibility for Treatment on Arm 1

1. **Disease status:** Patients must have no evidence of leukemia after transplant. Patients who have evidence of leukemia (MRD or overt relapse) prior to initiation of WT1-specific T cell therapy will be eligible for treatment on Arm 2. See **Section 7.C** for definition of relapse or MRD.

2. **Timing:** Patients can be treated post-HCT as soon as CD8+ WT1-specific transduced T cells have been generated, expanded, and completed Quality Control (QC) testing.

3. Patients should have evidence of post-transplant recovery of hematopoiesis (absolute neutrophil count [ANC] ≥1000/µl and platelets ≥50,000/µl for at least one week) prior to the initiation of T cell infusions.

4. Patients on immunosuppressive therapy for GVHD at the time of therapy are eligible for treatment if: (i) the patient has not developed grade III-IV acute GVHD prior to T cell therapy (**Protocol Appendix A**), (ii) the patient is not receiving corticosteroids, or if the dose of corticosteroids has been tapered to ≤0.5 mg/kg/day of prednisone (or an equivalent dose of an alternative glucocorticoid), and patients symptoms have been stable or improving for at least 14 days on tapering of immunosuppressive therapy, (iii) if patient is receiving MMF; MMF should be tapered to ≤1 gm/day prior to starting a T cell infusion or preferably be discontinued, particularly prior to the last infusion, which is followed by IL-2 administration, as MMF can lead rapid cell death of activated proliferating T cells.

B) Exclusions for Treatment on Arm 1

1. Patients who have evidence of leukemia (MRD or overt relapse) prior to initiation of WT1-specific T cell therapy (these patients will be eligible for treatment on Arm 2).

2. Patients with grade III or IV acute GVHD at any time prior to study therapy (**Protocol Appendix A**).

3. Anti-thymocyte globulin or other T cell suppressive treatment (such as anti-CD3 mAb) within one month prior to scheduled T cell infusion.

4. Prednisone >0.5 mg/kg/day (or an equivalent dose of an alternative glucocorticoid).

5. MMF should be tapered to ≤1 gm/day prior to starting T cell infusion or preferably be discontinued, particularly prior to the last infusion, which is followed by IL-2 administration, as MMF can lead to rapid cell death of activated proliferating T cells. Concurrent use of calcineurin inhibitors (cyclosporine, FK506) is not strictly an exclusion criterion, although attempts should be made to discontinue or reduce dosages of such drugs if possible.

6. Graft rejection or graft failure.

7. Karnofsky performance status score (age ≥16 years) or Lansky play score (age <16 years) ≤40%.

8. Unable to generate adequate numbers of $CD8^+$ WT1-specific transduced T cells from virus-specific T cells that were derived from the patient's matched donor.

9. Ongoing ≥grade 3 cardiac, pulmonary, renal, gastrointestinal or hepatic toxicities according to NCI CTCAE version 4 toxicity criteria.

10. Neurologic: Encephalopathy.

11. Any condition or organ toxicity that is deemed by the principle investigator or the attending physician to place the patient at unacceptable risk for treatment on the protocol.

12. Pregnancy or breast-feeding.

C) Administration of Therapy on Arm 1

Patients will receive two intravenous infusions of $CD8^+$ WT1-specific transduced T cells if no serious infusion related toxicities are observed (see **Section 18**). Cell doses for infusions will be based on patient's height and weight prior to first infusion (baseline); if time interval between infusions is greater than 60 days, or if the weight prior to subsequent infusion has changed by >20% compared to baseline weight, the subsequent cell dose will be calculated on the new weight. Actual cell doses administered may be less than the planned cell doses if insufficient numbers of transduced cells are generated. The interval between T cell infusions may be prolonged if deemed clinically to be in the best interest of the patient due to ongoing events.

Table 8. Schedule of WT1-specific T cell infusions on Arm 1

	**Infusion #1**	**Infusion #2**
**Treatment day***	0	14
**Cell Dose (WT1-specific transduced T cells)**	$1.0 \times 10^{10}/m^2$	$1.0 \times 10^{10}/m^2$
**IL-2**		2.5×10^5 U/m² every 12 hours for 14 days

* If indicated, second infusions will be delayed (**see Section 14.A.c.3**).

1. First infusion of CD8⁺ WT1-specific transduced T cells; Day 0

Patients will receive the infusion of T cells at the Seattle Cancer Care Alliance, either on the Immunotherapy service or alternatively in the infusion room. Patients admitted at the University of Washington Medical Center at the time of planned infusion will receive the T cells as an inpatient. The infusion will consist of $CD8^+$ WT1-specific transduced T cells at a planned cell dose of **1.0×10^{10} cells/m²** administered by intravenous (IV) infusion over approximately 45 minutes (+/- 15minutes) or longer for patients who are 15-30kg. Infusion time may be longer if required based on CPF endotoxin results or patient reaction to infusion. Vital signs and O_2 saturation should be monitored and recorded at around the following time points time 0, every 15 minutes during T cell infusion, and then hourly for 2 hours following the T cell infusion (see **Section 15.E**). Infusion-related toxicities, if observed, will be treated as outlined in **Section 18.**

2. Second infusion of $CD8^+$ WT1-specific T cells; Day +14

Patients may receive a second infusion of $CD8^+$ WT1-specific transduced T cells at a dose of **1×10^{10} cell/m²** at least 14 days after the first infusion if the patient meets persistence criteria and did not experience a toxicity requiring delaying/discontinuation of study treatment (see **Section 19**). Monitoring, and management of toxicity will be performed as described for the first infusion.

Several study participants have received T cells at doses lower than the current dose of 1.0×10^{10} cells/m², prior to the protocol being modified as such, and are awaiting subsequent infusion of T cells at the current (higher) dose. Such patients who go on to receive a subsequent dose of 1.0×10^{10} cells/m² shall be considered to have completed the study treatment, and are therefore eligible to receive IL-2, as outlined below.

3. Criteria for delaying subsequent T cell infusion

Subsequent T cell infusion will be delayed if clinically indicated, or if WT1-specific T cell frequency in the peripheral blood is >3% of total $CD8^+$ T cells. Up to 60mL (45mL for patients who are 30-50kg, 1ml/kg for patients who are 15-30kg) of blood should be drawn within 21 days prior to the scheduled date for the subsequent T cell infusion for evaluation of persistence of adoptively transferred T cells. If the WT1-specific T cell frequency is >3%, the following T cell infusion will be held. Repeat evaluation of persistence of WT1-specific T cells should be performed up to twice weekly for one month and then at the PI discretion until the WT1-specific cell frequency is ≤3%. Subsequent T cell infusion may then be administered as soon as T cell product and an infusion appointment are available.

4. IL-2 administration

If the second T cell infusion is tolerated without serious toxicity as detailed in **Section 18**, patients should then receive twice daily SC injections of recombinant IL-2 at a dose of 2.5 x 10^5 U/m^2 approximately every 12 hours for 14 days, starting between 2-4 hours after completing the T cell infusion. Patients or caregivers will be trained on IL-2 administration. IL-2 therapy may be omitted in any patient with a contraindication to receiving the drug (e.g. graft versus host disease), as determined by the PI and/or treating physician. IL-2 therapy may be discontinued in any patient developing grade 3 or greater treatment-related toxicities while receiving daily SC IL-2 injections.

14.B. Plan of Treatment – Arm 2

Patients with history of AML and evidence of disease relapse or MRD post-transplant will be eligible for treatment on Arm 2 of the study. While T cells are being generated, it is anticipated that debulking systemic therapy will be administered for patients with overt relapse. Morphologic remission will not be required to receive the study treatment (lymphodepleting chemotherapy and WT1-specific T cells), although additional systemic therapy may be administered prior to T cells depending on patient-specific parameters (e.g. tempo of disease relapse and/or disease burden) and the preference of the patient's treating physician. For patients with MRD only post-transplant, it is preferred that systemic therapy not be administered prior to the study intervention, in order to maximize the number of patients with detectable disease at the time of T cell therapy.

Two T cell infusions at a dose of $1x10^{10}$ cells/m^2 will be infused at an interval of at least 21 days (**Figure 9**). The interval was extended from 14 to 21+ days due to the addition of lymphodepleting chemotherapy and allowance for recovery of blood counts between infusions. The first infusion may be given on or after day +60 post-transplant when $CD8^+$ WT1-specific transduced T cells have been generated. The second infusion will be followed by IL2 injections, as discussed further below (**Section 14B.d.4**). To limit the potential risk of toxicity second infusions of WT1-specific T cells will be delayed beyond 21 days until the frequency of WT1-specific T cells is ≤3% of $CD8^+$ T cells. Second infusion may also be delayed due to the patient's clinical status per the PI and the attending physician discretion.

Patients previously enrolled on Arm 2 who have not yet received both T cell infusions will complete the planned study treatment by proceeding with the 2nd T cell infusion but without lymphodepleting chemotherapy.

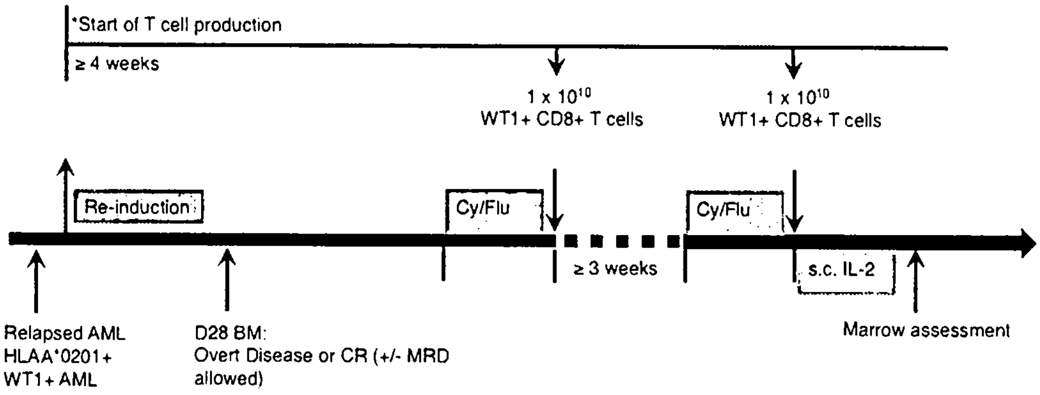

Figure 9. Revised treatment schema – Arm 2

A) Eligibility for Treatment on Arm 2

1. Disease status: Patients must have evidence of AML (MRD or overt relapse) post HCT. See **Section 7.C** for definition of relapse or MRD.

2. Timing: Patients can be treated post-HCT when $CD8^+$ WT1-specific transduced T cells have been generated, expanded, and completed QC testing. In addition, because of the potential negative impact of lymphodepleting chemotherapy on engraftment, patients must also be at least 60 days post-transplant to begin treatment.

3. Patients should have evidence of post-transplant and/or post-chemotherapy recovery of hematopoiesis (ANC ≥500/µl and platelets ≥30,000/µl for at least one week) prior to the initiation of T cell infusions. However, patients who have ANC <500/µl and/or platelets <30,000/µl due to underlying leukemia or lasting more than 6 weeks following systemic chemotherapy will be allowed to undergo therapy, but marrow suppression will not be evaluated as a stopping criteria in these patients.

4. Patients will receive two intravenous infusions of $CD8^+$ WT1-specific transduced T cells if no serious infusion related toxicities are observed (see **Section 18**). Cell doses for infusions will be based on patient's height and weight prior to first infusion (baseline); if time interval between infusions is greater than 60 days, or if the weight prior to subsequent infusion has changed by >20% compared to baseline weight, the subsequent cell dose will be calculated on the new weight. Actual cell doses administered may be less than the planned cell doses if insufficient numbers of transduced cells are generated. The interval between T cell infusions may be prolonged if deemed clinically to be in the best interest of the patient due to ongoing events.

5. Patients on immunosuppressive therapy for GVHD at the time of therapy are eligible for treatment if: (i) the patient has not developed grade III-IV acute GVHD prior to T cell therapy (**Appendix A**), (ii) the patient is not receiving corticosteroids, or if the dose of corticosteroids has been tapered to <0.5 mg/kg/day of prednisone (or an equivalent dose of an alternative glucocorticoid), and patients symptoms have been stable or improving for at least 14 days on

tapering of immunosuppressive therapy, (iii) if patient is receiving MMF; MMF should be tapered to ≤1 gm/day prior to starting T cell infusion or preferably be discontinued, particularly prior to the last infusion, which is followed by IL-2 administration, as MMF can lead rapid cell death of activated proliferating T cells.

B) Exclusions for Treatment on Arm 2

1. Patients with grade III or IV acute GVHD at any time prior to study therapy (**Appendix A**).

2. Anti-thymocyte globulin or other T cell suppressive treatment (such as anti-CD3 mAb) within one month prior to scheduled T cell infusion.

3. Prednisone >0.5 mg/kg/day (or an equivalent dose of an alternative glucocorticoid).

4. MMF should be tapered to ≤1 gm/day prior to starting T cell infusions or preferably be discontinued, particularly prior to the last infusion, which is followed by IL-2 administration, as MMF can lead to rapid cell death of activated proliferating T cells. Concurrent use of calcineurin inhibitors (cyclosporine, FK506) is not strictly an exclusion criterion, although attempts should be made to discontinue or reduce dosages of such drugs if possible.

5. Graft rejection or graft failure.

6. Patients with Karnofsky performance status score (age ≥16 years) or Lansky play score (age <16 years) ≤30%.

7. Unable to generate adequate numbers of $CD8^+$ WT1-specific transduced T cells from virus-specific T cells that were derived from the patient's matched donor.

8. Ongoing ≥grade 3 cardiac, pulmonary, renal, gastrointestinal, or hepatic toxicities according to NCI CTCAE version 4 toxicity criteria.

9. Neurologic: Encephalopathy.

11. Any condition or organ toxicity that is deemed by the principle investigator or the attending physician to place the patient at unacceptable risk for treatment on the protocol.

12. Pregnancy or breast-feeding.

C) Cytoreductive Therapy Prior to T Cell Infusion on Arm 2

It is expected that some patients (e.g. those with >5% bone marrow leukemic blasts at diagnosis of relapse) will receive cytoreductive therapy prior to T cell infusion, with such treatment decisions at the discretion of the treating physician. These patients will require a repeat bone marrow sampling prior to T cell therapy to document disease and marrow status. Patients with disease relapse who receive cytoreductive chemotherapy will begin treatment with $CD8^+$ WT1-specific transduced T cells after evidence of recovery of hematopoiesis (ANC ≥ 500/µl and platelets ≥30,000/µl), or at least 6 weeks after chemotherapy if neutrophil and/or platelet counts remain below threshold, given the risk of further increase in disease burden with prolonged delay in treatment. For patients with MRD only post-transplant, it is preferred that

systemic therapy not be administered prior to the study intervention, in order to maximize the number of patients with detectable disease at the time of T cell therapy.

D) Administration of Study Treatment on Arm 2

Patients will receive two intravenous infusions of $CD8^+$ WT1-specific transduced T cells if no serious infusion related toxicities are observed (see **Section 18**). Cell doses for infusions will be based on patient's height and weight prior to first infusion (baseline); if time interval between infusions is greater than 60 days, or if the weight prior to subsequent infusion has changed by >20% compared to baseline weight, the subsequent cell dose will be calculated on the new weight. Actual cell doses administered may be less than the planned cell doses if insufficient numbers of transduced cells are generated. The interval between T cell infusions may be prolonged if deemed clinically to be in the best interest of the patient due to ongoing events.

Table 9. Schedule of WT1-specific T cell infusions on Arm 1

	Infusion #1	Infusion #2
**Treatment day***	0	21+*
**cyclophosphamide / fludarabine**	Yes	Yes
**Cell Dose (WT1-specific transduced T cells)**	$1.0 \times 10^{10}/m^2$	$1.0 \times 10^{10}/m^2$
**IL-2**		2.5×10^5 U/m^2 every 12 hours for 14 days

* Timing of second infusions may be delayed beyond 21 days (**see Section 14B.d.3**).

1. <u>First infusion of $CD8^+$ WT1-specific transduced T cells; Day 0</u>

Patients will receive lymphodepleting chemotherapy prior to receiving donor-derived $CD8^+$ T cells transduced with TCR_{C4}. The suggested lymphodepletion regimen will consist of cyclophosphamide (300 mg/m2) and fludarabine (30 mg/m^2) daily for 3 days (days -4 to -2). Pediatric patients meeting treatment eligibility will receive chemotherapy and T cells under the continued care of the pediatric Hematology/Oncology service at Seattle Children's (Ambulatory Nursing Infusion Unit). It is planned that adult patients meeting treatment eligibility will receive chemotherapy and T cells in the outpatient setting at the Seattle Cancer Care Alliance under the care of the Immunotherapy service. Patients admitted at the University of Washington Medical Center or Seattle Children's Hospital at the time of planned infusion may receive the T cells as an inpatient. The infusion will consist of $CD8^+$ WT1-specific transduced T cells at a planned cell dose of **1.0×10^{10} cells/m^2** administered by intravenous (IV) infusion over approximately 45 minutes (+/- 15minutes) or longer for patients who are 15-30kg. Infusion time may be longer if required based on CPF endotoxin results or patient reaction to infusion. Vital signs and O_2 saturation should be monitored and recorded at around the following time points time 0, every 15 minutes during T cell infusion, and then hourly for 2 hours following the T cell infusion (see **Section 15.E**). Infusion-related toxicities, if observed, will be treated as outlined in **Section 18**.

2. <u>Second infusion of $CD8^+$ WT1-specific T cells; Day ≥21</u>

Patients may receive a second infusion of CD8+ WT1-specific transduced T cells at a dose of **1 x 10^{10} cell/m^2** at least 21 days after the first infusion if the patient meets persistence criteria and did not experience a toxicity requiring delaying/discontinuation of study treatment (see **Section 19**). Administration of lymphodepleting chemotherapy, provision of subsequent infusion of T cells, and monitoring will be performed as described above for infusion #1.

3. Criteria for delaying subsequent T cell infusion

Subsequent T cell infusion will be delayed if clinically indicated, or if WT1-specific T cell frequency in the peripheral blood is >3% of total CD8+ T cells. Up to 60mL (45mL for patients who are 30-50kg, 1ml/kg for patients who are 15-30kg) of blood should be drawn within 21 days prior to the scheduled date for the subsequent T cell infusion for evaluation of persistence of adoptively transferred T cells. If the WT1-specific T cell frequency is >3%, the following T cell infusion will be held. Repeat evaluation of persistence of WT1-specific T cells should be performed up to twice weekly for one month and then at the PI discretion until the WT1-specific cell frequency is ≤3%. Subsequent T cell infusion may then be administered as soon as T cell product and an infusion appointment are available.

4. IL-2 administration

If the second T cell infusion is tolerated without serious toxicity as detailed in **Section 18**, patients should then receive twice daily SC injections of recombinant IL-2 at a dose of 2.5 x 10^5 U/m^2 approximately every 12 hours for 14 days, starting between 2-4 hours after completing the T cell infusion. Patients or caregivers will be trained on IL-2 administration. IL-2 therapy may be omitted in any patient with a contraindication to receiving the drug (e.g. graft versus host disease), as determined by the PI and/or treating physician. IL-2 therapy may be discontinued in any patient developing grade 3 or greater treatment-related toxicities while receiving daily SC IL-2 injections.

14.C. Other Study Agents

14.C.1. Interleukin-2

IL-2 will be initiated within 6 hours of the second T cell infusion (second T cell infusion at a dose of 1.0 x 1010 cells/m2), at a dose of 250,000 U/m^2 s.c. twice daily x 14 days. The patient or the caregiver will be instructed on s.c. self-administration.

14.C.2. Cyclophosphamide

CY 300 mg/m^2 will be administered intravenously daily for 3 total doses (on days -4 to -2) prior to administration of T cells. Standard practice policy guidelines will be followed, and its administration will be completed at least 24 hours prior to the T cell infusion. Dose reductions will be allowed as per the patient's clinical situation and discretion of the PI or treating attending physician.

14.C.3. Fludarabine

Fludarabine at 30 mg/m^2 will also be administered daily for 3 total doses (on days -4 to -2) prior to administration of T cells. Standard practice policy guidelines will be followed, and its administration will be completed at least 24 hours prior to the T cell infusion. Dose reductions will be allowed as per the patient's clinical situation and discretion of the PI or treating attending physician.

15. EVALUATION

<u>To note</u>: The time points for evaluation, indicated in this section, may be varied due to clinical or logistic factors.

15.A. Patient and Donor Screening Prior to Enrollment

See Standard Practice Guidelines for standard Evaluation. In addition, the following samples will be obtained:

1. 5 mL of blood in EDTA (purple top) tube may be collected from the donor, if needed, for high resolution HLA typing.
2. 10 mL of donor's blood may be sent for EBV serology.
3. 2 mL of bone marrow should be sent in EDTA (purple top tubes) to the FHCRC Research Cell Bank (E1-305) for screening for WT1 expression, and for potential genetic markers (e.g. translocations, deletions, mutations) associated with the disease. If a patient does not have a sample containing leukemia cells at the FHCRC, we may request samples containing leukemia cells if they exist at outside facilities to assess for expression of WT1.
4. 5mL of blood in EDTA (purple top) tube may be sent on the patient, if needed, for high resolution HLA typing.
5. 10mL of blood in EDTA (purple top) tube may be sent on patient with circulating blasts for screening of WT1 expression and for potential genetic markers associated with disease.
6. If leukemic cells are detected by flow cytometry in blood or bone marrow during the clinical work up, the hematopathology lab may be asked to sort the leukemic cells for expression analysis of WT1 and other potential genetic markers.
7. Karnofsky performance status score (age ≥16 years) or Lansky play score (age <16 years) ≤40% for patient with no evidence of leukemia or MDS at time of screening (ARM1)
8. Patients with Karnofsky performance status score (age ≥16 years) or Lansky play score (age <16 years) ≤30% for patient with evidence of leukemia or MDS at time of screening (ARM2)

15.B. Patient Evaluation after Transplant and Prior to Planned Initiation of CTL Infusions

See Standard Practice Guidelines and transplant protocol guidelines for evaluation after transplant. When bone marrow is sampled for clinical evaluation (routinely at ~day +28 and +80-100 for myeloablative transplant and ~day+28, +56 and +80-100 for non-myeloablative transplant, as well as when clinically indicated), the following samples may also be requested:

1. 10 ml blood (3ml for patients who are 15-50kg) to be sent in EDTA tubes to the FHCRC Research Cell Bank (E1-305) for screening for WT1 expression, and for potential genetic markers (e.g. translocations, deletions, mutations) associated with the disease.
2. 2 mL of bone marrow aspirate to be sent in EDTA tubes to the FHCRC Research Cell Bank (E1-305) for screening for WT1 expression, and for potential genetic markers (e.g. translocations, deletions, mutations) associated with the disease.
3. 5 ml of bone marrow aspirate to be sent in ACD tube to the FHCRC Research Cell Bank (E1-305).

15.C. Patient Evaluation at Time of Planned Initiation of T Cell Infusions (within 30 days of T cell infusion, unless otherwise indicated)

A. History: An updated history.

B. Physical exam with attention for signs of pre-existing GVHD or toxicity to WT1-expressing tissues [e.g. pleuritis or pericarditis, pneumonitis, abdominal pain (particularly in region of splenic capsule or ovaries), and testicular pain].

C. Assessment of Karnofsky or Lansky performance status.

D. Laboratory studies including CBC, chemistry battery, and urinalysis, with special attention for evidence of pre-existing toxicity to WT1-expressing tissues as manifested by graft failure, renal dysfunction, or hematopoietic suppression.

1. Chest X-ray (CXR) and pulmonary function tests, if clinically indicated.

2. Assessment of chimerism by FISH or Variable Number Tandem Repeat (VNTR) analysis if clinically indicated.

3. Bone marrow aspirate and if clinically indicated, a biopsy should be performed within three weeks prior to initiation of T cell therapy. Bone marrow evaluation should include morphology, cytogenetics, flow cytometry, FISH, and PCR studies for MRD (if applicable). Research marrow aspirate samples also should be sent (5 ml in ACD) (2 ml in EDTA) to FHCRC Research Cell Bank (E1-305).

15.D. Evaluation Immediately Prior to each T Cell Infusion (Day 0)

1) Interval history and review of systems.

2) Physical exam, vital signs.

3) CBC, chemistry battery, urinalysis.

4) Serum or urine β-hCG pregnancy test within 14 days of planned T cells infusion.

5) 10 mL of blood in EDTA tubes should be sent to FHCRC Research Cell Bank (E1-305) (3mL for patients who are 30-50kg, not required for patients who are 15-30kg).

6) 60 mL of blood in ACD tubes should be sent to FHCRC Research Cell Bank (E1-305) (40 mL for patients who are 30-50kg, 1mL/kg for patients who are 15-30kg).

15.E. Evaluation During Each T Cell Infusion (Day 0)

1. Blood pressure, heart rate, temperature, respiratory rate and O_2 saturation (by pulse oximetry) will be recorded at around the following time points: time 0, every 15 minutes during the T cell infusion, then hourly for 2 hours following the T cell infusion.

2. Events will be managed by standard medical practice (**see also Section 18 for management of toxicities**).

15.F. Evaluation After Each T Cell Infusion

All post-treatment monitoring shall start from the T cell infusion for both first and second infusion (to avoid any confusion that such monitoring should begin after 2nd T cell infusion or completion of IL-2).

1. **For patients > 50kg**
   a. Interval history and review of systems on day +1, and weekly (starting on day +7) for four weeks after infusion #1; and day +1, and weekly for six weeks after infusion #2 (to correspond with four weeks of monitoring post-SC IL-2).

   b. Physical exam, vital signs on day +1, and weekly (starting on day +7) for four weeks after infusion #1; and day +1, and weekly for six weeks after infusion #2 (to correspond with four weeks of monitoring post-SC IL-2).

   c. CBC with differential and platelet count, serum electrolytes, BUN, creatinine, glucose, and urinalysis on day +1, +4, and weekly (starting on day +7) for four weeks after infusion #1; and day +1, +4, and weekly for six weeks after infusion #2 (to correspond with four weeks of monitoring post-SC IL-2).

   d. 10 mL in EDTA purple top tubes should be sent to FHCRC Research Cell Bank (E1-305) on day +1, +4, +7, +14, +21 and +28 following each infusion.

   e. 60mL of blood in ACD yellow top tubes should be sent to FHCRC Research Cell Bank (E1-305) on day +1, +4, +7, +14, +21 and +28 following each infusion.

2. **For patients 30-50kg**
   a. Interval history and review of systems on day +1, and weekly (starting on day +7) for four weeks after infusion #1; and day +1, and weekly for six weeks after infusion #2 (to correspond with four weeks of monitoring post-SC IL-2).

   b. Physical exam, vital signs on day +1, and weekly (starting on day +7) for four weeks after infusion #1; and day +1, and weekly for six weeks after infusion #2 (to correspond with four weeks of monitoring post-SC IL-2).

   c. CBC with differential and platelet count, serum electrolytes, BUN, creatinine, glucose, and urinalysis on day +1, +4, and weekly (starting on day +7) for four weeks after infusion #1; and day +1, +4, and weekly for six weeks after infusion #2 (to correspond with four weeks of monitoring post-SC IL-2).

   d. 3 mL in EDTA purple top tubes should be sent to FHCRC Research Cell Bank (E1-305) on day +1, +4, +7, +14, +21 and +28 following each infusion.

e. 40mL of blood in ACD yellow top tubes should be sent to FHCRC Research Cell Bank (E1-305) on day +1, +4, +7, +14, +21 and +28 following each infusion.

3. **For patients 15-30kg**
   a. Interval history and review of systems on day +1,+4, and weekly for four weeks after infusion #1; and on day +1, +4, and weekly for six weeks after infusion #2 (to correspond with four weeks of monitoring post-SC IL-2).

   b. Physical exam, vital signs on day +1, +4, and weekly for four weeks after infusion #1; and on day +1, +4, and weekly for six weeks after infusion #2 (to correspond with four weeks of monitoring post-SC IL-2).

   c. CBC with differential and platelet count, serum electrolytes, BUN, creatinine, glucose, and urinalysis on days +4, +14, +28 following infusion #1, and on days +4, +14, +28 and +42 following infusion #2. Note that additional laboratory monitoring may be performed more frequently outside of these specific dates, as per the primary treating pediatric team.

   d. 1mL/kg of blood in ACD yellow top tubes should be sent to FHCRC Research Cell Bank (E1-305) on day +4, +14 and +28 following infusion #1, and on days +4, +14, +28 and +42 following infusion #2. Note that additional laboratory monitoring may be performed more frequently outside of these specific dates, as per the primary treating pediatric team.

Additional evaluation and laboratory as indicated in section 15.G.1.b

15.G. Clinical and Laboratory Evaluation for Toxicity Following T Cell Infusions

The period of monitoring for treatment-related toxicity will start with the first T cell infusion. Patients will be followed during and after therapy as described below. To note: the evaluation days listed below are approximate, as many of the patients treated in our center reside out of the area, and it is anticipated that at four weeks after completing T cell infusions many patients will have returned home to the care of their primary physician, and not always be able to follow the exact time points as dictated by the protocol. In addition, frequency of monitoring may potentially be increased if indicated based on the patient's clinical condition.

1. Time points of evaluation for toxicities:
   a. During therapy:
   i. Interval history and physical exam: Day 0 prior to infusion, day +1 after each infusion and then weekly for 4 weeks following any T cell infusion, or for 6 weeks if IL-2 is administered. If the second infusion is delayed beyond 8 weeks, efforts will be made to obtain an interim H and P at 8 weeks, 3 months, and then every 3 months until the second infusion is administered or patient is taken off study.
   ii. Laboratory evaluation (as described in **Section 15.G.2.b**): Day 0 prior to each T cell infusion, days +1, +4, and then weekly after each infusion. For patients 15-30kg: laboratory evaluation on Day 0 prior to each T cell infusion, days +4, +14 and +28. If the second infusion is delayed beyond 8 weeks, clinical and research samples will be requested at 8 weeks, 3 months, and then every 3 months until the second infusion is administered, or more frequently as the P.I. or treating

physician recommends, depending on clinical factors and T cell persistence level.

iii. Bone marrow: Before any T cell infusion (if marrow sampling has not been performed within the prior three weeks), 3-5 days after IL-2 has been completed and then as clinically indicated (see also **Sections 15.H**). An additional "interim" restaging bone marrow sample may be requested following treatment with the first T cell infusion if the second T cell infusion is delayed by > 4 weeks.

b. After study therapy has been completed:

i. In the first 6 weeks after completion of the second T cell infusion if IL-2 is administered (or in the first 4 weeks after completion of the second T cell infusion if there is no inclusion of IL-2): weekly interval history and physical exam, and laboratory evaluation. For patients 15-30kg, interval history and physical exam, and laboratory evaluation as per 15.G.1.a.i and ii.

ii. ≥6 weeks after infusion of T cells, efforts will be made to obtain: Interval history and physical and laboratory evaluation at 6 weeks and 8 weeks; 3, 6, and 12 months for the first year and then annually for a total of 15 years depending on persistence of transferred cells and in compliance with FDA guidelines for patients receiving lentivirally-transduced cells (see also **Section 15.I**).

2. General Toxicity Assessment

a. Interval history and physical exam with vital signs, with particular attention for signs of GVHD or toxicity to WT1-expressing tissues [e.g. pleuritis or pericarditis, pneumonitis, abdominal pain (from splenic capsule or ovaries), and testicular pain].

b. Laboratory evaluation: CBC with differential and platelet count (with attention for signs of graft failure or hematopoietic toxicity), serum electrolytes, BUN, creatinine, glucose, and urinalysis (for evidence of renal toxicity).

c. Bone marrow: Bone marrow aspirate and biopsy, if needed, to evaluate for graft failure or hematopoietic toxicity (aplasia).

d. Weekly exam during SC IL-2 administration.

e. Daily record of temperature and symptoms by the patient or medical staff during SC IL-2 administration.

f. CXR day +1 after each T cell infusion, if clinically indicated.

3. GVHD Assessment

a. Skin involvement will be assessed by clinical exam, and, if abnormalities suggestive of GVHD are detected, the diagnosis should be established by skin biopsy. The percentage of body surface area involved will be recorded.

b. Gastrointestinal symptoms suspicious for GHVD may be evaluated by endoscopy and biopsy if indicated.

c. Liver function abnormalities should be evaluated in the context of the clinical status and a liver biopsy may be performed if indicated to establish a diagnosis.

d. GVHD assessment should be performed, if the patient is in Seattle, by a trained member of the medical staff, and staged and graded according to established criteria (**Protocol Appendices A and B**).

4. Toxicity

The occurrence of infusion related toxicity requiring treatment discontinuation will result in no further infusions being given and also in the administration of glucocorticoids to ablate the transferred T cells (see **Section 19** for details).

15.H. Evaluation of Persistence and Function of Adoptively Transferred T Cells and for Evidence of Leukemia/MDS

1. Blood samples:
   a. To evaluate persistence and function of adoptively transferred T cells: up to 60mL for patients > 50kg (40mL for patients who are 30-50kg, 1mL/kg for patients who are 15-30kg) of blood in ACD yellow top tubes to FHCRC Research Cell Bank (E1-305) should be obtained immediately prior to each infusion and as close as possible to the time points: days +1, +4, +7 and +14 after each infusion of T cells (days +4, +14, +28 for patients who are 15-30kg).

   b. If second T cell infusion is delayed, up to 60 mL (40mL for patients who are 30-50kg, 1mL/kg for patients who are 15-30kg) of blood in yellow top tubes may be obtained up to twice weekly for a month and then at the PI's discretion and sent to the FHCRC Research Cell Bank (E1-305) to evaluate for WT1 specific T cell persistence. All other research samples may be discontinued until T cell therapy resumes.

   c. 60 mL (40mL for patients who are 30-50kg) blood samples in ACD yellow top tubes to FHCRC Research Cell Bank (E1-305) will be obtained at days +1, +4, +7 and +14 after each infusion of T cells and approximately 3-5 days after the last T cell infusion and completion of IL-2 injections, weekly in the first four weeks, week 6, week 8, three months, six months, and 12 months.

      For patients who are 15-30kg, 1mL/kg of peripheral blood in ACD yellow top tubes will be obtained at days +4, +14, +28 after each T cells and approximately 3-5 days after the last T cell infusion and completion of IL-2 injections. At week 8, three months, six months, and 12 months up to 2mL/kg of blood samples will be obtained.

      If adoptively transferred T cells are <0.1% of $CD8^+$ T cells, persistence evaluation may be discontinued or evaluation frequency decreased, per the PI's discretion. If persistent adoptively transferred T cells are detected additional samples may be requested.

   d. 10 mL (3mL for patients who are 30-50kg) blood sample in EDTA purple top tubes should be sent to FHCRC Research Cell Bank (E1-305) to be evaluated for expression of WT1 at days +1, +4, +7 and +14 after each infusion of T cells and approximately 3-5 days after the last T cell infusion and completion of IL-2 injections, weekly in the first four weeks, week 6, week 8, three months, six months, and 12 months.

      For patients who are 15-30kg, up to 3mL blood sample in EDTA purple top tubes should be obtained and sent to FHCRC Research Cell Bank (E1-305) for evaluation of WT1 expression at week 8, three months, 6 months and 12 months.

2. Bone marrow samples:
   a. Patients will be assessed for evidence of disease by morphology, flow cytometry, PCR or cytogenetic analysis of bone marrow samples obtained as close as possible to days +0 (if marrow sampling has not been performed within the prior three weeks), 3-5 days after IL2 has been completed, and then as clinically indicated.
   b. In addition to the samples sent for clinical purposes:

i.5 ml of bone marrow (3 ml for patients 15-30 kg) in ACD yellow top tubes to FHCRC Research Cell Bank (E1-305) should be sent of any bone marrow samples obtained during this study to be evaluated for the presence of adoptively transferred T cells by WT1 and EBV (or CMV) HLA-A*0201 double peptide/multimer analysis and for other immunological parameters.

ii.2 ml of bone marrow in EDTA purple top tubes should be sent to FHCRC Research Cell Bank (E1-305) of any bone marrow samples obtained during this study to be evaluated for expression of WT1.

3. Other samples: If a biopsy of an organ is performed for clinical reasons, an additional sample may be obtained for research tests.

15. I. <u>Evaluation for long term effects of using lentivirally transduced T cells</u>

1. <u>Testing for Replication Competent Lentivirus (RCL)</u>

In compliance with FDA Guidance, "Supplemental Guidance on Testing for Replication Competent Retrovirus in Retroviral Vector Based Gene Therapy Products and During Follow-up of Patients in Clinical Trials Using Retroviral Vectors" (November 28, 2006, the same rules apply to lentiviruses), every effort will be made to obtain blood samples for testing for RCL at the following time points: Pretreatment, at 3, 6, and 12 months for the first year, and annually thereafter. If all post-treatment assays are negative during the first year, subsequent yearly samples will be archived for future analysis, if such analysis becomes clinically or scientifically indicated. Samples will be archived with appropriate safeguards to ensure long-term storage and an efficient system for the prompt linkage and retrieval of the stored samples with the medical records of the patient and the production lot records. If any post-treatment samples are positive, further analysis of the RCL and more extensive patient follow-up will be undertaken, in consultation with Center for Biologics Evaluation & Research (CBER).

2. <u>Long-Term Follow-Up</u>

Every effort will be made to follow patients for 15 years, in compliance with the FDA Guidance, "Gene Therapy Clinical Trials - Observing Subjects for Delayed Adverse Events" (November 28, 2006). As per this guidance, viruses that have a potential to integrate, including lentiviruses, "present sufficient risk that long-term follow-up (LTFU) observations are necessary to mitigate long-term risks to subjects receiving these vectors." The patients on this study will have follow-up clinical visits on the same time points as testing for RCL occurs (pretreatment, at 3, 6, and 12 months for the first year, and annually thereafter if possible). At clinical follow-up visits, patients will be examined for clinical evidence suggestive of lentiviral disease, such as cancer, neurologic disorders, or other hematologic disorders.

Additionally, efforts will be made to collect samples to determine levels of gene-modified cells in peripheral cells. Suspect clinical symptoms or findings will trigger RCL analysis of archived samples and/or attempting to obtain additional samples, in consultation with CBER. At clinic visits, patients will undergo physical examination and laboratory testing including CBC with differential, comprehensive metabolic panel, and levels of gene-modified cells. In case histories, physicians will be asked to record details of all exposures to mutagenic agents and other medications. Physicians will also be asked to record the emergence of new clinical conditions, including new malignancies, new incidence or exacerbation of a pre-existing neurologic disorder, new incidence or exacerbation of a prior rheumatologic or other autoimmune disorder, and new incidence of a hematologic disorder. Study subjects and health providers will be asked to cooperate in reporting delayed adverse events, including unexpected illness and

hospitalization. Patients will generally be seen by their doctors in their local area. However, the FHCRC in collaboration with Seattle Cancer Care Alliance (SCCA) will be available to assist in the LTFU of participants in this clinical trial. If patients die or develop neoplasms, recommendation will be made to assay for RCL in a biopsy sample of the neoplastic tissue or the pertinent autopsy tissue.

16. MANAGEMENT OF T CELL INFUSION IN PATIENTS WHO RELAPSE OR PROGRESS DURING T CELL THERAPY

Patients who started treatment with no evidence of disease (Arm 1) and relapsed during T cell therapy, or patients who started treatment with evidence of disease (Arm 2) and experience progression of their disease during T cell therapy will be evaluated for the characteristics of the leukemic cells and advised about treatment options by their treating physician.

T cell therapy may be continued if relapse or disease progression occurs prior to the last dose of T cells infusion, at the discretion of the PI. The T cell infusions may be interrupted to allow administration of cytoreductive therapy. Patients who receive cytoreductive chemotherapy may resume treatment with WT1-specific T cells after evidence of recovery of hematopoiesis (ANC >500/mm^3 and plts > 30K).

This decision is based on the extremely poor prognosis for these patients, the fact that they might benefit from infusion of available WT1-specific T cells, and the possibility of acquiring insights into safety and potential anti-leukemic effects of WT1-specific T cells.

Patients whose T cell therapy is interrupted for cytoreductive therapy will not undergo protocol specific evaluations, AE assessments or sample collections until T cell therapy resumes.

17. OPTIONS FOR FURTHER TREATMENT AFTER COMPLETION OF STUDY THERAPY

If adoptively transferred WT1-specific T cell infusion demonstrated anti-leukemic activity, the option of additional T cell infusions may be discussed with the patient and treating physician, at the discretion of the PI.

18. TOXICITIES

18.A. Toxicity Grading

Toxicities will be graded according to the NCI Common Terminology Criteria for Adverse Events (CTCAE) Version 4. The full text of the NCI CTCAE is available online at: http://evs.nci.nih.gov/ftp1/CTCAE/About.html.

18.B. Immediate Toxicities:

Immediate reaction to infusion (i.e. defined as those occurring during the first 24 hours following T cell infusion) might occur due to release of cytokines from T cells stimulated by the recognition of targets, due to direct cytotoxic effects of CTL on target tissues expressing WT1, from normal tissues expressing antigens recognized by mispaired TCR, or from allogeneic T cells not specific for EBV that were not completely excluded during the generation of the WT1-specific T cells.

a) **Milder reactions** (i.e. < grade 3 CTCAE v.4 or less severe than specified below) would include symptoms such as:

- Fever, chills, fatigue.
- Dyspnea, chest tightness, myalgia, or pain (chest, pleuritic, abdominal, back, testicular).
- Alteration in vital signs such as:
- Lowering of blood pressure, but with systolic BP ≥90 mm Hg, or ≤20 mm Hg below baseline.
- Tachycardia, but with HR ≤130 or ≤30 above baseline.
- Tachypnea, but with RR ≤32/min or ≤10 above baseline.
- Hypoxemia, but O_2 saturation ≥88% on room air, or ≤5% fall from baseline.
- Skin changes such as erythema, urticaria, or other rash.

Management will be **at the discretion of the PI or designee, and may include appropriate supportive care** such as:

- Acetaminophen or Demerol for fever and chills (All subjects who develop fever or chills should have a blood culture drawn).
- Acetaminophen for headache.
- Diphenhydramine for nausea and vomiting.
- Fluid administration for hypotension.
- Supplemental oxygen for hypoxemia.
- Decreasing the rate of the infusion

b) **More severe reactions** would include symptoms such as,

- Hypotension with systolic BP <90 mm Hg and >20 mm Hg below baseline.
- Tachycardia with HR >130 and >30 above baseline.
- Tachypnea with RR >32 and >10 above baseline.
- Hypoxemia with O_2 saturation of <88% and >5% fall from baseline.

Management will be by **modifying the infusion rate or terminating the infusion at the discretion of the PI or designee, and administering supportive medical care.**

- If patient responds to supportive care by normalization of vital signs or resolution of hypoxemia and PI deems it safe to continue, the infusion may be resumed/infusion rate increased.

- If the patient does not respond by normalization of vital signs or hypoxemia after supportive care alone, **methylprednisolone (MP) to ablate the infused T cells should be administered as per Section 18.D.**

c) **Any unexpected severe toxicity (see Section 18.E)** occurring in the first 24 hours (due to the T cell infusion and not attributable to a non-infusion related cause).

Management will be by **supportive medical care and MP will be administered, per the discretion of the PI or designee, as per Section 18.D.**

18.C. Delayed Toxicities

It is possible that the infusion of $CD8^+$ WT1-specific transduced T cells will result in delayed toxicities related to the recognition of tissues expressing WT1. It is anticipated that the symptoms and signs will occur within hours to four weeks after completion of either the T cell infusion or IL-2 administration. **Any toxicities requiring treatment discontinuation (as described in Section 18.E) occurring within four weeks of study treatment will be managed by supportive care and MP will be administered, per the discretion of the PI or designee, as per Section 18.D.** Evaluation and management of hematologic toxicities are described in **Section 18.E.2.** Acute GVHD will be managed as described in **Section 18.E.3**.

Any patient who develops clinical evidence of a **cytokine storm** will have a workup to exclude infection or other causes. Initial treatment will consist of supportive measures as dictated by the clinical and laboratory findings, and may include fluid replacement, antipyretics, oxygen supplementation, and broad-spectrum antibiotics if infection cannot be excluded as a potential etiology for the signs and symptoms. Serious and/or progressive symptoms and signs may result in the administration of corticosteroids as described in **Section 18.D** addressing management of serious adverse events.

Blood samples for research tests are collected in all patients prior to the T cell infusion and on days +1, +4, +7, and +14 (days +4, +14 and +28 for patients weighing 15-30kg) after the T cell infusion, as indicated in **Section 15.G**. Plasma will be isolated from each blood sample and stored for cytokine analysis. If patients have clinical evidence of a cytokine release storm, the samples will be analyzed for cytokine analysis (including IFN-γ, TNF-α, IL-2 concentrations).

18.D. Management of Severe Treatment-related Toxicities with Methylprednisolone

Patients who require glucocorticoids for treatment of severe toxicities will receive **Methylprednisolone (MP) at 2 mg/kg/day IV** if WT1-specific T cells are ≥0.05% of $CD8^+$ cells in peripheral blood, and the patient will be taken off study. MP (or an equivalent dose of an alternative glucocorticoid) can be tapered when symptoms are significantly improving or WT1-specific T cells are <0.05% of $CD8^+$ cells in peripheral blood. *These are general guidelines for the glucocorticoid taper and may have to be adjusted based on each patient's clinical situation.*

Patients receiving glucocorticoids should have **assessments of peripheral blood for the presence of WT1-specific CTL cells (vβ17$^+$ and dual-multimer$^+$)** will be performed according to the PI or designee discretion. Twenty ml of blood will be sent in ACD yellow top tube to the FHCRC Research Cell Bank (E1-305) for the evaluation of the presence of the infused T cells. Modification of WT1-specific CTL persistence assessment may be made per the PI or designee discretion.

18.E. Definition and Management/Evaluation of Non-hematologic and Hematologic Toxicities Requiring Treatment Discontinuation

- **Non-hematologic toxicity** requiring treatment discontinuation is defined as any grade 3 or 4 non-hematologic toxicity (CTCAE 4) that is deemed to be caused by infusion of the study treatment (i.e. not attributable to infection, recurrent/progressive leukemia, toxicity from other therapies, or any identifiable cause other than T cell infusion or IL-2 administration; and that is not an expected cytokine-mediated symptom as further described below) that occurs at any time point after the first T

cell infusion. It is expected that the onset of non-hematologic toxicities will be within four weeks after completion of study therapy although patients will be followed beyond four weeks for potential toxicities, as described in **Section 15.G.**

Transient high fever may occur due to release of cytokines from T cells stimulated by the recognition of targets, and therefore grade 4 toxicity from fever (>40 °C) lasting ≤36 hours will not require treatment discontinuation. Symptoms of cytokine release that are grade 3 or less (cytokine release syndrome, tachycardia, chills, rigors, fatigue, myalgias, arthralgias, dizziness, rash, urticaria, hypotension not requiring pressor support or > 3L fluid boluses in 24 hours, dyspnea or hypoxemia not requiring > 2L/min supplemental oxygen for longer than 48 hours) are similarly expected to possibly occur due to release of cytokines from T cells stimulated by the recognition of targets, and therefore will not require treatment discontinuation.

If possible, an attempt will be made to obtain biopsies of affected tissues to determine if transduced T cells are accumulating at the site.

Toxicities will be managed as described in **Section 18.C**.

2. Hematologic toxicity requiring treatment discontinuation (blood/bone marrow CTCAE 4) is defined as any new or recurrent onset of grade 4 hematologic toxicity that occurs at any time point after the first T cell infusion, and last for two consecutive days, such as neutropenia (ANC <500/mm^3) in the context of new bone marrow aplasia (<5% cellularity), which is <u>attributed</u> to the study treatment (i.e. not associated with prior chemotherapy, leukemic progression in the bone marrow, transplantation, infection, toxicity from other therapies, or any identifiable cause other than T cell infusion or IL-2 administration). Transient drop in lymphocyte count is expected after T cell infusion. Therefore, grade 4 lymphopenia, if returns to pre-infusion level within 14 days, will not require treatment discontinuation. Thrombocytopenia threshold for treatment discontinuation is platelets <20,000/mm^3. This differs from CTCAE 4 for guidelines for Grade 4 hematologic toxicity for thrombocytopenia, but will be used in this trial, as thrombocytopenia is a common problem in this patient population

Management: If a patient develops new onset hematologic toxicity such as ANC <500/mm^3 or platelets <20,000/mm^3 for two consecutive days, which cannot be explained by alternative cause, a bone marrow sample should be obtained.

- If the bone marrow shows evidence of aplasia, MP will be started as described in **Section 18.D** and study treatment discontinued. G-CSF may be administered at the discretion of the medical team.
- If the bone marrow shows no evidence of aplasia and no disease progression or other clear etiology for marrow suppression is found, patients will be observed for three more days. If by that time there is no improvement of bone marrow function and no clear etiology for marrow suppression (e.g. infection) is detected, MP will be started as described in **Section 18.D** and study treatment discontinued. G-CSF may be administered at the discretion of the medical team.
- If bone marrow cannot be obtained and no alternative cause of hematologic toxicity is identified by the third consecutive day of neutropenia or thrombocytopenia, MP will be started as described in **Section 18.D** and study treatment discontinued. G-CSF may be administered at the discretion of the medical team.

It is expected that onset of hematologic toxicities will be within four weeks after completion of study therapy, although patients will be followed beyond four weeks for potential toxicities as described in **Section 15.G**.

3. Acute GVHD: Unlike the unselected polyclonal population of T cells administered as part of DLI, this protocol utilizes selected populations of virus-specific $CD8^+$ T cells that have been transduced to recognize WT1. Therefore the incidence of classical GVHD due to CTL infusions is expected to be very low if at all detectable. The risk should be even further reduced compared to DLI since no naïve T cells will be included in the infusion.

Patients will be assessed for status of acute and/or chronic GVHD within 3 weeks prior to receiving the study treatment, and again at 3 months, 6 months and 12 months following any T cell infusion, and then annually, as clinically indicated.

Management: Patients developing grade III-IV acute GVHD will be treated for acute GVHD with glucocorticoids according to Standard Practice guidelines. If possible, biopsies of affected tissues will be obtained prior to glucocorticoids treatment to determine if transduced T cells are accumulating at the site. Patients receiving glucocorticoids will have assessments of peripheral blood for the presence of WT1-specific T cells. If GVHD is attributed to the study treatment, treatment will be discontinued.

- A patient will no longer be eligible to receive additional T cell infusions if the PI or designee determines that additional T cell infusions are not in the best interest of the patient.

18.F. Chronic GVHD

Patients found to have chronic GVHD will be treated as directed by their primary team and/or per recommendations of the GVHD or LTFU attending physicians. If possible, biopsies of affected tissues will be obtained prior to treatment to determine if transduced T cells are accumulating at the site.

18.G. Detection of Replication Competent Lentivirus (RCL)

Finding evidence of RCL or evidence suggestive of lentiviral disease (see **Section 15.I**) will be considered a treatment-related toxicity.

19. OFF STUDY CRITERIA

A patient's participation on the protocol will be terminated for any of the reasons listed below:

1. The participant withdraws consent.
2. Inability to generate transduced t cells that meet release criteria for t cell therapy.
3. Patient death.

If a patient progresses on T cell therapy, pursues alternative systemic therapy, cannot receive further T cell therapy due to toxicity or clinical status, or if the treating physician or the protocol PI deems that it is not in a patient's best interest to continue in this study, that patient will be designated as "off treatment". To comply with FDA requirements for long-term monitoring for potential toxicities related to use of lentivirally-transduced T cells, such patients who do not otherwise withdraw consent will nevertheless continue to be followed by the study team for survival outcomes and for late toxicities, as per **Section 15.I**.

20. PROTOCOL REGISTRATION AND SPECIAL CONSIDERATIONS

HLA-A*0201 is expressed in all ethnic groups, but is most common in Caucasians, which represents the most common ethnic group treated at our center, and therefore it is expected that the majority of patients on this trial will be Caucasian. However, patients from all ethnic groups will be enrolled if they meet all eligibility criteria. If these studies demonstrate safety and potential benefit, it is anticipated that TCR genes specific for WT1 in the context of other HLA alleles, more predominant in other ethnic groups, will be isolated and afford the option of expanding the trial to be more inclusive of all patients.

20.A. Projected Target Accrual - Arm 1

Table 5. Ethnic and gender distribution chart – Arm 1

TARGETED / PLANNED TREATMENT: Number of Subjects			
ETHNIC CATEGORY	Sex / Gender		
	**Females**	**Males**	**Total**
Hispanic or Latino	1	1	2
Not Hispanic or Latino	17	16	33
Ethnic Category Total of All Subjects	18	17	35
RACIAL CATEGORIES	Sex / Gender		
	**Females**	**Males**	**Total**
American Indian / Alaska Native	0	0	0
Asian	1	1	2
Native Hawaiian or Other Pacific Islander	0	1	1
Black or African American	1	0	1
White	16	15	31
Racial Categories: Total of All Subjects	18	17	35

20.B. Projected Target Accrual - Arm 2

Table 6. Ethnic and gender distribution chart – Arm 2

TARGETED / PLANNED TREATMENT: Number of Subjects			
	**Sex / Gender**		
ETHNIC CATEGORY	**Females**	**Males**	**Total**
Hispanic or Latino	1	1	2
Not Hispanic or Latino	9	9	18
Ethnic Category Total of All Subjects	10	10	20
	**Sex / Gender**		
RACIAL CATEGORIES	**Females**	**Males**	**Total**
American Indian / Alaska Native	0	0	0
Asian	1	1	2
Native Hawaiian or Other Pacific Islander	0	0	0
Black or African American	0	0	0
White	9	9	18
Racial Categories: Total of All Subjects	10	10	20

Since not all patient's enrolled on the study will receive treatment the enrollment numbers may be higher than those in the tables above.

21. GUIDELINES FOR ADVERSE EVENT REPORTING

21.A. Reporting of Adverse Events (AEs)

All unexpected *and* serious adverse events which may be due to study treatment or intervention *must* be reported to the FHCRC Institutional Review Office per their current reporting requirements.

All grade ≥3 CTCAE v.4 AEs will be collected from the time of first T cell infusion through 30 days after the last study intervention (T cell infusions + IL2). For those patients with an interval of > 3 months between T cell infusions, collection of all grade ≥3 AEs will temporarily put on hold after 3 months, and then resume at the onset of any additional study treatments (e.g. lymphodepleting chemotherapy and 2nd T cell infusion.) Beginning 30 days after last study intervention, study related toxicities will be collected as discussed in **Section 15** and **Section 18**.

All serious adverse events that are unexpected and related to study treatment will be reported to the FDA within 15 calendar days. The reports will include the date and time of onset, severity and duration of the event, the relationship to study treatment, the treatment given, and the eventual outcome.

21.B. Definitions

Definitions associated with reportable events can be found on the FHCRC IRO extranet website. (Relevant FHCRC policies include, but are not limited to the following documents. Please also refer to the FHCRC IRO website.)

Table 7. FHCRC IRB policies for reportable events

IRB Policy 2.6	Adverse Events and Other Unanticipated Problems Involving Risks to Subjects or Others	http://extranet.fhcrc.org/EN/sections/iro/irb/ae.html
IRB Policy 1.9	Noncompliance with the Office of the Director's Human Research Protection Program Policy	http://extranet.fhcrc.org/EN/sections/iro/irb/ae.html
IRB Policy 1.1	Reporting Obligations for Principal Investigators	http://extranet.fhcrc.org/EN/sections/iro/irb/policy/index.html
IRB Policy 2.2	Continuing Review	http://extranet.fhcrc.org/EN/sections/iro/irb/policy/index.html
IRB Policy 1.13	Investigational New Drugs (IND), Biologics and Investigational Device Exemptions (IDE)	http://extranet.fhcrc.org/EN/sections/iro/irb/policy/index.html

Toxicity grading

Toxicities will be graded using the NCI Common Terminology Criteria for Adverse Events (CTCAE) Version 4. Information on this scale can be found at http://evs.nci.nih.gov/ftp1/CTCAE/About.html.

22. DATA AND SAFETY MONITORING PLAN

22.A. Primary Monitoring

The PI of the study will have primary responsibility for ensuring that the protocol is conducted as approved by the Scientific Review Committee and Institutional Review Board. The PI will ensure that the monitoring plan is followed, that all data required for oversight of monitoring are accurately reported to the Protocol Office and Data Safety Monitoring Board (DSMB), that all adverse events are reported according to protocol guidelines, and that any adverse reactions reflecting patient safety concerns are appropriately reported.

22.B. Monitoring Plan

The PI, or a co-investigator on the study designated by the PI, will personally review with the Study Nurse the clinical course of all WT1-specific T cell infusions. At least twice monthly the PI or his/her designee will also review with the Study Nurse the progress of each patient undergoing therapy with WT1-specific T cells. At least monthly the PI will also meet with the Study Nurse to review the clinical course of all patients who have completed a course of T cell therapy.

22.C. Monitoring the Progress of Trials and the Safety of Participants

The FHCRC PI is responsible for monitoring this clinical trial, with oversight by a DSMB, the Protocol and Data Monitoring Committee (PDMC) at the FHCRC, and the FHCRC IRB. This is a Phase I/II study and the assessment of risk is considered above minimal.

The study will be monitored by the Immunotherapy Integrated Research Center (IIRC) DSMB. The DSMB will be responsible for safeguarding the interests of trial participants and assessing the safety and efficacy of the interventions during the trial. This responsibility will be exercised by providing recommendations about stopping or continuing the trial. To contribute to enhancing the integrity of the trial, the DSMB may also formulate recommendations relating to the selection, recruitment and retention of participants and their management; adherence to protocol-specified regimens; and the procedures for data management and quality control.

The DSMB will be advisory to the study Sponsor and the PI, who will be responsible for prompt review of the DSMB recommendations to guide decisions regarding continuation or termination of the trial and whether amendments to the protocol or changes in study conduct are required.

The DSMB is an independent, multidisciplinary group consisting of clinical experts and a statistician who collectively have experience in leukemia, lymphoma, hematology, biostatistics, and the conduct and monitoring of clinical trials. The DSMB will meet approximately every 6 months to review data. The current members are listed in the IIRC DSMB charter.

An external monitor will be retained to monitor study progress. The scope of monitoring will be based on the FHCRC/UW Data and Safety Monitoring Plan (DSMP): http://www.cancerconsortium.org/rto/prr/DSMPlan.pdf. Per the DSMP, subjects will be randomly selected for verification. An initial monitoring visit is expected within six months of enrollment of the first subject and preferred prior to enrollment of > 4 subjects as 100% verification is expected during an initial visit.

Flow of information concerning clinical trial participants originates with the clinicians and nurses in the clinic or referring clinicians at other institutions and will be transmitted to the FHCRC Research Nurse. At the FHCRC, health care providers and rotating attending physicians assess patients and will record their observations regarding toxicity and response outcomes in the medical record. Thus, multiple health care providers will be providing independent observations and participating in monitoring this trial. The PI may be a responsible clinician for some patients entered on this trial. However, assessments will be the sum total of the multiple clinicians involved with the patient, which should avert possible conflict of interest if the PI is the attending clinician for a protocol patient. If determination of adverse events is controversial, co-investigators will convene on an ad hoc basis as necessary to review the primary data and render a decision.

23. RECORDS

The Clinical Research Division at the FHCRC maintains a patient database that allows for the storage and retrieval of specific types of patient data including demographic information, protocol registration information, and data from the transplant course. These data are collected from a wide variety of sources and conform to institutionally established guidelines for coding, collection, key entry and verification. Each patient will be assigned a unique patient number (UPN) to assure patient confidentiality. Any publication or presentation will refer to patients by this number and not by name. Information about patients enrolled on this protocol that is

forwarded to agencies such as the FHCRC IRB, NIH, and FDA will refer to the patients only by their UPN.

Original inpatient and outpatient medical records will be maintained by the medical records departments at the institutions where the patients receive their care. The majority of their care related to this protocol will be received at the SCCA, UW Medical Center and Seattle Children's. The study nurse and/or data coordinator will maintain a Case Report Form (CRF) Notebook for each patient treated on this protocol. The CRF notebooks and their contents will be identified by the patient's initials and UPN only. All supporting documents used to verify the accuracy of the data in the case report forms will be kept separately. Patient research files will be kept in a locked, controlled-access building. At least monthly, the PI or a designated co-investigator will review and cross check the data entered on the case report forms with the source documents.

24. STATISTICAL CONSIDERATIONS

The primary objectives of this study are to estimate the toxicity rate associated with infusing donor $CD8^+$ T cells that have been transduced to express a high affinity T cell receptor specific for WT1 in patients at high risk for post-transplant relapse of AML, MDS, or CML (Arm 1) and to examine the anti-leukemic activity and potential toxicities of these cells in patients with relapsed AML, MDS, or CML post transplant (Arm 2). The protocol will enroll eligible patients at or post transplant, and accrual will continue until either 35 patients have been enrolled and treated on Arm 1 and 20 patients have been enrolled and treated on Arm 2 or until five years have elapsed.

Patients will be followed for 15 years, in compliance with the FDA guidance for patients that have received lentivirally-transduced cells. As with all transplant patients, individuals on this protocol will also be seen for a detailed follow-up at one year post HCT at the LTFU Clinic at the FHCRC. This should provide the opportunity to assess potential toxicities of the study therapy as well as anti-leukemic activity as reflected by prevention of relapse and/or prolongation of disease free survival. If the safety profile is acceptable (i.e. study is not stopped due to excess toxicity as detailed in stopping rules below) and the anti-leukemic efficacy in this study appears promising (as described below) then a subsequent study will be designed to more definitively address potential efficacy in preventing and treating relapse of leukemia or MDS after HCT. Such a subsequent study would also utilize additional insights obtained from this trial regarding limitations to efficacy and could involve exploring strategies to increase the observed anti-leukemic efficacy, e.g. by transducing T cells with a higher affinity TCR, and/or by co-administering a mAb that blocks signaling to the infused T cells through the PD-1 receptor.

From 2005-2010, an average of approximately 50 patients with high-risk AML, 25 patients with high-risk MDS, and 7 patients with CML beyond chronic phase underwent allogeneic transplant each year at our center. It is expected that nearly all of these patients will have disease that expresses WT1 significantly above background levels (~90-100% of cases),[7] and at the FHCRC ~40-44% of treated patients are HLA-A*0201 positive, similar to the general US population. Given these numbers, the desired patient accrual would appear to be achievable even after accounting for patients who are likely to be excluded due to Grade III-IV acute GVHD (~15-20%), meeting other exclusion criteria, and/or refusal to consent.

The sample size of 35 patients to be enrolled on Arm 1 was not chosen based on formal power calculations, but rather was chosen after consultation with our biostatistics team to represent a number that is achievable (as detailed above) and will be sufficient to provide important

descriptive information regarding the promise (see below) of this approach and determine if it is worthy of further study and to reveal if the toxicity is acceptable. Regarding the precision of estimates for the parameters that are of interest, with 35 patients we can be 80% confident that the estimate of toxicity (or any alternative binary outcome) will be within at least 0.11 of the true value.

The sample size of 20 patients to be treated on Arm 2 of this trial was not chosen based on formal power calculations, but rather was chosen after consultation with our biostatistics team to represent a number that is achievable and will be sufficient to provide important descriptive information regarding the promise (see below) of this approach and determine if it is worthy of further study. It is expected that ~15-20% of the high risk eligible patients, who enrolled on the study at the time of transplant, will have evidence of MRD or relapse by day 60 after myeloablative HCT and ~30-35% will have evidence of disease after non-myeloablative HCT by day 90.[125,126] These relapsed patients will be eligible for treatment on Arm 2 of this study. Based on the above anticipated relapse rates, we expect 6-8 patients to be eligible for treatment on Arm 2 per year. We also expect that 2-4 HLA-A*0201+ patients/year who received an HCT for lower risk AML, MDS, or CML will relapse post-HCT, with nearly all having disease that expresses WT1 significantly above background levels (~90-100%),[5] and these patients will also be offered enrollment on Arm 2 of this study. Assuming some patients will not fulfill inclusion criteria and some will choose not to participate on the trial, with the numbers above we anticipate no difficulty enrolling/treating 20 patients on Arm 2 of the trial within five years.

Regarding the precision of estimates for the parameters that are of interest, with 20 patients we can be 80% confident that the estimate of a binary outcome (such as durable response as defined below) will be within at least 0.14 of the true value. With 20 patients, we will not likely be able to say that outcomes such as response rate are statistically significantly better than historical rates, but if our observed rates are deemed encouraging as defined below, a larger trial could be designed to more definitively assess efficacy as stated above. Additionally, if evidence of anti-leukemic activity are demonstrated in Arm 2, in concert with data from Arm 1, and demonstrate the absence of serious toxicity, that would justify extending this therapy in normal risk leukemia patients undergoing HCT and patients not undergoing an HCT.

24.A. Analysis of Toxicity (Arm 1 and Arm 2)

Toxicity analysis will be performed for both study arms; however, each arm will be analyzed independently.

The period of monitoring for treatment-related toxicity will start with the first T cell infusion. For details regarding toxicity evaluation during treatment see **Section 15** and **Protocol Appendix A and B**. During the first month after completion of T cell therapy, patients will be followed with a weekly clinical exam and laboratory evaluation, and during the second month a clinical exam and laboratory evaluation will be performed every two weeks. For the rest of the first year, patients will be seen and laboratory tests performed at 3, 6, and 12 months after completion of therapy and annually thereafter for a total of 15 years in compliance with FDA guidelines for patients receiving lentivirally-transduced cells (see **Section 15.I.** for details). To note; the evaluation time points listed above are approximate, as many of the patients treated in our center reside out of the area, and it is anticipated that at four weeks after completing therapy many patients will have returned home to the care of their primary physician, and not always be able to follow the exact time points as dictated by the protocol. In addition, frequency of monitoring may potentially be increase if indicated based on the patient's clinical condition.

a) Toxicities to organs expressing WT1
As described earlier, potential toxicities of WT1-specific T cell infusions include damage to tissues that express low levels of WT1, possibly resulting in hematopoietic suppression or graft failure, renal dysfunction, pleuritis or pericarditis, abdominal pain (from splenic capsule or ovaries), and/or testicular or ovarian pain.

Treatment on both arms will be initiated as soon as the WT1-specific T cells have been generated. This means that patients may receive therapy at a time point when they are still at risk to develop GVHD or other transplant-related toxicities. Thus, there may be situations when study treatment-related toxicity may be difficult to differentiate from transplant-related toxicities. However, due to the poor prognosis of the patients enrolled on this study, the potentially higher risk of indeterminate toxicities will be explained to the patient and has been considered in the design of stopping criteria. **The toxicity analysis will be performed for Arm 1 and Arm 2 independently.**

Arm 1:
Patients after allogeneic HCT develop major organ (e.g. lung, liver, kidney) toxicity at a rate of 15-20%.[23] Based on this expected toxicity rate, the acceptable toxicity rate determined to be related to the study treatment on Arm 1 will be 30%. If there is sufficient evidence to suggest that the true toxicity rate exceeds 30%, Arm 1 will be suspended pending review by the DSMB. Sufficient evidence will be taken to mean any observed proportion of toxicities for which the associated one-sided lower 90% confidence limit exceeds the acceptable threshold. If the true significant (requiring treatment discontinuation) toxicity rate (as defined in **Section 19**) exceeds 30% on Arm 1, infusion of T cells transduced to express a high affinity WT1-specific TCR will be considered excessively toxic for use for prophylactic therapy post-HCT. With 35 treated patients, the likelihood of having stopped the trial if the true toxicity is only 15% would be 0.08, and if the true toxicity is 40% it would be 0.91 (estimated from 5,000 simulations).

1. Operationally, any of the following observed ratios of toxicities/treated patients would yield such a confidence limit and would warrant suspension of Arm 1 and DSMB review: 2/2, 3/3-4, 4/5-6, 5/7-9, 6/10-11, 7/12-14, 8/15-16, 9/17-19, 10/20-22, 11/23-25, 12/26-27, 13/28-30, 14/31-33, 15/34-35-. If the true probability of toxicity is 15%, then the probability of reaching one of the above ratios after 20 or 30 patients is approximately 0.08 and 0.08, respectively. For a true probability of toxicity of 40%, the probabilities of one of these ratios occurring is approximately 0.83 and 0.91, respectively (estimated from 5,000 simulations).

Arm 2:

1. As for arm 1, the acceptable toxicity rate on on arm 2 is 30%. If there is sufficient evidence to suggest that the true toxicity rate on this arm exceeds 30%, Arm 2 will be suspended pending review by the DSMB. Sufficient evidence will be taken to mean any observed proportion of toxicities for which the associated one-sided lower 90% confidence limit exceeds the acceptable threshold. If the true significant (requiring treatment discontinuation) toxicity rate (as defined in **Section 19**) exceeds 30% on Arm 2, infusion of T cells transduced to express a high affinity WT1-specific TCR will be considered excessively toxic for treating relapse post HCT.

2. Operationally, any of the following observed ratios would yield such a confidence limit and would warrant suspension of Arm 2 and DSMB review: 3/3-4 (3 of the first 3 or 4 patients),

4/5-6, 5/7-9, 6/10-11, 7/12-14, 8/15-16, 9/17-19, 10/20. If the true probability of toxicity is 15%, then the probability of reaching one of the above ratios after 20 patients is approximately 0.11. For a true probability of toxicity of 50%, the probability of one of these ratios occurring is approximately 0.79 (estimated from 5,000 simulations).

Clinical and laboratory information will be gathered that may be linked to or predict toxicities, and will be used to help design future clinical trials. This may include the patient's age, comorbidities, or transplant-related toxicities prior to T cell therapy, e.g. in WT1-expressing organs (such as renal insufficiency related to use of calcineurin inhibitors), as well as the peak frequency and the persistence of infused T cells after transfer.

b) Acute GVHD
Induction of acute GVHD is another potential toxicity caused by WT1-specific T cell infusion. At the FHCRC the incidence of grade III-IV acute GVHD in the first 30 days after DLI is ≤10%. Selecting virus-specific $CD8^+$ T cells (EBV- or CMV-specific) for transduction with the WT1-specific TCR decreases the possibility of acute GVHD by excluding T_N cells and eliminating most if not all alloreactive donor T cells. GVHD may also potentially result from the creation of new TCRs on transduced T cells as a result of mispairing of the endogenous α and β chains with the introduced β or α chains. However, several strategies are being employed to reduce/eliminate this risk, including introduction of a point mutation to create a cysteine in each of the introduced TCR chains that forms an interchain bond and has been shown to nearly completely eliminate any mispairing, and the use of virus-specific T cells for transduction with the TCR chains which greatly limits the repertoire of endogenous TCR chains available to form mismatched pairs.[66] Even in the absence of using these combined strategies, no toxicity from mismatched pairing has ever been described in human trials utilizing TCR-transduced T cells. It is expected that such toxicities would present in organs usually affected in acute GVHD (e.g. skin, gut, and liver), but could result in unusual manifestations of acute GVHD due to alloreactive cells with a unique specificity. Theoretically, IL-2 may also potentially increase the risk of acute GVHD. However, in a recent study at our institute, DLI given for relapsed acute leukemia was followed by IL-2 (1-3 x 10^6 IU/m^2 /day for nine days followed by 1 x 10^6 IU/m^2 /day for five days, which is 2-6 times higher than the dose of IL-2 that will be used in this study), without evidence of higher rate of grade II-IV GVHD compared to DLI without IL-2.[34] Also, in **BB-IND 12046 - FHCRC Protocol 1655,** patients have not experienced evidence of new or flared acute GVHD after receiving the same IL-2 treatment that will be used in this protocol.

In the absence of DLI, the cumulative incidence of grade III-IV acute GVHD is ~15-20% at the FHCRC and has reached a plateau by day +60 after myeloablative transplant and day +90 after non-myeloablative transplant. At the FHCRC, ~27% of patients will reach these time points without a diagnosis of any grade of acute GVHD, and only ~5% of this 27% of patients subsequently develop grade III-IV GVHD. In patients who have less than grade III GVHD at these time points, it is estimated that ~10% of those patients will progress to develop grade III-IV GVHD. It is therefore expected that some patients may experience grade III-IV acute GVHD after these time points, even if they would never have received the study therapy.

- Patients with grade III or IV acute GVHD prior to initiation of therapy will be considered ineligible for treatment on this trial. However, study treatment may start early post-transplant, potentially during the time period when acute GVHD may still develop due to the transplant itself, and not due to the infused T cells Based on the incidence of grade III-IV acute GVHD after HCT (15-20%)[81] and after DLI (20-30%), [2,3,32-34] the acceptable rate of grade III-IV acute GVHD determined to be related to the study treatment on each arm of the study will be 30%, in the context of the poor prognosis of the patients enrolling in this study

with the potential early initiation of T cell infusion during the time period when GVHD may still develop due to the transplant itself.. If there is sufficient evidence to suggest that the true grade III-IV acute GVHD rate exceeds 30% on any arm of the study, this arm will be suspended pending review by the DSMB. Rules that would trigger such a suspension for each or the study arms are as described above.

d) Chronic GVHD
It is expected that symptoms of GVHD due to T cell infusion would be similar to what has been observed with DLI, and thus similar to classical acute GVHD. However, it is also possible that the T cell infusions could lead to the development of chronic GVHD. The probability of developing extensive chronic GVHD one year after HCT in patients currently treated on protocols at the FHCRC is ~70%, and this rate will be used as a guideline to retrospectively assess at the end of the study the risk of chronic GVHD due to T cell infusions. However, based on this high frequency and the delayed time of occurrence after transplant, no formal stopping/suspension rules will be put in place for chronic GVHD, and all chronic GVHD will be treated per standard therapy.

As described above for toxicity, we will gather information in an attempt to determine if any increased risk of acute and/or chronic GVHD in patients treated with infusion of virus-specific T cells transduced to express a WT1-specific TCR is associated with either the infused T cells or other factors such as IL-2, and such information will be used to inform the potential design of subsequent trials. This will include correlating the timing of study therapy after transplant, previous GVHD, age, donor-patient gender mismatch, conditioning therapy for HCT, and GVHD prophylactic therapy, with the frequency of acute and chronic GVHD. Also, in patients who develop GVHD, efforts will be made to obtain biopsies and assess the samples for infiltration of the transduced T cells and to determine if there is emergence of an oligoclonal population with a particular set of endogenous TCR chains that might be responsible for forming alloreactivity by mispairing with the transduced TCR. Additionally, especially in cases of chronic GVHD, the frequency and persistence of infused T cells with development of GVHD will be determined. These analyses need to be considered as hypothesis generating as the power to observe statistically significant associations is somewhat limited given the sample size.

24.B. Analysis of Efficacy in Preventing Relapse (Arm 1)

Even though examination of safety is the primary objective of Arm 1 of this protocol, we shall also look at potential efficacy as defined by prevention of relapse at one year after transplant. For the high risk population that will be eligible for this arm, we would expect a one-year relapse probability of ~40% after myeloablative HCT and ~45% after non-myeloablative HCT.[28,29] The rate of 40% will therefore be used as a guideline for assessment of potential efficacy in the current trial, but no formal rules will be in place to deem this approach as potentially efficacious. Using 40% as the benchmark for the expected outcome in this trial, ≤9 relapses among 35 patients (observed rate of 26% or less) would be a statistically significant improvement over the fixed benchmark of 40% (one-sided p-value of .04), and the likelihood of making such an observation is 82% (i.e., power) if the true one-year relapse with the proposed regimen is 20%. Additionally, if the observed one-year relapse probability in this trial is lower than 40% and the treatment is not toxic, we will be sufficiently encouraged to consider this approach worthy of further study in a subsequent trial to address potential efficacy in preventing relapse more definitively.

As described above, the relapse rate will be examined and assessed to acquire insights into potential efficacy, but it is unlikely that formal conclusions will be drawn. The results will also be used to set benchmarks for potential future clinical trials aimed at improving anti-leukemic efficacy of T cell therapy. In addition to assessing prevention of relapse, information will be gathered that may be linked to efficacy after adoptive therapy for leukemia. This will include disease characteristics such as cytogenetics, level of expression of WT1 (and potentially other genetic markers), and other concurrent therapies the patient has or is receiving that may affect response (e.g. immuno-suppression). Although the small sample size will most likely preclude detecting statistically significant differences for such parameters, this information will be hypothesis generating and may again serve as a platform for designing future clinical trials.

24.C. <u>Analysis of Anti-leukemic Potential Efficacy (Arm 2)</u>

Relapse of AML and MDS after allogeneic HCT is typically characterized by progressive disease, and >75% of patients will die within one year of the diagnosis of relapse.[127,128] Patients with relapsed AML or MDS (see **Section 7.C** for definition of relapse), who have no evidence of disease after T cell therapy, will be considered complete responders (see **Protocol Appendix D** for response criteria). These patients will be followed to determine the duration of response. Treatment options for relapsed AML or MDS after HCT, e.g. chemotherapy or DLI, are limited by poor response rates and non-durable responses with less than 3% two-year overall survival for patients who relapse within six months after HCT. Durable response can be attained in a few patients who undergo a second HCT, but a second HCT is associated with a treatment-related mortality of ~50%. Using 3% as the benchmark, two or more two-year survivors among 20 treated patients (observed rate of 10% or more) would represent a statistically significant improvement over 3% (one-sided p-value of .03), and, if the true two-year overall survival (OS) with the proposed regimen is 20%, the likelihood of such an observation is 88% (i.e., power). Alternatively, the probability of observing a durable response in >20% of 20 patients (four or more responses among 20) is 0.13 if the true response rate is 0.10, 0.77 if the true response rate is 0.25, and 0.89 if the true response rate is 0.30.

Patients who do not attain a durable complete remission will be evaluated for the nature of their response. Samples will be requested from all patients with detectable disease after T cell therapy. If expression of WT1 and/or HLA-A2 has decreased in purified leukemia cells isolated following T cell therapy, it will be considered suggestive evidence that the T cells exerted immunologic pressure and that the leukemia may have escaped recognition by WT1-specific T cells by down-regulating presentation of antigen. The ability of the infused transduced T cells to recognize $WT1^+$ leukemia cells pre- and post-therapy will be assessed. Limited direct recognition of pre-treatment leukemia cells and/or escape due to decreased expression of HLA-A2 or WT1 will be considered presumptive evidence that the avidity of the transduced T cells may be too low, and that strategies to increase the recognition of leukemic cells by the transferred T cells (e.g. by transducing donor T cells with a synthetic higher affinity TCR), should be explored in subsequent clinical trials if safety from the current clinical trials proves acceptable. By contrast, if the safety data suggests this affinity TCR has reached the limits of acceptable toxicity, then this will be considered the maximal achievable efficacy with this approach. Patients with partial responses (PR) or stable disease (SD) (see **Protocol Appendix D**) will be considered as evidence that adoptive T cell therapy targeting WT1 is potentially effective and that strategies to increase the anti-leukemic activity of the transferred T cells, such as by increasing the number of infusions, cell doses, or affinity may result in improved therapeutic efficacy.

As described above, the response rate in this trial will be examined and assessed for insights into therapeutic promise. In addition to assessing responses to therapy, information will be gathered that may be linked to disease response after adoptive therapy for leukemia. This will include characterizing disease burden and characteristics at time of therapy, time of relapse after HCT, timing of adoptive immunotherapy, level of expression of WT1 (and potentially other genetic markers) by the leukemia, and other concurrent therapies the patient has received or is receiving that may affect response (e.g. immunosuppression). Although the small sample size will most likely preclude detecting significant statistical differences for such parameters, this information will be hypothesis generating and may serve as a platform for designing future clinical trials.

25. TERMINATION OF THE STUDY

The study will be stopped if any of the following events occur:

2. All cohorts have been filled and all patients have completed treatment.
3. Evidence is detected of RCL or leukemia due to insertional mutagenesis.
4. Stopping rules for toxicity have been met. Accrual will be put on hold for discussion with the DSMB regarding a possible change in study design.
5. The PI reserves the right to terminate the study at any time. The FDA may also terminate the study.

26. REFERENCES

1. Fefer A, Sullivan KM, Weiden P, et al. Graft versus leukemia effect in man: the relapse rate of acute leukemia is lower after allogeneic than after syngeneic marrow transplantation. *Progress in clinical and biological research.* 1987;244:401-408.
2. Collins RH, Jr., Shpilberg O, Drobyski WR, et al. Donor leukocyte infusions in 140 patients with relapsed malignancy after allogeneic bone marrow transplantation. *Journal of clinical oncology : official journal of the American Society of Clinical Oncology.* 1997;15(2):433-444.
3. Kolb HJ, Schattenberg A, Goldman JM, et al. Graft-versus-leukemia effect of donor lymphocyte transfusions in marrow grafted patients. *Blood.* 1995;86(5):2041-2050.
4. Cilloni D, Gottardi E, Messa F, et al. Significant correlation between the degree of WT1 expression and the International Prognostic Scoring System Score in patients with myelodysplastic syndromes. *J Clin Oncol.* 2003;21(10):1988-1995.
5. Menssen HD, Renkl HJ, Rodeck U, et al. Presence of Wilms' tumor gene (wt1) transcripts and the WT1 nuclear protein in the majority of human acute leukemias. *Leukemia.* 1995;9(6):1060-1067.
6. Tsuboi A, Oka Y, Ogawa H, et al. Constitutive expression of the Wilms' tumor gene WT1 inhibits the differentiation of myeloid progenitor cells but promotes their proliferation in response to granulocyte-colony stimulating factor (G-CSF). *Leuk Res.* 1999;23(5):499-505.
7. Yamagami T, Sugiyama H, Inoue K, et al. Growth inhibition of human leukemic cells by WT1 (Wilms tumor gene) antisense oligodeoxynucleotides: implications for the involvement of WT1 in leukemogenesis. *Blood.* 1996;87(7):2878-2884.
8. Gao L, Bellantuono I, Elsasser A, et al. Selective elimination of leukemic CD34(+) progenitor cells by cytotoxic T lymphocytes specific for WT1. *Blood.* 2000;95(7):2198-2203.
9. Gao L, Xue SA, Hasserjian R, et al. Human cytotoxic T lymphocytes specific for Wilms' tumor antigen-1 inhibit engraftment of leukemia-initiating stem cells in non-obese diabetic-severe combined immunodeficient recipients. *Transplantation.* 2003;75(9):1429-1436.
10. Ohminami H, Yasukawa M, Fujita S. HLA class I-restricted lysis of leukemia cells by a CD8(+) cytotoxic T-lymphocyte clone specific for WT1 peptide. *Blood.* 2000;95(1):286-293.
11. Oka Y, Elisseeva OA, Tsuboi A, et al. Human cytotoxic T-lymphocyte responses specific for peptides of the wild-type Wilms' tumor gene (WT1) product. *Immunogenetics.* 2000;51(2):99-107.
12. Oka Y, Udaka K, Tsuboi A, et al. Cancer immunotherapy targeting Wilms' tumor gene WT1 product. *J Immunol.* 2000;164(4):1873-1880.
13. Tsuboi A, Oka Y, Ogawa H, et al. Cytotoxic T-lymphocyte responses elicited to Wilms' tumor gene WT1 product by DNA vaccination. *J Clin Immunol.* 2000;20(3):195-202.
14. Xue SA, Gao L, Hart D, et al. Elimination of human leukemia cells in NOD/SCID mice by WT1-TCR gene-transduced human T cells. *Blood.* 2005;106(9):3062-3067.
15. Common Terminology Criteria for Adverse Events (CTCAE) v4.0. Cancer Therapy Evaluation Program; 2009. http://ctep.cancer.gov/protocolDevelopment/electronic_applications/ctc.htm - ctc_40_conversion.
16. Byrd JC, Mrozek K, Dodge RK, et al. Pretreatment cytogenetic abnormalities are predictive of induction success, cumulative incidence of relapse, and overall survival in adult patients with de novo acute myeloid leukemia: results from Cancer and Leukemia Group B (CALGB 8461). *Blood.* 2002;100(13):4325-4336.
17. Slovak ML, Kopecky KJ, Cassileth PA, et al. Karyotypic analysis predicts outcome of preremission and postremission therapy in adult acute myeloid leukemia: a Southwest Oncology Group/Eastern Cooperative Oncology Group Study. *Blood.* 2000;96(13):4075-4083.
18. Lowenberg B, Downing JR, Burnett A. Acute myeloid leukemia. *N Engl J Med.* 1999;341(14):1051-1062.
19. Stoiser B, Knobl P, Fonatsch C, et al. Prognosis of patients with a second relapse of acute myeloid leukemia. *Leukemia.* 2000;14(12):2059-2063.
20. Webb DK. Management of relapsed acute myeloid leukaemia. *Br J Haematol.* 1999;106(4):851-859.
21. Greenberg P, Cox C, LeBeau MM, et al. International scoring system for evaluating prognosis in myelodysplastic syndromes. *Blood.* 1997;89(6):2079-2088.
22. Kantarjian H, O'Brien S, Talpaz M, et al. Outcome of patients with Philadelphia chromosome-positive chronic myelogenous leukemia post-imatinib mesylate failure. *Cancer.* 2007;109(8):1556-1560.
23. Gooley TA, Chien JW, Pergam SA, et al. Reduced mortality after allogeneic hematopoietic-cell transplantation. *N Engl J Med.* 2010;363(22):2091-2101.

24. Appelbaum FR. Allogeneic hematopoietic stem cell transplantation for acute leukemia. *Semin Oncol.* 1997;24(1):114-123.
25. Clift RA, Buckner CD, Appelbaum FR, et al. Allogeneic marrow transplantation during untreated first relapse of acute myeloid leukemia. *Journal of clinical oncology : official journal of the American Society of Clinical Oncology.* 1992;10(11):1723-1729.
26. de Witte T, Hermans J, Vossen J, et al. Haematopoietic stem cell transplantation for patients with myelo-dysplastic syndromes and secondary acute myeloid leukaemias: a report on behalf of the Chronic Leukaemia Working Party of the European Group for Blood and Marrow Transplantation (EBMT). *British journal of haematology.* 2000;110(3):620-630.
27. Deeg HJ, Storer B, Slattery JT, et al. Conditioning with targeted busulfan and cyclophosphamide for hemopoietic stem cell transplantation from related and unrelated donors in patients with myelodysplastic syndrome. *Blood.* 2002;100(4):1201-1207.
28. Walter RB, Pagel JM, Gooley TA, et al. Comparison of matched unrelated and matched related donor myeloablative hematopoietic cell transplantation for adults with acute myeloid leukemia in first remission. *Leukemia : official journal of the Leukemia Society of America, Leukemia Research Fund, UK.* 2010;24(7):1276-1282.
29. Gyurkocza B, Storb R, Storer BE, et al. Nonmyeloablative allogeneic hematopoietic cell transplantation in patients with acute myeloid leukemia. *Journal of clinical oncology : official journal of the American Society of Clinical Oncology.* 2010;28(17):2859-2867.
30. Frassoni F, Barrett AJ, Granena A, et al. Relapse after allogeneic bone marrow transplantation for acute leukaemia: a survey by the E.B.M.T. of 117 cases. *Br J Haematol.* 1988;70(3):317-320.
31. Mortimer J, Blinder MA, Schulman S, et al. Relapse of acute leukemia after marrow transplantation: natural history and results of subsequent therapy. *Journal of clinical oncology : official journal of the American Society of Clinical Oncology.* 1989;7(1):50-57.
32. Choi SJ, Lee JH, Kim S, et al. Treatment of relapsed acute myeloid leukemia after allogeneic bone marrow transplantation with chemotherapy followed by G-CSF-primed donor leukocyte infusion: a high incidence of isolated extramedullary relapse. *Leukemia : official journal of the Leukemia Society of America, Leukemia Research Fund, UK.* 2004;18(11):1789-1797.
33. Inamoto Y, Fefer A, Sandmaier BM, et al. A phase I/II study of chemotherapy followed by donor lymphocyte infusion plus interleukin-2 for relapsed acute leukemia after allogeneic hematopoietic cell transplantation. *Biol Blood Marrow Transplant.* 2011;17(9):1308-1315.
34. Levine JE, Braun T, Penza SL, et al. Prospective trial of chemotherapy and donor leukocyte infusions for relapse of advanced myeloid malignancies after allogeneic stem-cell transplantation. *Journal of clinical oncology : official journal of the American Society of Clinical Oncology.* 2002;20(2):405-412.
35. Bosi A, Laszlo D, Labopin M, et al. Second allogeneic bone marrow transplantation in acute leukemia: results of a survey by the European Cooperative Group for Blood and Marrow Transplantation. *J Clin Oncol.* 2001;19(16):3675-3684.
36. Gratwohl A, Hermans J. Allogeneic bone marrow transplantation for chronic myeloid leukemia. Working Party Chronic Leukemia of the European Group for Blood and Marrow Transplantation (EBMT). *Bone Marrow Transplant.* 1996;17 Suppl 3:S7-9.
37. Gratwohl A, Hermans J, Goldman JM, et al. Risk assessment for patients with chronic myeloid leukaemia before allogeneic blood or marrow transplantation. Chronic Leukemia Working Party of the European Group for Blood and Marrow Transplantation. *Lancet.* 1998;352(9134):1087-1092.
38. Martinelli G, Montefusco V, Testoni N, et al. Clinical value of quantitative long-term assessment of bcr-abl chimeric transcript in chronic myelogenous leukemia patients after allogeneic bone marrow transplantation. *Haematologica.* 2000;85(6):653-658.
39. Reiter E, Greinix HT, Brugger S, et al. Long term follow up after allogeneic stem cell transplantation for chronic myelogenous leukemia. *Bone marrow transplantation.* 1998;22 Suppl 4:S86-88.
40. Arcese W, Goldman JM, D'Arcangelo E, et al. Outcome for patients who relapse after allogeneic bone marrow transplantation for chronic myeloid leukemia. Chronic Leukemia Working Party. European Bone Marrow Transplantation Group. *Blood.* 1993;82(10):3211-3219.
41. Elmaagacli AH, Beelen DW, Schaefer UW. A retrospective single centre study of the outcome of five different therapy approaches in 48 patients with relapse of chronic myelogenous leukemia after allogeneic bone marrow transplantation. *Bone Marrow Transplant.* 1997;20(12):1045-1055.
42. Little M, Holmes G, Walsh P. WT1: what has the last decade told us? *Bioessays.* 1999;21(3):191-202.

43. Hastie ND. Life, sex, and WT1 isoforms--three amino acids can make all the difference. *Cell.* 2001;106(4):391-394.
44. Scharnhorst V, van der Eb AJ, Jochemsen AG. WT1 proteins: functions in growth and differentiation. *Gene.* 2001;273(2):141-161.
45. Nakagama H, Heinrich G, Pelletier J, Housman DE. Sequence and structural requirements for high-affinity DNA binding by the WT1 gene product. *Molecular and cellular biology.* 1995;15(3):1489-1498.
46. Inoue K, Ogawa H, Sonoda Y, et al. Aberrant overexpression of the Wilms tumor gene (WT1) in human leukemia. *Blood.* 1997;89(4):1405-1412.
47. Bergmann L, Maurer U, Weidmann E. Wilms tumor gene expression in acute myeloid leukemias. *Leuk Lymphoma.* 1997;25(5-6):435-443.
48. Majeti R, Becker MW, Tian Q, et al. Dysregulated gene expression networks in human acute myelogenous leukemia stem cells. *Proc Natl Acad Sci U S A.* 2009;106(9):3396-3401.
49. Inoue K, Ogawa H, Yamagami T, et al. Long-term follow-up of minimal residual disease in leukemia patients by monitoring WT1 (Wilms tumor gene) expression levels. *Blood.* 1996;88(6):2267-2278.
50. Patmasiriwat P, Fraizer G, Kantarjian H, Saunders GF. WT1 and GATA1 expression in myelodysplastic syndrome and acute leukemia. *Leukemia.* 1999;13(6):891-900.
51. Sugiyama H. Wilms tumor gene (WT1) as a new marker for the detection of minimal residual disease in leukemia. *Leuk Lymphoma.* 1998;30(1-2):55-61.
52. Tamaki H, Ogawa H, Ohyashiki K, et al. The Wilms' tumor gene WT1 is a good marker for diagnosis of disease progression of myelodysplastic syndromes. *Leukemia.* 1999;13(3):393-399.
53. Rezvani K, Brenchley JM, Price DA, et al. T-cell responses directed against multiple HLA-A*0201-restricted epitopes derived from Wilms' tumor 1 protein in patients with leukemia and healthy donors: identification, quantification, and characterization. *Clin Cancer Res.* 2005;11(24 Pt 1):8799-8807.
54. Rezvani K, Grube M, Brenchley JM, et al. Functional leukemia-associated antigen-specific memory CD8+ T cells exist in healthy individuals and in patients with chronic myelogenous leukemia before and after stem cell transplantation. *Blood.* 2003;102(8):2892-2900.
55. Rezvani K, Yong AS, Savani BN, et al. Graft-versus-leukemia effects associated with detectable Wilms tumor-1 specific T lymphocytes after allogeneic stem-cell transplantation for acute lymphoblastic leukemia. *Blood.* 2007;110(6):1924-1932.
56. Scheibenbogen C, Letsch A, Thiel E, et al. CD8 T-cell responses to Wilms tumor gene product WT1 and proteinase 3 in patients with acute myeloid leukemia. *Blood.* 2002;100(6):2132-2137.
57. Keilholz U, Letsch A, Busse A, et al. A clinical and immunologic phase 2 trial of Wilms tumor gene product 1 (WT1) peptide vaccination in patients with AML and MDS. *Blood.* 2009;113(26):6541-6548.
58. Mailander V, Scheibenbogen C, Thiel E, Letsch A, Blau IW, Keilholz U. Complete remission in a patient with recurrent acute myeloid leukemia induced by vaccination with WT1 peptide in the absence of hematological or renal toxicity. *Leukemia.* 2004;18(1):165-166.
59. Maslak PG, Dao T, Krug LM, et al. Vaccination with synthetic analog peptides derived from WT1 oncoprotein induces T-cell responses in patients with complete remission from acute myeloid leukemia. *Blood.* 2010;116(2):171-179.
60. Oka Y, Tsuboi A, Murakami M, et al. Wilms tumor gene peptide-based immunotherapy for patients with overt leukemia from myelodysplastic syndrome (MDS) or MDS with myelofibrosis. *Int J Hematol.* 2003;78(1):56-61.
61. Oka Y, Tsuboi A, Taguchi T, et al. Induction of WT1 (Wilms' tumor gene)-specific cytotoxic T lymphocytes by WT1 peptide vaccine and the resultant cancer regression. *Proc Natl Acad Sci U S A.* 2004;101(38):13885-13890.
62. Zhou J, Shen X, Huang J, Hodes RJ, Rosenberg SA, Robbins PF. Telomere length of transferred lymphocytes correlates with in vivo persistence and tumor regression in melanoma patients receiving cell transfer therapy. *J Immunol.* 2005;175(10):7046-7052.
63. Oka Y, Tsuboi A, Oji Y, Kawase I, Sugiyama H. WT1 peptide vaccine for the treatment of cancer. *Curr Opin Immunol.* 2008;20(2):211-220.
64. Klebanoff CA, Gattinoni L, Restifo NP. CD8+ T-cell memory in tumor immunology and immunotherapy. *Immunol Rev.* 2006;211:214-224.
65. Rosenberg SA, Yang JC, Restifo NP. Cancer immunotherapy: moving beyond current vaccines. *Nature medicine.* 2004;10(9):909-915.
66. Ho WY, Nguyen HN, Wolfl M, Kuball J, Greenberg PD. In vitro methods for generating CD8+ T-cell clones for immunotherapy from the naive repertoire. *J Immunol Methods.* 2006;310(1-2):40-52.

67. Schumacher TN. T-cell-receptor gene therapy. *Nat Rev Immunol.* 2002;2(7):512-519.
68. Johnson LA, Morgan RA, Dudley ME, et al. Gene therapy with human and mouse T-cell receptors mediates cancer regression and targets normal tissues expressing cognate antigen. *Blood.* 2009;114(3):535-546.
69. Morgan RA, Dudley ME, Wunderlich JR, et al. Cancer regression in patients after transfer of genetically engineered lymphocytes. *Science.* 2006;314(5796):126-129.
70. Kuball J, Dossett ML, Wolfl M, et al. Facilitating matched pairing and expression of TCR chains introduced into human T cells. *Blood.* 2007;109(6):2331-2338.
71. Gustafsson C, Govindarajan S, Minshull J. Codon bias and heterologous protein expression. *Trends Biotechnol.* 2004;22(7):346-353.
72. Scholten KB, Kramer D, Kueter EW, et al. Codon modification of T cell receptors allows enhanced functional expression in transgenic human T cells. *Clin Immunol.* 2006;119(2):135-145.
73. Sallusto F, Geginat J, Lanzavecchia A. Central memory and effector memory T cell subsets: function, generation, and maintenance. *Annu Rev Immunol.* 2004;22:745-763.
74. Berger C, Jensen MC, Lansdorp PM, Gough M, Elliott C, Riddell SR. Adoptive transfer of effector CD8+ T cells derived from central memory cells establishes persistent T cell memory in primates. *J Clin Invest.* 2008;118(1):294-305.
75. Gattinoni L, Klebanoff CA, Palmer DC, et al. Acquisition of full effector function in vitro paradoxically impairs the in vivo antitumor efficacy of adoptively transferred CD8+ T cells. *J Clin Invest.* 2005;115(6):1616-1626.
76. Klebanoff CA, Gattinoni L, Torabi-Parizi P, et al. Central memory self/tumor-reactive CD8+ T cells confer superior antitumor immunity compared with effector memory T cells. *Proc Natl Acad Sci U S A.* 2005;102(27):9571-9576.
77. Vetsika EK, Callan M. Infectious mononucleosis and Epstein-Barr virus. *Expert Rev Mol Med.* 2004;6(23):1-16.
78. Staras SA, Dollard SC, Radford KW, Flanders WD, Pass RF, Cannon MJ. Seroprevalence of cytomegalovirus infection in the United States, 1988-1994. *Clin Infect Dis.* 2006;43(9):1143-1151.
79. Appay V, Dunbar PR, Callan M, et al. Memory CD8+ T cells vary in differentiation phenotype in different persistent virus infections. *Nat Med.* 2002;8(4):379-385.
80. Guerreiro M, Na IK, Letsch A, et al. Human peripheral blood and bone marrow Epstein-Barr virus-specific T-cell repertoire in latent infection reveals distinct memory T-cell subsets. *Eur J Immunol.* 2010;40(6):1566-1576.
81. Iancu EM, Corthesy P, Baumgaertner P, et al. Clonotype selection and composition of human CD8 T cells specific for persistent herpes viruses varies with differentiation but is stable over time. *J Immunol.* 2009;183(1):319-331.
82. Tussey L, Speller S, Gallimore A, Vessey R. Functionally distinct CD8+ memory T cell subsets in persistent EBV infection are differentiated by migratory receptor expression. *Eur J Immunol.* 2000;30(7):1823-1829.
83. van Leeuwen EM, de Bree GJ, Remmerswaal EB, et al. IL-7 receptor alpha chain expression distinguishes functional subsets of virus-specific human CD8+ T cells. *Blood.* 2005;106(6):2091-2098.
84. Anderson BE, McNiff J, Yan J, et al. Memory CD4+ T cells do not induce graft-versus-host disease. *The Journal of clinical investigation.* 2003;112(1):101-108.
85. Chen BJ, Cui X, Sempowski GD, Liu C, Chao NJ. Transfer of allogeneic CD62L- memory T cells without graft-versus-host disease. *Blood.* 2004;103(4):1534-1541.
86. Mielcarek M, Martin PJ, Leisenring W, et al. Graft-versus-host disease after nonmyeloablative versus conventional hematopoietic stem cell transplantation. *Blood.* 2003;102(2):756-762.
87. Greenberg PD. Adoptive T cell therapy of tumors: mechanisms operative in the recognition and elimination of tumor cells. *Adv Immunol.* 1991;49:281-355.
88. Gimmi CD, Morrison BW, Mainprice BA, et al. Breast cancer-associated antigen, DF3/MUC1, induces apoptosis of activated human T cells. *Nat Med.* 1996;2(12):1367-1370.
89. Staveley-O'Carroll K, Sotomayor E, Montgomery J, et al. Induction of antigen-specific T cell anergy: An early event in the course of tumor progression. *Proc Natl Acad Sci U S A.* 1998;95(3):1178-1183.
90. Ettinghausen SE, Moore JG, White DE, Platanias L, Young NS, Rosenberg SA. Hematologic effects of immunotherapy with lymphokine-activated killer cells and recombinant interleukin-2 in cancer patients. *Blood.* 1987;69(6):1654-1660.

91. Rosenberg SA, Yang JC, Topalian SL, et al. Treatment of 283 consecutive patients with metastatic melanoma or renal cell cancer using high-dose bolus interleukin 2. *JAMA.* 1994;271(12):907-913.
92. Miller JS, Tessmer-Tuck J, Pierson BA, et al. Low dose subcutaneous interleukin-2 after autologous transplantation generates sustained in vivo natural killer cell activity. *Biol Blood Marrow Transplant.* 1997;3(1):34-44.
93. Chapuis AG, Thompson JA, Margolin KA, et al. Transferred melanoma-specific CD8+ T cells persist, mediate tumor regression, and acquire central memory phenotype. *Proc Natl Acad Sci U S A.* 2012;109(12):4592-4597.
94. Anderson BE, McNiff J, Yan J, et al. Memory CD4+ T cells do not induce graft-versus-host disease. *J Clin Invest.* 2003;112(1):101-108.
95. Koreth J, Matsuoka K, Kim HT, et al. Interleukin-2 and regulatory T cells in graft-versus-host disease. *N Engl J Med.* 2011;365(22):2055-2066.
96. Chapuis AG, Casper C, Kuntz S, et al. HIV-specific CD8+ T cells from HIV+ individuals receiving HAART can be expanded ex vivo to augment systemic and mucosal immunity in vivo. *Blood.* 2011;117(20):5391-5402.
97. Riddell SR, Watanabe KS, Goodrich JM, Li CR, Agha ME, Greenberg PD. Restoration of viral immunity in immunodeficient humans by the adoptive transfer of T cell clones. *Science.* 1992;257(5067):238-241.
98. Kennedy-Nasser AA, Bollard CM, Heslop HE. Immunotherapy for epstein-barr virus-related lymphomas. *Mediterr J Hematol Infect Dis.* 2009;1(2):e2009010.
99. Qasim W, Gaspar HB, Thrasher AJ. Progress and prospects: gene therapy for inherited immunodeficiencies. *Gene Ther.* 2009;16(11):1285-1291.
100. Bonini C, Grez M, Traversari C, et al. Safety of retroviral gene marking with a truncated NGF receptor. *Nat Med.* 2003;9(4):367-369.
101. Montini E, Cesana D, Schmidt M, et al. Hematopoietic stem cell gene transfer in a tumor-prone mouse model uncovers low genotoxicity of lentiviral vector integration. *Nat Biotechnol.* 2006;24(6):687-696.
102. Durakovic N, Radojcic V, Powell J, Luznik L. Rapamycin promotes emergence of IL-10-secreting donor lymphocyte infusion-derived T cells without compromising their graft-versus-leukemia reactivity. *Transplantation.* 2007;83(5):631-640.
103. Yee C, Thompson JA, Byrd D, et al. Adoptive T cell therapy using antigen-specific CD8+ T cell clones for the treatment of patients with metastatic melanoma: in vivo persistence, migration, and antitumor effect of transferred T cells. *Proceedings of the National Academy of Sciences of the United States of America.* 2002;99(25):16168-16173.
104. Chapuis AG, Ragnarsson GB, Nguyen HN, et al. Transferred WT1-Reactive CD8+ T Cells Can Mediate Antileukemic Activity and Persist in Post-Transplant Patients. *Science translational medicine.* 2013;5(174):174ra127.
105. Nash RA, Gooley T, Davis C, Appelbaum FR. The problem of thrombocytopenia after hematopoietic stem cell transplantation. *Stem cells.* 1996;14 Suppl 1:261-273.
106. Appelbaum FR. Allogeneic hematopoietic stem cell transplantation for acute leukemia. *Seminars in oncology.* 1997;24(1):114-123.
107. de Witte T, Hermans J, Vossen J, et al. Haematopoietic stem cell transplantation for patients with myelo-dysplastic syndromes and secondary acute myeloid leukaemias: a report on behalf of the Chronic Leukaemia Working Party of the European Group for Blood and Marrow Transplantation (EBMT). *British journal of haematology.* 2000;110(3):620-630.
108. Bejanyan N, Weisdorf DJ, Logan BR, et al. Survival of patients with acute myeloid leukemia relapsing after allogeneic hematopoietic cell transplantation: a center for international blood and marrow transplant research study. *Biol Blood Marrow Transplant.* 2015;21(3):454-459.
109. Klebanoff CA, Khong HT, Antony PA, Palmer DC, Restifo NP. Sinks, suppressors and antigen presenters: how lymphodepletion enhances T cell-mediated tumor immunotherapy. *Trends in immunology.* 2005;26(2):111-117.
110. Murali-Krishna K, Ahmed R. Cutting edge: naive T cells masquerading as memory cells. *Journal of immunology (Baltimore, Md : 1950).* 2000;165(4):1733-1737.
111. Cho BK, Rao VP, Ge Q, Eisen HN, Chen J. Homeostasis-stimulated proliferation drives naive T cells to differentiate directly into memory T cells. *The Journal of experimental medicine.* 2000;192(4):549-556.
112. Greenberg PD, Cheever MA, Fefer A. Detection of early and delayed antitumor effects following curative adoptive chemoimmunotherapy of established leukemia. *Cancer research.* 1980;40(12):4428-4432.

113. Vierboom MP, Nijman HW, Offringa R, et al. Tumor eradication by wild-type p53-specific cytotoxic T lymphocytes. *The Journal of experimental medicine.* 1997;186(5):695-704.
114. Lutsiak ME, Semnani RT, De Pascalis R, Kashmiri SV, Schlom J, Sabzevari H. Inhibition of CD4(+)25+ T regulatory cell function implicated in enhanced immune response by low-dose cyclophosphamide. *Blood.* 2005;105(7):2862-2868.
115. Miller JS, Weisdorf DJ, Burns LJ, et al. Lymphodepletion followed by donor lymphocyte infusion (DLI) causes significantly more acute graft-versus-host disease than DLI alone. *Blood.* 2007;110(7):2761-2763.
116. Dudley ME, Wunderlich JR, Robbins PF, et al. Cancer regression and autoimmunity in patients after clonal repopulation with antitumor lymphocytes. *Science (New York, NY).* 2002;298(5594):850-854.
117. Dudley ME, Wunderlich JR, Yang JC, et al. A phase I study of nonmyeloablative chemotherapy and adoptive transfer of autologous tumor antigen-specific T lymphocytes in patients with metastatic melanoma. *Journal of Immunotherapy.* 2002;25(3):243-251.
118. Cameron J Turtle CB, Daniel Sommermeyer, Laila-Aicha Hanafi, Barbara Pender, Emily M Robinson, Katherine Melville, Tanya M Budiarto, Natalia N Steevens, Colette Chaney, Sindhu Cherian, Brent L Wood, Lorinda Soma, Xueyan Chen, Shelly Heimfeld, Michael C Jensen, Stanley R. Riddell, David G Maloney. Anti-CD19 Chimeric Antigen Receptor-Modified T Cell Therapy for B Cell Non-Hodgkin Lymphoma and Chronic Lymphocytic Leukemia: Fludarabine and Cyclophosphamide Lymphodepletion Improves In Vivo Expansion and Persistence of CAR-T Cells and Clinical Outcomes. *Blood cancer journal.* 2015;126:184.
119. Dummer W, Ernst B, LeRoy E, Lee D, Surh C. Autologous regulation of naive T cell homeostasis within the T cell compartment. *Journal of immunology (Baltimore, Md : 1950).* 2001;166(4):2460-2468.
120. Woo EY, Chu CS, Goletz TJ, et al. Regulatory CD4(+)CD25(+) T cells in tumors from patients with early-stage non-small cell lung cancer and late-stage ovarian cancer. *Cancer research.* 2001;61(12):4766-4772.
121. Woo EY, Yeh H, Chu CS, et al. Cutting edge: Regulatory T cells from lung cancer patients directly inhibit autologous T cell proliferation. *Journal of immunology (Baltimore, Md : 1950).* 2002;168(9):4272-4276.
122. Dudley ME, Wunderlich JR, Yang JC, et al. Adoptive cell transfer therapy following non-myeloablative but lymphodepleting chemotherapy for the treatment of patients with refractory metastatic melanoma. *Journal of clinical oncology : official journal of the American Society of Clinical Oncology.* 2005;23(10):2346-2357.
123. Shaffer BC, Le Luduec JB, Forlenza C, et al. Phase II Study of Haploidentical Natural Killer Cell Infusion for Treatment of Relapsed or Persistent Myeloid Malignancies Following Allogeneic Hematopoietic Cell Transplantation. *Biology of blood and marrow transplantation : journal of the American Society for Blood and Marrow Transplantation.* 2016;22(4):705-709.
124. Gunturu KS, Meehan KR, Mackenzie TA, et al. Cytokine working group study of lymphodepleting chemotherapy, interleukin-2, and granulocyte-macrophage colony-stimulating factor in patients with metastatic melanoma: clinical outcomes and peripheral-blood cell recovery. *Journal of clinical oncology : official journal of the American Society of Clinical Oncology.* 2010;28(7):1196-1202.
125. Walter RB, Pagel JM, Gooley TA, et al. Comparison of matched unrelated and matched related donor myeloablative hematopoietic cell transplantation for adults with acute myeloid leukemia in first remission. *Leukemia.*24(7):1276-1282.
126. Gyurkocza B, Storb R, Storer BE, et al. Nonmyeloablative allogeneic hematopoietic cell transplantation in patients with acute myeloid leukemia. *J Clin Oncol.*28(17):2859-2867.
127. Frassoni F, Barrett AJ, Granena A, et al. Relapse after allogeneic bone marrow transplantation for acute leukaemia: a survey by the E.B.M.T. of 117 cases. *Br J Haematol.* 1988;70(3):317-320.
128. Mortimer J, Blinder MA, Schulman S, et al. Relapse of acute leukemia after marrow transplantation: natural history and results of subsequent therapy. *J Clin Oncol.* 1989;7(1):50-57.
129. Cheson BD, Bennett JM, Kopecky KJ, et al. Revised recommendations of the International Working Group for Diagnosis, Standardization of Response Criteria, Treatment Outcomes, and Reporting Standards for Therapeutic Trials in Acute Myeloid Leukemia. *J Clin Oncol.* 2003;21(24):4642-4649.
130. Cheson BD, Greenberg PL, Bennett JM, et al. Clinical application and proposal for modification of the International Working Group (IWG) response criteria in myelodysplasia. *Blood.* 2006;108(2):419-425.
131. Kröger N, Bacher U, Bader P, et al. NCI First International Workshop on the Biology, Prevention, and Treatment of Relapse after Allogeneic Hematopoietic Stem Cell Transplantation: Report from the Committee on Disease-Specific Methods and Strategies for Monitoring Relapse following Allogeneic Stem Cell Transplantation. Part I: Methods, Acute Leukemias, and Myelodysplastic Syndromes. *Biology of Blood and Marrow Transplantation.*16(9):1187-1211.

132. Hughes T, Deininger M, Hochhaus A, et al. Monitoring CML patients responding to treatment with tyrosine kinase inhibitors: review and recommendations for harmonizing current methodology for detecting BCR-ABL transcripts and kinase domain mutations and for expressing results. *Blood.* 2006;108(1):28-37.
133. Hughes TP, Hochhaus A, Branford S, et al. Long-term prognostic significance of early molecular response to imatinib in newly diagnosed chronic myeloid leukemia: an analysis from the International Randomized Study of Interferon and STI571 (IRIS). *Blood.* 116(19):3758-3765.
134. Talpaz M, Shah NP, Kantarjian H, et al. Dasatinib in imatinib-resistant Philadelphia chromosome-positive leukemias. *N Engl J Med.* 2006;354(24):2531-2541.
135. Radich JP. How I monitor residual disease in chronic myeloid leukemia. *Blood.* 2009;114(16):3376-3381.

27. APPENDICES

APPENDIX A

Staging and grading of acute GVHD*

Staging

Skin	1	Rash on <25% of skin
	2	Rash on 25-50% of skin
	3	Rash on >50% of skin
	4	Generalized erythroderma with bullous formation
Liver	1	Bilirubin 2.0-3.0 mg/dl [a]
	2	Bilirubin 3.1-6.0 mg/dl
	3	Bilirubin 6.1-15.0 mg/dl
	4	Bilirubin > 15.0 mg/dl
Gut	1	Diarrhea > 500 mL /day or >280 mL/m ^{2} [b] or persistent nausea [c]
	2	Diarrhea >1000 mL /day or >555 mL/m ^{2}
	3	Diarrhea >1500 mL /day or >833 mL/m ^{2}
	4	Severe abdominal pain with or without ileus

Grade [d]	Skin	Liver	Gut
I	Stage 1-2	None	None
II	Stage 3 or	Stage 1 or	Stage 1
III	-	Stage 2-3 or	Stage 2-4
IV [e]	Stage 4	Stage 4	-

* Przepiorka *et al.* Bone Marrow Transplantation 15:825-8, 1995

[a] Range given as total Bilirubin. Downgrade one stage if an additional cause of elevated bilirubin has been documented

[b] For pediatric patients, the volume of diarrhea should be based on body surface area. Downgrade one stage if an additional cause of diarrhea has been documented.

[c] Persistent nausea with histologic evidence of GVHD in the stomach or duodenum

[d] Criteria for grading given as minimum degree of organ involvement required to confer that grade

[e] Grade IV may also include lesser organ involvement but with extreme decrease in performance status

APPENDIX B

Chronic GVHD-scoring

Mild chronic GVHD involves only 1 or 2 organs or sites (except the lung: see below), with no clinically significant functional impairment (maximum of score 1 in all affected organs or sites). Moderate chronic GVHD involves (1) at least 1 organ or site with clinically significant but no major disability (maximum score of 2 in any affected organ or site) or (2) 3 or more organs or sites with no clinically significant functional impairment (maximum score of 1 in all affected organs or sites). A lung score of 1 will also be considered moderate chronic GVHD. Severe chronic GVHD indicates major disability caused by chronic GVHD (score of 3 in any organ or site). A lung score of 2 or greater will also be considered severe chronic GVHD.

	SCORE 0	SCORE 1	SCORE 2	SCORE 3
PERFORMANCE SCORE: ☐ KPS ECOG LPS	☐ Asymptomatic and fully active (ECOG 0; KPS or LPS 100%)	☐ Symptomatic, fully ambulatory, restricted only in physically strenuous activity (ECOG 1, KPS or LPS 80-90%)	☐ Symptomatic, ambulatory, capable of self-care, >50% of waking hours out of bed (ECOG 2, KPS or LPS 60-70%)	☐ Symptomatic, limited self-care, >50% of waking hours in bed (ECOG 3-4, KPS or LPS <60%)
SKIN<br>*Clinical features:*<br>☐ Maculopapular rash<br>☐ Lichen planus-like features<br>☐ Papulosquamous lesions or ichthyosis<br>☐ Hyperpigmentation<br>☐ Hypopigmentation<br>☐ Keratosis pilaris<br>☐ Erythema<br>☐ Erythroderma<br>☐ Poikiloderma<br>☐ Sclerotic features<br>☐ Pruritus<br>☐ Hair involvement<br>☐ Nail involvement<br>% BSA involved ☐	☐ No Symptoms	☐ <18% BSA with disease signs but NO sclerotic features	☐ 19-50% BSA OR involvement with superficial sclerotic features "not hidebound" (able to pinch)	☐ >50% BSA OR deep sclerotic features "hidebound" (unable to pinch) OR impaired mobility, ulceration or severe pruritus
MOUTH	☐ No symptoms	☐ Mild symptoms with disease signs but not limiting oral intake significantly	☐ Moderate symptoms with disease signs with partial limitation of oral intake	☐ Severe symptoms with disease signs on examination with major limitation of oral intake
EYES<br><br>Mean tear test (mm):<br>☐ >10<br>☐ 6-10<br>☐ ≤5<br>☐ Not done	☐ No symptoms	☐ Mild dry eye symptoms not affecting ADL (requiring eyedrops ≤ 3 x per day) OR asymptomatic signs of keratoconjunctivitis sicca	☐ Moderate dry eye symptoms partially affecting ADL (requiring drops > 3 x per day or punctal plugs), WITHOUT vision impairment	☐ Severe dry eye symptoms significantly affecting ADL (special eyeware to relieve pain) OR unable to work because of ocular symptoms OR loss of vision caused by keratoconjunctivitis sicca
GI TRACT	☐ No symptoms	☐ Symptoms such as dysphagia, anorexia, nausea, vomiting, abdominal pain or diarrhea without significant weight loss (<5%)	☐ Symptoms associated with mild to moderate weight loss (5-15%)	☐ Symptoms associated with significant weight loss >15%, requires nutritional supplement for most calorie needs OR esophageal dilation
LIVER	☐ Normal LFT	☐ Elevated Bilirubin, AP*, AST or ALT <2 x ULN	☐ Bilirubin >3 mg/dl or Bilirubin, enzymes 2-5 x ULN	☐ Bilirubin or enzymes > 5 x ULN

	SCORE 0	SCORE 1	SCORE 2	SCORE 3
LUNGS† FEV1 ☐	☐ No symptoms	☐ Mild symptoms (shortness of breath after climbing one flight of steps)	☐ Moderate symptoms (shortness of breath after walking on flat ground)	☐ Severe symptoms (shortness of breath at rest; requiring O_2)
DLCO ☐	☐ FEV1 > 80% OR LFS=2	☐ FEV1 60-79% OR LFS 3-5	☐ FEV1 40-59% OR LFS 6-9	☐ FEV1 ≤39% OR LFS 10-12
JOINTS AND FASCIA	☐ No symptoms	☐ Mild tightness of arms or legs, normal or mild decreased range of motion (ROM) AND not affecting ADL	☐ Tightness of arms or legs OR joint contractures, erythema thought due to fasciitis, moderate decrease ROM AND mild to moderate limitation of ADL	☐ Contractures WITH significant decrease of ROM *AND* significant limitation of ADL (unable to tie shoes, button shirts, dress self etc.)
GENITAL TRACT	☐ No symptoms	☐ Symptomatic with mild signs on exam AND no effect on coitus and minimal discomfort with gynecologic exam	☐ Symptomatic with moderate signs on exam AND with mild dyspareunia or discomfort with gynecologic exam	☐ Symptomatic WITH advanced signs (stricture, labial agglutination or severe ulceration) AND severe pain with coitus or inability to insert vaginal speculum

Other indicators, clinical manifestations or complications related to chronic GVHD (check all that apply and assign a score to its severity (0-3) based on its functional impact where applicable (none - 0,mild -1, moderate -2, severe - 3)

Esophageal stricture or web___ Pericardial Effusion___ Pleural Effusion(s)___

Ascites (serositis)___ Nephrotic syndrome___ Peripheral Neuropathy___

Myasthenia Gravis___ Cardiomyopathy___ Eosinophilia > 500µl___

Polymyositis___ Cardiac conduction defects___ Coronary artery involvement___

Platelets <100,000/µl ___ Progressive onset___

OTHERS: Specify:____

Organ scoring of chronic GVHD. *AP may be elevated in growing children, and not reflective of liver dysfunction. †Pulmonary scoring should be performed using both the symptom and pulmonary function testing (PFT) scale whenever possible. When discrepancy exists between pulmonary symptom or PFT scores the higher value should be used for final scoring. Scoring using the Lung Function Score (LFS) is preferred, but if DLCO is not available, grading using FEV1 should be used. The LFS is a global assessment of lung function after the diagnosis of bronchiolitis obliterans has already been established [29]. The percent predicted FEV1 and DLCO (adjusted for hematocrit but not alveolar volume) should be converted to a numeric score as follows:>80% =1; 70-79% = 2; 60-69% = 3; 50-59% = 4; 40-49% = 5; <40% =6. The LFS _ FEV1 score _ DLCO score, with a possible range of 2-12. GVHD indicates graft versus host disease; ECOG, Eastern Cooperative Oncology Group; KPS, Karnofsky Performance Status; LPS, Lansky Performance Status; BSA, body surface area; ADL, activities of daily living; LFTs, liver function tests; AP, alkaline phosphatase; ALT, alanine aminotransferase; AST, aspartate aminotransferase; ULN, upper limit of normal.

From: Filipovich AH, et. al:Biology of Blood and Marrow Transplant. 2005 Dec;11(12):945-56.National Institutes of Health consensus development project on criteria for clinical trials in chronic graft-versus-host disease: I. Diagnosis and staging working group report.

APPENDIX C
Donor Criteria for Leukapheresis

Clinical parameter	Criterion for healthy donors
Donor Questionnaire for Allogeneic Apheresis Product intended for infusion in recipient	Required—donors deemed suitable for apheresis for therapeutic purposes will be eligible for research apheresis under this protocol
Virology Testing	Required
Blood Pressure	Systolic: 90-180<br>Diastolic: 50-100
Pulse	50-110 and Regular
Weight	≥50 Kg
Temperature	≤37.5C (99.5F)
WBC	≥ 3,000
HCT	Men: ≥35%<br>Women: ≥ 34%
Platelets	>130,000
Cardiovascular complications during therapeutic apheresis specifically, any chest pain, clinical evidence of cardiac ischemia, or significant arrhythmia	No cardiovascular complications unless deemed safe by cardiovascular consult service after appropriate medical management.

APPENDIX D

Response criteria:

Acute Myeloid Leukemia (AML) and Myelodysplastic Syndrome (MDS) [129-131]

Criteria	Complete Response (CR)	Partial Response (PR)	Stable Disease (SD)
**Morphologic**	Bone Marrow blasts <5%;<br>no blasts with Auer rods; no extramedullary disease<br><br>Transfusion independent<br>Platelets ≥100,000/μl<br>Absolute neutrophil count >1000/μl<br>(CRi: incomplete hematologic recovery<br>CRp: incomplete platelet recovery)	Decrease of at least 50% in the percentage of blasts to 5-25% in the bone marrow and the normalization of blood counts as for CR.<br>(PRi and PRp if incomplete hematologic or platelet recovery)	Failure to achieve at least PR, but no evidence of progression for >4 weeks.
**Cytogenetic**	Disappearance of previous cytogenetic abnormality	≥50% reduction of abnormal metaphases	See above
**FISH**	Disappearance of previous FISH abnormality	As defined by individual assay.	See above
**Molecular**	Disappearance of molecular mutation/marker	As defined by individual assay.	See above
**Flow cytometric**	Disappearance of cells with aberrant phenotype.	≥10 fold reduction in percentage of leukemic cells if initial percentage of BM blasts is ≤5%. Otherwise follow morphologic criteria.	See above

Chronic Myeloid Leukemia (CML) [132-135]

1) Patients with CML beyond chronic phase at the beginning of T cell therapy:
Hematologic response: Complete Hematologic Response (CHR): WBC <10,000/μl and platelet count <450,000/μl maintained for at least 4 weeks.

2) Patients with CML in chronic phase. It is considered a response when patients reach a disease category indicating lower disease burden (example minimal response reaching the level

of minor response after T cell therapy). Complete response is considered attaining complete molecular response (CMR):

Disease categories:
1. >96% Ph+ cells (Full cytogenetic relapse)
2. 66-95% Ph+ cells
3. 36-65% Ph+ cells
4. 1-35% Ph+ cells
5. No Ph+ cells but > MMR (BCR-ABL>0.1% on the international scale)
6. No BCR-ABL transcripts by quantitative PCR **(CMR).**

3) Response in all CML patients receiving T cell therapy will also be assessed by quantitation of BCR-ABL transcripts by quantitative PCR in peripheral blood

APPENDIX E

Potential Adverse Events Associated or Expected with Hematopoietic Cell Transplantation

1. Opportunistic infections, including viral and fungal infections, can results in severe pulmonary, neurologic, hepatic and other organ dysfunction, and possible death.

2. Gastrointestinal toxicity. Nausea and vomiting can be anticipated during the entire course of ablative therapy. Mucositis and diarrhea should be expected. Prednisone can cause GI bleeding.

3. Cardiac toxicity. Cardiotoxicity (congestive heart failure, pericardial effusion, EKG changes) is uncommonly associated with chemotherapy agents and TBI and these sequelae may prove lethal.

4. Pulmonary toxicity. Diffuse interstitial pneumonitis of unknown etiology occurs with some regularity after BMT and interstitial fibrosis occurs much more rarely. Both are well-described complications of intensive chemotherapy and TBI regimens and may prove lethal.

5. Hepatic toxicity. Veno-occlusive disease of the liver is a common toxicity of high-dose chemoradiotherapy and may result in death. Tacrolimus may cause elevation of ALT/AST.

6. Renal dysfunction. Chemoradiotherapy may uncommonly cause renal dysfunction. More commonly, nephrotoxicity results from tacrolimus and generally responds to dose reduction. Rarely, idiopathic or calcineurin inhibitor-associated hemolytic-uremic syndrome may occur and may be progressive and fatal. A syndrome of moderate renal insufficiency and hemolysis has been seen 5-7 months post HSCT after intensive conditioning plus TBI.

7. Hemorrhagic cystitis, manifested either as gross or microscopic hematuria, is a common toxicity after high-dose chemoradiotherapy, but usually associated with regimens that include cyclophosphamide. Hemorrhagic cystitis may predispose to a long-term increased risk of bladder cancer.

8. Central nervous system toxicity. Radiation and chemotherapy can cause CNS toxicity, including seizures, depressed mental status, or leukoencephalopathy. Calcineurin inhibitors can cause seizures or other CNS toxicity.

9. Marrow aplasia. Severe neutropenia, thrombocytopenia, and anemia, is expected to occur for a period of 7 to 42 days following infusion of marrow. Transfusion of platelets and red blood cells is expected as supportive care. Transfusion of blood products may be associated with acquisition of HIV or a hepatitis virus. Neutropenia may increase the risk for acquiring serious infection. Thrombocytopenia may increase the risk of life-threatening hemorrhage. Hemorrhagic or infectious complications during the expected period of aplasia may result in death.

10. Miscellaneous. Alopecia and sterility are expected complications of the program as a whole. Cataract development is possible after TBI and/or steroids. Deficiencies of growth hormone, thyroid hormone, and sex hormones are possible after TBI. Calcineurin inhibitors can cause transient gingival hyperplasia, tremor, seizure, hypertension, headache, dysesthesia and hirsutism. Steroid therapy can also contribute to fluid retention, easy bruising, hypertension,

aseptic necrosis of bone and increased susceptibility to infection. Hospitalization during conditioning and recovery period is expected to be 5-9 weeks in duration.

APPENDIX F

Arm 2 (new cohort)		Event	History and PE[8]	Chemistry Battery/ Urinalysis	CBC w/ differential	Recipient Battery	GVHD Assessment[1]	HLA*A 0201 WT1 expression EBV Serology[3]	Research Blood[9] 10mL EDTA Purple 60mL ACD Yellow	Bone Marrow Aspirate[2,9] 2mL EDTA Purple 5mL ACD Yellow
Screening		Evaluation for study participation	X	X	X	X	X	X		
Leukapheresis		Donor leukapheresis				X[11]				
Re-induction										
Day -7[6]		Evaluate count recovery			X					
Lympho-depletion[16]	Day -4	Cyclophosphamide $300mg/m^2$ Fludarabine $30mg/m^2$								
	Day -3									
	Day -2									
Pre-Treatment[4,5]		Treatment Eligibility	X	X	X		X		X	X[7]
Week 0 (Infusion 1)	Day 0	T cell infusion ($1x10^{10}$ cells/m^2)	X	X	X				X	
	Day +1	Evaluations post T cell infusion	X	X	X				X	
	Day +4			X	X				X	
Week 1	Day +7		X	X	X				X	
Week 2	Day +14		X	X	X				X	
Week 3	Day +21		X	X	X				X	
Week 4	Day +28		X	X	X				X	
Week 8[10]	Day +56		X	X	X				X	X[7, 13]
If second T cell infusion is delayed beyond 8 weeks, an interim H&P is to be completed at 3 months and every 3 months until second infusion or taken off study										
Day -7[6]		Evaluate count sufficiency			X					
Pre-Treatment[4,5]		Treatment Eligibility	X	X	X		X		X	X[7]
Week 0 (Infusion 2)	Day 0	T cell infusion ($1x10^{10}$ cells/m^2) IL-2 Administration	X	X	X				X	

		250,000 U/m² BID for 14 days								
Arm 2 (new cohort)		Event	History and PE[8]	Chemistry Battery/ Urinalysis	CBC w/ differential	Recipient Battery	GVHD Assessment[1]	HLA*A 0201 WT1 expression EBV Serology[3]	Research Blood[9] 10mL EDTA Purple 60mL ACD Yellow	Bone Marrow Aspirate[2,9] 2mL EDTA Purple 5mL ACD Yellow
Week 0 (Infusion 2)	Day +1	IL-2 Administration 250,000 U/m² BID for 14 days[15]	X	X	X				X	
	Day +4								X	
Week 1	Day +7		X	X	X				X	
Week 2	Day +14		X	X	X				X	
Week 3	Day +21	Evaluations post-T cell infusion	X	X	X				X	X
Week 4	Day +28		X	X	X		X		X	X
Week 6	Day +42		X	X	X				X	X
Week 8 (month 2)	Day +56		X	X	X				X	X
Week 12 (month 3)	Day +84		X	X	X				X	X
Week 24 (month 6)	Day +168		X	X	X				X	
Year 1	Long-Term Follow-Up[14]		X						X	

1) Provider to complete study-specific GVHD Assessment Form (15.G.3; **Appendix B**)
2) To be collected at every clinical bone marrow procedure (**15.H**); 10mL EDTA purple top whole blood is to be collected along with bone marrow aspirate research samples
3) EBV serology is to be assessed for the final 5 patients on Arm 2 (new cohort)
4) Must be performed $\leq$ 30 days of T cell infusion (**15.C**)
5) Chest X-ray and pulmonary function tests are to be completed if clinically indicated
6) Serum or urine pregnancy test to be performed $\leq$ 14 days of T cell infusion
7) Must be performed $\leq$ 21 days of T cell infusion and as close as possible to day 0 (**15.H.2.a**)
8) Karnofsky or Lansky performance status to be recorded at each clinic visit
9) As defined in section **15.F.1.d and 15.F.1.e**; refer to section **15.F.2** for patients 30-50 kg and **15.F.3** for patients 15-30kg
10) If T cell infusion is delayed >8 weeks, efforts will be made to obtain an interim H&P at 8 weeks, 3 months, then every 3 months until second infusion is administered or patient is taken off study (**15.G.1.a.i**).
11) Must be performed $\leq$ 30 days prior to leukapheresis
12) To be collected 3-5 days after last dose of IL-2 (**15.H.2.a**)
13) An additional "interim" restaging bone marrow may be requested if second infusion is delayed >4 weeks
14) To be performed q1 year for 15 years (**15.I.2**)
15) First dose of IL-2 is to be administered 2-4 hours after second T cell infusion (**14.B**)
16) Standard administration guidelines are to be followed for cyclophosphamide and fludarabine (**14.B.d.1**)
