# Supplementary material for: A phase I/II trial of WT1-specific TCR gene therapy for patients with acute myeloid leukemia and active disease post-allogeneic hematopoietic cell transplantation: skewing towards NK-like phenotype impairs T cell function and persistence
Source: Nat Commun. 2025 Jun 5;16:5214. doi: 10.1038/s41467-025-60394-0 (PMC12141728; doi:10.1038/s41467-025-60394-0)
Supplement: Supplementary file 2 — Reporting Summary [file 41467_2025_60394_MOESM2_ESM.pdf]

nature portfolio

Corresponding author(s): Aude Chapuis

Last updated by author(s): Apr 17, 2025

Reporting Summary

Nature Portfolio wishes to improve the reproducibility of the work that we publish. This form provides structure for consistency and transparency in reporting. For further information on Nature Portfolio policies, see our Editorial Policies and the Editorial Policy Checklist.

Statistics

For all statistical analyses, confirm that the following items are present in the figure legend, table legend, main text, or Methods section.

n/a	Confirmed	
☐	☒	The exact sample size (n) for each experimental group/condition, given as a discrete number and unit of measurement
☐	☒	A statement on whether measurements were taken from distinct samples or whether the same sample was measured repeatedly
☐	☒	The statistical test(s) used AND whether they are one- or two-sided<br>*Only common tests should be described solely by name; describe more complex techniques in the Methods section.*
☐	☒	A description of all covariates tested
☐	☒	A description of any assumptions or corrections, such as tests of normality and adjustment for multiple comparisons
☐	☒	A full description of the statistical parameters including central tendency (e.g. means) or other basic estimates (e.g. regression coefficient) AND variation (e.g. standard deviation) or associated estimates of uncertainty (e.g. confidence intervals)
☐	☒	For null hypothesis testing, the test statistic (e.g. F, t, r) with confidence intervals, effect sizes, degrees of freedom and P value noted<br>*Give P values as exact values whenever suitable.*
☒	☐	For Bayesian analysis, information on the choice of priors and Markov chain Monte Carlo settings
☒	☐	For hierarchical and complex designs, identification of the appropriate level for tests and full reporting of outcomes
☒	☐	Estimates of effect sizes (e.g. Cohen's d, Pearson's r), indicating how they were calculated

Our web collection on statistics for biologists contains articles on many of the points above.

Software and code

Policy information about availability of computer code

Data collection

Provide a description of all commercial, open source and custom code used to collect the data in this study, specifying the version used OR state that no software was used.

Data analysis

- Excel
- FlowJo
- R
- Python
- See methods section for complete details

For manuscripts utilizing custom algorithms or software that are central to the research but not yet described in published literature, software must be made available to editors and reviewers. We strongly encourage code deposition in a community repository (e.g. GitHub). See the Nature Portfolio guidelines for submitting code & software for further information.

Data

Policy information about availability of data

All manuscripts must include a data availability statement. This statement should provide the following information, where applicable:

- Accession codes, unique identifiers, or web links for publicly available datasets
- A description of any restrictions on data availability
- For clinical datasets or third party data, please ensure that the statement adheres to our policy

The gene expression data, including de-identified BAM files and count matrices, generated in this study have been deposited in the NCBI Gene Expression Omnibus (GEO) under accession number GSE285214. Previously published mass cytometry data analyzed in this manuscript are available on Zenodo. Previously published AML scRNAseq data analyzed in this study are available from the European Genome-Phenome Archive (EGA) (https://ega-archive.org) under the accession numbers EGAS50000000357, EGAS00001004444 and EGAS00001004894; the NCBI's Database of Genotypes and Phenotypes (dbGaP; https://www.ncbi.nlm.nih.gov/gap) under accession number phs003015.v1.p1; and the GEO repository under accession numbers GSE128933 and GSE185381. Previously published solid tumor scRNAseq data are available in the GEO repository under accession number GSE215121 (melanoma), GSE148071 (lung), and GSE211644 (pancreas).
The remaining data ara available within the Supplementary Information, or Source Data file.

Research involving human participants, their data, or biological material

Policy information about studies with human participants or human data. See also policy information about sex, gender (identity/presentation), and sexual orientation and race, ethnicity and racism.

Reporting on sex and gender	Sex and age were not considered in the study design, as the sample size was limited and exploratory in nature. Accordingly, no sex- or age-based analyses were performed. The sex and age of participants were recorded based on clinical documentation and are reported in aggregate. Consent to specifically report or share individual-level data, that could potentially identify the patients based on age, sex, institution of treatment and disease was not obtained.
Reporting on race, ethnicity, or other socially relevant groupings	Race and ethnicity were not analyzed in relation to the study outcomes. The focus of this study was on clinical and biological factors related to acute myeloid leukemia and its treatment, and race and ethnicity were not included as primary factors in the analysis.
Population characteristics	Detailed characteristics of treated patients are described in Table 1 and supplementary Table S1 of the manuscript.
Recruitment	All HLA A*0201+ participants were identified through the Clinical Coordinator's Office, which tracks all patients referred to Fred Hutchinson Cancer Center for allogeneic stem cell transplantation. The study was presented to potential participants during their Arrival Conference on the Transplant Service by the Attending Physician, who was not part of the research team. The Attending Physicians rotate on a monthly basis, and the Clinical Coordinators were also not involved in the research team. If a patient expressed interest in participating, they signed a Screening Informed Consent to assess eligibility. If eligible and still interested, they then signed a Treatment Informed Consent. Following this, they proceeded with their allogeneic transplantation, and a portion of the donor's G-CSF-mobilized cells was used to generate the infused cells as soon as indicated according to the protocol.
Ethics oversight	The study was conducted in compliance with all relevant ethical regulations and received approval from the Institutional Review Board at Fred Hutchinson Cancer Center. The study was conducted in accordance with the Declaration of Helsinki

Note that full information on the approval of the study protocol must also be provided in the manuscript.

Field-specific reporting

Please select the one below that is the best fit for your research. If you are not sure, read the appropriate sections before making your selection.

☒ Life sciences ☐ Behavioural & social sciences ☐ Ecological, evolutionary & environmental sciences

For a reference copy of the document with all sections, see nature.com/documents/nr-reporting-summary-flat.pdf

Life sciences study design

All studies must disclose on these points even when the disclosure is negative.

Sample size	The sample size for TTCR-C4-treated patients (n=15) was not determined through formal power calculations. Instead, it was based on feasibility, with the goal of providing descriptive data to assess whether further studies were warranted and to evaluate the acceptability of toxicity levels.
Data exclusions	All the patients who received the infusion product TTCR-C4 underwent immune-monitoring
Replication	The study's key experiments were performed in a single cohort of patients (n=15). Due to the feasibility-driven design and sample size constraints, no formal replication of the results in an independent cohort was conducted. However, the results were analyzed across multiple timepoints and were consistent within the cohort. For in silico validation, data from publicly available datasets were compared to assess the

	generalizability of the observed trends.
Randomization	Not applicable - no randomization was performed
Blinding	All clinical data (e.g. lymphocyte counts, platelets, hematocrit, marrow immunohistochemistry, clinical flow cytomtry, clinical cytogenetics) were taken from clinical lab reports and therefore blinded. For all other laboratory studies care was taken to perform all manipulations on the same day and under the same experimental conditions. Patient group allocation could not be blinded as the screening and logistics necessary to infuse patients with TTCR-C4 required coordination between multiple groups.

Reporting for specific materials, systems and methods

We require information from authors about some types of materials, experimental systems and methods used in many studies. Here, indicate whether each material, system or method listed is relevant to your study. If you are not sure if a list item applies to your research, read the appropriate section before selecting a response.

Materials & experimental systems

n/a	Involved in the study	
☐	☒	Antibodies
☐	☒	Eukaryotic cell lines
☒	☐	Palaeontology and archaeology
☒	☐	Animals and other organisms
☐	☒	Clinical data
☒	☐	Dual use research of concern
☒	☐	Plants

Methods

n/a	Involved in the study	
☒	☐	ChIP-seq
☐	☒	Flow cytometry
☒	☐	MRI-based neuroimaging

Antibodies

Antibodies used	Spectral flow cytometry was performed on a 5-laser Cytek Aurora (Fig. 2A-F). Cells were stained with fluorochrome-conjugated antibodies against surface and intracellular markers, including TIGIT (A15153G, BV421, BioLegend), CCR7 (3D12, BB700, BD Biosciences), Ki-67 (B56, BV480, BD Biosciences), CD57 (NK-1, BUV395), CD45RA (HI100, BV570, BioLegend), CD27 (O323, BV605, BioLegend), CD95/FAS (DX29, BV650, BioLegend), CD127/IL-7Ra (A019D5, BV711, BioLegend), CD28 (CD28.2, BV785, BioLegend), T-Bet (4B10, KIRAVIA Blue 520, Cell Signaling Technology), CD8 (RPA-T8, BUV737), TCF1/TCF7 (C63D9, PE), PD-1 (EH12.2H7, PE-Dazzle 594, BioLegend), CD69 (FN50, PE-Cy5, BioLegend), Granulysin (DH2, PE-Cy7), KLRG1 (Z7-205.rMAb, PerCP-eFluor 710), Granzyme K (G3H69, RB780, BD Biosciences), Tetramer (APC, Fred Hutch in house production), Granzyme B (GB11, Alexa Fluor 700, BD Biosciences), CD38 (HIT2, APC-Fire 810, BioLegend), CD3 (UCHT1, APC-Fire 750, BioLegend), TIM3 (F38-2E2, PE-Fire 810, BioLegend), and viability dye (LD Blue, Invitrogen). A dump channel excluded myeloid using antibodies to CD33 (WM53, BV510, BioLegend) and CD14 (63D3, BV510, BioLegend). All flow cytometry staining procedures were validated and performed as previously described in Mazziotta, F., et al. Blood (2024), and Rutella, S., et al. J Clin Invest (2022).<br><br>Flow cytometry and intracellular cytokine staining (Fig. 2G): TTCR-C4 in infusion products and PBMCs post-transfer were identified by binding to the A*0201/WT1126-134 tetramer APC (Fred Hutch in house production), and analyzed by flow cytometry after staining with fluorochrome-conjugated mAbs to CD14 BV510 (clone M5E1, BioLegend), live/dead Fixable Aqua Dead Cell Stain (Invitrogen) (AVID/CD14 dump channel), CD4 BUV395 (clone SK3, BD Biosciences), CD3 BV570 (clone UCHT1, BioLegend), CD8 BV711 (clone RPA-T8, BD Biosciences), IFNy V450 (clone B27, BD Biosciences). Intracellular cytokine staining: Intracellular cytokine staining and stimulations were performed as previously described and validated (Moncunill, G. et al., Cytometry. Part A : the journal of the International Society for Analytical Cytology 2014), with the following modifications: tetramers specific for HLA A*0201/WT126-134 (FHCRC in house production, APC) were added at 1:1000 and 1:400 respectively in 50mcl R10 and incubated 30 minutes at room temperature before the addition of the stimulation cocktail containing the WT1126-134 peptide in R10 at a final per peptide concentration of 1 microg/mL. Cells were analyzed on an LSRII (Becton Dickinson) using FACS-Diva software.
Validation	Validation was performed through titration using PBMCs to determine the optimal antibody concentration for inclusion in the final staining cocktail, and/or based on information provided in the manufacturer's datasheets.

Eukaryotic cell lines

Policy information about cell lines and Sex and Gender in Research

Cell line source(s)	K562 tumor cell line (CCL-243), was obtained from the ATCC
Authentication	Cells were purchased from ATCC and were not further authenticated
Mycoplasma contamination	K562 cell line tested negative for mycoplasma.
Commonly misidentified lines (See ICLAC register)	No commonly misidentified cell lines were used.

Clinical data

Policy information about clinical studies

All manuscripts should comply with the ICMJEguidelines for publication of clinical research and a completedCONSORT checklist must be included with all submissions.

Clinical trial registration	NCT01640301
Study protocol	The study protocol is reported in a supplementary note in Supplementary information
Data collection	All HLA-A*0201+ participants were identified through the Clinical Coordinator's Office, which tracks all patients referred to Fred Hutch for allogeneic transplantation. The Attending Physician on the Transplant Service (not involved in the research team) was notified and presented the study to potential participants during their Arrival Conference. If interested, patients signed a screening informed consent to formally determine eligibility. Those meeting screening criteria and remaining interested signed a treatment informed consent. Patients then proceeded with allogeneic transplantation. A portion of the donor's G-CSF–mobilized collection was used to generate the cell product, which was infused post-transplant. Because the study was not randomized and required active participant willingness (e.g., extended hospitalization post-transplant to receive cells), we acknowledge the possibility of self-selection bias that could have influenced results.
Outcomes	Primary endpoint was safety; secondary endpoints were persistence and efficacy

Plants

Seed stocks	*Report on the source of all seed stocks or other plant material used. If applicable, state the seed stock centre and catalogue number. If plant specimens were collected from the field, describe the collection location, date and sampling procedures.*
Novel plant genotypes	*Describe the methods by which all novel plant genotypes were produced. This includes those generated by transgenic approaches, gene editing, chemical/radiation-based mutagenesis and hybridization. For transgenic lines, describe the transformation method, the number of independent lines analyzed and the generation upon which experiments were performed. For gene-edited lines, describe the editor used, the endogenous sequence targeted for editing, the targeting guide RNA sequence (if applicable) and how the editor was applied.*
Authentication	*Describe any authentication procedures for each seed stock used or novel genotype generated. Describe any experiments used to assess the effect of a mutation and, where applicable, how potential secondary effects (e.g. second site T-DNA insertions, mosiacism, off-target gene editing) were examined.*

Flow Cytometry

Plots

Confirm that:

- [x] The axis labels state the marker and fluorochrome used (e.g. CD4-FITC).
- [x] The axis scales are clearly visible. Include numbers along axes only for bottom left plot of group (a 'group' is an analysis of identical markers).
- [x] All plots are contour plots with outliers or pseudocolor plots.
- [x] A numerical value for number of cells or percentage (with statistics) is provided.

Methodology

Sample preparation	Peripheral Blood Mononuclear Cells (PBMC) and bone marrow aspirates: White blood cells from the described patients were collected with standard phlebotomy/marrow aspirates, isolated with standard Ficoll gradient, and viably cryopreserved. Infusion product: Please see methods section for full description of preparation of therapeutic product. For all flow cytometry, cryopreserved PBMCs or marrow lymphocytes were thawed and rested overnight in RPMI supplemented with 10% fetal bovine serum (FBS) (R10) before staining/assay performance.
Instrument	5-laser Cytek Aurora and LSRII (Becton Dickinson) using FACS-Diva software
Software	-FlowJo v10<br>-R
Cell population abundance	At least 1 x 10E6 events gated on the lymphocyte population were acquired for each sample.
Gating strategy	*Describe the gating strategy used for all relevant experiments, specifying the preliminary FSC/SSC gates of the starting cell population, indicating where boundaries between "positive" and "negative" staining cell populations are defined.*

- [x] Tick this box to confirm that a figure exemplifying the gating strategy is provided in the Supplementary Information.
